# NIR-II photothermal CSGGT hydrogel for antibacterial therapy and accelerated healing of infected wounds

**DOI:** 10.1039/d6ra06536h

**Published:** 2026-09-01

**Authors:** Yani Zou, Yingao Jiao, Jiehao Zhou, Helin Xu, Qiqi Liu, Dengyao Gao, Yan Gao, Yihong Yang, Yanlei Liu, Hongbo Liu

**Affiliations:** a Department of Radiation Oncology, Fuyang People's Hospital Affiliated to Anhui Medical University 501 Sanqing Road, Yingzhou District Fuyang Anhui 236000 China; b Institute of Intelligent Health Diagnosis and Treatment, School of Automation and Intelligent Sensing, Shanghai Jiao Tong University 800 Dongchuan Road Shanghai 200240 People's Republic of China liuyanlei@sjtu.edu.cn; c Department of Emergency Medicine, Fuyang People's Hospital Affiliated to Anhui Medical University 501 Sanqing Road, Yingzhou District Fuyang Anhui 236000 China 15856806657@163.com liuhongbo@bbmu.edu.cn

## Abstract

Infected skin wounds demand integrated strategies for rapid bacterial clearance and tissue repair. We developed a β-glucan–gelatin–tannic acid–Cu_9_S_8_ (CSGGT) composite hydrogel combining NIR-II photothermal therapy (1064 nm) with a bioactive physical network. The hydrogel exhibited self-healing, injectability, rapid swelling, and excellent biocompatibility. Cu_9_S_8_ nanoparticles (photothermal conversion efficiency: 43.87%) enabled efficient bacterial killing (≥2.5-log reduction for *S. aureus* and *E. coli*) under laser irradiation. In an *S. aureus*-infected murine full-thickness wound model, CSGGT + NIR achieved near-complete closure by day 12, with robust re-epithelialization, regenerated skin appendages, dense collagen deposition, and reduced inflammatory infiltration, significantly outperforming all controls. No systemic toxicity was observed. This work presents a combined therapeutic platform that merges immediate photothermal antibacterial action with sustained pro-healing microenvironment support, offering a promising non-antibiotic solution for infected wound management.

## Abstract 2

Infected skin wounds demand integrated strategies for rapid bacterial clearance and tissue repair.

## Graphical abstract

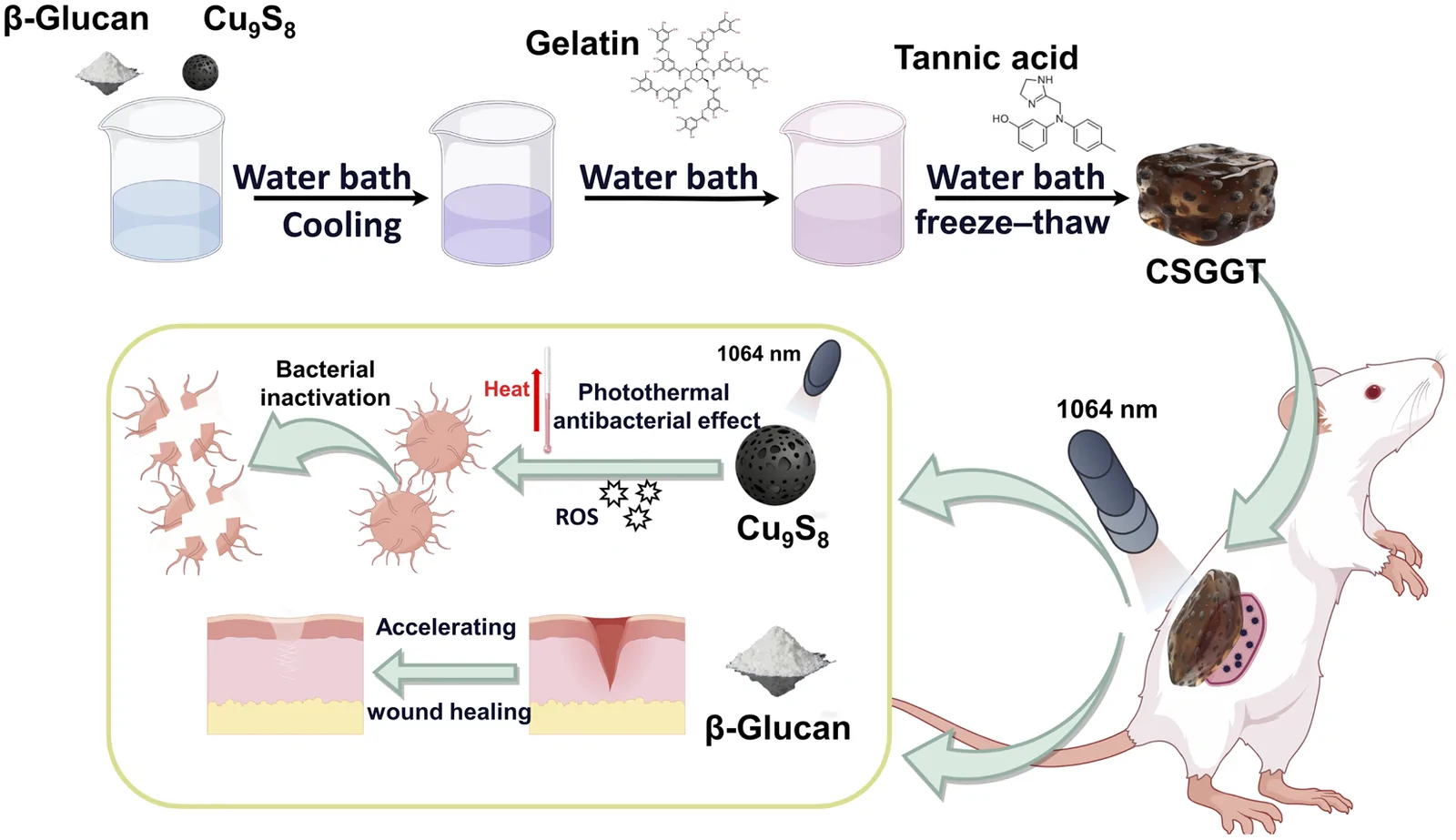

## 1. Introduction

Skin wound infection remains a formidable clinical challenge.^1^ Bacterial colonization and the overuse of antibiotics have accelerated the spread of drug-resistant pathogens, while persistent inflammatory responses trigger oxidative tissue damage and disrupt endogenous regenerative programs, resulting in delayed wound healing.^2^ Conventional treatment strategies, such as silver-containing dressings^3^ and systemic antibiotic therapy,^4^ are widely employed but exhibit critical limitations.^5^ These include the accelerated emergence of drug-resistant bacterial strains, an inability to actively regulate the local wound microenvironment,^6^ and a lack of intrinsic pro-regenerative functionality.^7^ As a result, current approaches struggle to fulfill the therapeutic imperative of simultaneously achieving effective infection control, inflammation resolution, and tissue regeneration, an integrated paradigm increasingly recognized as essential for advanced wound care.

Photothermal therapy (PTT) represents a novel, non-antibiotic-dependent paradigm for physical antibacterial treatment.^8^ By converting light energy into heat (typically 50–60 °C) through photothermal materials, PTT denatures bacterial proteins and disrupts membrane structures, achieving rapid and broad-spectrum bactericidal effects.^9^ Importantly, this mechanism is less likely to induce resistance compared to traditional antibiotic therapies.^10^ In terms of light source selection, near-infrared II (NIR-II, 1000–1350 nm), particularly at 1064 nm, offers significant advantages over the conventional NIR-I window (700–950 nm).^11^ Due to lower absorption coefficients of water molecules and hemoglobin in this wavelength range, along with reduced light scattering, the 1064 nm laser achieves penetration depths of 5–10 mm, approximately two to three times greater than those achieved with 808 nm lasers.^12^ This enhanced penetration enables more focused energy deposition and minimizes thermal damage to healthy tissues, thereby providing precise intervention for deep-seated infections.^13^

At the material level, copper sulfides such as Cu_9_S_8_ nanoparticles exhibit remarkable potential due to their strong localized surface plasmon resonance (LSPR) absorption at 1064 nm, high photothermal conversion efficiency (reported between 40% and 50%), excellent photothermal stability, and intrinsic auxiliary antibacterial and pro-angiogenic properties of copper ions.^14^ These features make Cu_9_S_8_ an ideal candidate for PTT applications. Equally important is the biofunctional design of the dressing matrix. β-Glucan, approved by the FDA as a natural immunomodulatory polysaccharide, not only maintains a moist wound microenvironment through its high water-holding capacity but also specifically binds to macrophage Dectin-1 receptors.^15,16^ This interaction drives the polarization of M1 macrophages toward the M2 phenotype, inhibits the NF-κB pathway, downregulates TNF-α and IL-6, upregulates IL-10 and TGF-β, and promotes VEGF secretion and fibroblast migration, all of which create favorable conditions for tissue regeneration.^17^ Tannic acid, a natural polyphenol, contributes to the construction of smart responsive networks through its dynamic crosslinking, antioxidant, and auxiliary antibacterial properties.^18^ Together, these components synergize to form a multifunctional hydrogel system that addresses both infection control and tissue repair.

Hydrogels are widely regarded as promising wound dressings owing to their high water content (>90%), excellent biocompatibility, and three-dimensional network structure that closely resembles the native extracellular matrix (ECM).^19^ These features support key aspects of wound healing: the moist microenvironment enhances cell migration and re-epithelialization, while injectability and self-healing properties enable minimally invasive application and conformal adaptation to irregular wound geometries.^20^ Current strategies for functional hydrogel design generally fall into three categories. The first involves antibiotic loading, for example, vancomycin-loaded chitosan/gelatin hydrogels.^21^ However, sustained or uncontrolled release of antibiotics may promote bacterial resistance. A second approach employs photothermal agents, such as polydopamine-modified hydrogels, but these systems are often restricted by the limited tissue penetration of NIR-I light (700–900 nm) and tend to suffer from performance loss due to nanoparticle aggregation during irradiation.^22^ A third line of research incorporates immunomodulatory factors, such as interleukin-4-loaded alginate hydrogels, yet these designs frequently lack integration between antibacterial and regenerative functions and rely heavily on covalent crosslinking, which can impair dynamic responsiveness and hinder controlled biodegradation.^23^ A critical limitation across many existing platforms is the decoupling of rapid bactericidal action from long-term tissue repair.^24^ Few systems are capable of simultaneously achieving efficient bacterial clearance, modulating the inflammatory microenvironment, and actively supporting tissue regeneration in a coordinated manner.^25^ This gap underscores the need for a multifunctional hydrogel that combines deep-penetrating NIR-II photothermal responsiveness, a dynamically reversible physical network, and intrinsic bioactivity to achieve spatiotemporally coordinated infection control and tissue restoration.

**Scheme 1:**
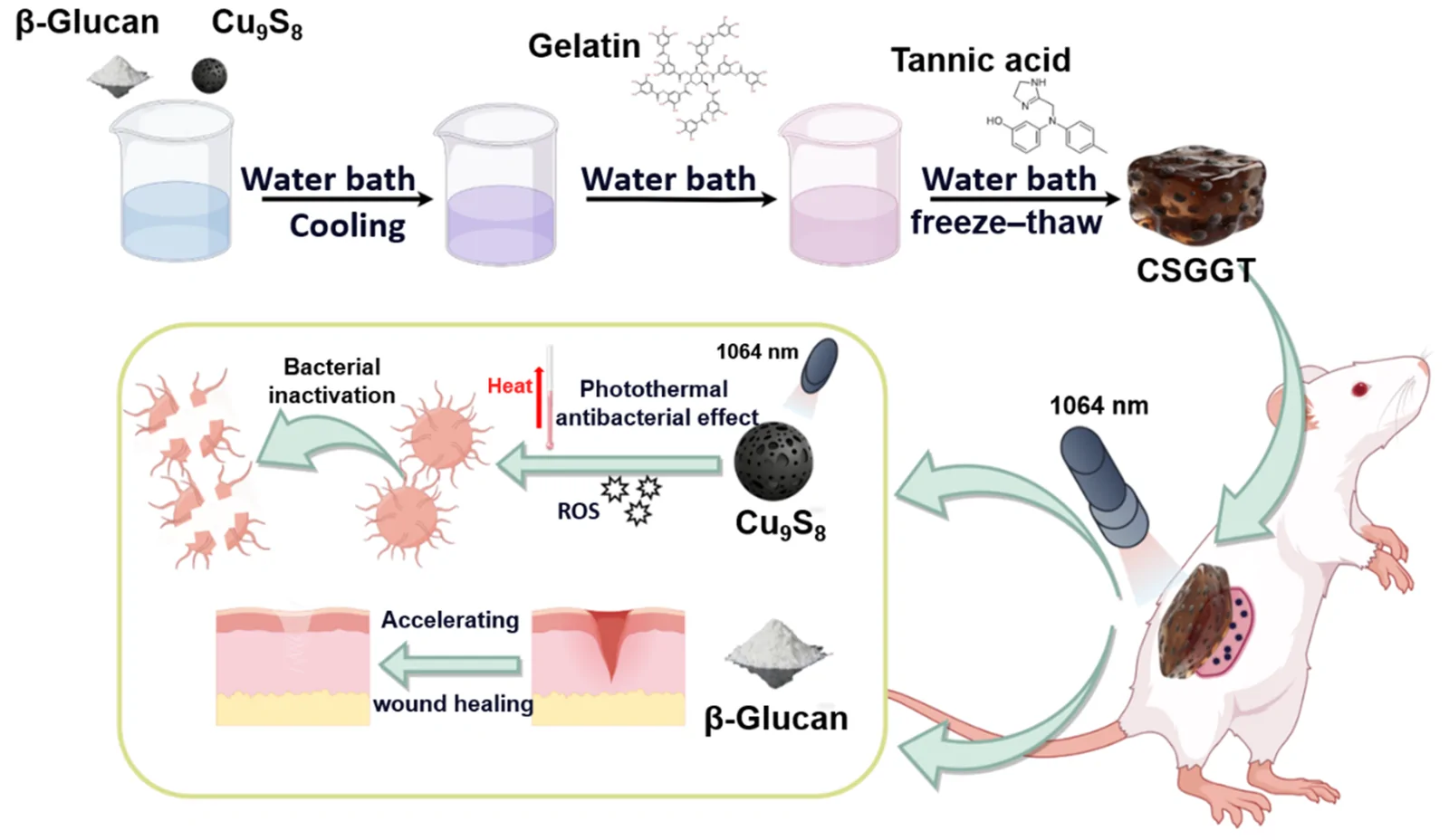
Schematic illustration of the fabrication process of the β-glucan–gelatin–tannic acid–Cu_9_S_8_ composite hydrogel (CSGGT) and its synergistic antibacterial and wound healing mechanism.

Guided by the principle of “antibacterial-repair synergy,” we developed a β-glucan–gelatin–tannic acid–Cu_9_S_8_ composite hydrogel (Scheme 1). In this system, β-glucan and gelatin jointly mimic the ECM and provide RGD-like motifs that support cell adhesion and proliferation. Cu_9_S_8_ nanoparticles are homogeneously dispersed throughout the matrix and efficiently convert 1064 nm NIR-II light into heat, enabling rapid eradication of bacteria. Importantly, the photothermal stimulus synergizes with β-glucan for bacterial elimination and inflammation alleviation, thereby realizing a sequential therapeutic sequence comprising immediate bacterial elimination, subsequent inflammation alleviation, and active tissue regeneration.

## 2. Experimental section

Detailed experimental details, including complete material synthesis procedures, instrument models and parameters, the ethical approval number for animal experiments, and statistical analysis methods, are provided in the SI.

## 3. Results

### 3.1 Synthesis and characterization

**Fig. 1:**
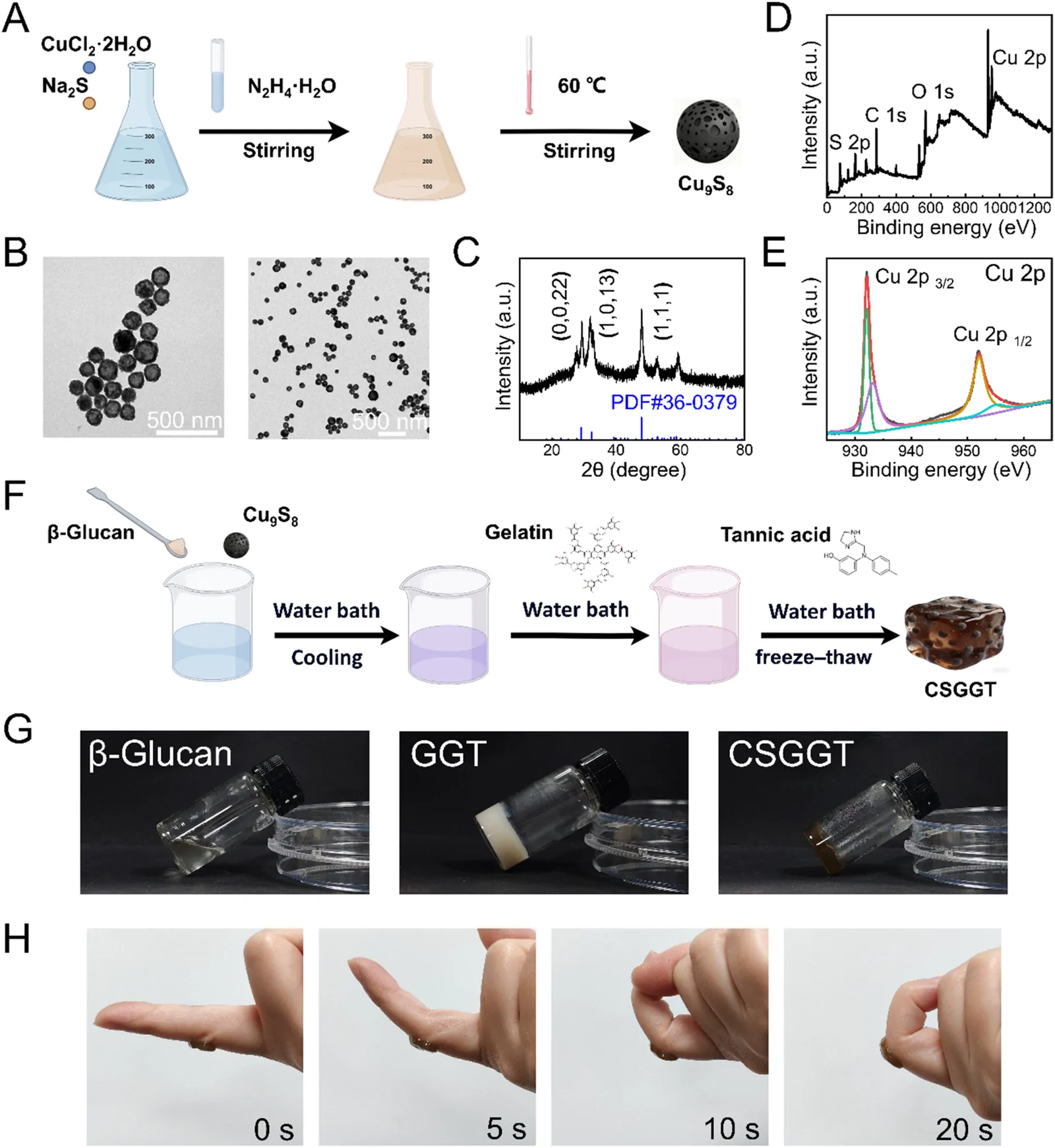
Synthesis and characterization of Cu_9_S_8_ NPs and fabrication, morphology, and adhesive performance of the CSGGT composite hydrogel. (A) Schematic illustration of the fabrication process for Cu_9_S_8_. (B) Representative TEM images of Cu_9_S_8_ NPs at different magnifications (scale bar: 500 nm). (C) XRD pattern of Cu_9_S_8_ NPs. (D) XPS survey spectrum of Cu_9_S_8_ NPs. (E) High-resolution XPS spectrum of Cu 2p for Cu_9_S_8_ NPs. (F) Schematic illustration of the fabrication process for the CSGGT hydrogel. (G) Photographs of β-glucan solution, GGT hydrogel, and CSGGT hydrogel. (H) Photographs showing the adhesive performance of CSGGT hydrogel on a finger at different time points (0, 5, 10, and 20 s).

Cu_9_S_8_ nanoparticles (NPs) were synthesized *via* a hydrothermal route using Cu_2_O as the precursor, following a previously reported protocol^26^ (Fig. 1A). Transmission electron microscopy (TEM) revealed that the resulting NPs were uniformly sized, with an average diameter of ∼150 nm, and exhibited a well-defined hollow morphology (Fig. 1B). The crystal structure was confirmed by X-ray diffraction (XRD). As shown in Fig. 1C, the pattern displays three sharp peaks at 29.3°, 32.1°, and 47.9°, which can be readily indexed to the (0 0 22), (1 0 13), and (1 1 1) reflections of hexagonal Cu_9_S_8_, respectively, indicating successful formation of phase-pure Cu_9_S_8_. To probe the surface chemistry and oxidation states, X-ray photoelectron spectroscopy (XPS) was performed. The survey scan confirmed the presence of Cu, S, O, and adventitious C (Fig. 1D). In the high-resolution Cu 2p spectrum (Fig. 1E), the main peaks at 932.1 eV (Cu 2p_3/2_) and 951.9 eV (Cu 2p_1/2_),^27^ along with the absence of strong satellite features, suggest that copper exists predominantly as Cu^+^, with a minor contribution from Cu^2+^. Meanwhile, the S 2p region (Fig. S1) shows a doublet at 162.1 and 163.1 eV, corresponding to S^2−^ in the sulfide lattice, and a higher-binding-energy shoulder at 168.8 eV, attributable to surface-oxidized sulfur species (*e.g.*, SO_*x*_^2−^), a common feature in metal sulfides exposed to ambient conditions.

Subsequently, a β-glucan–gelatin–tannic acid–Cu_9_S_8_ composite hydrogel (denoted CSGGT) was fabricated by incorporating Cu_9_S_8_ photothermal nanoparticles into a matrix composed primarily of β-glucan, with gelatin and tannic acid (TA) added at a mass ratio of 10 : 8 : 1 (Fig. 1F). Fig. S2 illustrates the functional mechanisms of individual components of CSGGT and their synergistic interactions. The hydrogel was formed through four sequential steps: thermal-induced swelling, integration of gelatin chains, polyphenol-mediated crosslinking reinforcement, and freeze–thaw rearrangement. A control hydrogel lacking Cu_9_S_8_ (denoted GGT) was prepared under identical conditions for direct comparison. During gelation, the abundant amino, carbonyl, and hydroxyl groups on gelatin side chains served as key sites for hydrogen bonding and other weak non-covalent interactions. β-Glucan and gelatin first associated reversibly *via* hydrogen bonds and van der Waals forces, forming physical domains that markedly enhanced the system's viscoelasticity. The subsequent addition of TA introduced further crosslinking points: its multiple phenolic hydroxyl groups engaged in multipoint interactions with hydrophilic moieties on both β-glucan and gelatin, thereby increasing network density and improving energy dissipation capacity. As shown in Fig. 1G, this hierarchy of physical interactions transformed the initially loose, semi-fluid β-glucan mixture into a cohesive, self-supporting three-dimensional network that is characteristic of multivalent physical hydrogels. Both GGT and CSGGT readily formed stable hydrogels, confirming effective water retention within the polymer matrix. Notably, the CSGGT patch adhered tightly to skin across a wide range of bending angles (Fig. 1H), demonstrating reliable conformal contact even under dynamic deformation. This combination of robust adhesion and flexibility not only shields the wound from external contaminants and pathogens but also helps maintain a moderately moist microenvironment, which is widely recognized as conducive to healing.^28^

**Fig. 2:**
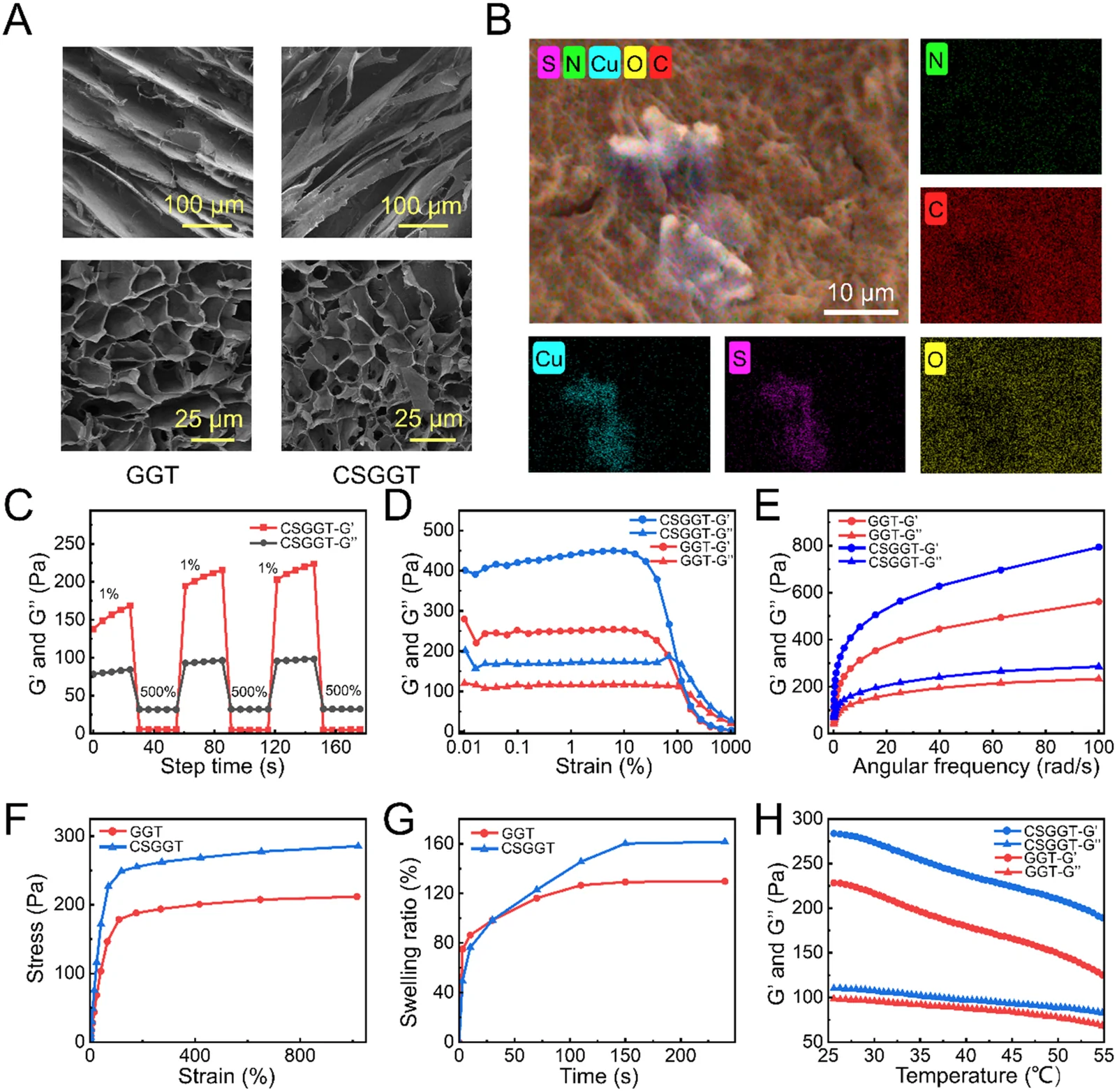
Morphological, compositional, and mechanical characterization of GGT and CSGGT hydrogels. (A) SEM images of GGT and CSGGT hydrogels (scale bars: 100 µm and 25 µm). (B) EDS elemental mapping of CSGGT. (C) Thixotropic recovery behavior of GGT and CSGGT hydrogels under alternating 1% and 500% strain steps. (D) Strain-dependent storage modulus (*G*′) and loss modulus (*G*″) of GGT and CSGGT hydrogels. (E) Frequency-dependent *G*′ and *G*″ of GGT and CSGGT hydrogels. (F) Stress–strain curves of GGT and CSGGT hydrogels. (G) Swelling ratio of GGT and CSGGT hydrogels over time. (H) Temperature-dependent *G*′ and *G*″ of GGT and CSGGT hydrogels.

To characterize the microstructure and macroscopic performance of CSGGT, a series of complementary techniques was employed. Scanning electron microscopy (SEM) revealed that CSGGT possessed a dense, continuous 3D porous network with thick pore walls and well-interconnected channels (Fig. 2A). Compared with the Cu_9_S_8_-free GGT hydrogel, incorporation of Cu_9_S_8_ nanoparticles as nanofillers significantly enhances the structural stability of the hydrogel network. This reinforcement effect may be attributed to surface hydrogen bonding and adsorption interactions between the nanoparticles and β-glucan/gelatin chains, as well as altered chain aggregation and rearrangement during the freeze–thaw process. Energy-dispersive X-ray spectroscopy (EDS) mapping (Fig. 2B) confirmed the presence of C, N, O, Cu, and S throughout the CSGGT hydrogel. Importantly, the Cu and S signals were uniformly distributed across the network, verifying homogeneous dispersion of Cu_9_S_8_ NPs. This uniform embedding ensures stable anchoring of the photothermal agent and provides a structural basis for efficient and consistent photothermal therapy. As shown in Fig. S3, the pore size distribution of CSGGT mainly ranged from 1 to 4 µm, with a peak around 2 µm, following an approximately normal distribution. Such a uniform microporous architecture supports efficient mass transport of nutrients, wound exudate, and therapeutic agents, creating a favorable microenvironment for photothermal antibacterial activity and tissue regeneration. As a functional dressing in direct contact with wound tissue, the hydrogel must balance biocompatibility, moisture management, and mechanical robustness, key requirements for clinical translation. In dynamic physiological environments, it needs to withstand repeated deformation without premature failure or excessive rigidity that could impede healing. Favorable mechanical properties not only protect the wound from external stress but also support essential cellular processes such as migration, proliferation, and tissue remodeling. Thixotropy tests (Fig. 2C) showed that both GGT and CSGGT rapidly recovered their elastic-dominant state (*G*′ > *G*″) after alternating high (500%) and low (1%) strain cycles, indicating fast network reformation following shear-induced disruption, a hallmark of self-healing physical hydrogels. Over a broad strain range (0.01–100%, Fig. 2D) and angular frequency sweep (1–100 rad s^−1^, Fig. 2E), both hydrogels maintained *G*′ consistently above *G*″, confirming a stable network structure with a wide linear viscoelastic region. Viscosity measurements revealed pronounced shear-thinning behavior (Fig. 2F): viscosity decreased sharply with increasing shear rate and quickly rebounded upon shear removal. This injectable, shear-responsive characteristic is highly advantageous for minimally invasive delivery and conformal coverage of irregularly shaped wounds.

As illustrated in Fig. S4, CSGGT exhibited significantly higher shear stress than GGT under identical strain conditions, indicating that Cu_9_S_8_ incorporation substantially reinforced the mechanical stiffness of the network. Swelling tests (Fig. 2G) further showed that CSGGT underwent rapid and extensive hydration within seconds of PBS exposure, enabling swift uptake of wound exudate and localized enrichment of therapeutic components at the wound bed. This immediate hydration not only accelerates drug release but also establishes the moist conditions necessary for reactive oxygen species (ROS) generation in photodynamic processes, thereby promoting cell migration, granulation tissue formation, and wound closure. Given its intended use in photothermal therapy, the temperature-dependent rheological behavior of CSGGT was also evaluated. As shown in Fig. 2H, the hydrogel maintained *G*′ > *G*″ across a temperature range of 25–55 °C, confirming that the network remains elasticity-dominated under both physiological and photothermal treatment conditions—an essential prerequisite for *in situ* therapeutic efficacy. Overall, CSGGT outperformed GGT in storage modulus, shear viscosity, and stress–strain response, underscoring the multifunctional role of Cu_9_S_8_ as a physical reinforcing filler. Beyond contributing mechanical strength through weak interactions (*e.g.*, hydrogen bonding and surface adsorption) with β-glucan and gelatin chains, Cu_9_S_8_ also influenced chain aggregation and reorganization during freeze–thaw cycling, leading to a more uniform and densely packed microstructure. This synergistic effect translated into enhanced elastic modulus, shear resistance, and toughness. Fig. S5 demonstrates the sustained and slow copper ion release profile of CSGGT. Upon NIR laser irradiation, the cumulative release rates at 24 h and 48 h increase slightly due to thermal expansion of the hydrogel network, while the overall release behavior remains well-controlled. Collectively, these results demonstrate that CSGGT forms a dense, stable, and multifunctional 3D network through multiscale physical crosslinking. Its rapid swelling, injectability, mechanical robustness, and compatibility with photothermal/photodynamic strategies position it as a promising platform for advanced wound management. Moreover, the hierarchically crosslinked architecture not only ensures microstructural uniformity and stability but also endows the material with distinct self-healing capability and thermal adaptability, critical attributes for maintaining reliable performance in real-world therapeutic applications.

### 3.2 Photothermal performance characterization

**Fig. 3:**
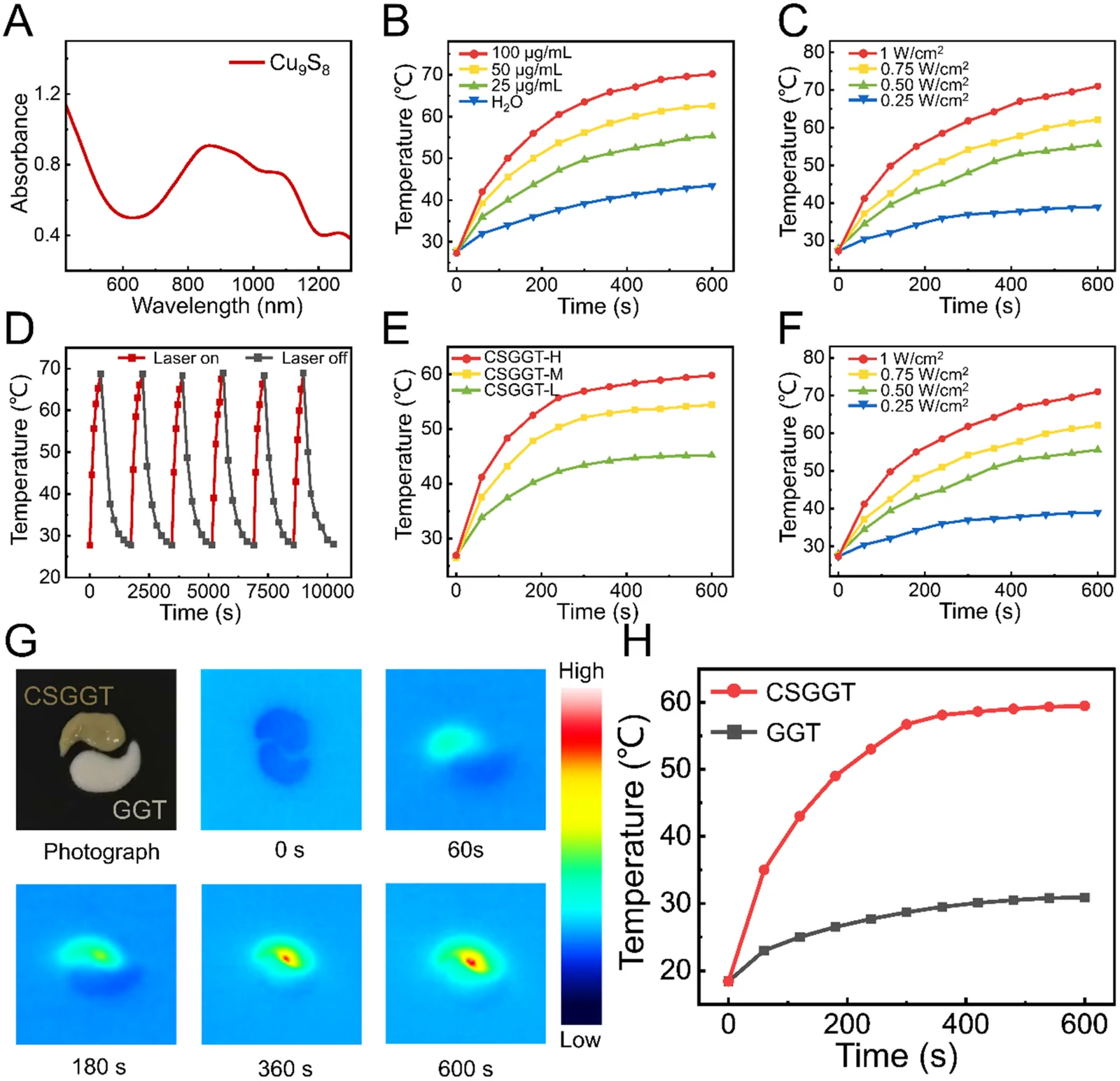
Photothermal performance of Cu_9_S_8_ nanoparticles and CSGGT composite hydrogel. (A) UV-vis-NIR absorption spectrum of Cu_9_S_8_ nanoparticles. (B) Temperature elevation curves of Cu_9_S_8_ aqueous solutions (Cu_9_S_8_ solutions at 25, 50, 100, 200 µg mL^−1^; 1064 nm laser at 1.0 W cm^−2^). (C) Temperature elevation curves of Cu_9_S_8_ solution (100 µg mL^−1^ Cu_9_S_8_ solution; 1064 nm laser at 0.25, 0.50, 0.75, 1.0 W cm^−2^). (D) Photothermal stability of Cu_9_S_8_ solution (100 µg mL^−1^ Cu_9_S_8_ solution; 1064 nm laser at 1.0 W cm^−2^; 6 on–off cycles). (E) Temperature elevation curves of CSGGT hydrogels with different Cu_9_S_8_ loadings (CSGGT-L (0.5 mg mL^−1^), CSGGT-M (1.0 mg mL^−1^), CSGGT-H (2.0 mg mL^−1^); 1064 nm laser at 1.0 W cm^−2^). (F) Temperature elevation curves of CSGGT-H hydrogel (1064 nm laser at 0.25, 0.50, 0.75, 1.0 W cm^−2^). (G) Photograph and thermal images of CSGGT and GGT hydrogels. (H) Temperature elevation curves of CSGGT and GGT hydrogels (1064 nm laser at 1.0 W cm^−2^).

To assess the photothermal antibacterial potential of the CSGGT hydrogel dressing, we first recorded the ultraviolet-visible-near-infrared (UV-vis-NIR) absorption spectrum of Cu_9_S_8_ NPs. As shown in Fig. 3A, a strong absorption band centered at 1064 nm was observed, confirming that Cu_9_S_8_ NPs are well suited for NIR-II photothermal therapy and possess high light-to-heat conversion capability. These characteristics make them promising candidates for treating bacteria-infected skin wounds. We next examined the concentration- and power-dependent photothermal response of Cu_9_S_8_ NPs in aqueous suspension. The temperature rise increased progressively with higher NP concentrations (Fig. 3B) and greater laser powers (Fig. 3C), demonstrating that the photothermal output can be precisely tuned by adjusting either parameter. Importantly, the system exhibited excellent reversibility. During six consecutive on–off laser cycles at 1064 nm, the heating–cooling profiles overlapped almost perfectly (Fig. 3D), which confirms the remarkable photothermal stability of Cu_9_S_8_ under repeated irradiation. To quantify this performance, we calculated the photothermal conversion efficiency (*η*) using a standard method based on time–temperature decay curves (Fig. S6). The resulting *η* value reached 43.87% (Fig. S7), reflecting the high efficiency of Cu_9_S_8_ in converting NIR light into heat. Encouraged by these results, we integrated Cu_9_S_8_ into the hydrogel matrix and prepared three formulations with low, medium, and high nanoparticle loadings, designated CSGGT-L, CSGGT-M, and CSGGT-H, respectively. Under identical 1064 nm laser irradiation, the equilibrium temperature of the hydrogels increased steadily with Cu_9_S_8_ content (Fig. 3E). This result confirms that the photothermal heating capacity of CSGGT can be rationally modulated through nanoparticle dosage. At a fixed Cu_9_S_8_ loading, the heating rate also scaled with laser power (Fig. 3F), further validating the responsive and controllable nature of the composite system. To visualize this effect in real time, we performed infrared (IR) thermal imaging on CSGGT and the Cu_9_S_8_-free control (GGT) placed side by side under 1064 nm irradiation. As shown in Fig. 3G, the CSGGT region heated rapidly and uniformly, while the GGT region remained essentially at ambient temperature throughout the exposure. This stark contrast was quantitatively confirmed by the temperature–time traces in Fig. 3H, which clearly demonstrate that the photothermal effect originates exclusively from Cu_9_S_8_ and is effectively retained within the hydrogel matrix.

### 3.3 *In vitro* antibacterial performance

**Fig. 4:**
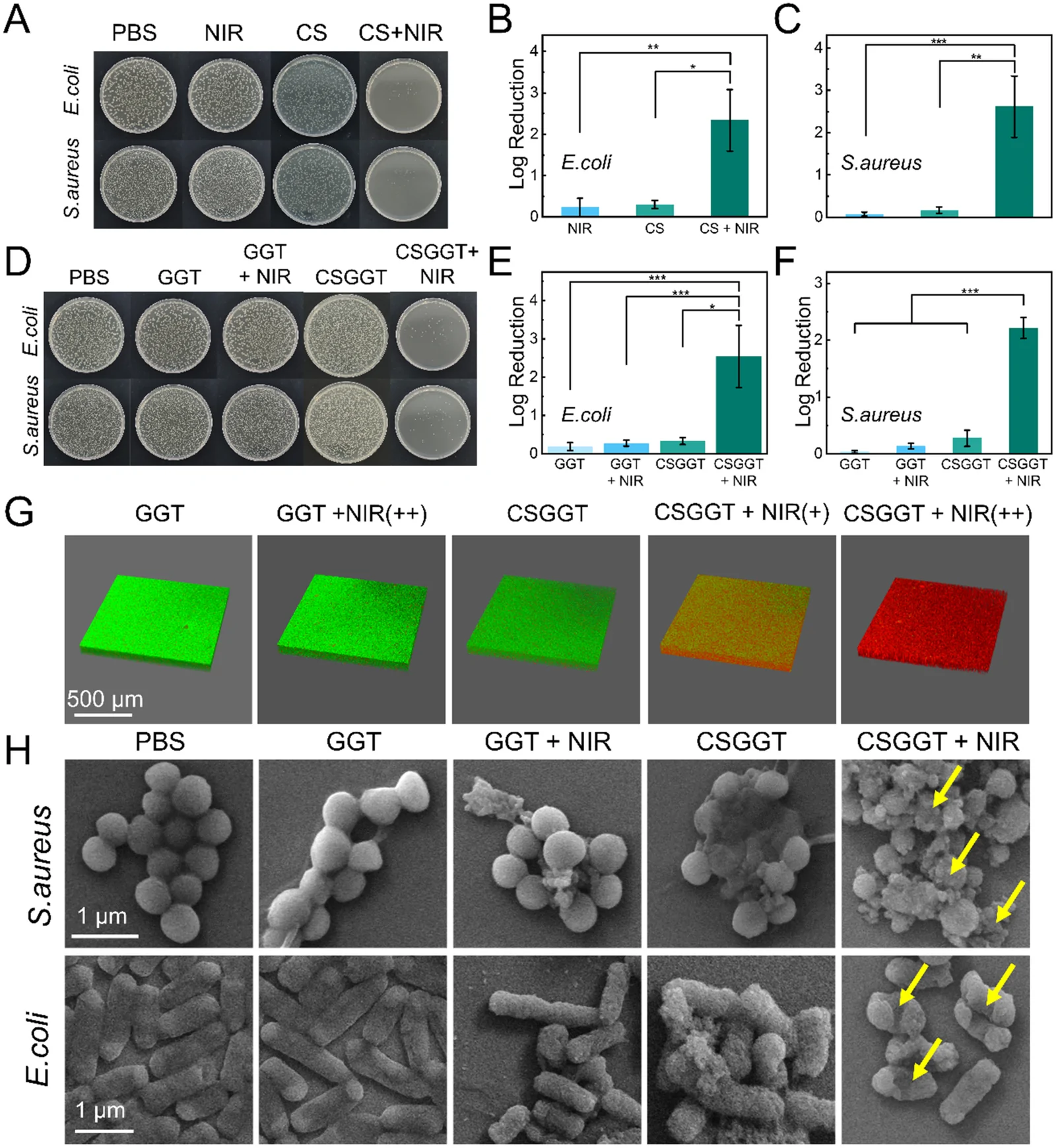
*In vitro* antibacterial performance of Cu_9_S_8_ NPs and CSGGT hydrogel against *E. coli* and *S. aureus*. (A) Photographs of bacterial colonies on agar plates after different treatment. (B and C) Corresponding log reduction of *E. coli* (B) and *S. aureus* (C) after treatment with CS under different conditions. (D) Photographs of bacterial colonies on agar plates after different treatment. (E and F) Corresponding log reduction of *E. coli* (E) and *S. aureus* (F) after treatment with hydrogels under different conditions. (G) 3D Live/Dead staining images of *S. aureus* after different treatment (scale bar: 500 µm). (H) SEM images of *S. aureus* and *E. coli* after different treatments (scale bar: 1 µm; yellow arrows indicate severe bacterial membrane damage) (***P < 0.001, **P < 0.01, *P < 0.05).

Encouraged by the strong photothermal capability of CSGGT, we next evaluated its antibacterial efficacy *in vitro*. Initial experiments employed the standard agar plate counting method to assess the antibacterial activity of Cu_9_S_8_ NPs against *Escherichia coli* (*E. coli*) and *Staphylococcus aureus* (*S. aureus*). As shown in Fig. 4A–C, neither laser irradiation alone nor Cu_9_S_8_ NPs without irradiation (CS group) exhibited significant bactericidal effects. In stark contrast, the CS + NIR group, leveraging the superior photothermal conversion of Cu_9_S_8_, achieved substantial bacterial reduction: 2.34-log for *E. coli* and 2.62-log for *S. aureus*. Moreover, the extent of bacterial killing could be precisely tuned by adjusting NIR irradiation intensity and duration, demonstrating controllable antibacterial activity. We then compared the antibacterial performance of GGT and CSGGT hydrogels. As illustrated in Fig. 4D–F, GGT, GGT + NIR, and CSGGT (without irradiation) showed negligible activity against both bacterial strains. However, upon NIR irradiation, the CSGGT + NIR group induced a dramatic enhancement in antibacterial efficacy, achieving 2.54-log reduction for *E. coli* and 2.21-log for *S. aureus*. These results confirm that incorporating Cu_9_S_8_ into the GGT matrix transforms it into an effective photothermal antibacterial dressing capable of eradicating multiple pathogenic bacteria under NIR-II irradiation. To further visualize the impact of photothermal treatment on bacterial viability, we performed dual-fluorescence staining with SYTO 9 (green, stains all bacteria) and propidium iodide (PI, red, penetrates only membrane-compromised cells), followed by confocal laser scanning microscopy (CLSM) imaging. As shown in Fig. S8, untreated control bacteria displayed uniform bright green fluorescence (SYTO 9^+^/PI^−^), indicating intact membranes and full viability. Similarly, samples treated with GGT, GGT + high-intensity light, or CSGGT alone showed fluorescence patterns indistinguishable from the control, consistent with the plate-count results and confirming minimal bactericidal effect under these conditions. In contrast, after CSGGT-mediated low-dose photothermal treatment, a subset of bacteria exhibited co-localized green and red signals (SYTO 9^+^/PI^−^), appearing as yellow-orange merged fluorescence—indicative of partial membrane damage that permits limited PI influx while retaining some membrane integrity. Under high-dose photothermal treatment, however, the majority of bacteria emitted only intense red fluorescence (SYTO 9^−^/PI^+^), with complete loss of green signal, signifying irreversible membrane rupture and cell death. These observations directly demonstrate that the CSGGT-based photothermal system can induce graded, laser-parameter-dependent damage to bacterial membranes, enabling precise and efficient bacterial eradication. To elucidate the underlying antibacterial mechanism, we examined morphological changes in *E. coli* and *S. aureus* after various treatments using scanning electron microscopy (SEM). As shown in Fig. 4H, untreated bacteria maintained smooth, intact surfaces. While GGT or CSGGT alone caused some bacterial aggregation, no obvious structural damage was observed, even in the GGT + NIR group, suggesting preserved cellular viability. Strikingly, in the CSGGT + NIR group (highlighted by yellow arrows), *E. coli* cells exhibited severe wrinkling and surface roughening, losing their characteristic rod-like morphology. *S. aureus* cells were even more severely affected, showing fragmentation and leakage of intracellular contents. These morphological disruptions provide direct visual evidence that the photothermal action of CSGGT compromises bacterial structural integrity, leading to irreversible inactivation.

### 3.4 Biocompatibility and wound healing promotion

**Fig. 5:**
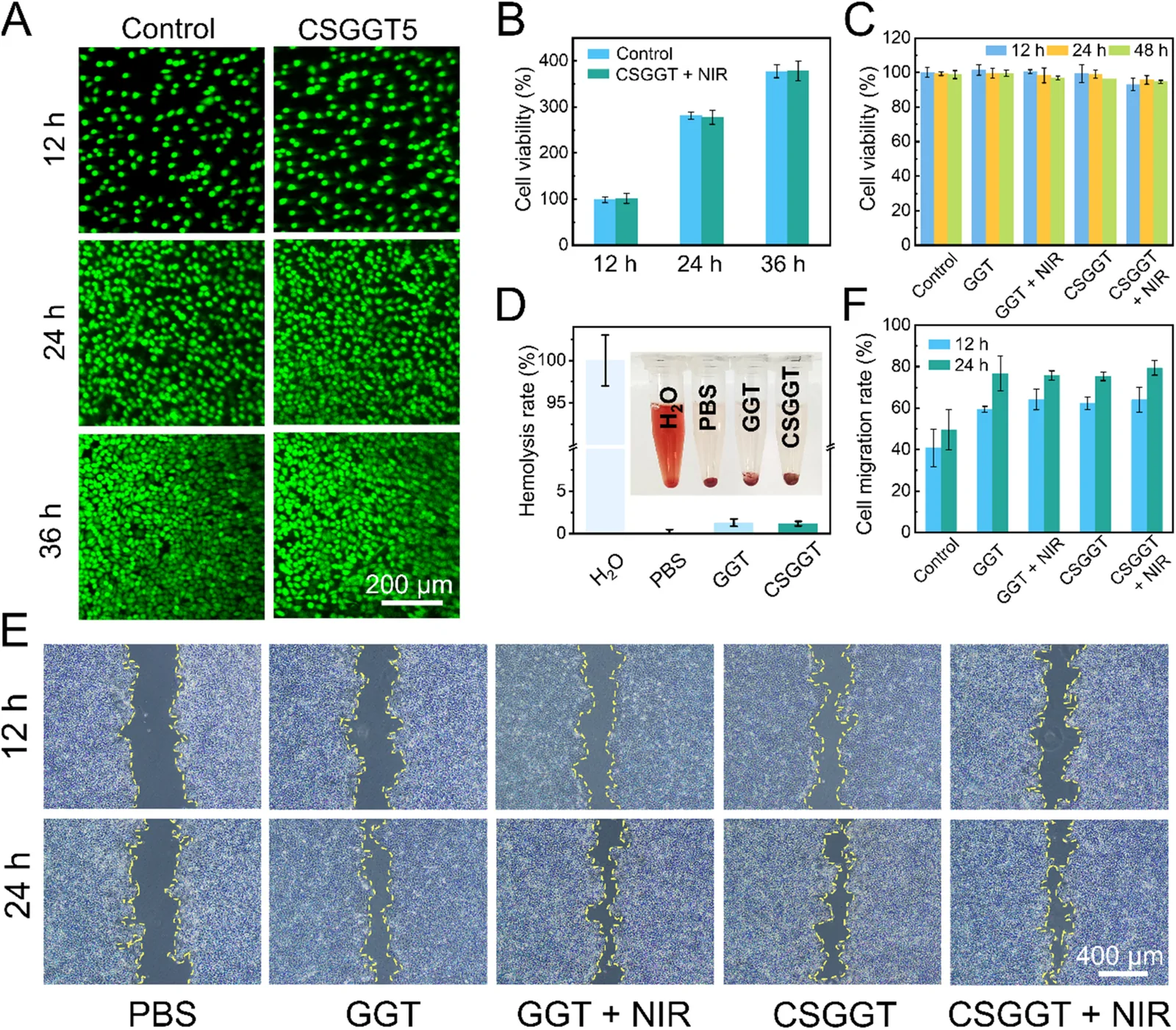
Biocompatibility and wound healing promotion of CSGGT hydrogel. (A) Live/Dead CLSM images of L929 fibroblasts cultured with CSGGT5 at 12, 24, and 36 h (scale bar: 200 µm). (B) Quantitative analysis of cell viability corresponding to (A). (C) CCK-8 assay of L929 fibroblasts after different treatment. (D) Hemolysis assay of CSGGT hydrogels, with corresponding quantitative hemolysis rate (inset: photographs of hemolysis). (E) Scratch assay images of L929 fibroblasts after different treatment (scale bar: 400 µm). (F) Quantitative analysis of cell migration rate corresponding to (E) (***P < 0.001, **P < 0.01, *P < 0.05).

The prerequisite for CSGGT hydrogel as a wound dressing is its excellent biocompatibility. We initially cultured L929 fibroblasts in the presence of CSGGT hydrogel solution and performed Live/Dead staining at 12 h, 24 h, and 36 h, followed by high-resolution confocal laser scanning microscopy (Fig. 5A). The results showed that L929 cells maintained comparable viability to the control group at different time points, with slightly higher cell activity observed at 36 h (Fig. 5B), suggesting a potential role in promoting cell proliferation. This finding was corroborated by CCK-8 assays (Fig. 5C), which demonstrated that even under NIR irradiation, L929 cell viability remained within an optimal range when exposed to CSGGT hydrogel. To further assess hemocompatibility, we incubated CSGGT hydrogel with murine red blood cells. As shown in Fig. 5D, the supernatant from the positive control exhibited a distinct red color, indicative of severe hemolysis due to erythrocyte rupture. In contrast, the supernatants from GGT and CSGGT solutions were optically clear, similar to the negative PBS control, confirming the excellent blood compatibility of the CSGGT hydrogel. Next, we evaluated the effect of CSGGT photothermal stimulation on cell migration using a scratch assay to simulate the chronic wound microenvironment. As illustrated in Fig. 5E, both GGT and CSGGT hydrogel groups exhibited enhanced cell migration compared to the control group, likely due to the proliferative effects of β-glucan. CSGGT + NIR maintained comparable cell migration capacity to the GGT and CSGGT groups, confirming that photothermal antibacterial treatment does not compromise the pro-healing bioactivity of the hydrogel matrix, enabling compatible antibacterial and repair functions. Notably, the CSGGT + NIR group demonstrated similar cell migration promotion as the other three groups (Fig. 5F). This indicates that the photothermal antibacterial process mediated by Cu_9_S_8_ NPs did not significantly interfere with the wound healing properties of the β-glucan-based hydrogel. Consequently, CSGGT can simultaneously exert effective bactericidal action and promote wound healing, achieving synergistic benefits through spatial and temporal control. These findings collectively demonstrate that CSGGT hydrogel not only possesses excellent biocompatibility but also promotes cellular proliferation and migration, making it a promising dual-functional material for wound management. Its ability to combine efficient photothermal therapy with enhanced tissue repair capabilities underscores its potential for clinical applications in treating infected wounds.

### 3.5 Transcriptomic profiling of immunomodulation and pro-healing mechanisms

To further decipher the molecular mechanisms underlying the cytoprotective and pro-regenerative effects observed *in vitro*, we performed transcriptome sequencing on HaCaT cells exposed to different conditioned microenvironments. The bacterial supernatant group was treated with sterile *S. aureus* culture supernatant to mimic the inflammatory milieu of infected skin wounds. The control group was incubated with bacterial supernatant collected after CSGGT + NIR photothermal treatment, together with CSGGT hydrogel extract, to recapitulate the integrated therapeutic microenvironment combining bacterial clearance and bioactive hydrogel intervention.

**Fig. 6:**
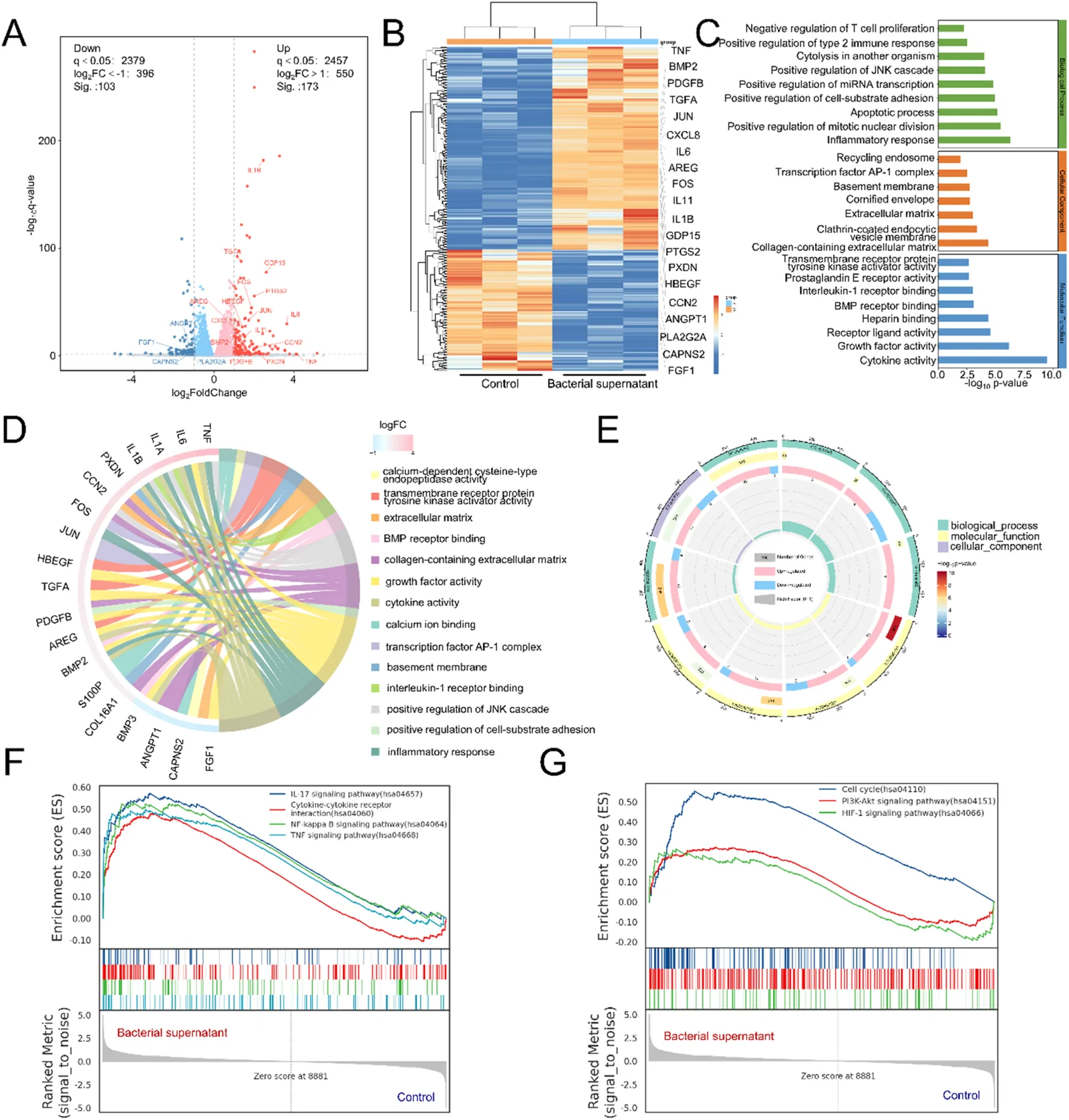
Transcriptomic analysis of HaCaT cells under different conditioned microenvironments. (A) Volcano plot of differentially expressed genes between the two groups, with representative functional genes labeled. (B) Hierarchical clustering heatmap of differentially expressed genes. (C) Bar plot of Gene Ontology (GO) enrichment analysis. (D) GO enrichment chord diagram. (E) GO enrichment circular plot. (F) Gene Set Enrichment Analysis (GSEA) results for pro-inflammatory pathways. (G) GSEA results for pro-healing pathways.

Differential gene expression analysis revealed distinct transcriptional profiles between the two groups. Compared with the bacterial supernatant group, the control group yielded 276 significantly differentially expressed genes, among which 173 were upregulated and 103 were downregulated (Fig. 6A). The volcano plot and hierarchical clustering heatmap demonstrated that the altered genes were involved in multiple functional categories closely related to wound healing, including inflammatory response, cell proliferation, extracellular matrix remodeling, and growth factor signaling. Pro-inflammatory genes such as IL1B, TNF, IL6, CXCL8, and PTGS2 were substantially downregulated in the control group, indicating that the therapeutic microenvironment effectively repressed the production of pro-inflammatory mediators in epidermal cells. Meanwhile, genes associated with tissue repair, angiogenesis, and matrix remodeling, including TGFB1, FGF1, PDGFB, ANGPT1, and GDF15, exhibited upregulated expression. Transcription factors FOS and JUN, which are implicated in stress response and tissue regeneration, also showed significant expression changes, reflecting broad modulation of cellular repair programs (Fig. 6B).

We next conducted Gene Ontology (GO) enrichment analysis to systematically annotate the functional implications of the differentially expressed genes. Significant enrichment was observed across all three GO domains: biological process, cellular component, and molecular function. In the biological process category, enriched terms included inflammatory response, apoptotic process, positive regulation of JNK cascade, positive regulation of cell–substrate adhesion, negative regulation of T cell proliferation, and positive regulation of mitotic nuclear division. These terms capture both the inflammatory and cellular stress states induced by bacterial infection, and the regulatory effects of treatment on cell proliferation, adhesion, and immune homeostasis. For cellular components, enrichment was concentrated in structures such as extracellular matrix, collagen-containing extracellular matrix, basement membrane, recycling endosome, and transcription factor AP-1 complex, consistent with the role of CSGGT in extracellular matrix remodeling and intracellular signal regulation. In the molecular function category, the differentially expressed genes were significantly enriched in growth factor activity, cytokine activity, transmembrane receptor protein tyrosine kinase activator activity, interleukin-1 receptor binding, and BMP receptor binding, highlighting the coordinated involvement of growth factor and immune signaling pathways in the therapeutic effects (Fig. 6C). The GO enrichment chord diagram and circle plot further visualized the associations between individual genes and functional terms, corroborating the reliability of the enrichment results (Fig. 6D and E).

To explore pathway-level alterations induced by the therapeutic microenvironment, we performed Gene Set Enrichment Analysis (GSEA) on the whole transcriptome dataset. Classic pro-inflammatory pathways, including the IL-17 signaling pathway, cytokine–cytokine receptor interaction, NF-κB signaling pathway, and TNF signaling pathway, were significantly enriched in the bacterial supernatant group, reflecting robust activation of inflammatory signaling cascades in epidermal cells upon bacterial stimulation. In the control group, these pro-inflammatory pathways showed markedly diminished enrichment, confirming that CSGGT + NIR treatment effectively attenuates bacteria-driven hyperinflammatory responses at the pathway level (Fig. 6F). In contrast, pathways associated with cell proliferation and tissue regeneration, namely cell cycle, PI3K–Akt signaling pathway, and HIF-1 signaling pathway, were enriched in the control group. The PI3K–Akt pathway is a central regulator of cell survival, proliferation, and migration. The HIF-1 pathway plays a critical role in angiogenesis and metabolic adaptation during wound repair. Activation of the cell cycle pathway directly corresponds to enhanced cell proliferative capacity. These results indicate that the CSGGT-based therapeutic microenvironment promotes epidermal regeneration by activating pro-healing signaling programs, which is consistent with the pro-migratory and pro-proliferative phenotypes observed in cellular assays (Fig. 6G).

Collectively, the transcriptomic data provide molecular evidence that the therapeutic microenvironment generated by CSGGT + NIR treatment suppresses pro-inflammatory pathways and activates pro-repair programs in epidermal cells. This overall effect may stem from a combination of reduced bacterial toxin burden, photothermal modification of bacterial components, and the direct bioactivity of the hydrogel. This finding corroborates the integrated therapeutic design of the system and supports the interpretation of *in vivo* wound healing outcomes.

### 3.6 *In vivo* antibacterial performance

**Fig. 7:**
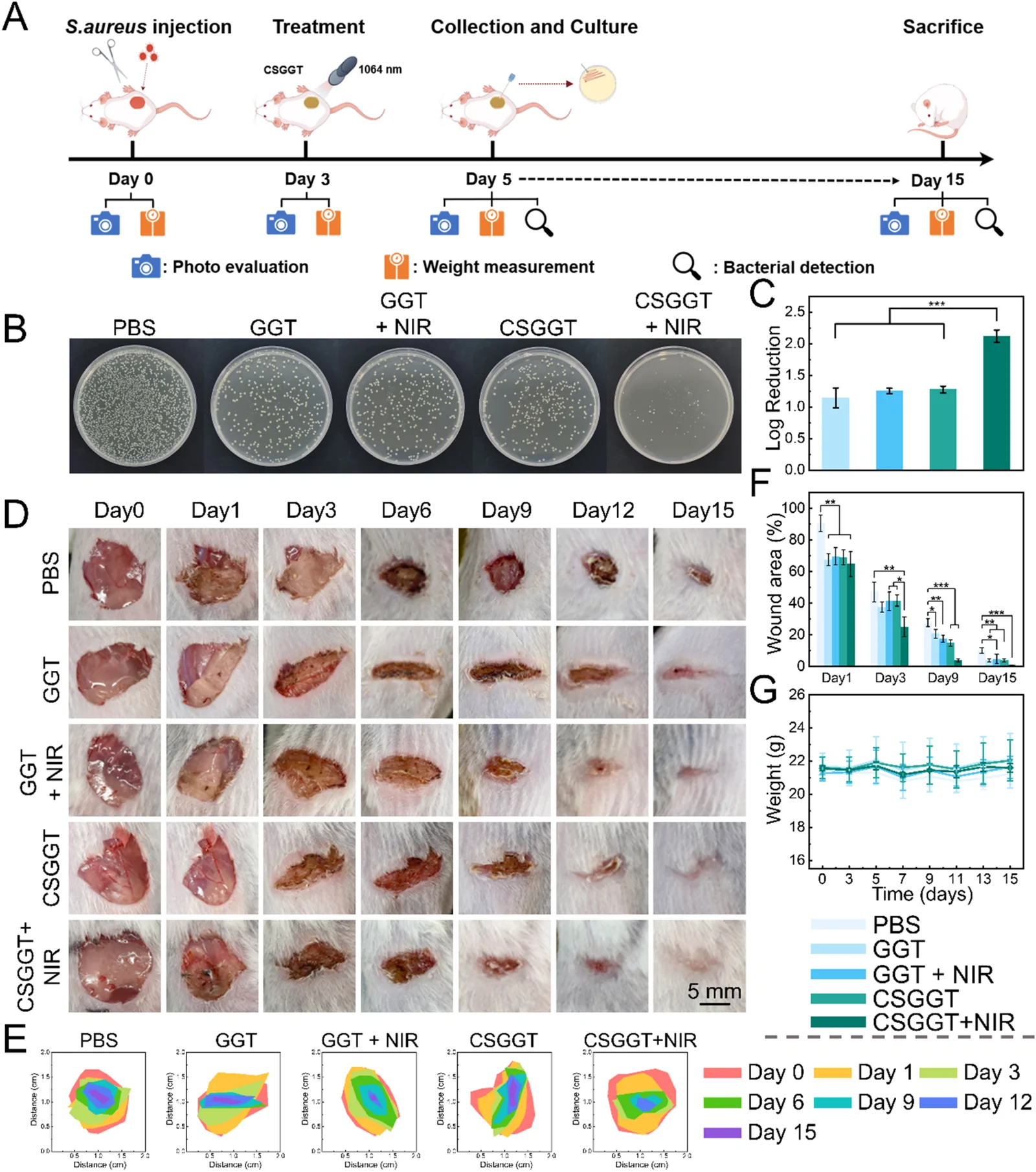
*In vivo* antibacterial efficacy and wound healing performance of CSGGT hydrogel in a *Staphylococcus aureus*-infected full-thickness skin wound model. (A) Schematic illustration of the *in vivo* therapeutic protocol. (B) Photographs of bacterial colonies on agar plates after different treatments for 3 days. (C) Corresponding log reduction of *S. aureus* after different treatments. (D) Representative digital photographs of infected wounds at different time points (day 0, 1, 3, 6, 9, 12, 15) after treatment with different hydrogels (scale bar: 5 mm). (E) Wound healing trajectory maps for each treatment group at day 1, 3, 6, 9, 12, and 15 post-treatment. (F) Quantitative analysis of wound area percentage at day 1, 3, 9, and 15 post-treatment. (G) Body weight changes of mice in different treatment groups over 15 days (***P < 0.001, **P < 0.01, *P < 0.05).

Encouraged by the *in vitro* results, we established a full-thickness skin wound model infected with *Staphylococcus aureus* in BALB/c mice to evaluate the *in vivo* antibacterial efficacy of the CSGGT hydrogel. The experimental protocol is illustrated in Fig. 7A. Briefly, a circular full-thickness wound approximately 10 mm in diameter was created on the dorsal skin of each mouse and inoculated with *S. aureus*. Wound areas were photographed daily, and body weight was monitored throughout the study. On day 3 post-infection, CSGGT hydrogel was applied to the wound site, followed by near-infrared (NIR) irradiation for photothermal therapy (PTT). Two days after treatment (day 5), infected skin tissues were harvested from each group, homogenized, serially diluted, and plated on agar for bacterial colony counting after 24 h of incubation. As shown in Fig. 7B and C, bacterial colonies remained detectable in the GGT, GGT + NIR, and CSGGT groups but were significantly reduced compared with the PBS control. This suggests that both GGT and CSGGT hydrogels can partially suppress bacterial proliferation even in the absence of PTT, likely by physically trapping bacteria within the hydrogel matrix and thereby interfering with their growth and colonization. In striking contrast, the CSGGT + NIR group exhibited a dramatic reduction in bacterial burden, consistent with its potent *in vitro* bactericidal activity. This result confirms that CSGGT enables effective *in vivo* photothermal antibacterial action when combined with NIR irradiation. We next assessed the impact of CSGGT on wound healing and tissue regeneration. As shown in Fig. 7D, the control group displayed the slowest healing progression, with a large open wound still present at day 12. Although the GGT, GGT + NIR, and CSGGT groups showed improved closure relative to the control, residual wounds or visible scarring remained evident at day 12. By contrast, the CSGGT + NIR group exhibited markedly accelerated re-epithelialization: wounds were nearly closed by day 9, and by day 12, the healed area was covered with newly grown hair, indicating functional skin recovery. Quantitative analysis of wound closure kinetics is presented in Fig. 7E and F. At day 9, the residual wound areas were 27.7 ± 2.5% (control), 20.8 ± 2.8% (GGT), 17.4 ± 2.2% (GGT + NIR), 15.1 ± 2.1% (CSGGT), and only 4.0 ± 1.0% (CSGGT + NIR)—the lowest among all groups. By day 12, these values further decreased to 10.2 ± 1.8% (control), 4.0 ± 1.0% (GGT), 4.7 ± 3.0% (GGT + NIR), 3.9 ± 1.0% (CSGGT), and 0.8 ± 0.2% (CSGGT + NIR). The superior healing outcome in the CSGGT + NIR group, accompanied by minimal scar formation, likely stems from the combined effects of rapid infection clearance and the intrinsic bioactivity of β-glucan. Consistent with the documented immunomodulatory effect of β-glucan *via* Dectin-1 receptor on macrophage polarization, accelerated healing in this study suggest that the hydrogel may alleviate inflammation and promote repair by regulating macrophage phenotype. Together, these findings demonstrate that CSGGT operates through a multimodal therapeutic mechanism: early-stage photothermal sterilization effectively eliminates bacterial infection, while the subsequent anti-inflammatory and pro-regenerative actions of the hydrogel matrix accelerate wound closure and skin reconstruction. Finally, as shown in Fig. 7G, no significant differences in body weight were observed across all treatment groups over the course of the experiment. This indicates that the hydrogel application and photothermal treatment did not induce systemic toxicity or adversely affect the general health of the animals.

**Fig. 8:**
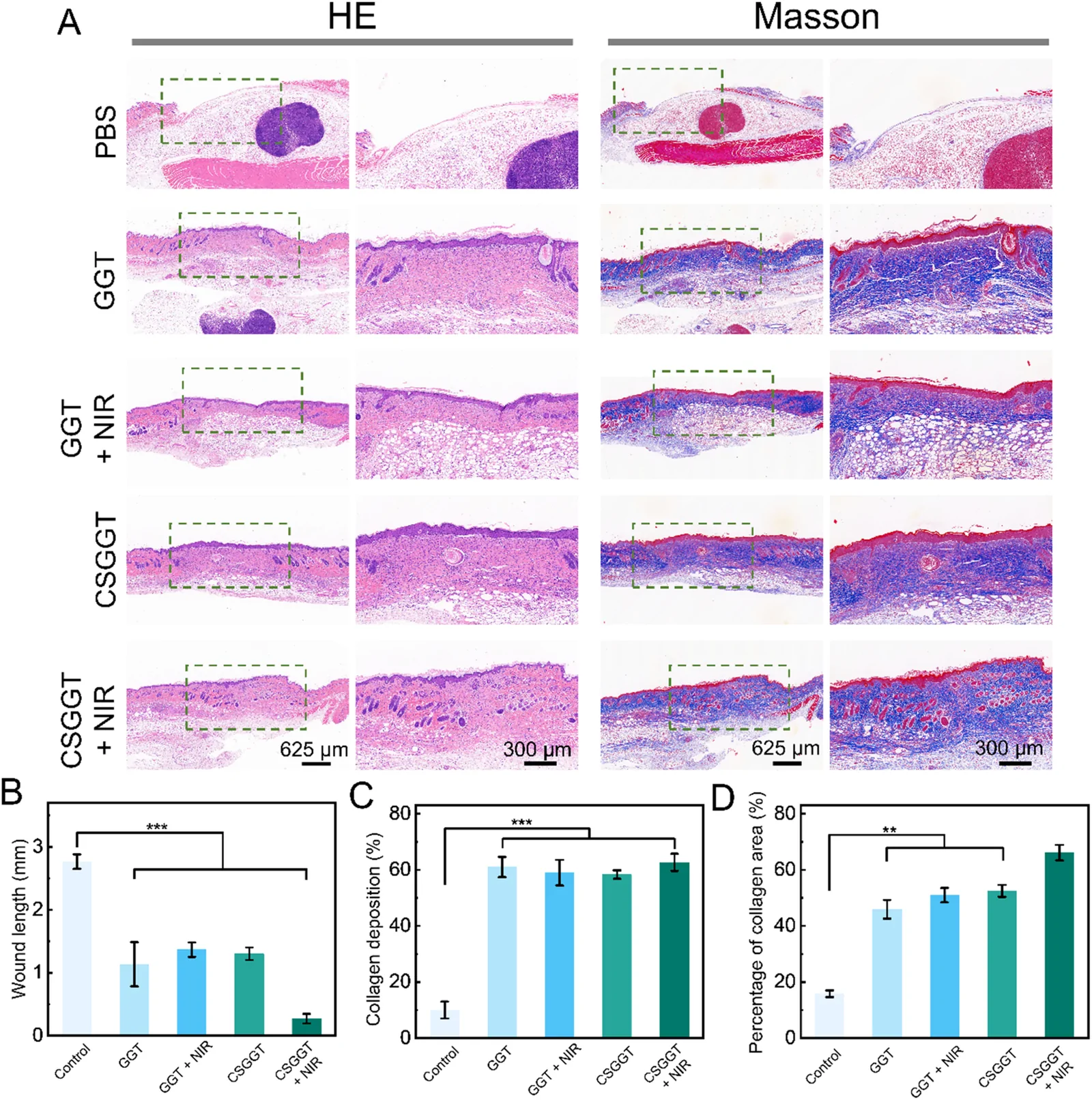
Histological evaluation of skin wound repair following different treatments. (A) Representative H&E and Masson's trichrome staining images of skin wound sections from different treatment groups (scale bars: 625 µm and 300 µm). (B) Quantitative analysis of wound length in different treatment groups. (C) Quantitative analysis of collagen deposition in different treatment groups (***P < 0.001, **P < 0.01, *P < 0.05). (D) Quantitative analysis of the collagen‑positive area ratio in the whole wound area.

Further histological evaluation was performed using hematoxylin and eosin (H&E) and Masson's trichrome staining to assess the therapeutic efficacy of CSGGT hydrogel in treating infected skin wounds at the tissue level (Fig. 8A). H&E staining revealed that the control group exhibited an unhealed wound with no evidence of re-epithelialization, incomplete granulation tissue formation, and persistent inflammatory cell infiltration. The dermal architecture remained severely disrupted, and no signs of hair follicle or sebaceous gland regeneration were observed, consistent with the macroscopic findings shown in Fig. 7D. In contrast, all other treatment groups demonstrated varying degrees of epithelial regeneration and tissue remodeling. Notably, the GGT and CSGGT groups showed partial re-epithelialization, with newly formed epidermis covering part of the wound bed. However, the most remarkable improvement was observed in the CSGGT + NIR group, where a continuous and well-differentiated stratified epidermis had fully regenerated, and multiple mature hair follicles and sebaceous glands were clearly visible within the healed area—indicating advanced restoration of normal skin appendages. Quantitative analysis of wound length (Fig. 8B) further confirmed these observations: while the control group showed only minimal wound contraction over time, the GGT, GGT + NIR, and CSGGT groups exhibited significantly reduced wound lengths—less than half that of the control. Most strikingly, the CSGGT + NIR group achieved a wound length reduction to approximately one-tenth of the control, aligning perfectly with the high wound closure rate observed in Fig. 7F. Collagen deposition is a critical indicator of dermal repair during wound healing. As the major structural component of the extracellular matrix, collagen provides mechanical support and serves as a scaffold for cellular migration and neovascularization. In Masson's staining, collagen fibers are stained blue, allowing for visual and quantitative assessment of fibrous tissue regeneration. As shown in Fig. 8C and D, the control group displayed markedly sparse collagen deposition, reflecting poor connective tissue remodeling. In contrast, all hydrogel-treated groups exhibited significantly enhanced collagen accumulation. Specifically, both GGT and CSGGT groups showed comparable levels of collagen deposition, indicating that β-glucan-based hydrogels effectively promote extracellular matrix synthesis and dermal reconstruction. Importantly, the CSGGT + NIR group demonstrated the highest degree of collagen organization and density, with tightly packed, parallel-aligned collagen fibers extending across the wound site, suggesting not only increased deposition but also improved structural integrity and maturity of the regenerated dermis. These histological results collectively demonstrate that CSGGT hydrogel, particularly when combined with NIR irradiation, not only accelerates wound closure and re-epithelialization but also promotes robust collagen synthesis and dermal regeneration. The synergistic effect of photothermal antibacterial action and bioactive hydrogel components enables a comprehensive therapeutic strategy that supports the full spectrum of wound healing processes, from infection control to tissue remodeling and functional recovery.

## 4. Conclusions

The CSGGT hydrogel integrates NIR-II photothermal antibacterial action with a bioactive matrix to accelerate infected wound healing. Its dynamic physical network ensures practical functionality (self-healing, injectability), while Cu_9_S_8_ enables rapid, localized bacterial clearance. *In vivo* results demonstrate significantly accelerated healing with restored skin architecture and reduced histological inflammation, directly evidenced by wound metrics and histopathology. By temporally coordinating early-stage bacterial elimination and sustained tissue regeneration, CSGGT addresses a critical clinical need without antibiotics. This study provides an experimentally validated foundation for next-generation wound dressings.

## Conflicts of interest

The authors declare no conflict of interest.

## Supplementary Material

RA-OLF-D6RA06536H-s001

## Data Availability

The data will be made available on request. Supplementary information (SI): detailed supplementary experimental protocols, supplementary physicochemical characterization data of the CSGGT composite hydrogel, the functional mechanisms of individual components of CSGGT,3D live/dead staining images of *S. aureus* and the cumulative copper ion release profile. All supplementary figures and tables are sequentially numbered and fully annotated. See DOI: https://doi.org/10.1039/d6ra06536h.
