# Supplementary material for: NIR-II photothermal CSGGT hydrogel for antibacterial therapy and accelerated healing of infected wounds
Source: RSC Adv. 2026 Sep 1;16(42):51545–59. doi: 10.1039/d6ra06536h (PMC13531522; doi:10.1039/d6ra06536h)
Supplement: Supplementary file 1 [file RA-016-D6RA06536H-s001.pdf]

Supplementary Information (SI) for RSC Advances.
This journal is © The Royal Society of Chemistry 2026

Supporting Information

NIR-II Photothermal CSGGT Hydrogel for Antibacterial Therapy and Accelerated Healing of Infected Wounds

Yani Zou[1]† Yingao Jiao[2]† Jiehao Zhou[3]† Helin Xu[3] Qiqi Liu[3] Dengyao Gao[2] Yan Gao[2] Yihong Yang[3] Yanlei Liu[2]* Hongbo Liu[3]**

1 Department of Radiation Oncology, Fuyang People's Hospital Affiliated to Anhui Medical University, 501 Sanqing Road, Yingzhou District, Fuyang, Anhui 236000, China; 2 Institute of Intelligent Health Diagnosis and Treatment, School of Automation and Intelligent Sensing, Shanghai Jiao Tong University, 800 Dongchuan Road, Shanghai, 200240, People's Republic of China; 3 Department of Emergency Medicine, Fuyang People's Hospital Affiliated to Anhui Medical University, 501 Sanqing Road, Yingzhou District, Fuyang, Anhui 236000, China.

†These authors contributed equally to this work

Correspondence: Yihong Yang, Department of Emergency Medicine, Fuyang People's Hospital Affiliated to Anhui Medical University, 501 Sanqing Road, Yingzhou District, Fuyang, Anhui 236000, China, Email 15856806657@163.com

Yanlei Liu Institute of Intelligent Health Diagnosis and Treatment, School of

Automation and Intelligent Sensing, Shanghai Jiao Tong University, 800 Dongchuan Road, Shanghai, 200240, People's Republic of China, Email liuyanlei@sjtu.edu.cn; Hongbo Liu, Department of Emergency Medicine, Fuyang People's Hospital Affiliated to Anhui Medical University, 501 Sanqing Road, Yingzhou District, Fuyang, Anhui 236000, China, Email liuhongbo@bbmu.edu.cn

Experimental Section

Materials

All chemicals were of analytical grade and used as received. $CuCl_2$, Na_2S, $N_2H_4 \cdot H_2O$, and ethanol were purchased from Aladdin Biochemical Technology Co., Ltd. β-Glucan was supplied by Sichuan Hetai Xinguang Biotechnology Co., Ltd. Gelatin was obtained from Shanghai Titan Scientific Co., Ltd., and tannic acid was provided by Shanghai Macklin Biochemical Co., Ltd. Ultrapure water (18.2 MΩ·cm at 25 °C) used throughout the experiments was produced by a Merck Milli-Q purification system. The SYTO 9/propidium iodide (PI) staining kit was provided by Beyotime Biotechnology.

Measurements and characterizations

The morphology of the materials was characterized using transmission electron microscopy (TEM; Talos F200X G2). UV-Vis-NIR absorption spectra were recorded on a Lambda 950 spectrophotometer. Crystal structures of Cu_9S_8 and CCC were analyzed by X-ray diffraction (D8 ADVANCE Da Vinci). Elemental composition and chemical states were determined by X-ray photoelectron spectroscopy (XPS; AXIS

Ultra DLD). Real-time temperature changes were monitored using an infrared thermal imaging camera (Testo 875-1). Fluorescence images were captured using a high-resolution laser scanning confocal microscope (Leica STELLARIS 5). Scanning electron microscopy (SEM) and energy-dispersive X-ray spectroscopy (EDS) were performed using a Carl Zeiss Sigma 500 field-emission scanning electron microscope (Germany), equipped for in-situ materials characterization. The instrument achieves a secondary electron (SE) resolution of 0.8 nm at 15 kV and 1.4 nm at 1 kV (with in-lens deceleration). The EDS system has an energy resolution better than 127 eV at the Mn-Kα line and is capable of elemental analysis across the range from beryllium (Be, Z = 4) to californium (Cf, Z = 98). Rheological measurements were carried out on a rotational rheometer (TA Instruments, USA), equipped with a Peltier plate temperature control system, an environmental test chamber (ETC), and a liquid nitrogen cooling accessory. A sandblasted upper parallel plate geometry with a diameter of 20 mm was used for all tests.

Synthesis of Cu_9S_8

Cu_9S_8 nanoparticles were synthesized according to a previously reported method. Briefly, 500 mg of polyvinylpyrrolidone (PVP-K30) and 40 mg of copper (II) chloride dihydrate were dissolved in 100 mL of deionized water under stirring for 30 min. Subsequently, 100 mL of NaOH solution (pH = 8) was added, and the mixture was stirred for an additional 15 min. Then, 5 μL of hydrazine hydrate was introduced dropwise, followed by another 10 min of stirring. Next, 2 mL of Na_2S solution (120 mg/mL) was added, and the reaction mixture was heated at 60 °C with continuous

stirring for 4 h. The resulting product was collected by centrifugation at 8000 rpm for 10 min, washed three times with deionized water.

Synthesis of CSGGT

A CSGGT composite hydrogel was fabricated by mixing β-glucan (60 mg/mL), gelatin (48 mg/mL), and tannic acid (6 mg/mL) at a weight ratio of 10:8:1, followed by incorporation of Cu_9S_8 nanoparticles as a photothermal agent.(CSGGT-L: 0.5 mg/mL Cu9S8 (0.05 wt% in the final hydrogel); CSGGT-M: 1.0 mg/mL Cu9S8 (0.1 wt%); CSGGT-H: 2.0 mg/mL Cu9S8 (0.2 wt%)) The preparation involved four sequential steps: (1) swelling of β-glucan at 90 °C to form an open-chain network; (2) cooling to 60 °C and introducing gelatin to establish a primary elastic network via hydrogen bonding and van der Waals interactions; (3) adding tannic acid to enable multiscale physical crosslinking through phenolic–hydroxyl hydrogen bonds and π–π stacking; and (4) subjecting the hydrogel to three freeze–thaw cycles (−80 °C/4 °C) to induce polymer chain rearrangement and long-range physical crosslinks.

In Vitro Photothermal Performance Evaluation

A series of in vitro experiments were conducted to assess the photothermal properties of Cu_9S_8 nanoparticles. An aqueous dispersion of Cu_9S_8 nanoparticles (0.1 mg/mL) was irradiated with a 1064 nm laser at power densities of 0.25, 0.5, 0.75, and 1.0 W/cm^2 for 600 seconds, and the temperature change was recorded every 10 seconds using an infrared thermal camera. Photothermal stability was evaluated by subjecting the same 0.1 mg/mL Cu_9S_8 solution to six consecutive cycles of laser irradiation (600 s) followed by cooling to room temperature, with temperature monitored throughout. The

photothermal conversion efficiency (η) was calculated according to a standard method using the following parameters: heat transfer coefficient (h), surface area (S), maximum steady-state temperatures of the Cu_9S_8 nanoparticle solution (T_max) and surrounding water (T_surr), incident laser power (I), and absorbance of Cu_9S_8 at 1064 nm. Additionally, CSGGT and GGT hydrogels were uniformly spread onto glass dishes, irradiated with a 1064 nm laser, and their temperature evolutions were recorded separately using infrared thermal imaging.

Cell Culture

L929 murine fibroblast cell line and HaCaT human immortalized keratinocyte cell line were used in this study. Cells were maintained in a humidified incubator at 37 °C with 5% CO_2. The culture medium was refreshed every 2 days. When cells reached 80–90% confluence, they were digested with 0.25% trypsin-EDTA solution for subculture. Cells within passage 3–8 were used for all experiments. Both the L929 murine fibroblast cell line and the HaCaT human immortalized keratinocyte cell line used in this study were purchased from Shanghai Fuheng Biotechnology Co., Ltd.

Preparation of Hydrogel Extracts

Sterile GGT and CSGGT hydrogels (with 1.0 mg/mL Cu_9S_8 loading for CSGGT) were cut into pieces with a total surface area of 1.25 cm^2 per mL of extraction medium.The hydrogel pieces were immersed in complete cell culture medium and incubated at 37 °C for 24 h with gentle shaking, then filtered through sterile filter to obtain the hydrogel extract, which was stored at 4 °C for subsequent cell experiments.

Live/Dead Cell Staining Assay

L929 cells were seeded in 20 mm confocal culture dishes at a density of 5 × 10^4 cells per dish and cultured for 24 h to allow full adhesion.

The original culture medium was replaced with 1 mL of GGT extract, CSGGT extract, or fresh complete medium (blank control). For the photothermal treatment groups, cells were irradiated with a 1064 nm NIR laser at a power density of 0.75 W/cm^2 for 5 min after adding CSGGT extract.After incubation for 12 h, 24 h, and 36 h respectively, cells were gently washed twice with pre-warmed PBS. Calcein-AM/PI staining working solution (2 μM Calcein-AM and 4.5 μM propidium iodide in PBS) was added to each dish, followed by incubation at 37 °C in the dark for 15 min. After washing once with PBS, cells were observed and imaged under a confocal laser scanning microscope (CLSM). Calcein-AM (live cells) was excited at 488 nm and detected at 500–540 nm; PI (dead cells) was excited at 535 nm and detected at 590–640 nm.Cell viability was quantified by counting live and dead cells from at least 3 random fields per sample using ImageJ software, calculated as:

Cell viability (%) = (number of live cells / total number of cells) × 100%

Cell Counting Kit-8 (CCK-8) Viability Assay

L929 cells were seeded in 96-well plates at a density of 5 × 10^3 cells per well (100 μL per well) and cultured for 24 h to adhere.The culture medium was replaced with 100 μL of GGT extract, CSGGT extract, or fresh complete medium. For photothermal groups, cells received 1064 nm laser irradiation at 0.75 W/cm^2 for 5 min. Blank wells containing only medium without cells were set up for background calibration.After 24 h of incubation, 10 μL of CCK-8 reagent was added to each well, and the plates were

incubated at 37 °C in the dark for 1.5 h.The absorbance at 450 nm was measured using a microplate reader (Tecan Infinite M200, Switzerland).

Cell viability was calculated as:

Cell viability (%) = [(OD(sample) – OD(blank)) / (OD(control) – OD(blank))] × 100%

All experiments were performed in 5 replicate wells and repeated 3 times independently.

Scratch Wound Migration Assay

L929 cells were seeded in 6-well plates at a density of 2×10^5 cells per well and cultured until they formed a confluent monolayer (100% confluence).

A straight, uniform scratch was made vertically across the center of each well using a sterile 200 μL pipette tip. Floating cells and cell debris were removed by gently washing 3 times with pre-warmed PBS.

The medium was replaced with serum-free DMEM containing GGT extract, CSGGT extract, or no additives (control). Photothermal groups received 1064 nm laser irradiation at 0.75 W/cm^2 for 5 min.

The scratch area was photographed immediately (0 h) and after 24 h of incubation using an inverted optical microscope. Three representative fields were captured per well.

The scratch area at each time point was measured using ImageJ software. The cell migration rate was calculated as:

Migration rate (%) = [(Area(0 h) – Area(24 h)) / Area(0 h)] × 100%

In Vitro Antibacterial Assay and Bacterial Viability Assessment

Staphylococcus aureus and Escherichia coli were separately suspended in Luria-Bertani

(LB) broth to a final concentration of 10^6 CFU/mL. For Cu_9S_8 treatment, the bacterial suspensions were incubated with Cu_9S_8 nanoparticles at a concentration of 100 μg/mL. For photothermal treatment, the suspensions were irradiated with a 1064 nm laser at a power density of 1 W/cm^2 for 5 min. Various treatment groups—including control, Cu_9S_8 alone, NIR alone, and Cu_9S_8 + NIR—were prepared accordingly. After 30 min of incubation post-treatment, samples were serially diluted 1000-fold in LB broth. Aliquots of 100 μL from each dilution were plated onto LB agar plates, spread evenly using sterile glass beads, and incubated at 37 °C for 24 h for colony-forming unit (CFU) enumeration. The antibacterial efficacy of hydrogels was evaluated using an analogous protocol, wherein GGT or CSGGT hydrogel was added to the bacterial suspensions prior to NIR irradiation (where applicable). Following treatment, all bacterial suspensions were centrifuged at 3000 rpm for 10 min to collect cell pellets. The pellets were washed twice with phosphate-buffered saline (PBS), then dehydrated sequentially in graded ethanol series (30%, 50%, 80%, and 100% v/v, two cycles per concentration, 10 min each). The dehydrated samples were deposited onto silicon wafers and sputter-coated for scanning electron microscopy (SEM) observation of morphological changes. For live/dead fluorescence staining, bacteria were cultured in glass-bottom four-well chamber slides and subjected to the same treatment conditions. After 30 min of incubation, samples were stained with SYTO 9/propidium iodide (PI) dual-fluorescence dye and imaged using confocal laser scanning microscopy (CLSM) to assess bacterial viability and membrane integrity in three-dimensional space.

Transcriptomic Sequencing Analysis

HaCaT human immortalized keratinocytes were cultured as described previously. Two conditioned media were prepared to mimic the inflammatory microenvironment of infected wounds and the integrated therapeutic microenvironment respectively:

Bacterial supernatant group (inflammatory control): S. aureus was cultured to the mid-logarithmic growth phase and adjusted to a concentration of 1×10^7 CFU/mL. Sterile bacterial culture supernatant was collected by centrifugation and 0.22 μm filtration, then mixed with complete DMEM at a volume ratio of 1:9 (final bacterial supernatant content: 10%, v/v).

Therapeutic microenvironment group: The same concentration of S. aureus suspension was co-incubated with CSGGT hydrogel (final Cu_9S_8 concentration: 1.0 mg/mL) at 37 °C for 30 min, followed by 1064 nm NIR laser irradiation at a power density of 1.0 W/cm^2 for 5 min. The treated bacterial supernatant was collected by centrifugation and sterile filtration. CSGGT hydrogel extract was supplemented into the medium, with a final extract concentration of 5% (v/v) and a final bacterial supernatant content of 10% (v/v).

HaCaT cells were seeded in 60 mm culture dishes at a density of 1×10^6 cells per dish, and cultured to 70–80% confluence. The original medium was replaced with the corresponding conditioned medium, and cells were incubated for 24 h under standard culture conditions.

RNA extraction and sequencing

After 24 h of treatment, total RNA was extracted from cells using TRIzol reagent. Qualified RNA samples were sent to Shanghai OE Biotech Co., Ltd for transcriptome

library construction and high-throughput sequencing.

Animal Model

Eight-week-old male BALB/c mice were obtained from the Shanghai Laboratory Animal Center and housed in a specific pathogen-free (SPF) animal facility. All animal procedures were conducted in strict accordance with the ethical guidelines approved by the Institutional Animal Care and Use Committee of Shanghai Jiao Tong University. Mice were maintained under standard laboratory conditions with free access to sterile food and water. A full-thickness skin wound infection model was established by inoculating Staphylococcus aureus onto bilateral excisional wounds created on the dorsal skin of each mouse. All experimental procedures were in accordance with the veterinary guidelines of the American Veterinary Medical Association (AVMA). Approval for our animal experiments was provided by Shanghai Public ealth Clinical Center and Shanghai Jiao Tong University Laboratory nimal Center (A2025464).

In Vivo Anti-Infective Evaluation

To evaluate the anti-infective efficacy of CSGGT, mice were randomly assigned to five treatment groups (n = 5-6 per group): (1) PBS, (2) GGT hydrogel, (3) GGT + NIR, (4) CSGGT hydrogel, and (5) CSGGT + NIR. Detailed in vivo irradiation parameters:

Laser wavelength: 1064 nm

Power density: 0.75 W/cm^2

Irradiation duration: 5 min per treatment

Treatment frequency: single treatment administered on day 3 post-infection

Spot diameter: ~1.2 cm, fully covering the entire wound bed

Wound areas were photographed daily, and body weights were recorded throughout the experiment. On designated days post-treatment, infected skin tissues were harvested, homogenized in 100 μL of sterile saline, and serially diluted for bacterial culture and colony counting. Additionally, excised wound tissues were fixed, sectioned, and subjected to hematoxylin and eosin (H&E) and Masson's trichrome staining to assess histopathological features, inflammatory infiltration, and collagen deposition.

Statistical Analysis

Quantitative data are presented as the mean ± SD. The one-tailed or two-tailed Student's t-test was used to identify any significant differences. All statistical differences were calculated by using Origin 22.

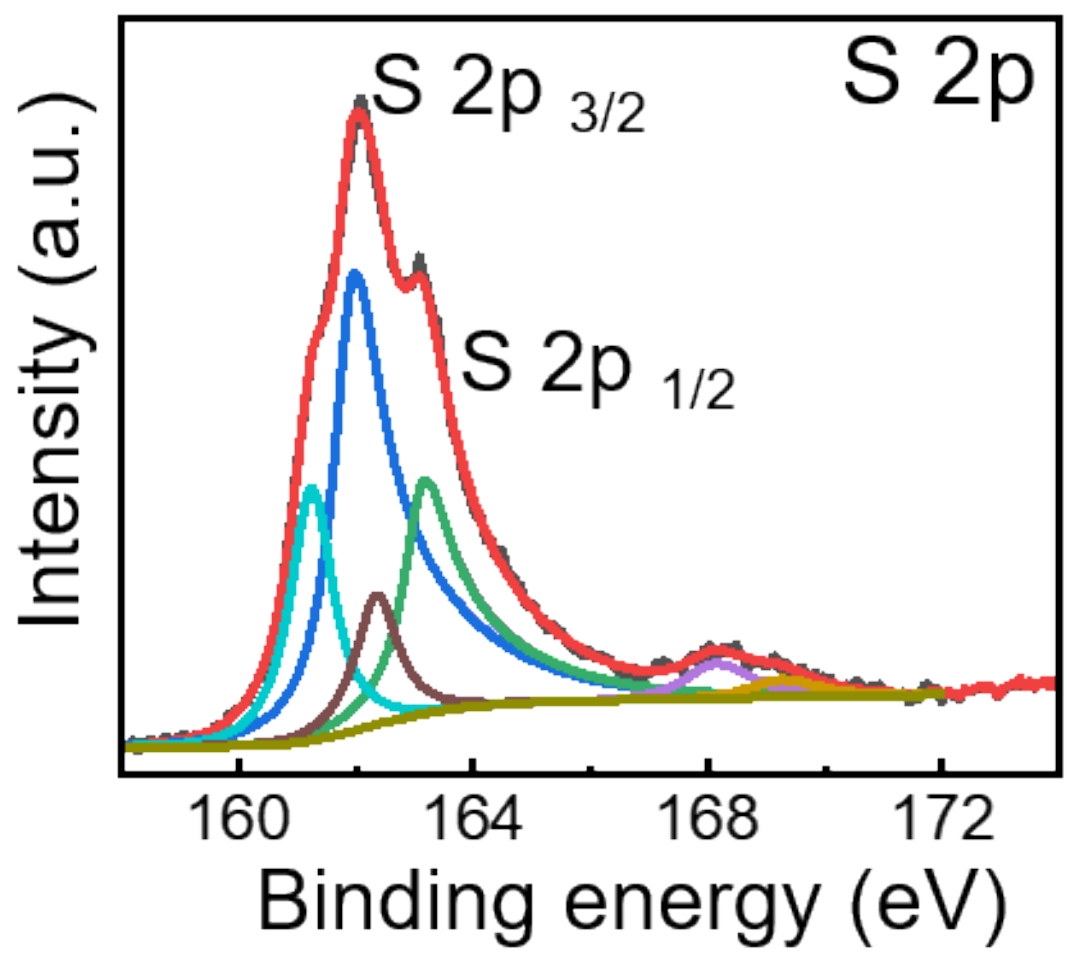

Figure S1. High-resolution XPS spectrum of S 2p for Cu_9S_8 NPs.

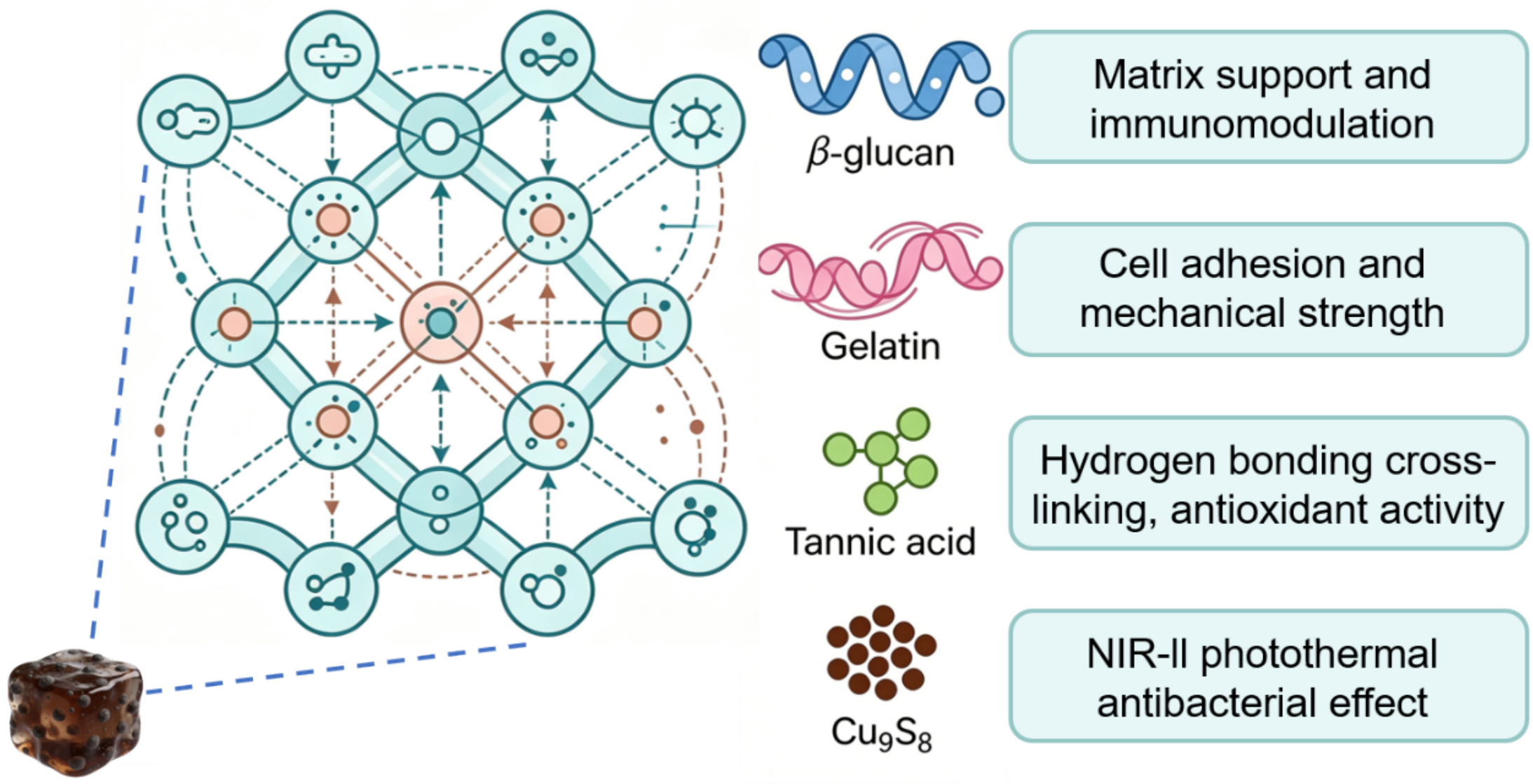

Figure S2. The functional mechanisms of individual components of CSGGT.

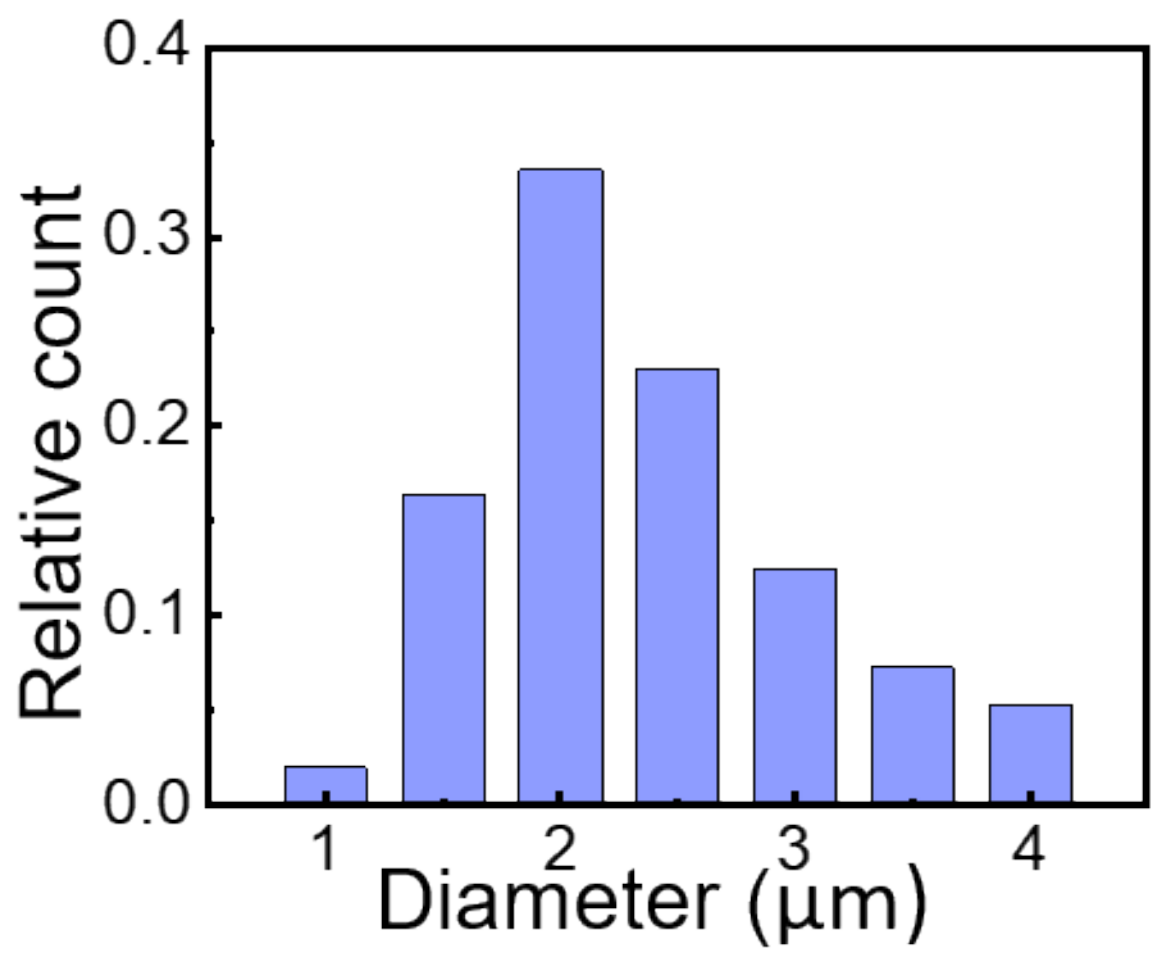

Figure S3. Pore size distribution of CSGGT hydrogel analyzed from SEM images.

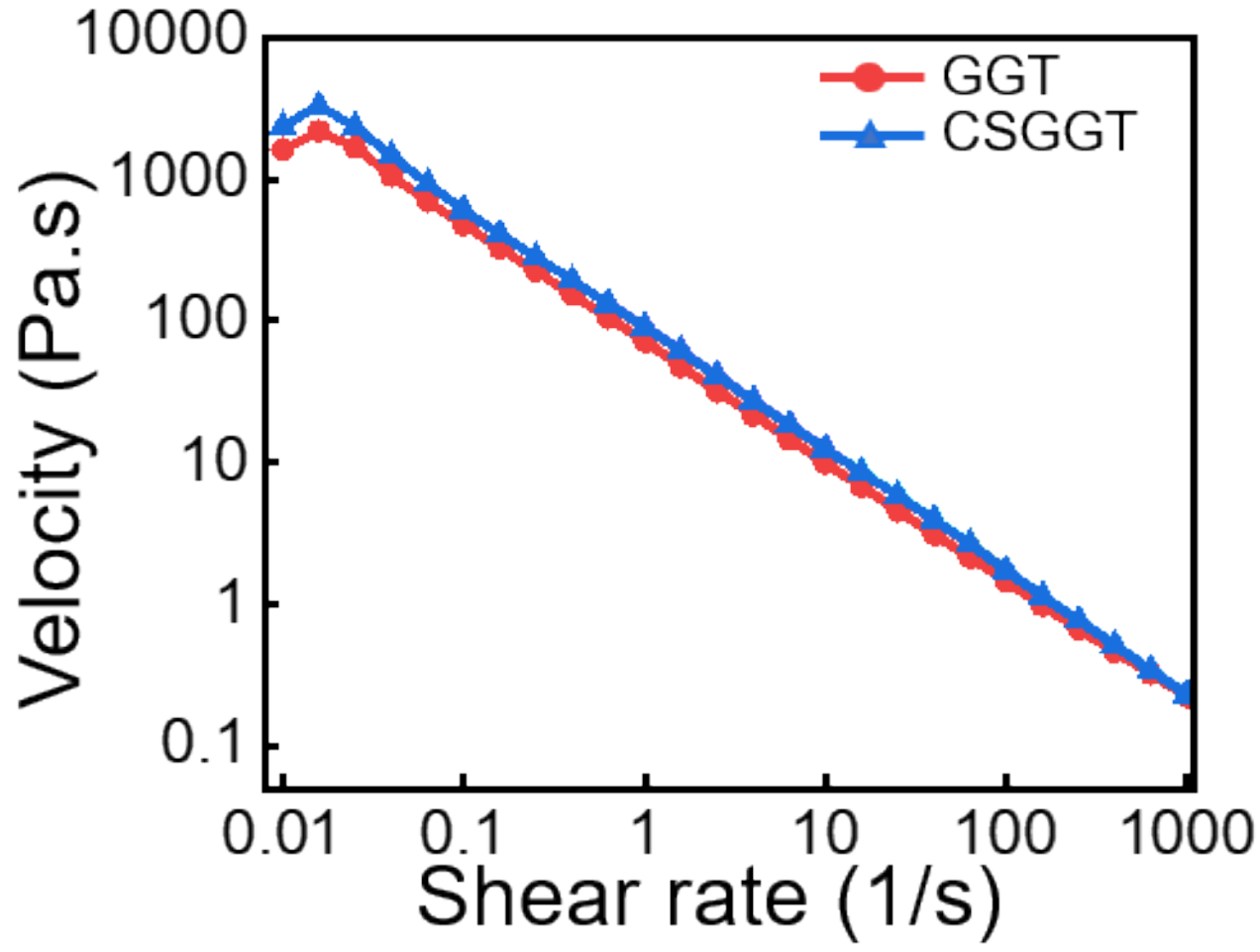

Figure S4. Shear stress–strain curves of GGT and CSGGT hydrogels under shear deformation.

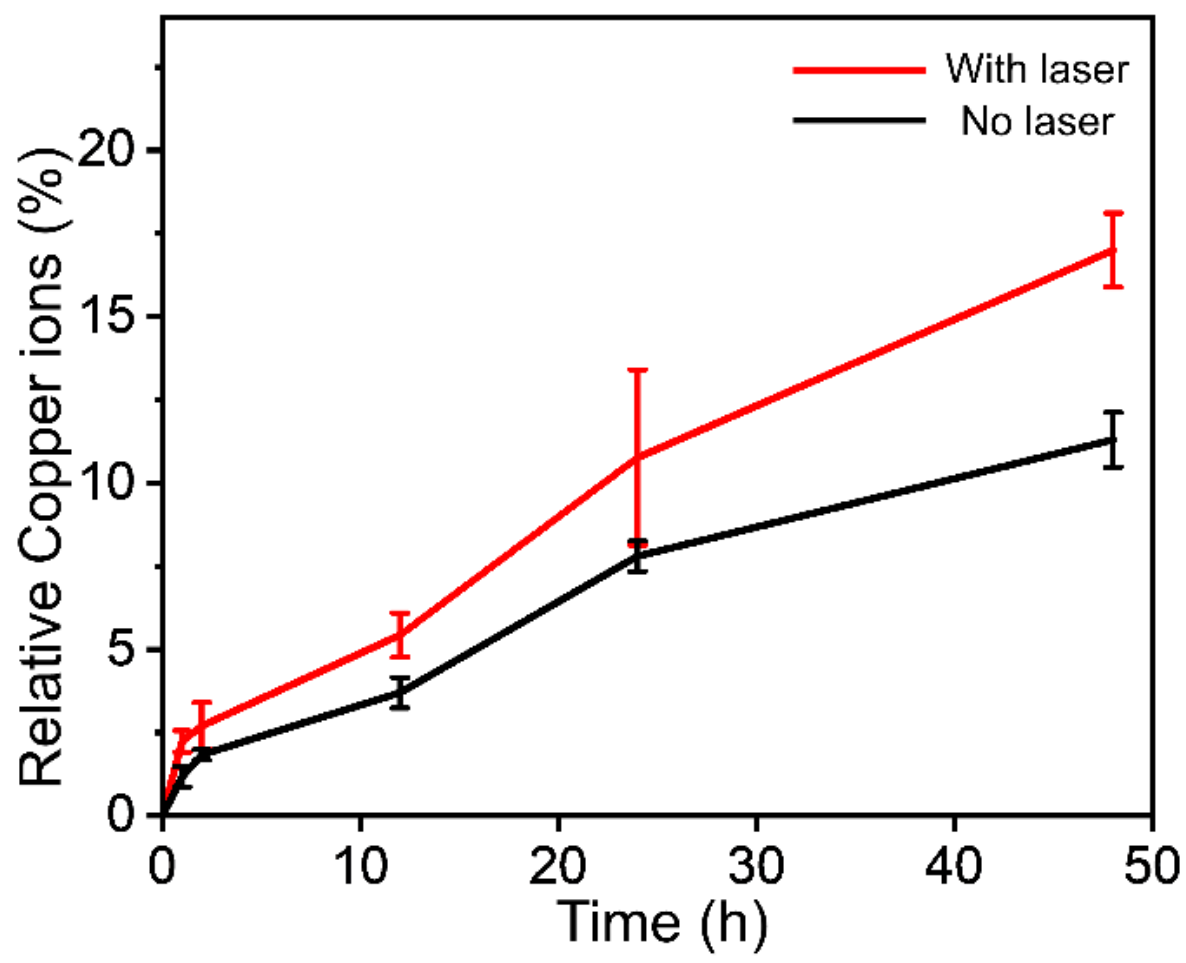

Figure S5. Cu ions release profiles before (a) and after (b) laser irradiation.

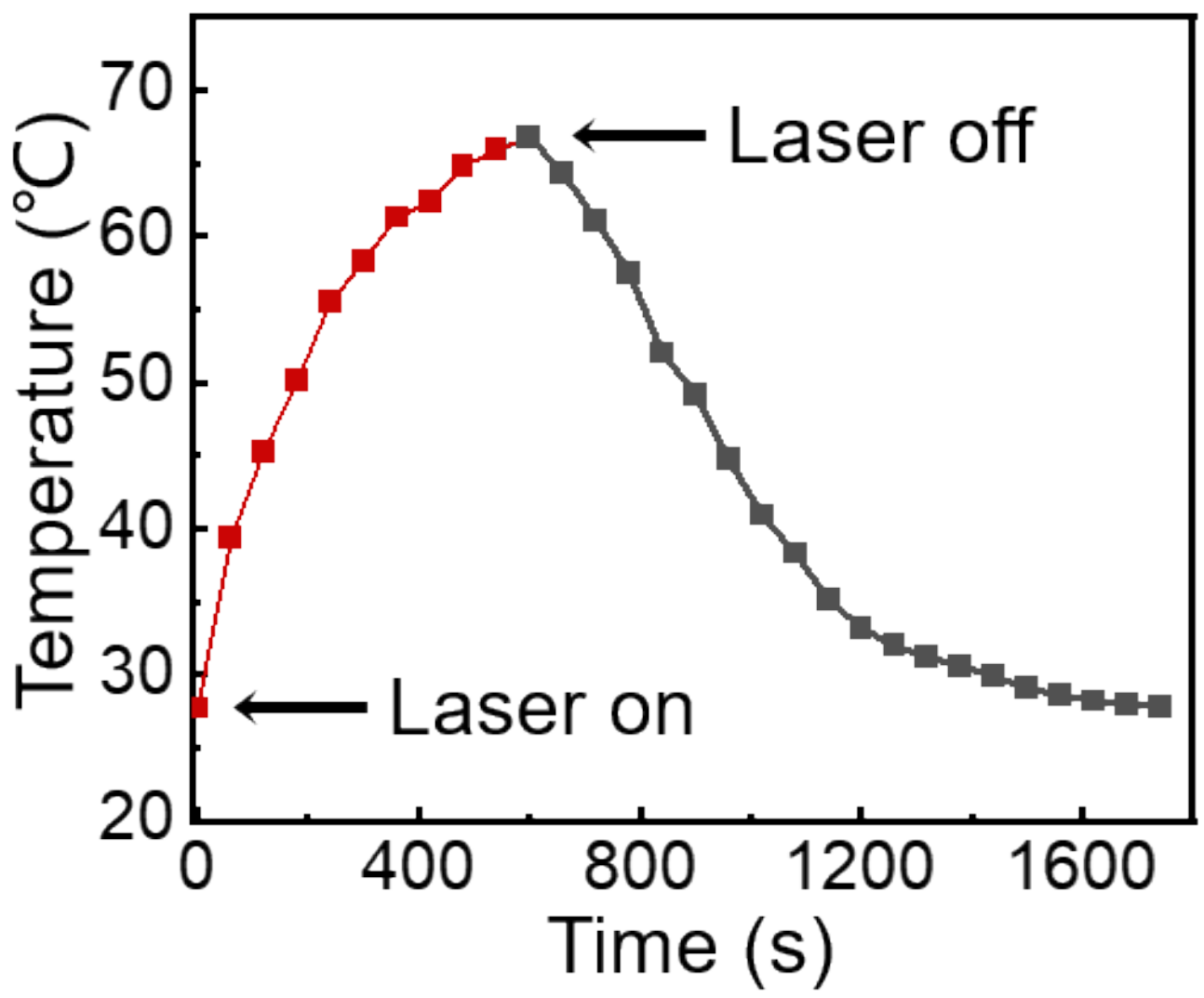

Figure S6. Time-dependent temperature decay curves of Cu_9S_8 aqueous dispersion after laser irradiation.

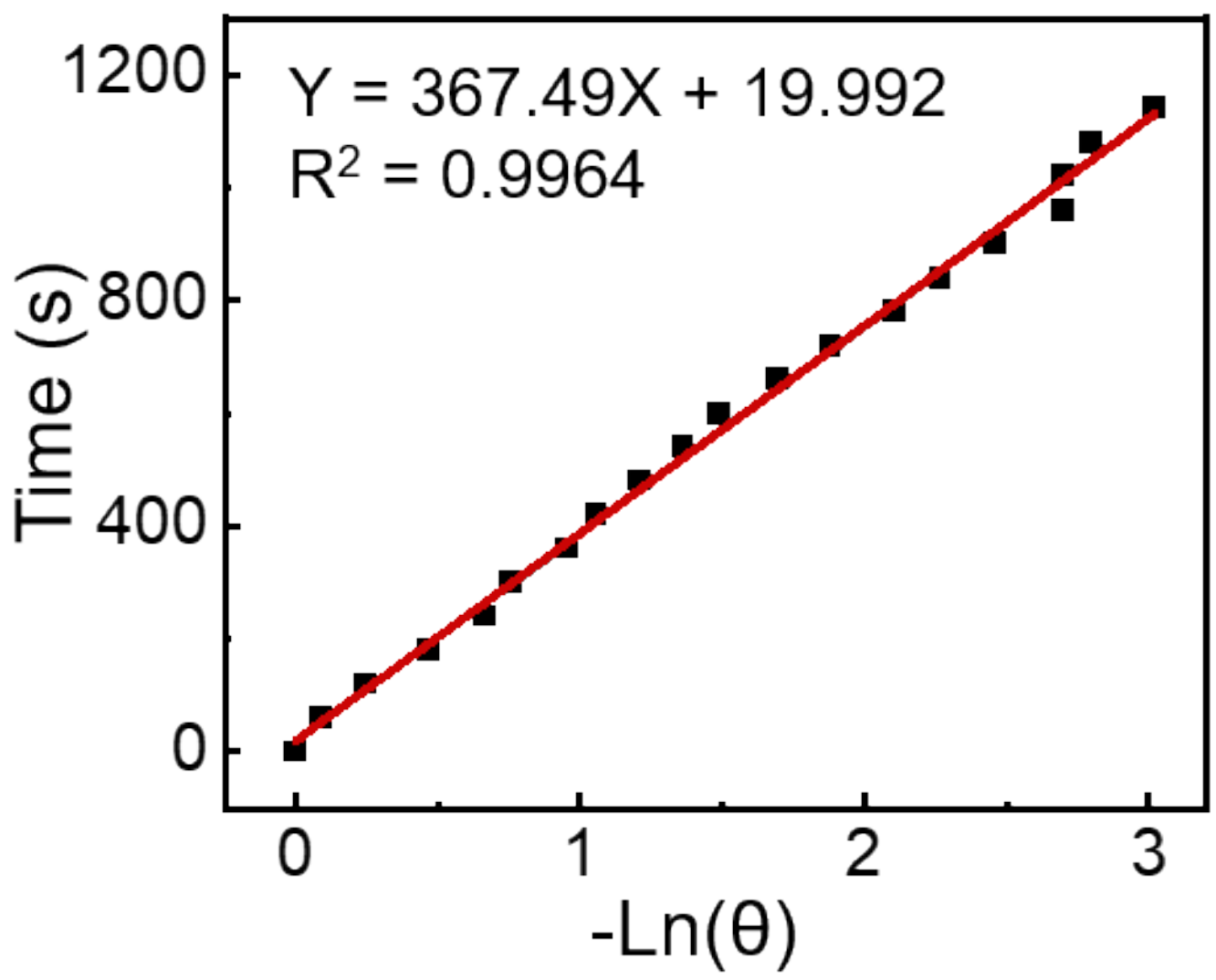

Figure S7. Photothermal conversion efficiency (η) of Cu_9S_8 nanoparticles.

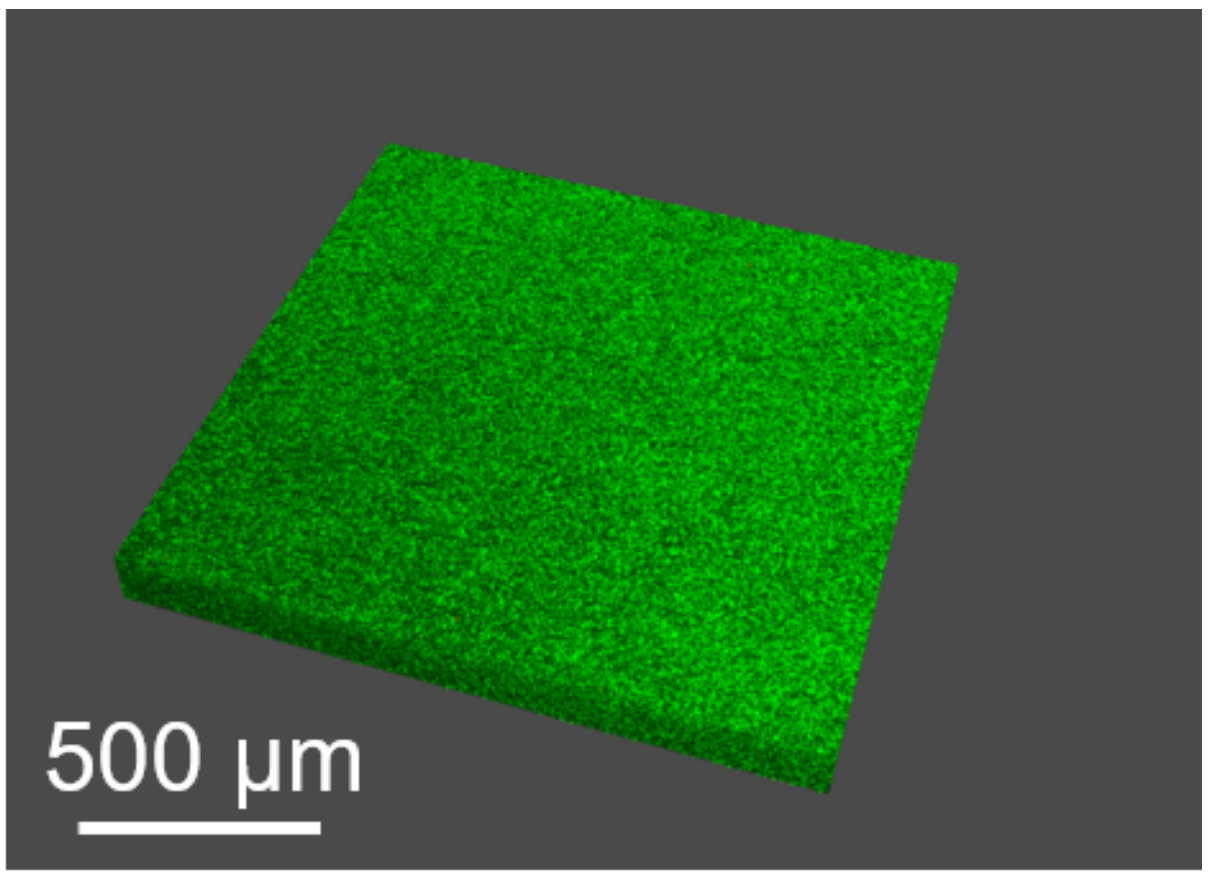

Figure S8. 3D live/dead staining images of *S. aureus*. (scale bar: 500 µm)

The core data include:

(1) Material characterization data: X-ray photoelectron spectroscopy (XPS) survey spectrum and high-resolution Cu 2p spectrum, X-ray diffraction (XRD) patterns, and UV-vis-NIR absorption spectra of Cu_9S_8 nanoparticles;

X-ray photoelectron spectroscopy (XPS) survey spectrum and high-resolution Cu 2p spectrum:

969.95	56766.2	57297.38	57022.17	57023.69	57051.84	57202.75	57085.62	-531.1758
969.9	56717	57266.84	56990.13	56991.65	57019.87	57171.71	57053.98	-549.8359
969.85	56590.4	57236.32	56958.08	56959.61	56987.91	57140.68	57022.36	-645.918
969.8	56978	57205.79	56926.02	56927.55	56955.93	57109.65	56990.73	-227.793
969.75	56642	57175.25	56893.93	56895.46	56923.92	57078.59	56959.06	-533.2461
969.7	57154.6	57144.75	56861.87	56863.41	56891.94	57047.57	56927.43	9.85547
969.65	57053	57114.21	56829.76	56831.3	56859.91	57016.51	56895.76	-61.21094
969.6	57432.2	57083.7	56797.65	56799.2	56827.89	56985.46	56864.09	348.5039
969.55	56929.9	57053.19	56765.55	56767.1	56795.87	56954.43	56832.44	-123.293

969.5	56630.4	57022.69	56733.43	56734.98	56763.83	56923.39	56800.77	-392.293
969.45	56558.5	56992.17	56701.28	56702.84	56731.77	56892.33	56769.07	-433.6719
969.4	56672	56961.68	56669.14	56670.71	56699.71	56861.29	56737.39	-289.6797
969.35	56164	56931.18	56636.98	56638.55	56667.64	56830.24	56705.7	-767.1836
969.3	56264.3	56900.65	56604.78	56606.35	56635.52	56799.15	56673.96	-636.3477
969.25	56271.9	56870.16	56572.61	56574.18	56603.43	56768.1	56642.27	-598.2656
969.2	56290.5	56839.64	56540.37	56541.95	56571.28	56737	56610.51	-549.1367
969.15	56356.2	56809.16	56508.18	56509.76	56539.17	56705.95	56578.8	-452.9609
969.1	56278.9	56778.64	56475.92	56477.51	56507	56674.86	56547.04	-499.7461
969.05	56050.6	56748.15	56443.68	56445.27	56474.85	56643.78	56515.29	-697.5469
969	55877.1	56717.63	56411.39	56412.99	56442.65	56612.67	56483.5	-840.5273
968.95	56303.5	56687.14	56379.12	56380.72	56410.46	56581.59	56451.73	-383.6406
968.9	56515.4	56656.6	56346.79	56348.39	56378.22	56550.45	56419.91	-141.2031
968.85	56456	56626.13	56314.49	56316.11	56346.01	56519.36	56388.13	-170.1289
968.8	56288.7	56595.63	56282.16	56283.78	56313.77	56488.25	56356.32	-306.9258
968.75	55874.8	56565.13	56249.82	56251.44	56281.51	56457.13	56324.49	-690.3242
968.7	55991.9	56534.6	56217.43	56219.05	56249.21	56425.98	56292.63	-542.6992
968.65	56213.4	56504.12	56185.07	56186.7	56216.94	56394.88	56260.81	-290.7227
968.6	56223.1	56473.62	56152.67	56154.3	56184.63	56363.74	56228.96	-250.5195
968.55	56200.4	56443.12	56120.25	56121.89	56152.31	56332.6	56197.09	-242.7227
968.5	55682.9	56412.64	56087.84	56089.48	56119.98	56301.47	56165.22	-729.7461
968.45	55723.1	56382.16	56055.4	56057.05	56087.63	56270.33	56133.34	-659.0547

968.4	55975.4	56351.64	56022.91	56024.57	56055.24	56239.15	56101.43	-376.2422
968.35	55881.1	56321.17	55990.46	55992.11	56022.88	56208.02	56069.54	-440.0703
968.3	55672.7	56290.68	55957.95	55959.61	55990.46	56176.85	56037.61	-617.9766
968.25	55503.4	56260.2	55925.45	55927.12	55958.05	56145.69	56005.7	-756.8047
968.2	55427.3	56229.73	55892.93	55894.6	55925.63	56114.53	55973.77	-802.4297
968.15	55009.7	56199.21	55860.35	55862.03	55893.14	56083.32	55941.79	-1189.516
968.1	55248.7	56168.73	55827.77	55829.45	55860.66	56052.13	55909.81	-920.0273
968.05	55517.7	56138.23	55795.18	55796.86	55828.15	56020.92	55877.82	-620.5313
968	55276.5	56107.75	55762.57	55764.26	55795.64	55989.73	55845.84	-831.25
967.95	55618.1	56077.24	55729.91	55731.61	55763.08	55958.49	55813.8	-459.1406
967.9	55407	56046.77	55697.27	55698.97	55730.54	55927.29	55781.79	-639.7656
967.85	55489.7	56016.28	55664.59	55666.3	55697.95	55896.06	55749.75	-526.5781
967.8	55325.4	55985.8	55631.91	55633.61	55665.36	55864.83	55717.71	-660.3984
967.75	55155	55955.3	55599.19	55600.9	55632.73	55833.59	55685.64	-800.3047
967.7	55499.6	55924.82	55566.45	55568.16	55600.09	55802.34	55653.56	-425.2188
967.65	55287.9	55894.34	55533.7	55535.42	55567.44	55771.09	55621.48	-606.4414
967.6	55222.6	55863.85	55500.9	55502.63	55534.74	55739.82	55589.36	-641.2461
967.55	54932.1	55833.37	55468.11	55469.84	55502.04	55708.56	55557.25	-901.2656
967.5	54889.2	55802.89	55435.28	55437.02	55469.32	55677.28	55525.11	-913.6914
967.45	54821.3	55772.44	55402.46	55404.2	55436.6	55646.03	55492.99	-951.1367
967.4	55049.5	55741.96	55369.59	55371.34	55403.83	55614.74	55460.83	-692.457
967.35	55118.2	55711.47	55336.69	55338.44	55371.02	55583.43	55428.64	-593.2695

967.3	55174.6	55681	55303.78	55305.54	55338.22	55552.13	55396.46	-506.4023
967.25	55119.1	55650.53	55270.84	55272.61	55305.38	55520.82	55364.25	-531.4258
967.2	54933.6	55620.08	55237.91	55239.67	55272.54	55489.53	55332.05	-686.4766
967.15	54597.9	55589.63	55204.94	55206.71	55239.68	55458.21	55299.84	-991.7305
967.1	54619.5	55559.15	55171.92	55173.7	55206.77	55426.88	55267.58	-939.6523
967.05	54399.3	55528.69	55138.9	55140.68	55173.84	55395.54	55235.32	-1129.391
967	54346.6	55498.23	55105.84	55107.63	55140.89	55364.2	55203.04	-1151.625
966.95	54694.2	55467.78	55072.77	55074.56	55107.92	55332.85	55170.75	-773.5781
966.9	54314.6	55437.34	55039.69	55041.48	55074.94	55301.51	55138.46	-1122.734
966.85	53898	55406.87	55006.54	55008.34	55041.9	55270.13	55106.13	-1508.867
966.8	54146.7	55376.44	54973.41	54975.22	55008.88	55238.77	55073.81	-1229.742
966.75	54616.9	55345.99	54940.23	54942.05	54975.8	55207.39	55041.46	-729.0938
966.7	54217.9	55315.52	54907	54908.82	54942.68	55175.97	55009.05	-1097.617
966.65	54216.4	55285.07	54873.77	54875.59	54909.55	55144.57	54976.66	-1068.672
966.6	54392.5	55254.63	54840.51	54842.34	54876.4	55113.16	54944.26	-862.1328
966.55	54512.8	55224.2	54807.23	54809.06	54843.22	55081.75	54911.85	-711.3984
966.5	54318	55193.76	54773.91	54775.75	54810.02	55050.32	54879.41	-875.7617
966.45	54238.7	55163.35	54740.59	54742.44	54776.8	55018.91	54846.98	-924.6523
966.4	54030.8	55132.92	54707.22	54709.07	54743.54	54987.47	54814.5	-1102.121
966.35	54341.4	55102.52	54673.84	54675.7	54710.27	54956.04	54782.04	-761.1211
966.3	54220.5	55072.11	54640.43	54642.29	54676.97	54924.6	54749.54	-851.6094
966.25	54143	55041.71	54606.99	54608.86	54643.64	54893.15	54717.04	-898.707

966.2	54084.4	55011.3	54573.51	54575.38	54610.27	54861.68	54684.5	-926.8984
966.15	53933.4	54980.91	54540.02	54541.89	54576.89	54830.21	54651.96	-1047.512
966.1	54200.7	54950.55	54506.52	54508.4	54543.5	54798.77	54619.43	-749.8516
966.05	54276.4	54920.19	54472.98	54474.87	54510.08	54767.31	54586.88	-643.7891
966	54269.6	54889.82	54439.4	54441.29	54476.61	54735.82	54554.29	-620.2148
965.95	53667.3	54859.45	54405.79	54407.68	54443.11	54704.33	54521.68	-1192.145
965.9	53677.1	54829.11	54372.17	54374.07	54409.61	54672.86	54489.08	-1152.012
965.85	53778.7	54798.79	54338.52	54340.43	54376.07	54641.38	54456.47	-1020.09
965.8	53330.3	54768.45	54304.83	54306.75	54342.5	54609.88	54423.83	-1438.152
965.75	53494.5	54738.14	54271.12	54273.04	54308.9	54578.38	54391.18	-1243.637
965.7	53565.7	54707.83	54237.38	54239.31	54275.28	54546.88	54358.51	-1142.129
965.65	53969.8	54677.52	54203.6	54205.54	54241.61	54515.36	54325.82	-707.7188
965.6	53499.2	54647.24	54169.8	54171.74	54207.93	54483.85	54293.13	-1148.039
965.55	53380.6	54616.96	54135.98	54137.92	54174.22	54452.33	54260.42	-1236.359
965.5	53719.5	54586.71	54102.13	54104.08	54140.5	54420.82	54227.71	-867.2109
965.45	53679.7	54556.44	54068.23	54070.18	54106.71	54389.28	54194.95	-876.7422
965.4	53400.2	54526.21	54034.32	54036.28	54072.92	54357.76	54162.21	-1126.012
965.35	53381.2	54495.99	54000.38	54002.34	54039.1	54326.23	54129.44	-1114.793
965.3	53566.1	54465.77	53966.39	53968.36	54005.23	54294.69	54096.65	-899.668
965.25	53632.1	54435.58	53932.39	53934.37	53971.36	54263.16	54063.87	-803.4805
965.2	53073.8	54405.41	53898.37	53900.36	53937.45	54231.64	54031.07	-1331.613
965.15	53342	54375.25	53864.3	53866.3	53903.51	54200.1	53998.25	-1033.246

965.1	53373.5	54345.1	53830.2	53832.2	53869.53	54168.55	53965.42	-971.5977
965.05	53386.2	54314.95	53796.06	53798.07	53835.52	54137	53932.56	-928.7539
965	53498	54284.86	53761.93	53763.94	53801.5	54105.48	53899.73	-786.8633
964.95	53504.2	54254.75	53727.73	53729.74	53767.43	54073.92	53866.84	-750.5508
964.9	52952.1	54224.71	53693.54	53695.57	53733.37	54042.41	53833.99	-1272.605
964.85	53397	54194.64	53659.28	53661.31	53699.23	54010.86	53801.09	-797.6445
964.8	53185.5	54164.63	53625.03	53627.07	53665.11	53979.34	53768.21	-979.1328
964.75	52829.9	54134.64	53590.74	53592.78	53630.94	53947.82	53735.31	-1304.742
964.7	52896	54104.64	53556.39	53558.44	53596.72	53916.27	53702.38	-1208.641
964.65	53454	54074.7	53522.05	53524.1	53562.5	53884.77	53669.47	-620.7031
964.6	53360.6	54044.74	53487.63	53489.68	53528.21	53853.21	53636.51	-684.1406
964.55	53122.6	54014.85	53453.22	53455.29	53493.93	53821.71	53603.58	-892.25
964.5	53006.6	53984.96	53418.76	53420.84	53459.61	53790.19	53570.62	-978.3633
964.45	52999.9	53955.12	53384.29	53386.37	53425.26	53758.69	53537.67	-955.2227
964.4	53295.5	53925.29	53349.77	53351.85	53390.87	53727.17	53504.69	-629.7891
964.35	53193.9	53895.52	53315.23	53317.33	53356.47	53695.69	53471.73	-701.6172
964.3	52988.8	53865.76	53280.68	53282.78	53322.05	53664.21	53438.77	-876.9609
964.25	53061.3	53836.02	53246.06	53248.16	53287.56	53632.71	53405.76	-774.7148
964.2	52832.6	53806.32	53211.43	53213.54	53253.07	53601.23	53372.77	-973.7227
964.15	52788.7	53776.68	53176.78	53178.9	53218.55	53569.78	53339.79	-987.9766
964.1	52961.3	53747.04	53142.07	53144.2	53183.98	53538.31	53306.77	-785.7383
964.05	52915.6	53717.46	53107.37	53109.5	53149.41	53506.88	53273.78	-801.8633

964	52565.1	53687.91	53072.62	53074.76	53114.8	53475.45	53240.78	-1122.813
963.95	52809.4	53658.4	53037.84	53039.98	53080.15	53444.02	53207.77	-849.0039
963.9	53753.1	53628.93	53003.02	53005.17	53045.47	53412.6	53174.75	124.1758
963.85	53501.1	53599.5	52968.18	52970.34	53010.77	53381.2	53141.74	-98.40234
963.8	52999	53570.14	52933.34	52935.5	52976.06	53349.84	53108.75	-571.1406
963.75	52904.5	53540.79	52898.43	52900.6	52941.29	53318.46	53075.73	-636.293
963.7	53087.3	53511.51	52863.51	52865.69	52906.51	53287.11	53042.72	-424.207
963.65	53076	53482.28	52828.56	52830.75	52871.71	53255.79	53009.72	-406.2773
963.6	53028	53453.11	52793.6	52795.79	52836.89	53224.49	52976.74	-425.1133
963.55	52915.8	53423.98	52758.59	52760.79	52802.02	53193.2	52943.75	-508.1758
963.5	52512.4	53394.9	52723.56	52725.77	52767.13	53161.93	52910.76	-882.5
963.45	52589.5	53365.87	52688.49	52690.71	52732.21	53130.66	52877.77	-776.3672
963.4	52956.8	53336.91	52653.41	52655.63	52697.27	53099.44	52844.8	-380.1094
963.35	53154.9	53308.01	52618.29	52620.52	52662.3	53068.23	52811.84	-153.1094
963.3	52317.5	53279.15	52583.14	52585.37	52627.29	53037.04	52778.87	-961.6523
963.25	52679	53250.36	52547.95	52550.2	52592.25	53005.86	52745.91	-571.3555
963.2	52861.4	53221.64	52512.75	52515	52557.2	52974.73	52712.96	-360.2383
963.15	52595.4	53192.98	52477.52	52479.77	52522.11	52943.61	52680.04	-597.582
963.1	52656.1	53164.4	52442.25	52444.52	52486.99	52912.53	52647.11	-508.2969
963.05	52066	53135.88	52406.96	52409.23	52451.85	52881.47	52614.21	-1069.883
963	52085.9	53107.42	52371.63	52373.91	52416.67	52850.43	52581.31	-1021.52
962.95	52122.1	53079.03	52336.27	52338.55	52381.46	52819.41	52548.41	-956.9297

962.9	52409.2	53050.73	52300.88	52303.18	52346.23	52788.43	52515.55	-641.5273
962.85	52265.7	53022.51	52265.46	52267.76	52310.96	52757.48	52482.7	-756.8086
962.8	52151.4	52994.35	52230.01	52232.32	52275.66	52726.55	52449.85	-842.9531
962.75	51714.4	52966.28	52194.53	52196.85	52240.33	52695.66	52417.04	-1251.883
962.7	51665.6	52938.29	52159	52161.32	52204.96	52664.79	52384.22	-1272.684
962.65	51880.8	52910.37	52123.44	52125.77	52169.55	52633.94	52351.42	-1029.566
962.6	51872.6	52882.54	52087.84	52090.18	52134.11	52603.13	52318.64	-1009.938
962.55	51931.3	52854.82	52052.23	52054.57	52098.65	52572.37	52285.91	-923.5195
962.5	51733.4	52827.18	52016.58	52018.93	52063.16	52541.64	52253.19	-1093.785
962.45	51856.6	52799.65	51980.9	51983.26	52027.64	52510.95	52220.5	-943.0508
962.4	51873.4	52772.22	51945.18	51947.55	51992.08	52480.29	52187.84	-898.8203
962.35	51782.6	52744.9	51909.44	51911.82	51956.5	52449.68	52155.21	-962.2969
962.3	51541.6	52717.66	51873.64	51876.02	51920.86	52419.09	52122.59	-1176.055
962.25	51512.6	52690.54	51837.82	51840.21	51885.2	52388.55	52090.03	-1177.938
962.2	51706.8	52663.54	51801.97	51804.37	51849.52	52358.06	52057.5	-956.7383
962.15	51578.6	52636.65	51766.08	51768.49	51813.79	52327.61	52025	-1058.051
962.1	51446.6	52609.91	51730.17	51732.59	51778.05	52297.23	51992.56	-1163.309
962.05	51241.1	52583.26	51694.21	51696.64	51742.25	52266.86	51960.15	-1342.16
962	51422.7	52556.76	51658.22	51660.65	51706.43	52236.55	51927.79	-1134.059
961.95	51527.9	52530.38	51622.19	51624.63	51670.56	52206.29	51895.47	-1002.48
961.9	51236.3	52504.16	51586.15	51588.6	51634.69	52176.09	51863.23	-1267.859
961.85	51360.3	52478.07	51550.03	51552.49	51598.74	52145.92	51831.01	-1117.766

961.8	51578.3	52452.14	51513.91	51516.38	51562.79	52115.84	51798.88	-873.8398
961.75	51502.8	52426.38	51477.77	51480.25	51526.82	52085.81	51766.82	-923.582
961.7	51282.6	52400.75	51441.56	51444.05	51490.79	52055.82	51734.79	-1118.152
961.65	51514.7	52375.32	51405.35	51407.84	51454.75	52025.91	51702.88	-860.625
961.6	51450.6	52350.09	51369.11	51371.61	51418.68	51996.09	51671.04	-899.4844
961.55	51014.9	52324.99	51332.8	51335.31	51382.55	51966.29	51639.26	-1310.09
961.5	50917.3	52300.09	51296.48	51299	51346.4	51936.57	51607.58	-1382.793
961.45	50722.4	52275.38	51260.11	51262.64	51310.2	51906.9	51575.97	-1552.98
961.4	50636.7	52250.89	51223.72	51226.25	51273.99	51877.33	51544.47	-1614.188
961.35	50834.3	52226.61	51187.28	51189.82	51237.73	51847.82	51513.08	-1392.305
961.3	50818	52202.52	51150.8	51153.36	51201.43	51818.38	51481.77	-1384.52
961.25	50901.9	52178.69	51114.31	51116.88	51165.12	51789.03	51450.61	-1276.793
961.2	50808.8	52155.08	51077.78	51080.35	51128.77	51759.75	51419.55	-1346.281
961.15	50901.8	52131.71	51041.2	51043.77	51092.36	51730.55	51388.61	-1229.91
961.1	51024.6	52108.61	51004.58	51007.17	51055.93	51701.44	51357.82	-1084.008
961.05	51044.3	52085.77	50967.94	50970.54	51019.48	51672.41	51327.16	-1041.469
961	51043.9	52063.19	50931.26	50933.86	50982.98	51643.47	51296.65	-1019.293
960.95	50754.5	52040.91	50894.55	50897.16	50946.45	51614.63	51266.31	-1286.406
960.9	51006.9	52018.93	50857.8	50860.43	50909.89	51585.88	51236.13	-1012.027
960.85	51167.4	51997.27	50821.02	50823.65	50873.3	51557.25	51206.13	-829.8711
960.8	51047.8	51975.92	50784.21	50786.85	50836.68	51528.71	51176.32	-928.1211
960.75	50844.3	51954.91	50747.37	50750.02	50800.02	51500.27	51146.7	-1110.613

960.7	50281.9	51934.28	50710.52	50713.18	50763.36	51471.97	51117.32	-1652.383
960.65	50729.9	51913.98	50673.6	50676.27	50726.64	51443.75	51088.12	-1184.078
960.6	51007.6	51894.08	50636.66	50639.34	50689.89	51415.66	51059.16	-886.4766
960.55	50936.3	51874.56	50599.68	50602.37	50653.11	51387.69	51030.44	-938.2578
960.5	51152.4	51855.44	50562.67	50565.37	50616.29	51359.82	51001.96	-703.043
960.45	50732	51836.75	50525.63	50528.34	50579.45	51332.1	50973.76	-1104.754
960.4	50680.4	51818.51	50488.57	50491.29	50542.58	51304.51	50945.84	-1138.113
960.35	50934.7	51800.74	50451.45	50454.18	50505.66	51277.05	50918.21	-866.0391
960.3	51115.3	51783.43	50414.32	50417.06	50468.73	51249.73	50890.88	-668.1328
960.25	50916.4	51766.64	50377.17	50379.92	50431.78	51222.57	50863.89	-850.2383
960.2	50660.5	51750.34	50339.97	50342.73	50394.79	51195.53	50837.21	-1089.84
960.15	50694.4	51734.59	50302.76	50305.52	50357.77	51168.66	50810.91	-1040.191
960.1	51165.7	51719.41	50265.5	50268.28	50320.72	51141.96	50784.97	-553.7148
960.05	51313.4	51704.79	50228.22	50231	50283.64	51115.4	50759.4	-391.3945
960	50872.2	51690.78	50190.92	50193.71	50246.55	51089.02	50734.25	-818.5781
959.95	51059.3	51677.38	50153.58	50156.38	50209.41	51062.81	50709.51	-618.0781
959.9	51204.5	51664.63	50116.21	50119.03	50172.25	51036.77	50685.2	-460.125
959.85	51059.1	51652.56	50078.81	50081.64	50135.07	51010.93	50661.37	-593.4609
959.8	50928.1	51641.17	50041.39	50044.23	50097.85	50985.27	50638	-713.0703
959.75	50809.7	51630.51	50003.95	50006.8	50060.63	50959.81	50615.13	-820.8086
959.7	51054.4	51620.57	49966.47	49969.32	50023.36	50934.54	50592.76	-566.168
959.65	51764.9	51611.42	49928.97	49931.84	49986.07	50909.48	50570.94	153.4805

959.6	51905.1	51603.09	49891.45	49894.32	49948.77	50884.66	50549.69	302.0156
959.55	51523.7	51595.55	49853.91	49856.79	49911.44	50860.04	50529	-71.85156
959.5	51520.9	51588.88	49816.34	49819.24	49874.09	50835.66	50508.92	-67.98047
959.45	50655.2	51583.08	49778.75	49781.66	49836.72	50811.5	50489.45	-927.8789
959.4	50906.7	51578.2	49741.14	49744.06	49799.34	50787.59	50470.64	-671.4961
959.35	51269.3	51574.27	49703.5	49706.43	49761.91	50763.94	50452.49	-304.9688
959.3	51025.6	51571.29	49665.84	49668.78	49724.47	50740.53	50435.02	-545.6836
959.25	51535.9	51569.3	49628.16	49631.11	49687.02	50717.38	50418.26	-33.40234
959.2	51804.5	51568.34	49590.45	49593.41	49649.54	50694.51	50402.23	236.1641
959.15	51286.7	51568.43	49552.72	49555.7	49612.03	50671.91	50386.95	-281.7266
959.1	51618.9	51569.66	49514.97	49517.96	49574.51	50649.63	50372.48	49.23438
959.05	52207.3	51571.97	49477.2	49480.2	49536.96	50627.62	50358.79	635.332
959	51734.7	51575.45	49439.42	49442.43	49499.42	50605.93	50345.93	159.2539
958.95	51362.5	51580.09	49401.62	49404.63	49461.85	50584.55	50333.91	-217.5938
958.9	51403.5	51585.97	49363.8	49366.83	49424.27	50563.51	50322.78	-182.4727
958.85	51609.9	51593.15	49325.96	49329	49386.66	50542.81	50312.57	16.74609
958.8	51942.5	51601.56	49288.11	49291.16	49349.04	50522.45	50303.23	340.9414
958.75	52208.2	51611.28	49250.23	49253.29	49311.4	50502.44	50294.84	596.9219
958.7	51920.2	51622.35	49212.34	49215.42	49273.75	50482.8	50287.41	297.8477
958.65	52110	51634.82	49174.45	49177.54	49236.1	50463.56	50280.97	475.1836
958.6	51904.8	51648.75	49136.54	49139.64	49198.43	50444.73	50275.57	256.0508
958.55	51737.9	51664.07	49098.61	49101.72	49160.75	50426.28	50271.16	73.82422

958.5	51925.2	51680.86	49060.66	49063.79	49123.04	50408.24	50267.79	244.3359
958.45	52071.1	51699.17	49022.71	49025.85	49085.34	50390.65	50265.48	371.9297
958.4	52288.7	51719.01	48984.74	48987.89	49047.61	50373.49	50264.25	569.6875
958.35	52470.8	51740.5	48946.76	48949.92	49009.88	50356.82	50264.16	730.3008
958.3	52176	51763.54	48908.79	48911.96	48972.16	50340.63	50265.16	412.4609
958.25	52164.1	51788.2	48870.79	48873.97	48934.41	50324.9	50267.28	375.9063
958.2	52407.3	51814.51	48832.78	48835.97	48896.66	50309.68	50270.54	592.7891
958.15	52598.1	51842.52	48794.76	48797.97	48858.89	50294.99	50274.96	755.582
958.1	52557.1	51872.34	48756.74	48759.96	48821.13	50280.87	50280.6	684.7656
958.05	52358.1	51903.82	48718.71	48721.94	48783.36	50267.28	50287.38	454.2852
958	52393.1	51937.06	48680.66	48683.91	48745.57	50254.25	50295.32	456.043
957.95	52524.1	51972.13	48642.64	48645.89	48707.8	50241.86	50304.5	551.9727
957.9	52711	52009	48604.58	48607.85	48670	50230.04	50314.84	702.0039
957.85	52649	52047.83	48566.54	48569.82	48632.23	50218.92	50326.48	601.1719
957.8	52452.9	52088.4	48528.46	48531.76	48594.42	50208.39	50339.23	364.4961
957.75	52579.3	52130.9	48490.41	48493.72	48556.64	50198.57	50353.21	448.4023
957.7	52653.3	52175.3	48452.34	48455.66	48518.84	50189.44	50368.39	478.0039
957.65	52764	52221.62	48414.27	48417.61	48481.04	50181.04	50384.76	542.3828
957.6	52635.5	52269.99	48376.2	48379.54	48443.23	50173.42	50402.39	365.5078
957.55	53125	52320.24	48338.13	48341.49	48405.44	50166.55	50421.15	804.7617
957.5	52768.9	52372.45	48300.05	48303.42	48367.63	50160.47	50441.07	396.4492
957.45	52206.2	52426.65	48261.97	48265.35	48329.83	50155.22	50462.15	-220.4492

957.4	52408.3	52482.84	48223.88	48227.28	48292.02	50150.83	50484.36	-74.54297
957.35	52660.1	52541.17	48185.8	48189.21	48254.22	50147.38	50507.76	118.9336
957.3	52794.1	52601.39	48147.71	48151.13	48216.41	50144.79	50532.18	192.7109
957.25	53077.6	52663.64	48109.63	48113.06	48178.61	50143.17	50557.67	413.9609
957.2	53034.3	52727.91	48071.54	48074.99	48140.81	50142.53	50584.19	306.3906
957.15	53209.9	52794.2	48033.45	48036.91	48103.01	50142.91	50611.71	415.6953
957.1	53355.9	52862.66	47995.38	47998.86	48065.23	50144.41	50640.29	493.2422
957.05	53430.9	52932.99	47957.29	47960.79	48027.43	50146.96	50669.69	497.9063
957	53557.4	53005.36	47919.23	47922.73	47989.66	50150.66	50699.98	552.0391
956.95	53887.8	53079.73	47881.16	47884.69	47951.89	50155.54	50731.1	808.0703
956.9	53586.2	53156.1	47843.12	47846.65	47914.14	50161.66	50762.99	430.1016
956.85	53588.4	53234.6	47805.09	47808.64	47876.41	50169.12	50795.69	353.8008
956.8	53871.1	53314.9	47767.04	47770.61	47838.66	50177.83	50828.92	556.2031
956.75	53539.1	53397.15	47729.02	47732.59	47800.95	50187.93	50862.73	141.9531
956.7	53461.4	53481.31	47691	47694.59	47763.23	50199.45	50897.04	-19.91406
956.65	53561.8	53567.37	47653	47656.61	47725.54	50212.46	50931.76	-5.56641
956.6	53872.6	53655.41	47615	47618.62	47687.84	50227.06	50966.88	217.1875
956.55	53828.8	53745.14	47577.02	47580.65	47650.17	50243.22	51002.14	83.66016
956.5	53340.8	53836.65	47539.05	47542.7	47612.52	50261.04	51037.54	-495.8477
956.45	53873.5	53929.89	47501.09	47504.75	47574.87	50280.59	51072.93	-56.39063
956.4	54144	54024.8	47463.13	47466.81	47537.23	50301.93	51108.22	119.1953
956.35	54087.3	54121.54	47425.2	47428.89	47499.61	50325.23	51143.38	-34.23828

956.3	54242.9	54219.69	47387.27	47390.99	47462.01	50350.41	51178.09	23.21094
956.25	54212.1	54319.39	47349.38	47353.11	47424.45	50377.64	51212.34	-107.2852
956.2	54318.9	54420.54	47311.49	47315.24	47386.88	50406.97	51245.92	-101.6367
956.15	54493.8	54523.09	47273.63	47277.39	47349.34	50438.52	51278.73	-29.28906
956.1	54530.5	54627.17	47235.77	47239.55	47311.82	50472.45	51310.67	-96.66797
956.05	54411.5	54732.34	47197.94	47201.73	47274.31	50508.69	51341.42	-320.8359
956	54658.1	54838.72	47160.13	47163.94	47236.84	50547.45	51370.9	-180.6211
955.95	54985.5	54946.25	47122.35	47126.17	47199.39	50588.81	51398.93	39.24609
955.9	55145.6	55054.85	47084.58	47088.42	47161.96	50632.9	51425.31	90.75
955.85	54972.2	55164.65	47046.84	47050.7	47124.56	50679.95	51449.97	-192.4492
955.8	55128.3	55275.21	47009.13	47013	47087.19	50729.87	51472.55	-146.9141
955.75	55196.5	55386.67	46971.44	46975.33	47049.84	50782.89	51492.93	-190.168
955.7	54967	55498.97	46933.78	46937.69	47012.53	50839.17	51510.92	-531.9688
955.65	55055.6	55612.07	46896.15	46900.07	46975.25	50898.85	51526.36	-556.4688
955.6	55256.5	55726.12	46858.54	46862.48	46937.99	50962.2	51539.08	-469.6172
955.55	55525.1	55840.72	46820.97	46824.92	46900.77	51029.14	51548.8	-315.6211
955.5	55467.2	55956.07	46783.41	46787.39	46863.57	51099.97	51555.38	-488.8672
955.45	55540.4	56072.16	46745.9	46749.89	46826.41	51174.88	51558.68	-531.7617
955.4	55801.3	56189.03	46708.41	46712.42	46789.29	51254.05	51558.52	-387.7266
955.35	56285.2	56306.91	46670.96	46674.98	46752.2	51337.84	51554.77	-21.71094
955.3	56207.3	56425.51	46633.54	46637.58	46715.14	51426.17	51547.23	-218.207
955.25	56093.2	56545.11	46596.17	46600.23	46678.14	51519.43	51535.82	-451.9141

955.2	56131.3	56665.83	46558.83	46562.9	46641.17	51617.84	51520.4	-534.5313
955.15	56214.4	56787.83	46521.53	46525.62	46604.24	51721.67	51500.88	-573.4336
955.1	56548.4	56911.48	46484.26	46488.37	46567.35	51831.34	51477.2	-363.082
955.05	56822	57036.64	46447.04	46451.17	46530.51	51946.82	51449.27	-214.6445
955	57180.8	57163.73	46409.85	46414	46493.7	52068.54	51417.05	17.07422
954.95	57203.9	57293.08	46372.72	46376.89	46456.96	52196.86	51380.55	-89.17969
954.9	57327	57424.99	46335.63	46339.82	46420.25	52332.09	51339.73	-97.98828
954.85	57397.1	57560.07	46298.61	46302.81	46383.62	52474.83	51294.64	-162.9727
954.8	57663.4	57698.29	46261.61	46265.83	46347.01	52624.98	51245.29	-34.88672
954.75	57842.2	57840.34	46224.68	46228.92	46310.48	52783.21	51191.78	1.86328
954.7	57944.2	57986.68	46187.79	46192.05	46273.98	52949.88	51134.14	-42.47656
954.65	57893.3	58137.87	46150.96	46155.24	46237.55	53125.46	51072.51	-244.5703
954.6	58092	58294.72	46114.18	46118.47	46201.17	53310.7	51006.92	-202.7188
954.55	58239.9	58457.39	46077.46	46081.78	46164.86	53505.54	50937.61	-217.4922
954.5	58236.9	58626.75	46040.8	46045.13	46128.6	53710.77	50864.65	-389.8516
954.45	58649.5	58803.53	46004.19	46008.54	46092.4	53926.94	50788.22	-154.0273
954.4	59639.7	58988.45	45967.64	45972.02	46056.27	54154.63	50708.47	651.25
954.35	59497.9	59182.59	45931.17	45935.56	46020.21	54394.8	50625.53	315.3047
954.3	59393.8	59386.21	45894.76	45899.17	45984.22	54647.42	50539.68	7.58594
954.25	60102.7	59600.44	45858.41	45862.84	45948.3	54913.48	50451.05	502.2617
954.2	60521.1	59826.18	45822.15	45826.61	45912.46	55193.71	50359.86	694.918
954.15	60452.7	60064.35	45785.95	45790.43	45876.69	55488.83	50266.27	388.3516

954.1	60734.1	60316.28	45749.84	45754.33	45841	55800.05	50170.41	417.8242
954.05	61375	60582.24	45713.8	45718.31	45805.4	56127.32	50072.61	792.7578
954	61844	60863.63	45677.84	45682.37	45769.88	56471.89	49973	980.375
953.95	62095.9	61161.48	45641.97	45646.52	45734.45	56834.63	49871.78	934.4219
953.9	62684.8	61476.86	45606.19	45610.77	45699.11	57216.43	49769.12	1207.945
953.85	62590.4	61811.31	45570.5	45575.1	45663.88	57618.73	49665.12	779.0859
953.8	62823.8	62165.04	45534.91	45539.53	45628.73	58041.39	49560.13	658.7578
953.75	63077.9	62539.58	45499.42	45504.06	45593.7	58485.87	49454.22	538.3203
953.7	63171.9	62936.01	45464.02	45468.68	45558.75	58953.09	49347.56	235.8867
953.65	63421	63355.39	45428.72	45433.4	45523.91	59443.95	49240.3	65.60547
953.6	63927.7	63799.38	45393.53	45398.23	45489.18	59960.05	49132.51	128.3164
953.55	64736.5	64267.79	45358.43	45363.16	45454.55	60500.87	49024.5	468.7109
953.5	65417.3	64762.09	45323.45	45328.2	45420.04	61067.88	48916.33	655.2109
953.45	65652.3	65283.13	45288.59	45293.36	45385.65	61661.75	48808.13	369.1719
953.4	65981.9	65831.58	45253.84	45258.63	45351.38	62283.07	48700.01	150.3203
953.35	66788.3	66408.72	45219.21	45224.02	45317.23	62933.09	48592	379.5781
953.3	67321.3	67013.44	45184.72	45189.55	45283.23	63610.41	48484.41	307.8594
953.25	67369.5	67646.53	45150.36	45155.22	45249.36	64315.8	48377.23	-277.0313
953.2	67790.3	68307.77	45116.13	45121.01	45215.61	65048.98	48270.55	-517.4688
953.15	68385.7	68996.61	45082.03	45086.94	45182.01	65809.29	48164.47	-610.9063
953.1	69319.8	69713.02	45048.08	45053.01	45148.56	66596.7	48058.98	-393.2188
953.05	69655.1	70453.69	45014.29	45019.24	45115.27	67407.67	47954.36	-798.5859

953	69991.7	71217.45	44980.62	44985.59	45082.11	68241.07	47850.54	-1225.75
952.95	71008	72001.75	44947.11	44952.11	45049.11	69094.23	47747.63	-993.75
952.9	71833.3	72803.19	44913.77	44918.8	45016.29	69963.73	47645.7	-969.8906
952.85	72302.5	73618.73	44880.6	44885.64	44983.63	70846.55	47544.7	-1316.227
952.8	73034.2	74441.34	44847.6	44852.68	44951.15	71735.45	47444.87	-1407.141
952.75	73672.3	75266.27	44814.78	44819.88	44918.86	72625.71	47346.15	-1593.969
952.7	74426.3	76086.82	44782.14	44787.26	44886.74	73510.62	47248.61	-1660.523
952.65	75140.2	76895.48	44749.68	44754.82	44854.82	74382.59	47152.28	-1755.281
952.6	76180.9	77684.88	44717.42	44722.59	44823.1	75234.31	47057.13	-1503.977
952.55	77031.4	78443.89	44685.36	44690.56	44791.58	76054.49	46963.34	-1412.492
952.5	77720.4	79163.88	44653.52	44658.74	44760.28	76834.53	46870.88	-1443.477
952.45	78596	79834.73	44621.88	44627.13	44729.19	77564.3	46779.75	-1238.734
952.4	79490	80446.23	44590.46	44595.73	44698.33	78233.55	46690	-956.2344
952.35	80403	80988.92	44559.28	44564.58	44667.71	78832.88	46601.59	-585.9219
952.3	81039.3	81451.67	44528.32	44533.65	44637.32	79351.02	46514.66	-412.375
952.25	82097.2	81826.33	44497.61	44502.96	44607.18	79779.88	46429.16	270.875
952.2	82842	82105.13	44467.15	44472.53	44577.28	80111.66	46345.1	736.875
952.15	82767.7	82281.67	44436.94	44442.35	44547.65	80339.98	46262.51	486.0313
952.1	83281.9	82351.36	44406.98	44412.42	44518.28	80460.3	46181.32	930.5391
952.05	84018	82311.19	44377.27	44382.73	44489.15	80469.47	46101.64	1706.813
952	84058	82160.64	44347.84	44353.33	44460.31	80367.06	46023.46	1897.359
951.95	84154.1	81901.05	44318.66	44324.18	44431.73	80154.39	45946.74	2253.055

951.9	84126.3	81535.78	44289.76	44295.3	44403.42	79834.84	45871.5	2590.516
951.85	83708.7	81069.45	44261.11	44266.68	44375.38	79413.08	45797.65	2639.25
951.8	83425.2	80509.97	44232.72	44238.32	44347.6	78896.91	45725.31	2915.234
951.75	82429	79865.03	44204.6	44210.23	44320.09	78294.09	45654.42	2563.969
951.7	81357.7	79143.81	44176.72	44182.38	44292.83	77613.83	45584.94	2213.891
951.65	80152.8	78356.17	44149.09	44154.78	44265.83	76865.98	45516.86	1796.625
951.6	79554.1	77511.27	44121.7	44127.41	44239.06	76059.77	45450.12	2042.836
951.55	78442.5	76621.34	44094.54	44100.28	44212.53	75207.34	45384.79	1821.164
951.5	77150.4	75695.4	44067.59	44073.36	44186.22	74317.8	45320.78	1455
951.45	75662.2	74743	44040.86	44046.66	44160.14	73400.67	45258.1	919.2031
951.4	74475.3	73773	44014.33	44020.16	44134.26	72464.88	45196.69	702.2969
951.35	72960.1	72792.38	43988	43993.86	44108.58	71517.42	45136.5	167.7266
951.3	71492.7	71810.75	43961.85	43967.75	44083.1	70567.88	45077.59	-318.0469
951.25	70147.6	70833.39	43935.87	43941.8	44057.79	69621.56	45019.86	-685.7891
951.2	68574.7	69865.84	43910.05	43916.01	44032.64	68684.05	44963.3	-1291.141
951.15	67430.3	68912.78	43884.38	43890.37	44007.64	67760.05	44907.87	-1482.484
951.1	66127.7	67977.03	43858.85	43864.87	43982.8	66852.41	44853.51	-1849.328
951.05	64760.8	67063.98	43833.46	43839.51	43958.09	65966.5	44800.25	-2303.184
951	63698.1	66174.97	43808.18	43814.26	43933.5	65103.7	44748.04	-2476.867
950.95	63155.3	65311.91	43783.02	43789.13	43909.04	64265.95	44696.84	-2156.605
950.9	62143.5	64476.14	43757.96	43764.11	43884.7	63454.61	44646.62	-2332.641
950.85	61220.5	63667.63	43733.01	43739.19	43860.46	62669.69	44597.32	-2447.125

950.8	60526.1	62888.79	43708.15	43714.37	43836.32	61913.59	44548.97	-2362.688
950.75	59669.6	62138.84	43683.38	43689.63	43812.27	61185.56	44501.52	-2469.234
950.7	58840	61417.69	43658.7	43664.98	43788.32	60485.55	44454.95	-2577.691
950.65	58673.3	60725.01	43634.09	43640.41	43764.45	59813.23	44409.2	-2051.707
950.6	58289.2	60059.56	43609.57	43615.91	43740.66	59167.43	44364.25	-1770.359
950.55	57374.3	59422.25	43585.11	43591.49	43716.95	58549.02	44320.12	-2047.953
950.5	56797.2	58811.55	43560.71	43567.13	43693.31	57956.51	44276.74	-2014.348
950.45	56251.7	58226.63	43536.39	43542.84	43669.75	57389.1	44234.1	-1974.926
950.4	55456.3	57666.59	43512.13	43518.61	43646.25	56845.93	44192.17	-2210.289
950.35	54999.1	57129.91	43487.91	43494.43	43622.81	56325.49	44150.9	-2130.809
950.3	54618.4	56616.89	43463.74	43470.3	43599.42	55828.08	44110.3	-1998.488
950.25	54368.3	56125.94	43439.62	43446.21	43576.09	55352.15	44070.35	-1757.641
950.2	54045	55656.12	43415.54	43422.17	43552.8	54896.77	44031	-1611.121
950.15	53707.7	55206.49	43391.51	43398.18	43529.58	54461.02	43992.24	-1498.793
950.1	53545.7	54775.63	43367.51	43374.21	43506.39	54043.53	43954.03	-1229.93
950.05	53657.2	54363.66	43343.56	43350.3	43483.25	53644.39	43916.39	-706.457
950	52974.5	53969.19	43319.65	43326.43	43460.17	53262.26	43879.28	-994.6875
949.95	52541.2	53591.36	43295.77	43302.58	43437.12	52896.3	43842.66	-1050.164
949.9	52369.2	53229.39	43271.91	43278.77	43414.1	52545.72	43806.54	-860.1875
949.85	52156.8	52882.1	43248.1	43255	43391.14	52209.4	43770.87	-725.3008
949.8	51755	52549.53	43224.31	43231.25	43368.2	51887.36	43735.66	-794.5313
949.75	51957.9	52230.57	43200.55	43207.52	43345.3	51578.52	43700.88	-272.6719

949.7	51727.9	51924.54	43176.81	43183.82	43322.43	51282.21	43666.51	-196.6445
949.65	51705.4	51630.79	43153.1	43160.15	43299.59	50997.81	43632.54	74.60547
949.6	51750.7	51348.41	43129.42	43136.51	43276.8	50724.42	43598.94	402.2852
949.55	51238.2	51077.44	43105.76	43112.89	43254.03	50462.08	43565.71	160.7617
949.5	50685.6	50817	43082.12	43089.29	43231.29	50209.93	43532.84	-131.3945
949.45	50437.5	50566.55	43058.5	43065.71	43208.58	49967.46	43500.3	-129.0508
949.4	50248.6	50325.62	43034.9	43042.16	43185.9	49734.18	43468.08	-77.01563
949.35	49713.2	50093.46	43011.32	43018.63	43163.25	49509.41	43436.16	-380.2656
949.3	49452.9	49870.14	42987.75	42995.09	43140.6	49293.16	43404.53	-417.2422
949.25	50078.7	49654.98	42964.18	42971.57	43117.98	49084.81	43373.18	423.7188
949.2	50270.3	49447.58	42940.63	42948.06	43095.38	48883.95	43342.09	822.7227
949.15	50120.3	49247.56	42917.1	42924.57	43072.81	48690.22	43311.26	872.7383
949.1	49767.7	49054.36	42893.57	42901.09	43050.25	48503.07	43280.66	713.3398
949.05	49598	48868.05	42870.07	42877.63	43027.73	48322.59	43250.32	729.9453
949	49475.3	48688.12	42846.57	42854.18	43005.22	48148.24	43220.2	787.1836
948.95	49116.1	48514.23	42823.09	42830.73	42982.73	47979.73	43190.29	601.875
948.9	49093.5	48346.11	42799.61	42807.3	42960.25	47816.8	43160.58	747.3906
948.85	49408.1	48183.33	42776.14	42783.88	42937.8	47659	43131.07	1224.773
948.8	49480.6	48025.95	42752.67	42760.46	42915.36	47506.41	43101.74	1454.648
948.75	49164.7	47873.59	42729.23	42737.06	42892.95	47358.66	43072.6	1291.113
948.7	49223.3	47725.98	42705.78	42713.66	42870.55	47215.49	43043.63	1497.316
948.65	48956	47582.94	42682.35	42690.28	42848.17	47076.7	43014.83	1373.063

948.6	48779.3	47444.09	42658.92	42666.9	42825.81	46941.97	42986.18	1335.207
948.55	48982.6	47309.54	42635.5	42643.53	42803.46	46811.37	42957.69	1673.063
948.5	48829.9	47178.95	42612.09	42620.17	42781.14	46684.58	42929.34	1650.945
948.45	48396.6	47052.14	42588.68	42596.8	42758.82	46561.43	42901.13	1344.465
948.4	48322.6	46928.95	42565.27	42573.45	42736.52	46441.76	42873.05	1393.648
948.35	48233.1	46809.11	42541.88	42550.1	42714.25	46325.31	42845.09	1423.988
948.3	48360	46692.69	42518.48	42526.75	42691.97	46212.14	42817.25	1667.309
948.25	48532.3	46579.45	42495.09	42503.41	42669.72	46102.04	42789.54	1952.855
948.2	48521.4	46469.23	42471.69	42480.07	42647.48	45994.83	42761.93	2052.172
948.15	48070.7	46361.92	42448.3	42456.73	42625.25	45890.43	42734.43	1708.777
948.1	48067.6	46257.3	42424.92	42433.4	42603.04	45788.6	42707.03	1810.297
948.05	48303.7	46155.44	42401.53	42410.07	42580.84	45689.41	42679.72	2148.258
948	48354.2	46056.15	42378.15	42386.74	42558.66	45592.7	42652.51	2298.051
947.95	48133.6	45959.3	42354.78	42363.42	42536.49	45498.33	42625.4	2174.297
947.9	48039.1	45864.82	42331.4	42340.09	42514.33	45406.21	42598.37	2174.285
947.85	47916.9	45772.51	42308.03	42316.78	42492.2	45316.19	42571.43	2144.391
947.8	47429.2	45682.45	42284.65	42293.46	42470.07	45228.32	42544.56	1746.75
947.75	47469.2	45594.48	42261.28	42270.14	42447.95	45142.45	42517.77	1874.723
947.7	47514.6	45508.5	42237.9	42246.82	42425.85	45058.48	42491.05	2006.102
947.65	47454.4	45424.44	42214.52	42223.5	42403.75	44976.36	42464.41	2029.957
947.6	47110	45342.16	42191.14	42200.17	42381.66	44895.92	42437.81	1767.84
947.55	47075.3	45261.73	42167.75	42176.84	42359.59	44817.25	42411.29	1813.57

947.5	47054.2	45183.01	42144.36	42153.51	42337.52	44740.22	42384.83	1871.188
947.45	47126.6	45105.93	42120.96	42130.18	42315.46	44664.76	42358.43	2020.672
947.4	47188.2	45030.43	42097.56	42106.83	42293.41	44590.79	42332.07	2157.766
947.35	47124.7	44956.4	42074.15	42083.48	42271.37	44518.22	42305.77	2168.301
947.3	46648.9	44883.9	42050.74	42060.14	42249.34	44447.12	42279.53	1765
947.25	46620.6	44812.81	42027.32	42036.78	42227.32	44377.35	42253.33	1807.793
947.2	46826.3	44743.09	42003.9	42013.42	42205.31	44308.89	42227.18	2083.211
947.15	46461.4	44674.68	41980.47	41990.05	42183.3	44241.66	42201.07	1786.719
947.1	46501.8	44607.48	41957.03	41966.68	42161.3	44175.59	42175	1894.316
947.05	46416.6	44541.57	41933.59	41943.3	42139.31	44110.73	42148.98	1875.035
947	46317	44476.83	41910.13	41919.9	42117.32	44046.98	42122.99	1840.172
946.95	46951.7	44413.23	41886.66	41896.5	42095.35	43984.32	42097.05	2538.473
946.9	46719.3	44350.73	41863.19	41873.1	42073.38	43922.69	42071.13	2368.574
946.85	46389.5	44289.25	41839.71	41849.69	42051.42	43862.02	42045.25	2100.254
946.8	46160.7	44228.85	41816.23	41826.27	42029.47	43802.38	42019.41	1931.852
946.75	46287	44169.43	41792.73	41802.84	42007.53	43743.66	41993.61	2117.57
946.7	46142.9	44110.98	41769.23	41779.41	41985.59	43685.84	41967.83	2031.914
946.65	46040	44053.46	41745.71	41755.96	41963.66	43628.9	41942.08	1986.539
946.6	46121.9	43996.79	41722.19	41732.51	41941.74	43572.75	41916.35	2125.109
946.55	45872.3	43941.03	41698.65	41709.04	41919.83	43517.46	41890.66	1931.27
946.5	46074.5	43886.12	41675.11	41685.57	41897.92	43462.96	41864.99	2188.383
946.45	46335.8	43832.02	41651.55	41662.09	41876.03	43409.22	41839.34	2503.781

946.4	45915.8	43778.7	41627.98	41638.59	41854.13	43356.21	41813.72	2137.102
946.35	45727.2	43726.12	41604.41	41615.1	41832.26	43303.88	41788.13	2001.078
946.3	45751.8	43674.31	41580.82	41591.58	41810.38	43252.27	41762.54	2077.492
946.25	45716.5	43623.23	41557.23	41568.07	41788.52	43201.33	41737	2093.273
946.2	45682.9	43572.83	41533.61	41544.53	41766.66	43151.03	41711.46	2110.07
946.15	45525.9	43523.11	41509.99	41520.98	41744.81	43101.35	41685.94	2002.789
946.1	45287.8	43474.01	41486.36	41497.43	41722.97	43052.25	41660.44	1813.789
946.05	45657.9	43425.57	41462.7	41473.85	41701.13	43003.75	41634.95	2232.328
946	45732.1	43377.75	41439.04	41450.27	41679.3	42955.82	41609.48	2354.355
945.95	45637.8	43330.53	41415.36	41426.67	41657.48	42908.44	41584.03	2307.273
945.9	45615.6	43283.89	41391.68	41403.07	41635.67	42861.6	41558.59	2331.711
945.85	45512.7	43237.79	41367.98	41379.45	41613.87	42815.24	41533.17	2274.91
945.8	45500.9	43192.27	41344.27	41355.83	41592.08	42769.41	41507.76	2308.633
945.75	45708.3	43147.27	41320.55	41332.19	41570.3	42724.06	41482.36	2561.027
945.7	45655	43102.8	41296.81	41308.54	41548.54	42679.18	41456.98	2552.199
945.65	45096.4	43058.84	41273.07	41284.88	41526.78	42634.77	41431.62	2037.559
945.6	45299.1	43015.35	41249.32	41261.22	41505.04	42590.77	41406.27	2283.754
945.55	45041.4	42972.34	41225.54	41237.53	41483.3	42547.21	41380.91	2069.055
945.5	44906.9	42929.8	41201.75	41213.83	41461.57	42504.08	41355.57	1977.094
945.45	44897	42887.72	41177.95	41190.12	41439.86	42461.34	41330.25	2009.281
945.4	44859	42846.07	41154.13	41166.39	41418.15	42419	41304.92	2012.93
945.35	45080.1	42804.82	41130.29	41142.64	41396.45	42377	41279.6	2275.281

945.3	45385.8	42764.02	41106.45	41118.89	41374.77	42335.41	41254.29	2621.785
945.25	45328.8	42723.61	41082.58	41095.12	41353.09	42294.16	41228.98	2605.188
945.2	45082.1	42683.62	41058.71	41071.34	41331.44	42253.27	41203.69	2398.48
945.15	45021.9	42644.01	41034.82	41047.55	41309.79	42212.72	41178.4	2377.891
945.1	44989.8	42604.76	41010.92	41023.75	41288.17	42172.48	41153.12	2385.043
945.05	45013.2	42565.89	40987.01	40999.94	41266.55	42132.58	41127.84	2447.313
945	44695.6	42527.39	40963.08	40976.11	41244.95	42092.99	41102.58	2168.215
944.95	44672.3	42489.23	40939.13	40952.27	41223.36	42053.69	41077.3	2183.074
944.9	44805.2	42451.41	40915.18	40928.41	41201.79	42014.7	41052.04	2353.789
944.85	44327.1	42413.91	40891.2	40904.54	41180.23	41975.96	41026.78	1913.195
944.8	44161.5	42376.75	40867.2	40880.64	41158.68	41937.51	41001.52	1784.754
944.75	44141.1	42339.9	40843.2	40856.74	41137.15	41899.34	40976.25	1801.199
944.7	44115.8	42303.37	40819.16	40832.82	41115.63	41861.42	40950.99	1812.43
944.65	44228	42267.13	40795.11	40808.88	41094.12	41823.75	40925.72	1960.871
944.6	44104.6	42231.17	40771.04	40784.92	41072.63	41786.3	40900.45	1873.43
944.55	44145.8	42195.52	40746.96	40760.95	41051.16	41749.12	40875.18	1950.277
944.5	44310.7	42160.15	40722.85	40736.95	41029.69	41712.16	40849.91	2150.551
944.45	44173.1	42125.06	40698.73	40712.94	41008.24	41675.43	40824.63	2048.043
944.4	43694.8	42090.24	40674.59	40688.92	40986.81	41638.92	40799.36	1604.563
944.35	43831.7	42055.66	40650.43	40664.87	40965.39	41602.61	40774.07	1776.039
944.3	43990.3	42021.36	40626.25	40640.81	40944	41566.51	40748.78	1968.938
944.25	43871.7	41987.31	40602.04	40616.72	40922.62	41530.62	40723.48	1884.387

944.2	43402.3	41953.51	40577.82	40592.63	40901.25	41494.92	40698.19	1448.789
944.15	43503.2	41919.96	40553.58	40568.51	40879.91	41459.41	40672.88	1583.242
944.1	43407.3	41886.62	40529.31	40544.36	40858.57	41424.06	40647.56	1520.68
944.05	43333.3	41853.54	40505.03	40520.2	40837.26	41388.91	40622.24	1479.766
944	43145.8	41820.68	40480.72	40496.02	40815.97	41353.94	40596.91	1325.121
943.95	43186.7	41788.04	40456.39	40471.82	40794.69	41319.13	40571.56	1398.664
943.9	43475.3	41755.61	40432.02	40447.59	40773.42	41284.47	40546.2	1719.688
943.85	43094.9	41723.41	40407.65	40423.35	40752.2	41249.98	40520.85	1371.488
943.8	43003.4	41691.42	40383.25	40399.08	40730.97	41215.64	40495.47	1311.98
943.75	42939.3	41659.64	40358.83	40374.79	40709.78	41181.46	40470.09	1279.664
943.7	42968.9	41628.06	40334.38	40350.48	40688.6	41147.42	40444.69	1340.84
943.65	43049.4	41596.68	40309.91	40326.14	40667.45	41113.52	40419.29	1452.719
943.6	42939.6	41565.49	40285.41	40301.79	40646.31	41079.75	40393.86	1374.113
943.55	42182.4	41534.51	40260.89	40277.41	40625.2	41046.13	40368.43	647.8906
943.5	42504.5	41503.71	40236.34	40253.01	40604.11	41012.63	40342.98	1000.789
943.45	42122.8	41473.1	40211.76	40228.57	40583.04	40979.25	40317.51	649.7031
943.4	42908.2	41442.67	40187.16	40204.12	40562	40946	40292.03	1465.531
943.35	43166.8	41412.42	40162.53	40179.64	40540.99	40912.85	40266.53	1754.379
943.3	43066.3	41382.36	40137.88	40155.14	40520	40879.84	40241.02	1683.938
943.25	42796.5	41352.49	40113.21	40130.63	40499.04	40846.94	40215.5	1444.012
943.2	42768	41322.79	40088.52	40106.09	40478.12	40814.15	40189.98	1445.207
943.15	42312.9	41293.27	40063.8	40081.53	40457.22	40781.47	40164.43	1019.633

943.1	42217.6	41263.91	40039.06	40056.95	40436.37	40748.89	40138.88	953.6875
943.05	42387.9	41234.73	40014.28	40032.34	40415.53	40716.41	40113.29	1153.172
943	42120	41205.72	39989.49	40007.71	40394.73	40684.04	40087.7	914.2813
942.95	42205	41176.87	39964.66	39983.05	40373.96	40651.76	40062.09	1028.133
942.9	41945.4	41148.19	39939.81	39958.38	40353.23	40619.57	40036.46	797.2109
942.85	42057.3	41119.67	39914.94	39933.67	40332.54	40587.46	40010.81	937.6328
942.8	41811.2	41091.31	39890.04	39908.95	40311.88	40555.45	39985.15	719.8867
942.75	41860.2	41063.11	39865.1	39884.19	40291.25	40523.52	39959.46	797.0859
942.7	41700.7	41035.07	39840.14	39859.41	40270.66	40491.67	39933.76	665.6289
942.65	41616	41007.19	39815.15	39834.6	40250.11	40459.89	39908.04	608.8125
942.6	41768.5	40979.46	39790.13	39809.77	40229.61	40428.19	39882.29	789.043
942.55	41705.5	40951.88	39765.09	39784.91	40209.14	40396.57	39856.53	753.6172
942.5	41826.7	40924.46	39740.01	39760.02	40188.71	40365.02	39830.74	902.2422
942.45	41811	40897.2	39714.91	39735.12	40168.34	40333.55	39804.95	913.7969
942.4	41952.7	40870.09	39689.78	39710.18	40148	40302.14	39779.12	1082.605
942.35	41836.7	40843.14	39664.63	39685.23	40127.73	40270.8	39753.29	993.5547
942.3	41617.8	40816.34	39639.45	39660.24	40107.49	40239.52	39727.42	801.4648
942.25	41543.8	40789.7	39614.24	39635.24	40087.32	40208.31	39701.54	754.1055
942.2	41598.2	40763.21	39589.01	39610.22	40067.2	40177.16	39675.65	834.9922
942.15	41626.5	40736.87	39563.75	39585.17	40047.13	40146.08	39649.73	889.6328
942.1	41266.4	40710.68	39538.45	39560.09	40027.12	40115.04	39623.8	555.7188
942.05	41194.3	40684.65	39513.14	39534.99	40007.16	40084.07	39597.84	509.6484

942	41614.7	40658.77	39487.79	39509.86	39987.27	40053.15	39571.86	955.9258
941.95	41504.3	40633.04	39462.42	39484.71	39967.44	40022.29	39545.86	871.2578
941.9	41081.4	40607.48	39437.02	39459.54	39947.68	39991.48	39519.84	473.9219
941.85	41017.5	40582.05	39411.59	39434.34	39927.98	39960.7	39493.8	435.4492
941.8	40843.8	40556.79	39386.13	39409.12	39908.35	39929.99	39467.74	287.0078
941.75	40990.9	40531.68	39360.64	39383.86	39888.78	39899.31	39441.65	459.2188
941.7	40820.7	40506.73	39335.13	39358.59	39869.29	39868.69	39415.54	313.9727
941.65	40964.4	40481.92	39309.58	39333.29	39849.86	39838.1	39389.41	482.4766
941.6	40873.5	40457.29	39284	39307.96	39830.53	39807.55	39363.25	416.2148
941.55	40790.4	40432.8	39258.39	39282.61	39811.26	39777.05	39337.07	357.6016
941.5	40499.5	40408.47	39232.76	39257.22	39792.07	39746.58	39310.87	91.03125
941.45	40165.4	40384.3	39207.09	39231.82	39772.96	39716.15	39284.64	-218.8984
941.4	40244.5	40360.29	39181.39	39206.38	39753.94	39685.75	39258.38	-115.7891
941.35	39919.3	40336.44	39155.66	39180.92	39735.01	39655.38	39232.1	-417.1367
941.3	39934.5	40312.74	39129.89	39155.43	39716.16	39625.04	39205.79	-378.2422
941.25	40064.2	40289.21	39104.09	39129.9	39697.39	39594.73	39179.45	-225.0078
941.2	39918.6	40265.84	39078.25	39104.35	39678.72	39564.45	39153.08	-347.2422
941.15	39628.7	40242.64	39052.38	39078.77	39660.14	39534.2	39126.68	-613.9414
941.1	39511.8	40219.61	39026.48	39053.15	39641.67	39503.96	39100.26	-707.8086
941.05	39775.6	40196.73	39000.53	39027.5	39623.29	39473.74	39073.79	-421.1328
941	39460	40174.04	38974.56	39001.83	39605.02	39443.55	39047.31	-714.043
940.95	39408.1	40151.52	38948.55	38976.13	39586.85	39413.39	39020.8	-743.418

940.9	39213.2	40129.17	38922.5	38950.39	39568.79	39383.23	38994.25	-915.9727
940.85	39028.4	40107	38896.41	38924.63	39550.85	39353.1	38967.67	-1078.602
940.8	39167.9	40085.01	38870.29	38898.83	39533.02	39322.98	38941.05	-917.1094
940.75	39357.4	40063.19	38844.13	38872.99	39515.31	39292.87	38914.41	-705.793
940.7	39235.4	40041.57	38817.93	38847.13	39497.72	39262.78	38887.73	-806.168
940.65	38915.9	40020.13	38791.7	38821.23	39480.26	39232.71	38861.02	-1104.23
940.6	38766.6	39998.89	38765.43	38795.31	39462.95	39202.64	38834.28	-1232.285
940.55	38972.4	39977.84	38739.12	38769.36	39445.75	39172.59	38807.51	-1005.441
940.5	38957.2	39956.98	38712.77	38743.37	39428.7	39142.54	38780.7	-999.7852
940.45	38940.4	39936.34	38686.39	38717.35	39411.8	39112.51	38753.86	-995.9453
940.4	38899.1	39915.9	38659.97	38691.3	39395.04	39082.48	38726.99	-1016.801
940.35	39007.7	39895.68	38633.52	38665.22	39378.45	39052.46	38700.09	-887.9805
940.3	38908.1	39875.68	38607.02	38639.12	39362.02	39022.46	38673.15	-967.5742
940.25	38850.2	39855.89	38580.5	38612.98	39345.75	38992.46	38646.19	-1005.691
940.2	39001.3	39836.33	38553.94	38586.82	39329.65	38962.48	38619.2	-835.0273
940.15	39120.1	39817	38527.34	38560.63	39313.73	38932.5	38592.17	-696.9023
940.1	38862.1	39797.93	38500.71	38534.41	39298.01	38902.52	38565.11	-935.8242
940.05	38617.7	39779.08	38474.05	38508.17	39282.47	38872.55	38538.03	-1161.383
940	38421.5	39760.49	38447.35	38481.9	39267.13	38842.59	38510.91	-1338.988
939.95	38869.1	39742.14	38420.61	38455.61	39251.98	38812.63	38483.76	-873.043
939.9	38811.1	39724.06	38393.84	38429.28	39237.04	38782.68	38456.57	-912.957
939.85	38903.3	39706.27	38367.03	38402.93	39222.34	38752.73	38429.37	-802.9648

939.8	38724.8	39688.73	38340.2	38376.55	39207.85	38722.78	38402.13	-963.9258
939.75	38829	39671.47	38313.32	38350.16	39193.59	38692.84	38374.86	-842.4727
939.7	38832.5	39654.52	38286.42	38323.74	39179.57	38662.91	38347.56	-822.0156
939.65	38896.9	39637.85	38259.49	38297.29	39165.8	38632.98	38320.24	-740.9492
939.6	38830.9	39621.5	38232.52	38270.83	39152.3	38603.05	38292.88	-790.6055
939.55	39028.7	39605.46	38205.52	38244.34	39139.06	38573.13	38265.5	-576.7617
939.5	39213.4	39589.74	38178.5	38217.84	39126.09	38543.21	38238.1	-376.3438
939.45	38821.2	39574.36	38151.44	38191.32	39113.41	38513.29	38210.67	-753.1602
939.4	39005.7	39559.32	38124.36	38164.77	39101.02	38483.38	38183.21	-553.6211
939.35	39402.8	39544.65	38097.24	38138.21	39088.96	38453.48	38155.73	-141.8477
939.3	39362.6	39530.32	38070.1	38111.64	39077.19	38423.57	38128.22	-167.7188
939.25	39355.8	39516.38	38042.94	38085.05	39065.77	38393.69	38100.7	-160.582
939.2	38564.4	39502.82	38015.75	38058.45	39054.67	38363.8	38073.15	-938.4258
939.15	38789.9	39489.67	37988.54	38031.84	39043.93	38333.92	38045.58	-699.7695
939.1	39381.6	39476.93	37961.29	38005.21	39033.57	38304.04	38017.98	-95.32813
939.05	39516.7	39464.6	37934.02	37978.57	39023.56	38274.16	37990.36	52.10156
939	39282.8	39452.7	37906.72	37951.91	39013.94	38244.29	37962.72	-169.8984
938.95	39396.5	39441.26	37879.41	37925.26	39004.73	38214.43	37935.07	-44.75781
938.9	39275.4	39430.28	37852.07	37898.59	38995.94	38184.58	37907.39	-154.8789
938.85	38806.7	39419.79	37824.71	37871.91	38987.6	38154.73	37879.7	-613.0938
938.8	38919	39409.78	37797.33	37845.23	38979.68	38124.88	37851.98	-490.7813
938.75	39330.6	39400.28	37769.92	37818.54	38972.23	38095.04	37824.24	-69.67578

938.7	39420.4	39391.3	37742.49	37791.84	38965.25	38065.2	37796.48	29.09766
938.65	39226.6	39382.86	37715.04	37765.13	38958.77	38035.37	37768.71	-156.2578
938.6	39516.7	39375.01	37687.57	37738.43	38952.83	38005.54	37740.91	141.6914
938.55	39774.4	39367.71	37660.08	37711.72	38947.4	37975.72	37713.11	406.6836
938.5	39293.9	39361.03	37632.57	37685.02	38942.53	37945.92	37685.29	-67.12891
938.45	39300.4	39354.96	37605.05	37658.32	38938.23	37916.11	37657.45	-54.5625
938.4	39619.4	39349.54	37577.51	37631.61	38934.53	37886.32	37629.6	269.8633
938.35	39491.7	39344.8	37549.95	37604.91	38931.48	37856.53	37601.73	146.8984
938.3	39487.8	39340.74	37522.38	37578.22	38929.04	37826.75	37573.85	147.0625
938.25	39384.2	39337.38	37494.78	37551.52	38927.27	37796.97	37545.95	46.82031
938.2	39638.1	39334.77	37467.18	37524.84	38926.2	37767.21	37518.05	303.3281
938.15	39862.1	39332.93	37439.55	37498.16	38925.84	37737.45	37490.13	529.1719
938.1	39657.6	39331.93	37411.92	37471.5	38926.28	37707.7	37462.2	325.6719
938.05	39799.6	39331.72	37384.27	37444.84	38927.46	37677.96	37434.26	467.8789
938	39573.1	39332.39	37356.61	37418.2	38929.47	37648.24	37406.32	240.7148
937.95	39507.7	39333.95	37328.94	37391.57	38932.32	37618.52	37378.36	173.7539
937.9	39532.7	39336.45	37301.26	37364.96	38936.05	37588.81	37350.39	196.2539
937.85	39536.7	39339.96	37273.56	37338.36	38940.76	37559.11	37322.41	196.7422
937.8	39686.7	39344.45	37245.85	37311.78	38946.38	37529.42	37294.42	342.2539
937.75	39880.4	39349.99	37218.13	37285.21	38953.01	37499.73	37266.43	530.4102
937.7	39455.6	39356.65	37190.41	37258.68	38960.7	37470.07	37238.43	98.94922
937.65	39583.4	39364.47	37162.67	37232.16	38969.5	37440.41	37210.41	218.9258

937.6	39929.4	39373.55	37134.92	37205.66	38979.5	37410.75	37182.4	555.8516
937.55	39863.7	39383.85	37107.17	37179.19	38990.67	37381.12	37154.38	479.8516
937.5	40030.7	39395.48	37079.4	37152.75	39003.11	37351.49	37126.34	635.2188
937.45	40187.2	39408.52	37051.64	37126.34	39016.9	37321.88	37098.32	778.6758
937.4	40032.4	39423.02	37023.87	37099.96	39032.1	37292.28	37070.29	609.375
937.35	39908	39439.13	36996.09	37073.62	39048.84	37262.7	37042.25	468.8672
937.3	40208.8	39456.81	36968.32	37047.32	39067.09	37233.13	37014.22	751.9883
937.25	40445.8	39476.21	36940.54	37021.05	39087	37203.58	36986.18	969.5898
937.2	40273.8	39497.41	36912.75	36994.83	39108.65	37174.04	36958.15	776.3867
937.15	39824	39520.53	36884.97	36968.66	39132.15	37144.52	36930.12	303.4727
937.1	39902.8	39545.73	36857.19	36942.53	39157.66	37115.02	36902.09	357.0742
937.05	40183	39572.99	36829.4	36916.45	39185.17	37085.53	36874.05	610.0117
937	40693.5	39602.51	36801.61	36890.41	39214.86	37056.05	36846.02	1090.992
936.95	40697.7	39634.43	36773.83	36864.43	39246.89	37026.61	36818	1063.273
936.9	40866	39668.88	36746.05	36838.52	39281.38	36997.17	36789.98	1197.121
936.85	41382.8	39706.11	36718.28	36812.67	39318.56	36967.76	36761.96	1676.688
936.8	41046.6	39746.14	36690.52	36786.89	39358.46	36938.38	36733.97	1300.465
936.75	40830	39789.2	36662.77	36761.18	39401.32	36909.02	36705.98	1040.805
936.7	40913	39835.49	36635.03	36735.55	39447.32	36879.69	36678.01	1077.512
936.65	41113.8	39885.21	36607.29	36709.99	39496.68	36850.38	36650.04	1228.59
936.6	40769.6	39938.68	36579.57	36684.51	39549.69	36821.09	36622.09	830.918
936.55	41075.3	39995.95	36551.85	36659.12	39606.4	36791.84	36594.14	1079.355

936.5	41023.2	40057.34	36524.15	36633.81	39667.15	36762.61	36566.22	965.8555
936.45	41492.8	40123.13	36496.45	36608.6	39732.2	36733.39	36538.3	1369.672
936.4	41665.5	40193.6	36468.78	36583.49	39801.82	36704.22	36510.4	1471.898
936.35	41367.9	40269.17	36441.12	36558.48	39876.45	36675.07	36482.52	1098.727
936.3	41425.1	40349.91	36413.47	36533.59	39956.13	36645.95	36454.66	1075.191
936.25	41902	40436.27	36385.84	36508.8	40041.32	36616.86	36426.81	1465.734
936.2	41972.9	40528.59	36358.23	36484.13	40132.37	36587.81	36398.99	1444.305
936.15	41902.7	40627.27	36330.64	36459.59	40229.63	36558.79	36371.18	1275.434
936.1	41968.8	40732.84	36303.08	36435.2	40333.67	36529.8	36343.41	1235.961
936.05	41971.3	40845.39	36275.54	36410.93	40444.56	36500.86	36315.65	1125.91
936	42361.4	40965.5	36248.02	36386.81	40562.87	36471.94	36287.93	1395.898
935.95	42361	41093.61	36220.52	36362.85	40689.04	36443.07	36260.22	1267.387
935.9	42240.3	41230.2	36193.05	36339.05	40823.52	36414.23	36232.55	1010.102
935.85	42233.2	41375.93	36165.61	36315.42	40967	36385.44	36204.9	857.2695
935.8	42395	41530.88	36138.19	36291.96	41119.54	36356.68	36177.29	864.1172
935.75	42711.5	41695.79	36110.8	36268.69	41281.86	36327.96	36149.7	1015.715
935.7	42855.9	41871.16	36083.45	36245.62	41454.45	36299.29	36122.14	984.7422
935.65	43435.9	42057.51	36056.13	36222.75	41637.86	36270.66	36094.62	1378.387
935.6	43473.4	42255.71	36028.84	36200.11	41832.89	36242.08	36067.14	1217.691
935.55	43479.8	42465.73	36001.59	36177.7	42039.56	36213.55	36039.7	1014.07
935.5	43621.1	42688.42	35974.38	36155.53	42258.67	36185.06	36012.29	932.6797
935.45	43884.3	42924.35	35947.21	36133.63	42490.79	36156.64	35984.93	959.9531

935.4	43929.5	43174.07	35920.08	36111.99	42736.46	36128.26	35957.61	755.4258
935.35	44385	43438.54	35893	36090.65	42996.61	36099.94	35930.34	946.457
935.3	44554.2	43717.6	35865.96	36069.61	43271.08	36071.68	35903.12	836.6016
935.25	44480.1	44012.16	35838.98	36048.89	43560.78	36043.48	35875.95	467.9453
935.2	44498	44322.77	35812.04	36028.51	43866.21	36015.34	35848.82	175.2344
935.15	45198.5	44649.95	35785.15	36008.48	44187.91	35987.25	35821.75	548.5469
935.1	45584.3	44994.71	35758.32	35988.86	44526.84	35959.23	35794.74	589.5859
935.05	45992.1	45356.63	35731.54	35969.63	44882.56	35931.29	35767.78	635.4688
935	46216	45736.63	35704.83	35950.83	45255.98	35903.41	35740.89	479.375
934.95	46353.6	46135.12	35678.18	35932.5	45647.49	35875.61	35714.07	218.4805
934.9	46860.5	46552.5	35651.6	35914.65	46057.46	35847.88	35687.3	307.9961
934.85	47112.7	46989.72	35625.09	35897.34	46486.79	35820.24	35660.62	122.9805
934.8	47156.2	47445.93	35598.65	35880.57	46934.62	35792.68	35634.01	-289.7344
934.75	47482.8	47921.91	35572.28	35864.4	47401.69	35765.2	35607.47	-439.1094
934.7	48003.7	48417.83	35545.99	35848.86	47888.14	35737.8	35581.01	-414.1328
934.65	48634	48933.78	35519.78	35834	48394	35710.5	35554.63	-299.7813
934.6	48823.5	49470.45	35493.66	35819.9	48919.91	35683.29	35528.34	-646.9531
934.55	49449	50026.36	35467.62	35806.57	49464.34	35656.18	35502.13	-577.3555
934.5	50055.8	50601.95	35441.68	35794.12	50027.68	35629.17	35476.02	-546.1523
934.45	50314.8	51196.89	35415.84	35782.61	50609.5	35602.27	35450.01	-882.0898
934.4	50580.8	51810.66	35390.09	35772.16	51209.2	35575.47	35424.1	-1229.859
934.35	51123.2	52443.45	35364.45	35762.91	51826.79	35548.79	35398.29	-1320.246

934.3	51723.8	53092.82	35338.91	35754.99	52459.73	35522.22	35372.6	-1369.016
934.25	52546.3	53758.56	35313.49	35748.64	53107.59	35495.78	35347.02	-1212.258
934.2	53565.8	54439.52	35288.18	35744.14	53768.91	35469.46	35321.55	-873.7148
934.15	54078	55134.32	35263	35741.91	54441.94	35443.27	35296.21	-1056.32
934.1	54366.4	55842.36	35237.96	35742.5	55125.5	35417.23	35271.01	-1475.961
934.05	55150.4	56560.18	35213.04	35746.58	55815.46	35391.32	35245.94	-1409.777
934	56137.2	57286.9	35188.27	35755.19	56509.94	35365.57	35221.02	-1149.699
933.95	57108.5	58020.71	35163.65	35769.67	57205.78	35339.98	35196.23	-912.2148
933.9	58148.7	58759.92	35139.18	35791.84	57899.45	35314.54	35171.61	-611.2188
933.85	58580.9	59503.93	35114.87	35824.24	58587.87	35289.28	35147.15	-923.0273
933.8	59270.8	60249.72	35090.73	35869.95	59264.91	35264.19	35122.87	-978.918
933.75	60219.3	60997.67	35066.77	35933.24	59926.7	35239.29	35098.75	-778.3711
933.7	61185.2	61748.11	35042.98	36019.57	60568.11	35214.57	35074.82	-562.9102
933.65	62758.9	62502.53	35019.39	36135.88	61183.7	35190.05	35051.08	256.3711
933.6	63529.4	63264.92	34995.99	36291.15	61768.49	35165.73	35027.53	264.4766
933.55	64024.9	64038.33	34972.8	36495.75	62315.17	35141.63	35004.2	-13.43359
933.5	65520.2	64830.93	34949.83	36763.01	62818.58	35117.75	34981.08	689.2656
933.45	66403.3	65652.63	34927.07	37108.66	63272.91	35094.1	34958.18	750.6719
933.4	67597.3	66516.28	34904.55	37551.16	63672.58	35070.68	34935.51	1081.016
933.35	68822.1	67439.19	34882.26	38112.58	64012.8	35047.51	34913.09	1382.914
933.3	69628.8	68438.34	34860.22	38815.5	64288.01	35024.6	34890.91	1190.453
933.25	70721.6	69537.25	34838.44	39686.93	64494.72	35001.94	34868.98	1184.352

933.2	72197.6	70761.08	34816.92	40755.14	64629.8	34979.56	34847.32	1436.523
933.15	73667.6	72136.93	34795.68	42049.48	64691.06	34957.46	34825.94	1530.672
933.1	74724.1	73695.03	34774.71	43601.38	64677.3	34935.66	34804.84	1029.07
933.05	76227	75459.45	34754.05	45435	64588.41	34914.15	34784.05	767.5547
933	78517.6	77457.84	34733.7	47576.98	64425.43	34892.96	34763.55	1059.758
932.95	80645.6	79712.25	34713.66	50047.38	64190.38	34872.1	34743.39	933.3516
932.9	82849	82238.75	34693.97	52859.26	63886.25	34851.58	34723.56	610.25
932.85	85440	85048.86	34674.63	56020.82	63516.42	34831.43	34704.09	391.1406
932.8	88291.9	88133.44	34655.68	59517.48	63086.33	34811.66	34685	158.4609
932.75	90865.3	91481.32	34637.11	63333.34	62600.71	34792.3	34666.31	-616.0234
932.7	94209.4	95064.45	34618.97	67434.84	62065.13	34773.36	34648.04	-855.0547
932.65	97709.9	98840.21	34601.27	71773.55	61485.4	34754.87	34630.21	-1130.313
932.6	100848	102755.8	34584.05	76291.56	60866.69	34736.86	34612.86	-1907.828
932.55	104799	106729.4	34567.31	80899.62	60216.36	34719.34	34596	-1930.391
932.5	108767	110679.9	34551.1	85511.73	59539.48	34702.36	34579.66	-1912.922
932.45	112819	114510.2	34535.45	90025.14	58841.59	34685.94	34563.88	-1691.203
932.4	116825	118114.1	34520.37	94328.56	58127.89	34670.1	34548.68	-1289.125
932.35	120811	121383.9	34505.91	98310.3	57402.39	34654.89	34534.1	-572.9375
932.3	123760	124199	34492.11	101843.5	56671.32	34640.33	34520.16	-438.9688
932.25	126702	126455.1	34478.96	104820.8	55937.87	34626.44	34506.9	246.9063
932.2	128715	128054.2	34466.51	107140.5	55205.68	34613.25	34494.32	660.7656
932.15	130565	128915.3	34454.76	108718.4	54478	34600.77	34482.45	1649.664

932.1	131751	128980.6	34443.73	109494.7	53756.8	34589	34471.3	2770.375
932.05	131385	128221.7	34433.43	109436.8	53046.31	34577.98	34460.88	3163.281
932	129442	126643.2	34423.85	108548.1	52347.77	34567.69	34451.19	2798.828
931.95	126987	124281	34414.98	106862.7	51662.95	34558.11	34442.21	2706.031
931.9	122903	121199.9	34406.81	104443.8	50993.38	34549.23	34433.92	1703.125
931.85	118867	117482.8	34399.3	101373.9	50339.5	34541.02	34426.29	1384.219
931.8	113703	113243.1	34392.43	97763.8	49703.88	34533.45	34419.3	459.8516
931.75	108566	108590.8	34386.15	93723.36	49086.5	34526.48	34412.91	-24.78906
931.7	102560	103643.6	34380.42	89369.74	48487.95	34520.07	34407.07	-1083.563
931.65	96907.9	98517.81	34375.2	84818.88	47908.64	34514.17	34401.73	-1609.914
931.6	91431	93318.06	34370.46	80175.61	47348.21	34508.75	34396.88	-1887.063
931.55	85336	88158.84	34366.14	75552.91	46808.14	34503.76	34392.45	-2822.844
931.5	80352.4	83123.9	34362.2	71035.17	46287.78	34499.15	34388.39	-2771.5
931.45	76179.9	78289.73	34358.6	66698.88	45787.07	34494.89	34384.69	-2109.828
931.4	72286.6	73718.02	34355.32	62605.84	45305.89	34490.96	34381.3	-1431.422
931.35	68195.9	69450.37	34352.32	58798.38	44843.45	34487.31	34378.19	-1254.469
931.3	64915.7	65528.85	34349.58	55317.73	44400.61	34483.92	34375.34	-613.1523
931.25	62123.9	61965.8	34347.07	52177.05	43976.46	34480.76	34372.72	158.1016
931.2	59343.1	58766.35	34344.76	49381.88	43570.63	34477.82	34370.3	576.75
931.15	56839.6	55924.89	34342.64	46926.99	43182.68	34475.07	34368.08	914.7109
931.1	54506.9	53424.16	34340.69	44795.99	42811.73	34472.48	34366.02	1082.738
931.05	53026.4	51249.11	34338.89	42973.4	42458.2	34470.06	34364.12	1777.285

931	51678.2	49370.62	34337.23	41431.06	42121.11	34467.78	34362.35	2307.578
930.95	49964.9	47760	34335.7	40140.79	41799.95	34465.63	34360.71	2204.898
930.9	48359.4	46387.31	34334.28	39073.17	41494.18	34463.61	34359.2	1972.09
930.85	47047	45221.42	34332.98	38197.97	41202.89	34461.7	34357.79	1825.578
930.8	46030.9	44236.5	34331.76	37489.2	40926.24	34459.88	34356.48	1794.395
930.75	44686.3	43404.34	34330.64	36919.55	40663.3	34458.15	34355.25	1281.961
930.7	44054.5	42700.68	34329.59	36465.34	40413.5	34456.51	34354.1	1353.816
930.65	43746	42104.04	34328.61	36105.61	40176.29	34454.95	34353.03	1641.965
930.6	43106	41595.11	34327.71	35821.91	39950.84	34453.45	34352.02	1510.895
930.55	42270.5	41159.17	34326.87	35599.5	39737.16	34452.03	34351.08	1111.332
930.5	41457.3	40782.15	34326.09	35425.12	39534.42	34450.68	34350.21	675.1484
930.45	41064.4	40452.98	34325.36	35288.23	39342.08	34449.38	34349.38	611.418
930.4	40678.2	40162.63	34324.69	35180.33	39159.63	34448.13	34348.61	515.5664
930.35	40063.8	39903.55	34324.06	35094.55	38986.35	34446.93	34347.88	160.2539
930.3	40195.7	39670.57	34323.48	35025.82	38822.19	34445.79	34347.21	525.125
930.25	40004.3	39458.81	34322.93	34969.91	38666.44	34444.69	34346.57	545.4922
930.2	39445.6	39264.68	34322.44	34923.73	38518.66	34443.64	34345.98	180.918
930.15	38945.5	39085.39	34321.98	34884.88	38378.39	34442.63	34345.42	-139.8867
930.1	38464.6	38918.56	34321.55	34851.6	38245.06	34441.65	34344.9	-453.957
930.05	38069.6	38762.88	34321.16	34822.63	38118.58	34440.71	34344.42	-693.2773
930	38152.3	38616.8	34320.79	34796.99	37998.41	34439.81	34343.96	-464.4961
929.95	37915.4	38479.26	34320.45	34773.98	37884.17	34438.94	34343.53	-563.8594

929.9	37539.1	38349.37	34320.14	34753.07	37775.51	34438.09	34343.13	-810.2695
929.85	37374.3	38226.3	34319.86	34733.87	37671.98	34437.28	34342.75	-852.0039
929.8	37158.9	38109.76	34319.59	34716.14	37573.51	34436.49	34342.4	-950.8633
929.75	36835.5	37999.07	34319.35	34699.65	37479.68	34435.73	34342.07	-1163.574
929.7	36590	37893.82	34319.13	34684.25	37390.21	34434.99	34341.75	-1303.816
929.65	36483.4	37793.59	34318.92	34669.8	37304.83	34434.27	34341.46	-1310.191
929.6	36834.2	37697.94	34318.73	34656.2	37223.19	34433.56	34341.18	-863.7422
929.55	36532.9	37606.8	34318.56	34643.4	37145.28	34432.89	34340.92	-1073.902
929.5	36475	37519.76	34318.41	34631.32	37070.75	34432.23	34340.68	-1044.762
929.45	36423.4	37436.56	34318.27	34619.9	36999.42	34431.59	34340.46	-1013.16
929.4	36258.4	37356.96	34318.14	34609.09	36931.09	34430.96	34340.25	-1098.563
929.35	35909.1	37280.65	34318.04	34598.84	36865.51	34430.36	34340.05	-1371.551
929.3	35695.7	37207.61	34317.94	34589.13	36802.66	34429.77	34339.87	-1511.91
929.25	35929.1	37137.55	34317.86	34579.9	36742.32	34429.2	34339.7	-1208.453
929.2	35583.1	37070.31	34317.79	34571.14	36684.33	34428.64	34339.55	-1487.207
929.15	35671.6	37005.71	34317.72	34562.81	36628.56	34428.1	34339.4	-1334.109
929.1	35809.7	36943.53	34317.67	34554.88	36574.83	34427.57	34339.26	-1133.832
929.05	35570.4	36883.79	34317.63	34547.33	36523.14	34427.05	34339.14	-1313.387
929	35379.4	36826.26	34317.59	34540.14	36473.33	34426.54	34339.02	-1446.863
928.95	35250.1	36770.84	34317.57	34533.29	36425.3	34426.05	34338.91	-1520.742
928.9	35180.4	36717.41	34317.55	34526.74	36378.94	34425.56	34338.82	-1537.016
928.85	34915.9	36665.8	34317.54	34520.48	36334.12	34425.09	34338.72	-1749.906

928.8	34910.5	36616.05	34317.53	34514.52	36290.87	34424.62	34338.64	-1705.547
928.75	35188.5	36567.98	34317.53	34508.8	36249.05	34424.16	34338.55	-1379.484
928.7	35319	36521.53	34317.53	34503.34	36208.59	34423.71	34338.48	-1202.531
928.65	35249.7	36476.61	34317.54	34498.12	36169.44	34423.27	34338.41	-1226.91
928.6	35344	36433.1	34317.56	34493.11	36131.48	34422.84	34338.35	-1089.102
928.55	35162.5	36391.04	34317.59	34488.32	36094.76	34422.42	34338.3	-1228.535
928.5	34707.2	36350.3	34317.62	34483.73	36059.16	34422.01	34338.25	-1643.098
928.45	34719.2	36310.83	34317.66	34479.33	36024.65	34421.61	34338.21	-1591.633
928.4	34918.9	36272.57	34317.7	34475.11	35991.16	34421.21	34338.18	-1353.672
928.35	34787.5	36235.41	34317.74	34471.05	35958.63	34420.82	34338.14	-1447.914
928.3	34690.9	36199.42	34317.79	34467.16	35927.08	34420.43	34338.12	-1508.523
928.25	34896.8	36164.48	34317.84	34463.41	35896.44	34420.05	34338.09	-1267.684
928.2	34768	36130.56	34317.89	34459.82	35866.67	34419.68	34338.07	-1362.563
928.15	34517	36097.62	34317.95	34456.36	35837.74	34419.31	34338.05	-1580.617
928.1	34599.5	36065.57	34318.01	34453.03	35809.58	34418.95	34338.04	-1466.07
928.05	34517.3	36034.46	34318.07	34449.82	35782.23	34418.59	34338.02	-1517.16
928	34233.5	36004.21	34318.13	34446.73	35755.63	34418.24	34338.02	-1770.715
927.95	34557.4	35974.79	34318.2	34443.76	35729.73	34417.89	34338	-1417.395
927.9	34395	35946.18	34318.26	34440.89	35704.53	34417.54	34337.99	-1551.176
927.85	34456.8	35918.29	34318.32	34438.12	35679.96	34417.2	34337.99	-1461.492
927.8	34900.1	35891.19	34318.39	34435.45	35656.07	34416.86	34337.98	-991.0859
927.75	34506.3	35864.79	34318.46	34432.88	35632.79	34416.52	34337.98	-1358.492

927.7	34621.2	35839.09	34318.53	34430.39	35610.12	34416.2	34337.98	-1217.895
927.65	34633	35814.05	34318.61	34428	35588.02	34415.87	34337.98	-1181.055
927.6	34586	35789.64	34318.68	34425.68	35566.46	34415.55	34337.99	-1203.641
927.55	34345.5	35765.87	34318.76	34423.45	35545.47	34415.23	34338	-1420.371
927.5	34362.1	35742.7	34318.84	34421.29	35525	34414.92	34338.01	-1380.594
927.45	34546.7	35720.1	34318.92	34419.2	35505.03	34414.61	34338.02	-1173.402
927.4	34754.4	35698.07	34319	34417.18	35485.55	34414.3	34338.03	-943.668
927.35	34658.8	35676.55	34319.08	34415.22	35466.53	34414	34338.04	-1017.75
927.3	34247.9	35655.58	34319.17	34413.33	35447.98	34413.71	34338.06	-1407.684
927.25	34299.7	35635.13	34319.25	34411.51	35429.88	34413.41	34338.08	-1335.426
927.2	34541	35615.15	34319.34	34409.73	35412.21	34413.12	34338.1	-1074.152
927.15	33928.8	35595.65	34319.43	34408.02	35394.96	34412.84	34338.12	-1666.852
927.1	33758.7	35576.59	34319.51	34406.36	35378.09	34412.55	34338.14	-1817.895
927.05	34119.9	35557.99	34319.59	34404.74	35361.62	34412.26	34338.15	-1438.094
927	34214.3	35539.82	34319.67	34403.17	35345.53	34411.97	34338.17	-1325.523
926.95	34322.3	35522.07	34319.75	34401.65	35329.81	34411.68	34338.18	-1199.77
926.9	34242.4	35504.72	34319.83	34400.18	35314.45	34411.39	34338.19	-1262.324
926.85	34129.7	35487.74	34319.91	34398.74	35299.41	34411.11	34338.2	-1358.039
926.8	34226.1	35471.17	34319.98	34397.35	35284.73	34410.83	34338.22	-1245.066
926.75	34356	35454.95	34320.06	34395.99	35270.36	34410.54	34338.23	-1098.953
926.7	34123	35439.1	34320.13	34394.68	35256.32	34410.26	34338.24	-1316.102
926.65	34034.1	35423.6	34320.21	34393.4	35242.59	34409.98	34338.25	-1389.5

926.6	34161.9	35408.41	34320.28	34392.16	35229.13	34409.71	34338.26	-1246.516
926.55	33985.9	35393.57	34320.35	34390.95	35215.98	34409.43	34338.27	-1407.672
926.5	33837.4	35379.05	34320.42	34389.77	35203.12	34409.15	34338.28	-1541.648
926.45	33845.3	35364.83	34320.48	34388.62	35190.52	34408.87	34338.28	-1519.531
926.4	33850.4	35350.91	34320.55	34387.5	35178.18	34408.59	34338.28	-1500.508
926.35	34256.3	35337.26	34320.61	34386.4	35166.09	34408.3	34338.27	-1080.957
926.3	34381.9	35323.91	34320.66	34385.34	35154.27	34408.02	34338.27	-942.0156
926.25	34034.3	35310.84	34320.72	34384.3	35142.69	34407.75	34338.27	-1276.539
926.2	33955.1	35298.03	34320.78	34383.29	35131.34	34407.46	34338.26	-1342.93
926.15	33974.2	35285.48	34320.83	34382.3	35120.23	34407.18	34338.25	-1311.281
926.1	33979.3	35273.17	34320.88	34381.34	35109.33	34406.9	34338.25	-1293.871
926.05	34021.3	35261.13	34320.93	34380.4	35098.66	34406.62	34338.23	-1239.824
926	34228.3	35249.31	34320.97	34379.48	35088.2	34406.34	34338.22	-1021.012
925.95	34106.5	35237.73	34321.02	34378.58	35077.95	34406.06	34338.21	-1131.234
925.9	33829.3	35226.38	34321.06	34377.7	35067.9	34405.78	34338.19	-1397.082
925.85	34009.2	35215.23	34321.1	34376.84	35058.03	34405.49	34338.17	-1206.035
925.8	34141.2	35204.32	34321.14	34376	35048.37	34405.21	34338.15	-1063.117
925.75	33962.4	35193.61	34321.18	34375.18	35038.89	34404.93	34338.13	-1231.211
925.7	34166.5	35183.11	34321.21	34374.38	35029.6	34404.64	34338.11	-1016.605
925.65	34041.6	35172.8	34321.24	34373.6	35020.48	34404.36	34338.08	-1131.195
925.6	34175.7	35162.67	34321.27	34372.82	35011.52	34404.08	34338.05	-986.9688
925.55	34197.8	35152.74	34321.3	34372.07	35002.75	34403.8	34338.03	-954.9414

925.5	34232.9	35143	34321.33	34371.34	34994.13	34403.52	34338	-910.1016
925.45	34081.5	35133.44	34321.36	34370.62	34985.68	34403.23	34337.97	-1051.941
925.4	34136.6	35124.06	34321.38	34369.92	34977.39	34402.96	34337.94	-987.457
925.35	34019	35114.83	34321.41	34369.23	34969.23	34402.68	34337.91	-1095.828
925.3	34055.7	35105.77	34321.43	34368.55	34961.24	34402.39	34337.87	-1050.07
925.25	34211.7	35096.89	34321.45	34367.89	34953.39	34402.11	34337.84	-885.1875
925.2	33890.6	35088.16	34321.47	34367.24	34945.68	34401.84	34337.8	-1197.559
925.15	33581.3	35079.57	34321.48	34366.6	34938.11	34401.55	34337.77	-1498.273
925.1	33584.6	35071.14	34321.49	34365.97	34930.66	34401.26	34337.72	-1486.535
925.05	33939.7	35062.85	34321.5	34365.35	34923.34	34400.97	34337.67	-1123.148
925	34054.3	35054.7	34321.5	34364.74	34916.16	34400.68	34337.62	-1000.402
924.95	34321.5	35046.7	34321.5	34364.14	34909.1	34400.39	34337.57	-725.1953
1350	248418							
1349	247719							
1348	252963							
1347	250351							
1346	238660							
1345	240104							
1344	246843							
1343	239368							
1342	236587							
1341	231541							

1340	233431
1339	236123
1338	236696
1337	237862
1336	234050
1335	240649
1334	232214
1333	239233
1332	229644
1331	231311
1330	227543
1329	230857
1328	228767
1327	235319
1326	229143
1325	221541
1324	228101
1323	228238
1322	223378
1321	220248
1320	218233
1319	220223

1318	226837
1317	219188
1316	225054
1315	226384
1314	222643
1313	221959
1312	225398
1311	226178
1310	220777
1309	214798
1308	213997
1307	221064
1306	213111
1305	222079
1304	219685
1303	220568
1302	223749
1301	214257
1300	218222
1299	218289
1298	219119
1297	222835

1296	225272
1295	221743
1294	219972
1293	222289
1292	225762
1291	225930
1290	223578
1289	224225
1288	224126
1287	216994
1286	222859
1285	221181
1284	224565
1283	218734
1282	213400
1281	218994
1280	226122
1279	225336
1278	223014
1277	225827
1276	223019
1275	218260

1274	225193
1273	224427
1272	224928
1271	224165
1270	223570
1269	228311
1268	222277
1267	223467
1266	228897
1265	234460
1264	232409
1263	228319
1262	229963
1261	229328
1260	223244
1259	227844
1258	227484
1257	232504
1256	229096
1255	231201
1254	232664
1253	230449

1252	230593
1251	231291
1250	224092
1249	230648
1248	234734
1247	234233
1246	233139
1245	236568
1244	238868
1243	236601
1242	233698
1241	234895
1240	235303
1239	235857
1238	238285
1237	243696
1236	239816
1235	242714
1234	236147
1233	243110
1232	242926
1231	243200

1230	248385
1229	246917
1228	245557
1227	241391
1226	253920
1225	243177
1224	246663
1223	247277
1222	249927
1221	245462
1220	242241
1219	240949
1218	237895
1217	238239
1216	235139
1215	239016
1214	239418
1213	229676
1212	228098
1211	237919
1210	231848
1209	234206

1208	231247
1207	235937
1206	236611
1205	228481
1204	236368
1203	234452
1202	239022
1201	241393
1200	236600
1199	235800
1198	241806
1197	233423
1196	238086
1195	237272
1194	235671
1193	243221
1192	237805
1191	236772
1190	241167
1189	242714
1188	234294
1187	240762

1186	241641
1185	246765
1184	241752
1183	248628
1182	241555
1181	241628
1180	242413
1179	248521
1178	238713
1177	242956
1176	242319
1175	244052
1174	251629
1173	244813
1172	240453
1171	246823
1170	252722
1169	248004
1168	246368
1167	250090
1166	244174
1165	251021

1164	252526
1163	247744
1162	257608
1161	252941
1160	256107
1159	254545
1158	251413
1157	251115
1156	253835
1155	250027
1154	253603
1153	258722
1152	256795
1151	254601
1150	249606
1149	257725
1148	256082
1147	255554
1146	253709
1145	255916
1144	264068
1143	259216

1142	261088
1141	256496
1140	260575
1139	264687
1138	263074
1137	266459
1136	263965
1135	263590
1134	261906
1133	263201
1132	265911
1131	273059
1130	269452
1129	265478
1128	265360
1127	268138
1126	263792
1125	266141
1124	269370
1123	269528
1122	271297
1121	272515

1120	271028
1119	275323
1118	270808
1117	264033
1116	272546
1115	266148
1114	273876
1113	269607
1112	272513
1111	269288
1110	273085
1109	272057
1108	274166
1107	272690
1106	274315
1105	269751
1104	272817
1103	271949
1102	269271
1101	274717
1100	279733
1099	280885

1098	285757
1097	285780
1096	281530
1095	283509
1094	286114
1093	277500
1092	276963
1091	279257
1090	272175
1089	274951
1088	277902
1087	271585
1086	273500
1085	268354
1084	270751
1083	271318
1082	269679
1081	271645
1080	280619
1079	275370
1078	269302
1077	272951

1076	271846
1075	270932
1074	277017
1073	279600
1072	277797
1071	279756
1070	280594
1069	282408
1068	287575
1067	281808
1066	279788
1065	286125
1064	289337
1063	284085
1062	283604
1061	288448
1060	285810
1059	285144
1058	286563
1057	289988
1056	287100
1055	292739

1054	293106
1053	289036
1052	285750
1051	288903
1050	293417
1049	291894
1048	293909
1047	291280
1046	292616
1045	298407
1044	296158
1043	300895
1042	304153
1041	293300
1040	301754
1039	301778
1038	297542
1037	296595
1036	294284
1035	300277
1034	298701
1033	307532

1032	302216
1031	307042
1030	306546
1029	306311
1028	307995
1027	311354
1026	308642
1025	308658
1024	304546
1023	308853
1022	304611
1021	311681
1020	311434
1019	309538
1018	312366
1017	317919
1016	313403
1015	318591
1014	313207
1013	309707
1012	314585
1011	319933

1010	322472
1009	314110
1008	307600
1007	319641
1006	318130
1005	311712
1004	328779
1003	320572
1002	319230
1001	323662
1000	318825
999	323354
998	324898
997	325267
996	327833
995	328932
994	333629
993	320098
992	330327
991	327394
990	328976
989	330225

988 323204

987 332258

986 321087

985 330934

984 318292

983 328286

982 332539

981 336950

980 331079

979 340830

978 338809

977 340312

976 347231

975 346195

974 343791

973 337652

972 335163

971 337341

970 335131

969 322354

968 319911

967 315024

966	310909
965	306390
964	303511
963	306160
962	302059
961	295189
960	292979
959	292524
958	301129
957	306726
956	318767
955	329031
954	356169
953	401501
952	431441
951	355913
950	299332
949	290785
948	269457
947	269746
946	267780
945	262959

944 249463

943 245360

942 238285

941 233702

940 232201

939 226094

938 232816

937 241909

936 253464

935 282118

934 340430

933 492087

932 551018

931 321792

930 233865

929 206157

928 210161

927 205504

926 200121

925 199378

924 202655

923 203271

922 200342

921 201584

920 198982

919 199097

918 197628

917 202578

916 204341

915 207285

914 204629

913 201971

912 203255

911 203693

910 201952

909 204693

908 209059

907 204749

906 200449

905 198304

904 203953

903 207288

902 204789

901 203175

900 209635

899 205580

898 204568

897 210224

896 209194

895 208099

894 212974

893 210603

892 211295

891 207678

890 211926

889 217004

888 216127

887 215793

886 215051

885 216382

884 210735

883 217034

882 214350

881 213933

880 220470

879 213370

878 219598

877 216091

876 215736

875 218062

874 219070

873 225825

872 222425

871 225030

870 218600

869 221216

868 220426

867 223372

866 221669

865 222761

864 224970

863 221023

862 225199

861 227193

860 226297

859 231085

858 230367

857 228584

856 231189

855 226077

854 225499

853 230323

852 226202

851 229446

850 230887

849 233697

848 233681

847 231157

846 234938

845 224380

844 228804

843 236708

842 236352

841 240625

840 237051

839 237946

838 240274

837 240981

836 239311

835 240003

834 241087

833 234574

832 240896

831 237976

830 242990

829 244864

828 241525

827 241312

826 243977

825 244504

824 243677

823 239688

822 242062

821 254108

820 248991

819 246475

818 243851

817 246601

816 247927

815 247768

814 248904

813 249056

812 250776

811 254201

810 255293

809 252282

808 249332

807 253920

806 254277

805 253394

804 254968

803 258259

802 259634

801 252788

800 260241

799 256900

798 255711

797 256508

796 257972

795 261646

794 259201

793 261047

792 267034

791 260296

790 259644

789 262695

788 258740

787 261964

786 263054

785 264268

784 267063

783 266803

782 271258

781 272333

780 272818

779 270020

778 272256

777 267665

776 270985

775 270799

774 270305

773 269464

772 271988

771 273703

770 275489

769 276111

768 270323

767 273606

766 272811

765 276763

764 276407

763 267712

762 275712

761 274467

760 271764

759 280895

758 275023

757 276485

756 281460

755 278589

754 277507

753 275348

752 275423

751 275777

750 279002

749 276042

748 276467

747 274699

746 274698

745 278171

744 278509

743 285713

742 273400

741 272790

740 275254

739 273199

738 278132

737 283057

736 280737

735 277237

734 273726

733 273465

732 276233

731 284576

730 282187

729 278491

728 272787

727 276513

726 273594

725 279599

724	279800
723	283184
722	285885
721	284266
720	284546
719	280964
718	287691
717	278893
716	275656
715	276757
714	275556
713	277262
712	272922
711	274778
710	266900
709	263584
708	257495
707	249198
706	247942
705	248665
704	249158
703	245559

702 255204

701 249655

700 255590

699 254111

698 248028

697 248764

696 249425

695 251811

694 252160

693 252849

692 250764

691 250172

690 250645

689 253081

688 252458

687 242359

686 244968

685 243995

684 244602

683 237820

682 244244

681 240093

680 242862

679 248119

678 247005

677 236298

676 247415

675 248027

674 249351

673 241768

672 243890

671 251587

670 253038

669 244861

668 247621

667 249415

666 253437

665 246785

664 245677

663 242385

662 246288

661 249190

660 250624

659 251307

658 245399

657 247910

656 245586

655 245205

654 249801

653 245235

652 247408

651 262411

650 263887

649 272108

648 267306

647 256627

646 241880

645 236589

644 228879

643 229759

642 236434

641 239545

640 232266

639 226350

638 215680

637 204913

636 203209

635 201414

634 198808

633 201912

632 197456

631 200100

630 204762

629 200805

628 198836

627 196274

626 194498

625 191931

624 192268

623 191213

622 194740

621 186914

620 188266

619 189571

618 185580

617 182992

616 187163

615 186434

614	180955
613	186562
612	187122
611	186960
610	177849
609	186452
608	188155
607	184876
606	181379
605	183186
604	181259
603	187820
602	185145
601	186161
600	180777
599	189091
598	187567
597	189147
596	187081
595	192663
594	195516
593	196087

592 194014

591 199416

590 194536

589 191982

588 187008

587 186407

586 181608

585 182901

584 179555

583 173828

582 171113

581 174635

580 171693

579 169717

578 171410

577 177230

576 182169

575 187693

574 197380

573 202880

572 210000

571 217028

570	241423
569	284823
568	253882
567	199366
566	170498
565	144632
564	130643
563	119808
562	121297
561	112316
560	109091
559	109732
558	105587
557	105860
556	108369
555	110252
554	107272
553	106651
552	106245
551	108782
550	112228
549	115867

548	107534
547	96889.6
546	92905.3
545	80046.3
544	79005.3
543	76635.2
542	79085.3
541	74661
540	74655.9
539	68823.9
538	67561.5
537	71591.5
536	75641.4
535	76923.7
534	91138.8
533	129909
532	172305
531	158303
530	96824.3
529	72803
528	67780.9
527	67625.2

526 68249.4

525 63955.8

524 64283.7

523 61321.3

522 65680

521 66503.4

520 62167.3

519 59303

518 64131.6

517 62987.9

516 62569

515 63463.1

514 67516

513 63756.6

512 64588

511 63237.6

510 65226.7

509 68103.5

508 63190.1

507 62908.3

506 65161.5

505 62805.9

504 63299.6

503 65348.9

502 63314.5

501 63273.9

500 65762

499 67971.4

498 68990.1

497 65854.1

496 63414.9

495 62693.1

494 64589.8

493 63704.5

492 65349.2

491 62462.9

490 66662.5

489 65659.3

488 65934.4

487 63542.5

486 64557.6

485 65958.4

484 66656.7

483 67399.8

482	66358.6
481	65555.7
480	66053
479	63956.5
478	65958.9
477	67530.1
476	67169.2
475	67266.1
474	64537.6
473	64928.4
472	66187.5
471	66639.6
470	65178.9
469	66098.7
468	65136.1
467	66753.1
466	64988.8
465	63670.8
464	67204
463	67298.3
462	66455.4
461	67818.1

460 65359.9

459 68515

458 70046.2

457 71871.4

456 71234.3

455 69116.2

454 68882.9

453 67593.5

452 69277.8

451 71169.4

450 68887.5

449 70782.3

448 69549

447 69743.2

446 71911.5

445 69182.9

444 67502.2

443 71168.6

442 71340

441 67461.7

440 71561.7

439 73332.4

438	71342.7
437	68498.8
436	72367.6
435	72408.1
434	72721.7
433	71809.5
432	72109.1
431	70107.3
430	73123.2
429	71494.3
428	71077.9
427	73312.6
426	73417.2
425	74091.5
424	76095.6
423	72513.3
422	73023.2
421	72170.1
420	72367.9
419	72759.6
418	75858
417	73441.2

416 73386.6

415 74685.4

414 73612.2

413 72143

412 75556

411 73909.9

410 72540.7

409 73571.9

408 75755.3

407 74056.4

406 69073.5

405 71953.2

404 74758.1

403 75526.6

402 75560.4

401 84021

400 104976

399 96112.1

398 78823.1

397 73582.5

396 72536

395 70670.9

394	70074.6
393	72957.4
392	73450.3
391	77098.8
390	74331.2
389	73798.1
388	73614.8
387	75218.3
386	70671.6
385	72675.1
384	76710.5
383	75773.9
382	74634.5
381	75925.1
380	74402.6
379	73547.8
378	71243
377	74627.5
376	72646.3
375	74885.8
374	78259.4
373	76095.2

372	73703
371	74383.5
370	75640.8
369	76197.3
368	76010
367	78819.7
366	75612.9
365	78887.4
364	79842.1
363	74342.1
362	73685.6
361	75541.4
360	76380.2
359	78039.1
358	79471.5
357	77927.7
356	78118.3
355	78337.4
354	81438
353	79302.2
352	81830.8
351	86131.4

350	78583.5
349	79399.4
348	80922.8
347	80461.9
346	79884.9
345	81557.5
344	81128.5
343	82195.9
342	81530.9
341	81151.5
340	80344.1
339	81603.6
338	83465.5
337	81997.6
336	84477.8
335	81995.1
334	83730.1
333	83584.9
332	83026.5
331	84817.6
330	84848.7
329	85431.5

328 84137.6

327 82943.3

326 89012.2

325 87195.4

324 84591.8

323 85520.8

322 85920

321 84837.6

320 90140.3

319 84220.2

318 89821.9

317 91528

316 86250.7

315 90519.1

314 90352.3

313 88215.5

312 90516.1

311 88437.3

310 96306.9

309 93845.7

308 93172.6

307 90674.4

306	92206.7
305	91431.5
304	96010.1
303	89865
302	94023.8
301	90672.1
300	88184.9
299	88352.7
298	88242.5
297	86459.8
296	86797.1
295	81117.3
294	86522.4
293	85890.5
292	85516.9
291	84371.6
290	88551.2
289	100962
288	115199
287	129022
286	181877
285	225297

284 164539

283 102125

282 84147.8

281 81872.4

280 79630.2

279 82773

278 82582.7

277 77739

276 77013.3

275 80370.2

274 82019.1

273 80959

272 80777.3

271 80379.8

270 78942.8

269 78660.6

268 81995.3

267 81417.1

266 80334.9

265 82429.3

264 80600.8

263 82995

262	78467.1
261	81440.6
260	83099.4
259	85956.6
258	83518.4
257	82388.6
256	84274.1
255	83793.3
254	86656.8
253	84079.6
252	87648
251	82852
250	85861.2
249	88914.5
248	85933.7
247	90744.3
246	88672.9
245	87288.7
244	88638.3
243	82041.6
242	84927.9
241	82424.9

240	82727.9
239	80545.4
238	80458.7
237	79961.8
236	78920.6
235	79191.5
234	83431.6
233	85834.8
232	86054.2
231	85297.9
230	91078.5
229	97306.3
228	109081
227	123396
226	122214
225	101940
224	88485.4
223	81554.9
222	75930.8
221	72487.2
220	76663.8
219	71411.2

218	74186.2
217	74028.3
216	72448.5
215	73269.1
214	75119.4
213	74362.1
212	76002.9
211	74060.8
210	71437.3
209	69558.9
208	73125.3
207	72625
206	74914.4
205	75357
204	75013.9
203	74219.3
202	73447.4
201	75625
200	75486.6
199	76893
198	79394
197	75690

196	73112.7
195	76396
194	72898.2
193	74973.6
192	78426.3
191	74013.1
190	79668.1
189	82458.4
188	80039
187	83264.9
186	80611.4
185	80564.4
184	85208
183	79779.6
182	78875
181	79181.6
180	76640.7
179	78556.3
178	72915.4
177	74231.6
176	72612.1
175	70772.9

174 68966.4

173 68233.5

172 66024.7

171 65644.6

170 71189.1

169 73849.4

168 75069.5

167 73195.5

166 76018.4

165 85313.9

164 111669

163 141937

162 140948

161 98484.6

160 68318.6

159 59667.7

158 58820.1

157 57826.4

156 60011.4

155 58584.4

154 57302.9

153 58941.9

152 58793.7

151 58139.7

150 61532.4

149 58256.2

148 60607.9

147 60185.5

146 59461.9

145 62761.2

144 62590.1

143 62184.5

142 63242.8

141 61005.9

140 61290.1

139 60529.7

138 59596.1

137 59731

136 60236.5

135 58564

134 58660

133 56333.6

132 58667

131 58002.5

130 54270.5

129 55090.1

128 58398.1

127 57234.9

126 60846.9

125 65454.1

124 73106.9

123 84110.3

122 90860.6

121 76437.7

120 62233.2

119 57639.5

118 53190.7

117 56419.9

116 53340.7

115 48620.3

114 51682

113 53036.1

112 52928.3

111 51245.1

110 49205.8

109 50654.9

108	49623.5
107	53631.4
106	53477.6
105	49547.6
104	52754
103	54621.5
102	56763.2
101	55584.7
100	58199.9
99	60411.2
98	56799
97	60469.2
96	57944.8
95	57412.2
94	53729.3
93	51914
92	47321.1
91	47039.2
90	44230.4
89	40850.4
88	42051.2
87	39019.1

86	38340
85	35487.8
84	36452.6
83	38386.2
82	40402.1
81	43087.1
80	50314.5
79	61980.6
78	76154.7
77	96969.8
76	105359
75	111066
74	85849.9
73	52364.1
72	33834.5
71	26784.2
70	22062.1
69	18280.7
68	19606.4
67	18604.9
66	17634
65	17335.6

64	16677.6
63	16916.4
62	16123.9
61	17413.4
60	15417.8
59	15518.4
58	14243.6
57	17610.6
56	16603.3
55	16943.8
54	16683.7
53	15596.2
52	16256.4
51	15881.9
50	16103
49	17868.3
48	18365.3
47	18951.6
46	17529.3
45	14949.6
44	15914
43	15088.9

42	17030.9
41	16575.6
40	16363
39	15711.4
38	16448.4
37	15676
36	16026.5
35	16004.5
34	15737.6
33	15958.4
32	16152.3
31	17188.5
30	18244.4
29	19642.5
28	19611.2
27	20007.1
26	19466.3
25	21088
24	19259.4
23	18646.9
22	18317.1
21	16263.9

20	16070.5
19	14759.1
18	16466.3
17	15683.4
16	14153.5
15	14127.8
14	13377.7
13	13886.1
12	12316.4
11	10391.9
10	9003.49
9	9102.57
8	10851.2
7	13052.8
6	18200.5
5	25290.7
4	35908.1
3	46157.5
2	34363.1
1	17686.4
0	6228.22
-1	1740.93

-2	1214.2
-3	461.466
-4	200.225
-5	90.2345
-6	106.835
-7	57.4004
-8	54.1656
-9	69.559
-10	263.535

X-ray diffraction (XRD) patterns

5	494.63964	5	1237
5.01	516.63964	5.01	1215
5.02	517.63964	5.02	1224
5.03	520.63964	5.03	1232
5.04	522.63964	5.04	1195
5.05	532.63964	5.05	1206
5.06	554.63964	5.06	1231
5.07	571.63964	5.07	1202
5.08	534.63964	5.08	1198
5.09	510.63964	5.09	1257
5.1	520.63964	5.1	1206
5.11	502.63964	5.11	1174

5.12	497.63964	5.12	1184
5.13	516.63964	5.13	1210
5.14	515.63964	5.14	1194
5.15	549.63964	5.15	1204
5.16	519.63964	5.16	1228
5.17	525.63964	5.17	1199
5.18	546.63964	5.18	1229
5.19	508.63964	5.19	1204
5.2	504.63964	5.2	1208
5.21	502.63964	5.21	1175
5.22	485.63964	5.22	1238
5.23	526.63964	5.23	1179
5.24	464.63964	5.24	1169
5.25	466.63964	5.25	1198
5.26	461.63964	5.26	1178
5.27	499.63964	5.27	1176
5.28	474.63964	5.28	1224
5.29	493.63964	5.29	1227
5.3	499.63964	5.3	1206
5.31	464.63964	5.31	1202
5.32	482.63964	5.32	1197
5.33	504.63964	5.33	1192

5.34	497.63964	5.34	1184
5.35	510.63964	5.35	1160
5.36	492.63964	5.36	1181
5.37	469.63964	5.37	1205
5.38	495.63964	5.38	1187
5.39	549.63964	5.39	1180
5.4	468.63964	5.4	1148
5.41	441.63964	5.41	1169
5.42	479.63964	5.42	1207
5.43	497.63964	5.43	1169
5.44	491.63964	5.44	1174
5.45	487.63964	5.45	1178
5.46	463.63964	5.46	1171
5.47	496.63964	5.47	1186
5.48	468.63964	5.48	1193
5.49	480.63964	5.49	1158
5.5	466.63964	5.5	1189
5.51	481.63964	5.51	1162
5.52	470.63964	5.52	1185
5.53	531.63964	5.53	1187
5.54	484.63964	5.54	1184
5.55	460.63964	5.55	1181

5.56	490.63964	5.56	1177
5.57	506.63964	5.57	1179
5.58	511.63964	5.58	1169
5.59	481.63964	5.59	1174
5.6	490.63964	5.6	1200
5.61	491.63964	5.61	1213
5.62	494.63964	5.62	1160
5.63	500.63964	5.63	1176
5.64	494.63964	5.64	1231
5.65	512.63964	5.65	1205
5.66	471.63964	5.66	1149
5.67	499.63964	5.67	1187
5.68	501.63964	5.68	1178
5.69	495.63964	5.69	1208
5.7	487.63964	5.7	1146
5.71	520.63964	5.71	1216
5.72	463.63964	5.72	1247
5.73	457.63964	5.73	1150
5.74	477.63964	5.74	1168
5.75	506.63964	5.75	1190
5.76	478.63964	5.76	1178
5.77	473.63964	5.77	1216

5.78	499.63964	5.78	1197
5.79	515.63964	5.79	1207
5.8	504.63964	5.8	1188
5.81	471.63964	5.81	1187
5.82	471.63964	5.82	1179
5.83	507.63964	5.83	1149
5.84	543.63964	5.84	1244
5.85	514.63964	5.85	1182
5.86	511.63964	5.86	1181
5.87	456.63964	5.87	1214
5.88	479.63964	5.88	1176
5.89	499.63964	5.89	1117
5.9	513.63964	5.9	1153
5.91	491.63964	5.91	1172
5.92	488.63964	5.92	1200
5.93	495.63964	5.93	1186
5.94	485.63964	5.94	1226
5.95	463.63964	5.95	1155
5.96	490.63964	5.96	1195
5.97	487.63964	5.97	1179
5.98	478.63964	5.98	1207
5.99	490.63964	5.99	1182

6	503.63964	6	1163
6.01	475.63964	6.01	1176
6.02	480.63964	6.02	1212
6.03	494.63964	6.03	1185
6.04	506.63964	6.04	1146
6.05	523.63964	6.05	1215
6.06	490.63964	6.06	1173
6.07	517.63964	6.07	1171
6.08	533.63964	6.08	1173
6.09	493.63964	6.09	1171
6.1	464.63964	6.1	1177
6.11	471.63964	6.11	1136
6.12	433.63964	6.12	1172
6.13	457.63964	6.13	1162
6.14	436.63964	6.14	1184
6.15	477.63964	6.15	1166
6.16	489.63964	6.16	1164
6.17	491.63964	6.17	1188
6.18	500.63964	6.18	1174
6.19	517.63964	6.19	1175
6.2	544.63964	6.2	1180
6.21	501.63964	6.21	1187

6.22	538.63964	6.22	1186
6.23	512.63964	6.23	1197
6.24	471.63964	6.24	1178
6.25	481.63964	6.25	1218
6.26	474.63964	6.26	1168
6.27	496.63964	6.27	1150
6.28	456.63964	6.28	1186
6.29	511.63964	6.29	1225
6.3	463.63964	6.3	1174
6.31	504.63964	6.31	1154
6.32	511.63964	6.32	1177
6.33	505.63964	6.33	1229
6.34	459.63964	6.34	1156
6.35	481.63964	6.35	1129
6.36	467.63964	6.36	1159
6.37	496.63964	6.37	1153
6.38	471.63964	6.38	1176
6.39	450.63964	6.39	1198
6.4	513.63964	6.4	1212
6.41	490.63964	6.41	1206
6.42	462.63964	6.42	1192
6.43	502.63964	6.43	1179

6.44	519.63964	6.44	1161
6.45	510.63964	6.45	1168
6.46	483.63964	6.46	1184
6.47	487.63964	6.47	1214
6.48	469.63964	6.48	1195
6.49	477.63964	6.49	1136
6.5	471.63964	6.5	1207
6.51	470.63964	6.51	1174
6.52	451.63964	6.52	1190
6.53	486.63964	6.53	1172
6.54	495.63964	6.54	1173
6.55	479.63964	6.55	1182
6.56	481.63964	6.56	1192
6.57	502.63964	6.57	1187
6.58	485.63964	6.58	1173
6.59	497.63964	6.59	1152
6.6	520.63964	6.6	1198
6.61	483.63964	6.61	1158
6.62	487.63964	6.62	1153
6.63	508.63964	6.63	1190
6.64	533.63964	6.64	1166
6.65	487.63964	6.65	1168

6.66	505.63964	6.66	1151
6.67	518.63964	6.67	1139
6.68	505.63964	6.68	1171
6.69	494.63964	6.69	1153
6.7	464.63964	6.7	1162
6.71	484.63964	6.71	1186
6.72	459.63964	6.72	1127
6.73	468.63964	6.73	1171
6.74	481.63964	6.74	1154
6.75	500.63964	6.75	1182
6.76	471.63964	6.76	1168
6.77	525.63964	6.77	1167
6.78	443.63964	6.78	1159
6.79	501.63964	6.79	1151
6.8	471.63964	6.8	1161
6.81	513.63964	6.81	1188
6.82	451.63964	6.82	1194
6.83	488.63964	6.83	1168
6.84	458.63964	6.84	1178
6.85	425.63964	6.85	1155
6.86	424.63964	6.86	1173
6.87	486.63964	6.87	1138

6.88	452.63964	6.88	1176
6.89	487.63964	6.89	1186
6.9	484.63964	6.9	1190
6.91	464.63964	6.91	1181
6.92	492.63964	6.92	1174
6.93	489.63964	6.93	1175
6.94	495.63964	6.94	1137
6.95	475.63964	6.95	1189
6.96	453.63964	6.96	1170
6.97	473.63964	6.97	1152
6.98	464.63964	6.98	1145
6.99	516.63964	6.99	1171
7	495.63964	7	1210
7.01	501.63964	7.01	1179
7.02	474.63964	7.02	1177
7.03	463.63964	7.03	1177
7.04	475.63964	7.04	1107
7.05	485.63964	7.05	1144
7.06	496.63964	7.06	1167
7.07	471.63964	7.07	1152
7.08	493.63964	7.08	1182
7.09	481.63964	7.09	1180

7.1	459.63964	7.1	1155
7.11	493.63964	7.11	1181
7.12	441.63964	7.12	1173
7.13	488.63964	7.13	1180
7.14	437.63964	7.14	1171
7.15	473.63964	7.15	1159
7.16	467.63964	7.16	1188
7.17	475.63964	7.17	1157
7.18	519.63964	7.18	1156
7.19	415.63964	7.19	1158
7.2	467.63964	7.2	1172
7.21	466.63964	7.21	1158
7.22	490.63964	7.22	1212
7.23	461.63964	7.23	1207
7.24	490.63964	7.24	1170
7.25	491.63964	7.25	1200
7.26	466.63964	7.26	1183
7.27	474.63964	7.27	1158
7.28	481.63964	7.28	1162
7.29	463.63964	7.29	1164
7.3	450.63964	7.3	1179
7.31	485.63964	7.31	1194

7.32	486.63964	7.32	1196
7.33	486.63964	7.33	1151
7.34	507.63964	7.34	1131
7.35	462.63964	7.35	1166
7.36	474.63964	7.36	1176
7.37	493.63964	7.37	1164
7.38	480.63964	7.38	1188
7.39	445.63964	7.39	1166
7.4	465.63964	7.4	1189
7.41	457.63964	7.41	1145
7.42	465.63964	7.42	1150
7.43	489.63964	7.43	1195
7.44	473.63964	7.44	1200
7.45	491.63964	7.45	1199
7.46	491.63964	7.46	1202
7.47	473.63964	7.47	1145
7.48	448.63964	7.48	1202
7.49	471.63964	7.49	1155
7.5	495.63964	7.5	1194
7.51	476.63964	7.51	1181
7.52	458.63964	7.52	1188
7.53	487.63964	7.53	1210

7.54	485.63964	7.54	1184
7.55	510.63964	7.55	1203
7.56	469.63964	7.56	1176
7.57	489.63964	7.57	1195
7.58	494.63964	7.58	1144
7.59	532.63964	7.59	1160
7.6	461.63964	7.6	1173
7.61	490.63964	7.61	1148
7.62	494.63964	7.62	1143
7.63	447.63964	7.63	1164
7.64	463.63964	7.64	1172
7.65	458.63964	7.65	1145
7.66	470.63964	7.66	1155
7.67	448.63964	7.67	1155
7.68	479.63964	7.68	1176
7.69	494.63964	7.69	1170
7.7	478.63964	7.7	1153
7.71	477.63964	7.71	1163
7.72	489.63964	7.72	1143
7.73	451.63964	7.73	1156
7.74	445.63964	7.74	1173
7.75	463.63964	7.75	1158

7.76	515.63964	7.76	1147
7.77	514.63964	7.77	1183
7.78	459.63964	7.78	1124
7.79	518.63964	7.79	1171
7.8	454.63964	7.8	1137
7.81	459.63964	7.81	1176
7.82	485.63964	7.82	1167
7.83	477.63964	7.83	1142
7.84	456.63964	7.84	1162
7.85	450.63964	7.85	1175
7.86	448.63964	7.86	1151
7.87	510.63964	7.87	1133
7.88	454.63964	7.88	1162
7.89	497.63964	7.89	1147
7.9	484.63964	7.9	1150
7.91	468.63964	7.91	1135
7.92	444.63964	7.92	1127
7.93	457.63964	7.93	1185
7.94	430.63964	7.94	1131
7.95	450.63964	7.95	1193
7.96	501.63964	7.96	1179
7.97	473.63964	7.97	1186

7.98	499.63964	7.98	1176
7.99	427.63964	7.99	1168
8	453.63964	8	1157
8.01	448.63964	8.01	1149
8.02	469.63964	8.02	1176
8.03	484.63964	8.03	1199
8.04	486.63964	8.04	1194
8.05	487.63964	8.05	1137
8.06	455.63964	8.06	1159
8.07	433.63964	8.07	1160
8.08	473.63964	8.08	1176
8.09	426.63964	8.09	1187
8.1	443.63964	8.1	1152
8.11	495.63964	8.11	1182
8.12	486.63964	8.12	1106
8.13	489.63964	8.13	1149
8.14	460.63964	8.14	1209
8.15	480.63964	8.15	1164
8.16	445.63964	8.16	1150
8.17	441.63964	8.17	1162
8.18	461.63964	8.18	1187
8.19	456.63964	8.19	1159

8.2	470.63964	8.2	1194
8.21	441.63964	8.21	1149
8.22	462.63964	8.22	1153
8.23	456.63964	8.23	1141
8.24	461.63964	8.24	1139
8.25	463.63964	8.25	1167
8.26	464.63964	8.26	1134
8.27	493.63964	8.27	1131
8.28	473.63964	8.28	1145
8.29	445.63964	8.29	1134
8.3	438.63964	8.3	1152
8.31	497.63964	8.31	1162
8.32	460.63964	8.32	1177
8.33	475.63964	8.33	1142
8.34	475.63964	8.34	1155
8.35	466.63964	8.35	1147
8.36	467.63964	8.36	1099
8.37	505.63964	8.37	1119
8.38	476.63964	8.38	1157
8.39	450.63964	8.39	1153
8.4	465.63964	8.4	1157
8.41	463.63964	8.41	1125

8.42	481.63964	8.42	1126
8.43	466.63964	8.43	1186
8.44	473.63964	8.44	1159
8.45	434.63964	8.45	1139
8.46	473.63964	8.46	1132
8.47	489.63964	8.47	1142
8.48	465.63964	8.48	1142
8.49	458.63964	8.49	1151
8.5	452.63964	8.5	1172
8.51	465.63964	8.51	1134
8.52	507.63964	8.52	1168
8.53	505.63964	8.53	1162
8.54	452.63964	8.54	1145
8.55	429.63964	8.55	1154
8.56	466.63964	8.56	1142
8.57	484.63964	8.57	1111
8.58	467.63964	8.58	1174
8.59	475.63964	8.59	1149
8.6	476.63964	8.6	1142
8.61	449.63964	8.61	1144
8.62	440.63964	8.62	1146
8.63	478.63964	8.63	1130

8.64	439.63964	8.64	1135
8.65	476.63964	8.65	1167
8.66	449.63964	8.66	1170
8.67	478.63964	8.67	1171
8.68	449.63964	8.68	1120
8.69	474.63964	8.69	1163
8.7	490.63964	8.7	1168
8.71	490.63964	8.71	1155
8.72	468.63964	8.72	1141
8.73	437.63964	8.73	1151
8.74	499.63964	8.74	1143
8.75	477.63964	8.75	1186
8.76	485.63964	8.76	1180
8.77	425.63964	8.77	1123
8.78	468.63964	8.78	1119
8.79	508.63964	8.79	1129
8.8	469.63964	8.8	1135
8.81	442.63964	8.81	1119
8.82	453.63964	8.82	1127
8.83	450.63964	8.83	1137
8.84	460.63964	8.84	1136
8.85	484.63964	8.85	1166

8.86	456.63964	8.86	1153
8.87	448.63964	8.87	1154
8.88	423.63964	8.88	1145
8.89	455.63964	8.89	1185
8.9	470.63964	8.9	1146
8.91	474.63964	8.91	1116
8.92	468.63964	8.92	1138
8.93	455.63964	8.93	1148
8.94	494.63964	8.94	1136
8.95	453.63964	8.95	1125
8.96	510.63964	8.96	1152
8.97	482.63964	8.97	1138
8.98	482.63964	8.98	1160
8.99	464.63964	8.99	1166
9	462.63964	9	1158
9.01	514.63964	9.01	1139
9.02	479.63964	9.02	1194
9.03	464.63964	9.03	1143
9.04	470.63964	9.04	1136
9.05	421.63964	9.05	1129
9.06	456.63964	9.06	1151
9.07	439.63964	9.07	1141

9.08	470.63964	9.08	1136
9.09	453.63964	9.09	1160
9.1	438.63964	9.1	1115
9.11	470.63964	9.11	1168
9.12	438.63964	9.12	1150
9.13	417.63964	9.13	1147
9.14	436.63964	9.14	1154
9.15	461.63964	9.15	1119
9.16	460.63964	9.16	1148
9.17	450.63964	9.17	1112
9.18	460.63964	9.18	1137
9.19	434.63964	9.19	1120
9.2	482.63964	9.2	1106
9.21	443.63964	9.21	1139
9.22	455.63964	9.22	1156
9.23	468.63964	9.23	1142
9.24	471.63964	9.24	1138
9.25	428.63964	9.25	1133
9.26	449.63964	9.26	1150
9.27	469.63964	9.27	1160
9.28	460.63964	9.28	1107
9.29	473.63964	9.29	1108

9.3	442.63964	9.3	1100
9.31	467.63964	9.31	1127
9.32	416.63964	9.32	1142
9.33	462.63964	9.33	1146
9.34	474.63964	9.34	1129
9.35	479.63964	9.35	1112
9.36	471.63964	9.36	1182
9.37	468.63964	9.37	1151
9.38	431.63964	9.38	1150
9.39	417.63964	9.39	1125
9.4	441.63964	9.4	1139
9.41	463.63964	9.41	1123
9.42	456.63964	9.42	1139
9.43	433.63964	9.43	1172
9.44	438.63964	9.44	1163
9.45	520.63964	9.45	1147
9.46	495.63964	9.46	1103
9.47	471.63964	9.47	1111
9.48	419.63964	9.48	1122
9.49	449.63964	9.49	1148
9.5	460.63964	9.5	1121
9.51	441.63964	9.51	1158

9.52	467.63964	9.52	1153
9.53	436.63964	9.53	1125
9.54	467.63964	9.54	1153
9.55	456.63964	9.55	1148
9.56	422.63964	9.56	1138
9.57	444.63964	9.57	1166
9.58	444.63964	9.58	1112
9.59	449.63964	9.59	1149
9.6	422.63964	9.6	1145
9.61	440.63964	9.61	1161
9.62	488.63964	9.62	1143
9.63	441.63964	9.63	1152
9.64	437.63964	9.64	1110
9.65	438.63964	9.65	1141
9.66	439.63964	9.66	1121
9.67	456.63964	9.67	1085
9.68	453.63964	9.68	1127
9.69	463.63964	9.69	1120
9.7	441.63964	9.7	1146
9.71	444.63964	9.71	1130
9.72	436.63964	9.72	1116
9.73	460.63964	9.73	1151

9.74	429.63964	9.74	1137
9.75	428.63964	9.75	1185
9.76	463.63964	9.76	1121
9.77	417.63964	9.77	1148
9.78	439.63964	9.78	1133
9.79	426.63964	9.79	1131
9.8	464.63964	9.8	1136
9.81	452.63964	9.81	1122
9.82	439.63964	9.82	1163
9.83	490.63964	9.83	1110
9.84	447.63964	9.84	1139
9.85	455.63964	9.85	1144
9.86	458.63964	9.86	1163
9.87	440.63964	9.87	1118
9.88	461.63964	9.88	1159
9.89	451.63964	9.89	1106
9.9	432.63964	9.9	1100
9.91	450.63964	9.91	1119
9.92	465.63964	9.92	1161
9.93	458.63964	9.93	1115
9.94	457.63964	9.94	1146
9.95	432.63964	9.95	1107

9.96	458.63964	9.96	1138
9.97	484.63964	9.97	1132
9.98	469.63964	9.98	1118
9.99	495.63964	9.99	1124
10	481.63964	10	1153
10.01	443.63964	10.01	1108
10.02	451.63964	10.02	1113
10.03	421.63964	10.03	1113
10.04	461.63964	10.04	1127
10.05	459.63964	10.05	1120
10.06	479.63964	10.06	1109
10.07	454.63964	10.07	1127
10.08	478.63964	10.08	1148
10.09	474.63964	10.09	1153
10.1	418.63964	10.1	1139
10.11	427.63964	10.11	1095
10.12	449.63964	10.12	1142
10.13	423.63964	10.13	1131
10.14	445.63964	10.14	1083
10.15	453.63964	10.15	1112
10.16	465.63964	10.16	1145
10.17	428.63964	10.17	1118

10.18	477.63964	10.18	1118
10.19	444.63964	10.19	1137
10.2	466.63964	10.2	1123
10.21	456.63964	10.21	1125
10.22	486.63964	10.22	1130
10.23	464.63964	10.23	1153
10.24	444.63964	10.24	1134
10.25	455.63964	10.25	1123
10.26	471.63964	10.26	1149
10.27	458.63964	10.27	1106
10.28	455.63964	10.28	1108
10.29	434.63964	10.29	1108
10.3	449.63964	10.3	1129
10.31	437.63964	10.31	1131
10.32	429.63964	10.32	1126
10.33	495.63964	10.33	1125
10.34	443.63964	10.34	1153
10.35	477.63964	10.35	1104
10.36	417.63964	10.36	1147
10.37	460.63964	10.37	1096
10.38	456.63964	10.38	1121
10.39	469.63964	10.39	1136

10.4	477.63964	10.4	1109
10.41	461.63964	10.41	1131
10.42	443.63964	10.42	1109
10.43	485.63964	10.43	1132
10.44	485.63964	10.44	1127
10.45	478.63964	10.45	1135
10.46	457.63964	10.46	1134
10.47	489.63964	10.47	1127
10.48	433.63964	10.48	1110
10.49	428.63964	10.49	1126
10.5	455.63964	10.5	1116
10.51	459.63964	10.51	1115
10.52	435.63964	10.52	1099
10.53	457.63964	10.53	1100
10.54	452.63964	10.54	1095
10.55	456.63964	10.55	1142
10.56	432.63964	10.56	1127
10.57	502.63964	10.57	1112
10.58	441.63964	10.58	1128
10.59	447.63964	10.59	1136
10.6	471.63964	10.6	1127
10.61	451.63964	10.61	1110

10.62	490.63964	10.62	1128
10.63	443.63964	10.63	1156
10.64	463.63964	10.64	1119
10.65	482.63964	10.65	1094
10.66	433.63964	10.66	1111
10.67	452.63964	10.67	1105
10.68	475.63964	10.68	1087
10.69	449.63964	10.69	1122
10.7	471.63964	10.7	1147
10.71	439.63964	10.71	1116
10.72	463.63964	10.72	1131
10.73	415.63964	10.73	1115
10.74	443.63964	10.74	1153
10.75	514.63964	10.75	1117
10.76	464.63964	10.76	1151
10.77	460.63964	10.77	1104
10.78	464.63964	10.78	1082
10.79	490.63964	10.79	1109
10.8	442.63964	10.8	1105
10.81	474.63964	10.81	1152
10.82	463.63964	10.82	1120
10.83	451.63964	10.83	1128

10.84	475.63964	10.84	1109
10.85	441.63964	10.85	1179
10.86	435.63964	10.86	1102
10.87	473.63964	10.87	1099
10.88	450.63964	10.88	1130
10.89	469.63964	10.89	1111
10.9	467.63964	10.9	1105
10.91	456.63964	10.91	1091
10.92	491.63964	10.92	1128
10.93	470.63964	10.93	1127
10.94	463.63964	10.94	1112
10.95	477.63964	10.95	1137
10.96	448.63964	10.96	1156
10.97	460.63964	10.97	1099
10.98	443.63964	10.98	1127
10.99	494.63964	10.99	1121
11	484.63964	11	1131
11.01	464.63964	11.01	1155
11.02	458.63964	11.02	1129
11.03	434.63964	11.03	1126
11.04	439.63964	11.04	1152
11.05	442.63964	11.05	1112

11.06	457.63964	11.06	1092
11.07	445.63964	11.07	1148
11.08	478.63964	11.08	1120
11.09	441.63964	11.09	1135
11.1	416.63964	11.1	1147
11.11	452.63964	11.11	1138
11.12	414.63964	11.12	1077
11.13	441.63964	11.13	1085
11.14	421.63964	11.14	1134
11.15	426.63964	11.15	1110
11.16	434.63964	11.16	1116
11.17	434.63964	11.17	1134
11.18	462.63964	11.18	1128
11.19	463.63964	11.19	1096
11.2	459.63964	11.2	1105
11.21	443.63964	11.21	1100
11.22	474.63964	11.22	1126
11.23	432.63964	11.23	1120
11.24	442.63964	11.24	1078
11.25	491.63964	11.25	1127
11.26	475.63964	11.26	1108
11.27	448.63964	11.27	1120

11.28	427.63964	11.28	1130
11.29	469.63964	11.29	1102
11.3	430.63964	11.3	1108
11.31	475.63964	11.31	1072
11.32	445.63964	11.32	1102
11.33	468.63964	11.33	1120
11.34	439.63964	11.34	1112
11.35	444.63964	11.35	1078
11.36	439.63964	11.36	1087
11.37	457.63964	11.37	1108
11.38	444.63964	11.38	1156
11.39	454.63964	11.39	1126
11.4	440.63964	11.4	1100
11.41	452.63964	11.41	1131
11.42	440.63964	11.42	1078
11.43	452.63964	11.43	1121
11.44	445.63964	11.44	1130
11.45	461.63964	11.45	1117
11.46	459.63964	11.46	1121
11.47	468.63964	11.47	1143
11.48	464.63964	11.48	1079
11.49	421.63964	11.49	1094

11.5	447.63964	11.5	1084
11.51	456.63964	11.51	1116
11.52	470.63964	11.52	1120
11.53	491.63964	11.53	1079
11.54	479.63964	11.54	1110
11.55	480.63964	11.55	1130
11.56	485.63964	11.56	1136
11.57	426.63964	11.57	1098
11.58	437.63964	11.58	1098
11.59	477.63964	11.59	1097
11.6	496.63964	11.6	1085
11.61	469.63964	11.61	1117
11.62	444.63964	11.62	1103
11.63	430.63964	11.63	1134
11.64	464.63964	11.64	1105
11.65	435.63964	11.65	1100
11.66	447.63964	11.66	1111
11.67	443.63964	11.67	1084
11.68	462.63964	11.68	1091
11.69	479.63964	11.69	1108
11.7	455.63964	11.7	1126
11.71	462.63964	11.71	1108

11.72	432.63964	11.72	1149
11.73	476.63964	11.73	1122
11.74	438.63964	11.74	1092
11.75	434.63964	11.75	1124
11.76	433.63964	11.76	1118
11.77	440.63964	11.77	1124
11.78	425.63964	11.78	1093
11.79	442.63964	11.79	1091
11.8	441.63964	11.8	1108
11.81	501.63964	11.81	1105
11.82	452.63964	11.82	1095
11.83	447.63964	11.83	1092
11.84	441.63964	11.84	1080
11.85	427.63964	11.85	1102
11.86	461.63964	11.86	1113
11.87	463.63964	11.87	1070
11.88	507.63964	11.88	1111
11.89	465.63964	11.89	1105
11.9	455.63964	11.9	1128
11.91	414.63964	11.91	1097
11.92	433.63964	11.92	1061
11.93	443.63964	11.93	1123

11.94	470.63964	11.94	1106
11.95	481.63964	11.95	1099
11.96	452.63964	11.96	1104
11.97	452.63964	11.97	1103
11.98	461.63964	11.98	1065
11.99	507.63964	11.99	1084
12	442.63964	12	1103
12.01	461.63964	12.01	1110
12.02	424.63964	12.02	1111
12.03	483.63964	12.03	1136
12.04	451.63964	12.04	1107
12.05	492.63964	12.05	1104
12.06	444.63964	12.06	1087
12.07	436.63964	12.07	1067
12.08	398.63964	12.08	1092
12.09	463.63964	12.09	1101
12.1	431.63964	12.1	1124
12.11	466.63964	12.11	1141
12.12	511.63964	12.12	1089
12.13	467.63964	12.13	1124
12.14	476.63964	12.14	1116
12.15	465.63964	12.15	1080

12.16	441.63964	12.16	1121
12.17	421.63964	12.17	1091
12.18	423.63964	12.18	1110
12.19	428.63964	12.19	1134
12.2	466.63964	12.2	1079
12.21	426.63964	12.21	1107
12.22	425.63964	12.22	1100
12.23	480.63964	12.23	1115
12.24	454.63964	12.24	1095
12.25	439.63964	12.25	1086
12.26	457.63964	12.26	1104
12.27	478.63964	12.27	1146
12.28	448.63964	12.28	1083
12.29	466.63964	12.29	1084
12.3	435.63964	12.3	1109
12.31	467.63964	12.31	1121
12.32	442.63964	12.32	1107
12.33	476.63964	12.33	1064
12.34	468.63964	12.34	1089
12.35	440.63964	12.35	1095
12.36	437.63964	12.36	1101
12.37	430.63964	12.37	1103

12.38	447.63964	12.38	1090
12.39	485.63964	12.39	1098
12.4	464.63964	12.4	1113
12.41	467.63964	12.41	1104
12.42	447.63964	12.42	1101
12.43	463.63964	12.43	1108
12.44	458.63964	12.44	1086
12.45	464.63964	12.45	1098
12.46	469.63964	12.46	1099
12.47	432.63964	12.47	1078
12.48	434.63964	12.48	1077
12.49	462.63964	12.49	1090
12.5	468.63964	12.5	1086
12.51	421.63964	12.51	1106
12.52	436.63964	12.52	1128
12.53	435.63964	12.53	1119
12.54	428.63964	12.54	1098
12.55	435.63964	12.55	1073
12.56	442.63964	12.56	1085
12.57	451.63964	12.57	1086
12.58	474.63964	12.58	1116
12.59	465.63964	12.59	1080

12.6	456.63964	12.6	1129
12.61	413.63964	12.61	1109
12.62	442.63964	12.62	1091
12.63	440.63964	12.63	1086
12.64	405.63964	12.64	1118
12.65	459.63964	12.65	1083
12.66	484.63964	12.66	1077
12.67	419.63964	12.67	1083
12.68	463.63964	12.68	1070
12.69	447.63964	12.69	1085
12.7	453.63964	12.7	1113
12.71	438.63964	12.71	1148
12.72	424.63964	12.72	1084
12.73	439.63964	12.73	1093
12.74	459.63964	12.74	1107
12.75	442.63964	12.75	1098
12.76	460.63964	12.76	1102
12.77	458.63964	12.77	1104
12.78	437.63964	12.78	1105
12.79	442.63964	12.79	1104
12.8	455.63964	12.8	1104
12.81	462.63964	12.81	1090

12.82	443.63964	12.82	1063
12.83	463.63964	12.83	1087
12.84	496.63964	12.84	1073
12.85	457.63964	12.85	1081
12.86	419.63964	12.86	1093
12.87	430.63964	12.87	1118
12.88	473.63964	12.88	1084
12.89	434.63964	12.89	1108
12.9	423.63964	12.9	1111
12.91	481.63964	12.91	1106
12.92	434.63964	12.92	1089
12.93	469.63964	12.93	1095
12.94	423.63964	12.94	1110
12.95	462.63964	12.95	1049
12.96	423.63964	12.96	1077
12.97	482.63964	12.97	1065
12.98	471.63964	12.98	1093
12.99	411.63964	12.99	1069
13	435.63964	13	1087
13.01	458.63964	13.01	1078
13.02	432.63964	13.02	1096
13.03	486.63964	13.03	1131

13.04	452.63964	13.04	1139
13.05	428.63964	13.05	1078
13.06	470.63964	13.06	1084
13.07	432.63964	13.07	1101
13.08	418.63964	13.08	1132
13.09	442.63964	13.09	1105
13.1	454.63964	13.1	1102
13.11	463.63964	13.11	1091
13.12	468.63964	13.12	1110
13.13	469.63964	13.13	1086
13.14	427.63964	13.14	1093
13.15	410.63964	13.15	1132
13.16	470.63964	13.16	1104
13.17	457.63964	13.17	1069
13.18	421.63964	13.18	1084
13.19	434.63964	13.19	1127
13.2	469.63964	13.2	1090
13.21	449.63964	13.21	1102
13.22	485.63964	13.22	1106
13.23	486.63964	13.23	1110
13.24	477.63964	13.24	1072
13.25	444.63964	13.25	1109

13.26	430.63964	13.26	1127
13.27	426.63964	13.27	1119
13.28	440.63964	13.28	1060
13.29	449.63964	13.29	1082
13.3	463.63964	13.3	1097
13.31	443.63964	13.31	1093
13.32	442.63964	13.32	1079
13.33	423.63964	13.33	1068
13.34	407.63964	13.34	1086
13.35	505.63964	13.35	1108
13.36	478.63964	13.36	1127
13.37	422.63964	13.37	1113
13.38	474.63964	13.38	1100
13.39	495.63964	13.39	1054
13.4	468.63964	13.4	1108
13.41	458.63964	13.41	1119
13.42	455.63964	13.42	1079
13.43	443.63964	13.43	1075
13.44	503.63964	13.44	1101
13.45	436.63964	13.45	1049
13.46	463.63964	13.46	1079
13.47	445.63964	13.47	1061

13.48	465.63964	13.48	1073
13.49	428.63964	13.49	1090
13.5	448.63964	13.5	1090
13.51	432.63964	13.51	1086
13.52	433.63964	13.52	1093
13.53	414.63964	13.53	1089
13.54	437.63964	13.54	1122
13.55	451.63964	13.55	1128
13.56	407.63964	13.56	1084
13.57	454.63964	13.57	1094
13.58	451.63964	13.58	1074
13.59	448.63964	13.59	1087
13.6	451.63964	13.6	1073
13.61	438.63964	13.61	1085
13.62	431.63964	13.62	1086
13.63	460.63964	13.63	1105
13.64	482.63964	13.64	1087
13.65	435.63964	13.65	1101
13.66	458.63964	13.66	1066
13.67	461.63964	13.67	1092
13.68	451.63964	13.68	1105
13.69	457.63964	13.69	1102

13.7	416.63964	13.7	1099
13.71	454.63964	13.71	1107
13.72	443.63964	13.72	1085
13.73	406.63964	13.73	1101
13.74	474.63964	13.74	1129
13.75	457.63964	13.75	1081
13.76	441.63964	13.76	1078
13.77	451.63964	13.77	1094
13.78	433.63964	13.78	1110
13.79	428.63964	13.79	1122
13.8	447.63964	13.8	1102
13.81	431.63964	13.81	1101
13.82	437.63964	13.82	1084
13.83	448.63964	13.83	1074
13.84	441.63964	13.84	1126
13.85	450.63964	13.85	1083
13.86	460.63964	13.86	1117
13.87	464.63964	13.87	1091
13.88	497.63964	13.88	1081
13.89	473.63964	13.89	1071
13.9	474.63964	13.9	1045
13.91	465.63964	13.91	1086

13.92	460.63964	13.92	1078
13.93	427.63964	13.93	1097
13.94	463.63964	13.94	1073
13.95	451.63964	13.95	1068
13.96	488.63964	13.96	1087
13.97	457.63964	13.97	1110
13.98	410.63964	13.98	1082
13.99	429.63964	13.99	1059
14	423.63964	14	1084
14.01	460.63964	14.01	1119
14.02	479.63964	14.02	1093
14.03	477.63964	14.03	1083
14.04	445.63964	14.04	1049
14.05	432.63964	14.05	1098
14.06	432.63964	14.06	1074
14.07	484.63964	14.07	1134
14.08	468.63964	14.08	1108
14.09	403.63964	14.09	1099
14.1	421.63964	14.1	1130
14.11	492.63964	14.11	1098
14.12	441.63964	14.12	1116
14.13	444.63964	14.13	1117

14.14	461.63964	14.14	1094
14.15	452.63964	14.15	1097
14.16	442.63964	14.16	1098
14.17	424.63964	14.17	1081
14.18	425.63964	14.18	1104
14.19	465.63964	14.19	1096
14.2	430.63964	14.2	1085
14.21	434.63964	14.21	1123
14.22	475.63964	14.22	1098
14.23	430.63964	14.23	1075
14.24	424.63964	14.24	1106
14.25	471.63964	14.25	1091
14.26	481.63964	14.26	1099
14.27	452.63964	14.27	1086
14.28	461.63964	14.28	1076
14.29	478.63964	14.29	1083
14.3	456.63964	14.3	1071
14.31	442.63964	14.31	1109
14.32	467.63964	14.32	1124
14.33	399.63964	14.33	1109
14.34	448.63964	14.34	1118
14.35	436.63964	14.35	1126

14.36	483.63964	14.36	1103
14.37	437.63964	14.37	1119
14.38	448.63964	14.38	1104
14.39	486.63964	14.39	1092
14.4	453.63964	14.4	1071
14.41	496.63964	14.41	1089
14.42	457.63964	14.42	1090
14.43	461.63964	14.43	1128
14.44	456.63964	14.44	1103
14.45	494.63964	14.45	1074
14.46	464.63964	14.46	1135
14.47	440.63964	14.47	1082
14.48	410.63964	14.48	1109
14.49	476.63964	14.49	1083
14.5	480.63964	14.5	1093
14.51	455.63964	14.51	1097
14.52	501.63964	14.52	1068
14.53	454.63964	14.53	1105
14.54	470.63964	14.54	1110
14.55	474.63964	14.55	1122
14.56	451.63964	14.56	1086
14.57	459.63964	14.57	1100

14.58	470.63964	14.58	1099
14.59	457.63964	14.59	1076
14.6	457.63964	14.6	1105
14.61	455.63964	14.61	1109
14.62	443.63964	14.62	1100
14.63	432.63964	14.63	1123
14.64	422.63964	14.64	1078
14.65	429.63964	14.65	1082
14.66	479.63964	14.66	1108
14.67	437.63964	14.67	1087
14.68	428.63964	14.68	1056
14.69	451.63964	14.69	1117
14.7	493.63964	14.7	1083
14.71	482.63964	14.71	1073
14.72	436.63964	14.72	1066
14.73	449.63964	14.73	1078
14.74	453.63964	14.74	1043
14.75	482.63964	14.75	1074
14.76	443.63964	14.76	1094
14.77	442.63964	14.77	1087
14.78	438.63964	14.78	1095
14.79	414.63964	14.79	1112

14.8	453.63964	14.8	1110
14.81	459.63964	14.81	1093
14.82	458.63964	14.82	1093
14.83	449.63964	14.83	1104
14.84	478.63964	14.84	1104
14.85	467.63964	14.85	1091
14.86	518.63964	14.86	1106
14.87	466.63964	14.87	1104
14.88	497.63964	14.88	1079
14.89	483.63964	14.89	1101
14.9	439.63964	14.9	1090
14.91	439.63964	14.91	1101
14.92	431.63964	14.92	1086
14.93	473.63964	14.93	1083
14.94	453.63964	14.94	1083
14.95	462.63964	14.95	1071
14.96	436.63964	14.96	1075
14.97	462.63964	14.97	1099
14.98	494.63964	14.98	1067
14.99	452.63964	14.99	1108
15	451.63964	15	1105
15.01	465.63964	15.01	1105

15.02	456.63964	15.02	1098
15.03	443.63964	15.03	1085
15.04	449.63964	15.04	1107
15.05	479.63964	15.05	1087
15.06	467.63964	15.06	1101
15.07	492.63964	15.07	1081
15.08	456.63964	15.08	1061
15.09	466.63964	15.09	1077
15.1	431.63964	15.1	1087
15.11	449.63964	15.11	1095
15.12	438.63964	15.12	1099
15.13	455.63964	15.13	1108
15.14	433.63964	15.14	1064
15.15	425.63964	15.15	1093
15.16	444.63964	15.16	1100
15.17	451.63964	15.17	1064
15.18	453.63964	15.18	1071
15.19	469.63964	15.19	1081
15.2	476.63964	15.2	1080
15.21	502.63964	15.21	1097
15.22	455.63964	15.22	1109
15.23	425.63964	15.23	1129

15.24	463.63964	15.24	1092
15.25	443.63964	15.25	1095
15.26	440.63964	15.26	1108
15.27	460.63964	15.27	1124
15.28	441.63964	15.28	1078
15.29	476.63964	15.29	1102
15.3	463.63964	15.3	1079
15.31	455.63964	15.31	1102
15.32	432.63964	15.32	1077
15.33	508.63964	15.33	1076
15.34	507.63964	15.34	1070
15.35	469.63964	15.35	1104
15.36	448.63964	15.36	1085
15.37	427.63964	15.37	1102
15.38	492.63964	15.38	1065
15.39	430.63964	15.39	1061
15.4	431.63964	15.4	1099
15.41	426.63964	15.41	1109
15.42	479.63964	15.42	1125
15.43	463.63964	15.43	1080
15.44	483.63964	15.44	1083
15.45	443.63964	15.45	1087

15.46	450.63964	15.46	1109
15.47	455.63964	15.47	1121
15.48	471.63964	15.48	1098
15.49	429.63964	15.49	1082
15.5	447.63964	15.5	1116
15.51	473.63964	15.51	1074
15.52	452.63964	15.52	1091
15.53	458.63964	15.53	1082
15.54	500.63964	15.54	1132
15.55	464.63964	15.55	1081
15.56	430.63964	15.56	1073
15.57	448.63964	15.57	1076
15.58	454.63964	15.58	1083
15.59	463.63964	15.59	1085
15.6	451.63964	15.6	1103
15.61	469.63964	15.61	1100
15.62	448.63964	15.62	1074
15.63	461.63964	15.63	1107
15.64	442.63964	15.64	1105
15.65	443.63964	15.65	1045
15.66	443.63964	15.66	1039
15.67	426.63964	15.67	1090

15.68	490.63964	15.68	1072
15.69	426.63964	15.69	1085
15.7	456.63964	15.7	1100
15.71	460.63964	15.71	1102
15.72	421.63964	15.72	1113
15.73	452.63964	15.73	1066
15.74	501.63964	15.74	1117
15.75	479.63964	15.75	1078
15.76	435.63964	15.76	1097
15.77	480.63964	15.77	1087
15.78	477.63964	15.78	1103
15.79	471.63964	15.79	1086
15.8	464.63964	15.8	1106
15.81	464.63964	15.81	1063
15.82	487.63964	15.82	1092
15.83	464.63964	15.83	1084
15.84	499.63964	15.84	1093
15.85	497.63964	15.85	1130
15.86	477.63964	15.86	1108
15.87	455.63964	15.87	1098
15.88	469.63964	15.88	1117
15.89	479.63964	15.89	1081

15.9	450.63964	15.9	1056
15.91	452.63964	15.91	1110
15.92	448.63964	15.92	1112
15.93	496.63964	15.93	1089
15.94	471.63964	15.94	1125
15.95	476.63964	15.95	1080
15.96	457.63964	15.96	1077
15.97	459.63964	15.97	1091
15.98	495.63964	15.98	1072
15.99	468.63964	15.99	1106
16	465.63964	16	1084
16.01	477.63964	16.01	1092
16.02	481.63964	16.02	1074
16.03	541.63964	16.03	1083
16.04	473.63964	16.04	1085
16.05	464.63964	16.05	1077
16.06	458.63964	16.06	1119
16.07	462.63964	16.07	1093
16.08	463.63964	16.08	1112
16.09	476.63964	16.09	1097
16.1	501.63964	16.1	1143
16.11	489.63964	16.11	1095

16.12	504.63964	16.12	1094
16.13	438.63964	16.13	1084
16.14	480.63964	16.14	1071
16.15	466.63964	16.15	1082
16.16	440.63964	16.16	1090
16.17	507.63964	16.17	1120
16.18	459.63964	16.18	1127
16.19	482.63964	16.19	1103
16.2	451.63964	16.2	1104
16.21	482.63964	16.21	1105
16.22	499.63964	16.22	1073
16.23	485.63964	16.23	1074
16.24	485.63964	16.24	1059
16.25	504.63964	16.25	1091
16.26	475.63964	16.26	1096
16.27	454.63964	16.27	1106
16.28	429.63964	16.28	1085
16.29	441.63964	16.29	1109
16.3	471.63964	16.3	1058
16.31	482.63964	16.31	1111
16.32	427.63964	16.32	1097
16.33	452.63964	16.33	1097

16.34	477.63964	16.34	1097
16.35	474.63964	16.35	1111
16.36	453.63964	16.36	1072
16.37	506.63964	16.37	1103
16.38	474.63964	16.38	1095
16.39	448.63964	16.39	1124
16.4	450.63964	16.4	1129
16.41	480.63964	16.41	1098
16.42	497.63964	16.42	1105
16.43	448.63964	16.43	1092
16.44	468.63964	16.44	1097
16.45	486.63964	16.45	1086
16.46	499.63964	16.46	1069
16.47	483.63964	16.47	1085
16.48	463.63964	16.48	1082
16.49	476.63964	16.49	1083
16.5	463.63964	16.5	1069
16.51	480.63964	16.51	1097
16.52	480.63964	16.52	1084
16.53	470.63964	16.53	1087
16.54	449.63964	16.54	1110
16.55	432.63964	16.55	1086

16.56	418.63964	16.56	1059
16.57	471.63964	16.57	1077
16.58	483.63964	16.58	1105
16.59	445.63964	16.59	1086
16.6	483.63964	16.6	1077
16.61	455.63964	16.61	1112
16.62	487.63964	16.62	1112
16.63	465.63964	16.63	1070
16.64	466.63964	16.64	1125
16.65	486.63964	16.65	1085
16.66	522.63964	16.66	1102
16.67	462.63964	16.67	1104
16.68	511.63964	16.68	1068
16.69	505.63964	16.69	1091
16.7	499.63964	16.7	1120
16.71	445.63964	16.71	1131
16.72	507.63964	16.72	1132
16.73	479.63964	16.73	1106
16.74	463.63964	16.74	1107
16.75	434.63964	16.75	1095
16.76	477.63964	16.76	1089
16.77	418.63964	16.77	1113

16.78	447.63964	16.78	1102
16.79	479.63964	16.79	1091
16.8	479.63964	16.8	1072
16.81	465.63964	16.81	1095
16.82	491.63964	16.82	1125
16.83	476.63964	16.83	1087
16.84	449.63964	16.84	1082
16.85	515.63964	16.85	1080
16.86	528.63964	16.86	1128
16.87	470.63964	16.87	1103
16.88	507.63964	16.88	1129
16.89	470.63964	16.89	1105
16.9	508.63964	16.9	1066
16.91	488.63964	16.91	1124
16.92	488.63964	16.92	1061
16.93	457.63964	16.93	1106
16.94	492.63964	16.94	1089
16.95	508.63964	16.95	1083
16.96	461.63964	16.96	1123
16.97	463.63964	16.97	1108
16.98	488.63964	16.98	1102
16.99	467.63964	16.99	1087

17	456.63964	17	1067
17.01	497.63964	17.01	1082
17.02	455.63964	17.02	1064
17.03	510.63964	17.03	1121
17.04	479.63964	17.04	1109
17.05	489.63964	17.05	1089
17.06	504.63964	17.06	1091
17.07	475.63964	17.07	1123
17.08	513.63964	17.08	1080
17.09	469.63964	17.09	1084
17.1	490.63964	17.1	1081
17.11	471.63964	17.11	1082
17.12	511.63964	17.12	1096
17.13	508.63964	17.13	1111
17.14	488.63964	17.14	1105
17.15	485.63964	17.15	1063
17.16	474.63964	17.16	1076
17.17	499.63964	17.17	1129
17.18	486.63964	17.18	1105
17.19	458.63964	17.19	1083
17.2	483.63964	17.2	1090
17.21	454.63964	17.21	1124

17.22	508.63964	17.22	1108
17.23	460.63964	17.23	1086
17.24	490.63964	17.24	1092
17.25	458.63964	17.25	1072
17.26	481.63964	17.26	1104
17.27	464.63964	17.27	1081
17.28	473.63964	17.28	1067
17.29	478.63964	17.29	1072
17.3	483.63964	17.3	1086
17.31	474.63964	17.31	1104
17.32	490.63964	17.32	1086
17.33	490.63964	17.33	1085
17.34	514.63964	17.34	1071
17.35	513.63964	17.35	1092
17.36	518.63964	17.36	1097
17.37	471.63964	17.37	1068
17.38	486.63964	17.38	1075
17.39	479.63964	17.39	1094
17.4	510.63964	17.4	1129
17.41	483.63964	17.41	1093
17.42	505.63964	17.42	1115
17.43	489.63964	17.43	1106

17.44	471.63964	17.44	1101
17.45	469.63964	17.45	1092
17.46	480.63964	17.46	1095
17.47	514.63964	17.47	1107
17.48	505.63964	17.48	1091
17.49	527.63964	17.49	1106
17.5	487.63964	17.5	1090
17.51	510.63964	17.51	1095
17.52	470.63964	17.52	1127
17.53	506.63964	17.53	1121
17.54	486.63964	17.54	1110
17.55	470.63964	17.55	1092
17.56	485.63964	17.56	1127
17.57	494.63964	17.57	1150
17.58	484.63964	17.58	1091
17.59	477.63964	17.59	1098
17.6	519.63964	17.6	1119
17.61	485.63964	17.61	1075
17.62	496.63964	17.62	1130
17.63	495.63964	17.63	1101
17.64	486.63964	17.64	1098
17.65	503.63964	17.65	1085

17.66	502.63964	17.66	1092
17.67	528.63964	17.67	1106
17.68	504.63964	17.68	1086
17.69	497.63964	17.69	1080
17.7	470.63964	17.7	1092
17.71	502.63964	17.71	1084
17.72	491.63964	17.72	1091
17.73	522.63964	17.73	1132
17.74	504.63964	17.74	1106
17.75	488.63964	17.75	1116
17.76	469.63964	17.76	1078
17.77	492.63964	17.77	1077
17.78	489.63964	17.78	1118
17.79	484.63964	17.79	1097
17.8	476.63964	17.8	1102
17.81	503.63964	17.81	1095
17.82	470.63964	17.82	1109
17.83	475.63964	17.83	1134
17.84	480.63964	17.84	1123
17.85	501.63964	17.85	1106
17.86	499.63964	17.86	1120
17.87	440.63964	17.87	1079

17.88	501.63964	17.88	1100
17.89	486.63964	17.89	1110
17.9	483.63964	17.9	1072
17.91	475.63964	17.91	1077
17.92	463.63964	17.92	1093
17.93	520.63964	17.93	1087
17.94	522.63964	17.94	1115
17.95	500.63964	17.95	1118
17.96	505.63964	17.96	1096
17.97	511.63964	17.97	1105
17.98	494.63964	17.98	1099
17.99	482.63964	17.99	1110
18	489.63964	18	1082
18.01	511.63964	18.01	1111
18.02	501.63964	18.02	1125
18.03	499.63964	18.03	1093
18.04	528.63964	18.04	1126
18.05	483.63964	18.05	1068
18.06	484.63964	18.06	1076
18.07	508.63964	18.07	1092
18.08	489.63964	18.08	1103
18.09	438.63964	18.09	1100

18.1	505.63964	18.1	1092
18.11	500.63964	18.11	1095
18.12	515.63964	18.12	1080
18.13	497.63964	18.13	1128
18.14	509.63964	18.14	1069
18.15	470.63964	18.15	1106
18.16	502.63964	18.16	1106
18.17	515.63964	18.17	1110
18.18	470.63964	18.18	1073
18.19	463.63964	18.19	1094
18.2	488.63964	18.2	1119
18.21	499.63964	18.21	1097
18.22	518.63964	18.22	1108
18.23	502.63964	18.23	1087
18.24	499.63964	18.24	1076
18.25	493.63964	18.25	1103
18.26	518.63964	18.26	1095
18.27	546.63964	18.27	1115
18.28	508.63964	18.28	1081
18.29	513.63964	18.29	1109
18.3	500.63964	18.3	1091
18.31	458.63964	18.31	1100

18.32	530.63964	18.32	1131
18.33	520.63964	18.33	1107
18.34	491.63964	18.34	1112
18.35	507.63964	18.35	1108
18.36	492.63964	18.36	1098
18.37	502.63964	18.37	1090
18.38	522.63964	18.38	1108
18.39	499.63964	18.39	1094
18.4	517.63964	18.4	1081
18.41	501.63964	18.41	1126
18.42	499.63964	18.42	1122
18.43	452.63964	18.43	1080
18.44	525.63964	18.44	1109
18.45	517.63964	18.45	1099
18.46	505.63964	18.46	1105
18.47	480.63964	18.47	1099
18.48	497.63964	18.48	1117
18.49	497.63964	18.49	1109
18.5	461.63964	18.5	1082
18.51	533.63964	18.51	1137
18.52	481.63964	18.52	1161
18.53	466.63964	18.53	1128

18.54	535.63964	18.54	1137
18.55	487.63964	18.55	1077
18.56	484.63964	18.56	1048
18.57	516.63964	18.57	1100
18.58	507.63964	18.58	1142
18.59	497.63964	18.59	1148
18.6	533.63964	18.6	1099
18.61	490.63964	18.61	1099
18.62	526.63964	18.62	1106
18.63	471.63964	18.63	1100
18.64	516.63964	18.64	1092
18.65	517.63964	18.65	1093
18.66	495.63964	18.66	1112
18.67	484.63964	18.67	1086
18.68	473.63964	18.68	1120
18.69	482.63964	18.69	1120
18.7	500.63964	18.7	1092
18.71	528.63964	18.71	1109
18.72	518.63964	18.72	1066
18.73	511.63964	18.73	1126
18.74	493.63964	18.74	1119
18.75	529.63964	18.75	1147

18.76	515.63964	18.76	1103
18.77	510.63964	18.77	1128
18.78	490.63964	18.78	1098
18.79	515.63964	18.79	1117
18.8	451.63964	18.8	1116
18.81	503.63964	18.81	1120
18.82	546.63964	18.82	1142
18.83	487.63964	18.83	1127
18.84	484.63964	18.84	1107
18.85	528.63964	18.85	1123
18.86	528.63964	18.86	1143
18.87	539.63964	18.87	1143
18.88	520.63964	18.88	1108
18.89	493.63964	18.89	1110
18.9	473.63964	18.9	1112
18.91	523.63964	18.91	1142
18.92	493.63964	18.92	1092
18.93	503.63964	18.93	1154
18.94	515.63964	18.94	1134
18.95	559.63964	18.95	1138
18.96	511.63964	18.96	1122
18.97	540.63964	18.97	1105

18.98	501.63964	18.98	1137
18.99	501.63964	18.99	1111
19	510.63964	19	1133
19.01	490.63964	19.01	1109
19.02	543.63964	19.02	1111
19.03	474.63964	19.03	1105
19.04	529.63964	19.04	1077
19.05	479.63964	19.05	1071
19.06	503.63964	19.06	1095
19.07	483.63964	19.07	1118
19.08	482.63964	19.08	1100
19.09	508.63964	19.09	1117
19.1	502.63964	19.1	1131
19.11	513.63964	19.11	1117
19.12	501.63964	19.12	1153
19.13	514.63964	19.13	1123
19.14	531.63964	19.14	1118
19.15	523.63964	19.15	1131
19.16	521.63964	19.16	1151
19.17	508.63964	19.17	1096
19.18	510.63964	19.18	1106
19.19	471.63964	19.19	1132

19.2	479.63964	19.2	1111
19.21	494.63964	19.21	1118
19.22	477.63964	19.22	1108
19.23	531.63964	19.23	1119
19.24	500.63964	19.24	1113
19.25	513.63964	19.25	1132
19.26	526.63964	19.26	1137
19.27	558.63964	19.27	1104
19.28	549.63964	19.28	1110
19.29	561.63964	19.29	1095
19.3	543.63964	19.3	1094
19.31	536.63964	19.31	1111
19.32	528.63964	19.32	1139
19.33	530.63964	19.33	1109
19.34	535.63964	19.34	1101
19.35	557.63964	19.35	1123
19.36	547.63964	19.36	1075
19.37	551.63964	19.37	1107
19.38	505.63964	19.38	1115
19.39	544.63964	19.39	1129
19.4	509.63964	19.4	1130
19.41	516.63964	19.41	1117

19.42	499.63964	19.42	1131
19.43	508.63964	19.43	1097
19.44	531.63964	19.44	1106
19.45	531.63964	19.45	1130
19.46	545.63964	19.46	1121
19.47	529.63964	19.47	1127
19.48	525.63964	19.48	1115
19.49	512.63964	19.49	1143
19.5	501.63964	19.5	1131
19.51	509.63964	19.51	1141
19.52	538.63964	19.52	1106
19.53	525.63964	19.53	1097
19.54	536.63964	19.54	1108
19.55	492.63964	19.55	1116
19.56	565.63964	19.56	1142
19.57	535.63964	19.57	1127
19.58	495.63964	19.58	1113
19.59	531.63964	19.59	1124
19.6	574.63964	19.6	1125
19.61	542.63964	19.61	1128
19.62	561.63964	19.62	1118
19.63	510.63964	19.63	1147

19.64	511.63964	19.64	1115
19.65	520.63964	19.65	1115
19.66	531.63964	19.66	1127
19.67	509.63964	19.67	1135
19.68	491.63964	19.68	1115
19.69	528.63964	19.69	1156
19.7	529.63964	19.7	1145
19.71	534.63964	19.71	1127
19.72	535.63964	19.72	1154
19.73	502.63964	19.73	1144
19.74	529.63964	19.74	1112
19.75	505.63964	19.75	1098
19.76	522.63964	19.76	1077
19.77	562.63964	19.77	1134
19.78	534.63964	19.78	1133
19.79	507.63964	19.79	1128
19.8	473.63964	19.8	1142
19.81	518.63964	19.81	1109
19.82	518.63964	19.82	1146
19.83	493.63964	19.83	1106
19.84	512.63964	19.84	1080
19.85	504.63964	19.85	1130

19.86	522.63964	19.86	1122
19.87	527.63964	19.87	1137
19.88	481.63964	19.88	1130
19.89	522.63964	19.89	1115
19.9	519.63964	19.9	1150
19.91	545.63964	19.91	1110
19.92	530.63964	19.92	1129
19.93	506.63964	19.93	1115
19.94	536.63964	19.94	1117
19.95	507.63964	19.95	1127
19.96	536.63964	19.96	1120
19.97	529.63964	19.97	1126
19.98	489.63964	19.98	1178
19.99	511.63964	19.99	1106
20	531.63964	20	1148
20.01	515.63964	20.01	1126
20.02	552.63964	20.02	1116
20.03	511.63964	20.03	1121
20.04	478.63964	20.04	1122
20.05	542.63964	20.05	1138
20.06	540.63964	20.06	1101
20.07	549.63964	20.07	1110

20.08	511.63964	20.08	1131
20.09	553.63964	20.09	1129
20.1	571.63964	20.1	1118
20.11	509.63964	20.11	1167
20.12	500.63964	20.12	1092
20.13	511.63964	20.13	1143
20.14	517.63964	20.14	1142
20.15	528.63964	20.15	1126
20.16	552.63964	20.16	1137
20.17	519.63964	20.17	1121
20.18	525.63964	20.18	1120
20.19	523.63964	20.19	1119
20.2	543.63964	20.2	1148
20.21	538.63964	20.21	1132
20.22	530.63964	20.22	1129
20.23	541.63964	20.23	1158
20.24	556.63964	20.24	1115
20.25	491.63964	20.25	1132
20.26	533.63964	20.26	1109
20.27	519.63964	20.27	1138
20.28	593.63964	20.28	1129
20.29	534.63964	20.29	1134

20.3	521.63964	20.3	1167
20.31	525.63964	20.31	1131
20.32	555.63964	20.32	1125
20.33	514.63964	20.33	1110
20.34	510.63964	20.34	1138
20.35	552.63964	20.35	1143
20.36	526.63964	20.36	1170
20.37	515.63964	20.37	1130
20.38	503.63964	20.38	1128
20.39	519.63964	20.39	1146
20.4	557.63964	20.4	1115
20.41	532.63964	20.41	1150
20.42	537.63964	20.42	1151
20.43	571.63964	20.43	1143
20.44	530.63964	20.44	1108
20.45	553.63964	20.45	1124
20.46	572.63964	20.46	1113
20.47	582.63964	20.47	1143
20.48	530.63964	20.48	1154
20.49	526.63964	20.49	1135
20.5	532.63964	20.5	1155
20.51	501.63964	20.51	1153

20.52	484.63964	20.52	1117
20.53	547.63964	20.53	1131
20.54	527.63964	20.54	1122
20.55	551.63964	20.55	1133
20.56	530.63964	20.56	1126
20.57	568.63964	20.57	1124
20.58	521.63964	20.58	1137
20.59	514.63964	20.59	1096
20.6	528.63964	20.6	1138
20.61	493.63964	20.61	1130
20.62	536.63964	20.62	1108
20.63	529.63964	20.63	1142
20.64	506.63964	20.64	1106
20.65	518.63964	20.65	1151
20.66	587.63964	20.66	1138
20.67	517.63964	20.67	1145
20.68	538.63964	20.68	1184
20.69	513.63964	20.69	1141
20.7	517.63964	20.7	1130
20.71	521.63964	20.71	1128
20.72	504.63964	20.72	1142
20.73	556.63964	20.73	1149

20.74	562.63964	20.74	1150
20.75	525.63964	20.75	1150
20.76	547.63964	20.76	1117
20.77	525.63964	20.77	1096
20.78	536.63964	20.78	1121
20.79	571.63964	20.79	1162
20.8	564.63964	20.8	1115
20.81	529.63964	20.81	1128
20.82	512.63964	20.82	1152
20.83	510.63964	20.83	1111
20.84	562.63964	20.84	1151
20.85	531.63964	20.85	1138
20.86	559.63964	20.86	1135
20.87	514.63964	20.87	1141
20.88	530.63964	20.88	1149
20.89	546.63964	20.89	1135
20.9	530.63964	20.9	1124
20.91	561.63964	20.91	1163
20.92	519.63964	20.92	1123
20.93	560.63964	20.93	1151
20.94	554.63964	20.94	1139
20.95	512.63964	20.95	1094

20.96	551.63964	20.96	1183
20.97	517.63964	20.97	1152
20.98	522.63964	20.98	1137
20.99	533.63964	20.99	1109
21	504.63964	21	1116
21.01	543.63964	21.01	1147
21.02	533.63964	21.02	1161
21.03	527.63964	21.03	1122
21.04	551.63964	21.04	1122
21.05	566.63964	21.05	1135
21.06	531.63964	21.06	1163
21.07	542.63964	21.07	1182
21.08	517.63964	21.08	1144
21.09	570.63964	21.09	1118
21.1	562.63964	21.1	1134
21.11	530.63964	21.11	1183
21.12	488.63964	21.12	1161
21.13	541.63964	21.13	1149
21.14	555.63964	21.14	1132
21.15	552.63964	21.15	1107
21.16	526.63964	21.16	1142
21.17	533.63964	21.17	1136

21.18	545.63964	21.18	1156
21.19	525.63964	21.19	1159
21.2	571.63964	21.2	1195
21.21	578.63964	21.21	1141
21.22	555.63964	21.22	1133
21.23	567.63964	21.23	1142
21.24	545.63964	21.24	1177
21.25	531.63964	21.25	1205
21.26	547.63964	21.26	1181
21.27	551.63964	21.27	1152
21.28	556.63964	21.28	1169
21.29	535.63964	21.29	1157
21.3	555.63964	21.3	1154
21.31	572.63964	21.31	1166
21.32	536.63964	21.32	1198
21.33	554.63964	21.33	1194
21.34	517.63964	21.34	1178
21.35	551.63964	21.35	1185
21.36	547.63964	21.36	1125
21.37	530.63964	21.37	1171
21.38	559.63964	21.38	1190
21.39	568.63964	21.39	1197

21.4	543.63964	21.4	1177
21.41	536.63964	21.41	1232
21.42	574.63964	21.42	1238
21.43	531.63964	21.43	1199
21.44	596.63964	21.44	1206
21.45	561.63964	21.45	1168
21.46	558.63964	21.46	1173
21.47	562.63964	21.47	1170
21.48	572.63964	21.48	1178
21.49	601.63964	21.49	1170
21.5	606.63964	21.5	1197
21.51	562.63964	21.51	1156
21.52	502.63964	21.52	1160
21.53	490.63964	21.53	1167
21.54	563.63964	21.54	1149
21.55	544.63964	21.55	1168
21.56	539.63964	21.56	1185
21.57	555.63964	21.57	1161
21.58	545.63964	21.58	1197
21.59	539.63964	21.59	1138
21.6	592.63964	21.6	1178
21.61	534.63964	21.61	1155

21.62	558.63964	21.62	1144
21.63	574.63964	21.63	1157
21.64	531.63964	21.64	1182
21.65	539.63964	21.65	1147
21.66	554.63964	21.66	1151
21.67	544.63964	21.67	1162
21.68	555.63964	21.68	1146
21.69	555.63964	21.69	1168
21.7	537.63964	21.7	1182
21.71	558.63964	21.71	1143
21.72	589.63964	21.72	1130
21.73	549.63964	21.73	1120
21.74	585.63964	21.74	1108
21.75	588.63964	21.75	1145
21.76	551.63964	21.76	1115
21.77	573.63964	21.77	1209
21.78	535.63964	21.78	1190
21.79	562.63964	21.79	1111
21.8	568.63964	21.8	1178
21.81	553.63964	21.81	1186
21.82	570.63964	21.82	1164
21.83	554.63964	21.83	1153

21.84	579.63964	21.84	1146
21.85	537.63964	21.85	1132
21.86	523.63964	21.86	1181
21.87	579.63964	21.87	1160
21.88	574.63964	21.88	1130
21.89	585.63964	21.89	1141
21.9	535.63964	21.9	1160
21.91	584.63964	21.91	1158
21.92	537.63964	21.92	1180
21.93	544.63964	21.93	1146
21.94	537.63964	21.94	1136
21.95	604.63964	21.95	1147
21.96	558.63964	21.96	1172
21.97	521.63964	21.97	1138
21.98	556.63964	21.98	1151
21.99	542.63964	21.99	1134
22	615.63964	22	1130
22.01	598.63964	22.01	1151
22.02	514.63964	22.02	1150
22.03	609.63964	22.03	1129
22.04	545.63964	22.04	1137
22.05	528.63964	22.05	1136

22.06	556.63964	22.06	1139
22.07	557.63964	22.07	1151
22.08	557.63964	22.08	1167
22.09	585.63964	22.09	1150
22.1	557.63964	22.1	1120
22.11	519.63964	22.11	1104
22.12	609.63964	22.12	1122
22.13	562.63964	22.13	1134
22.14	535.63964	22.14	1148
22.15	582.63964	22.15	1143
22.16	540.63964	22.16	1146
22.17	555.63964	22.17	1148
22.18	532.63964	22.18	1197
22.19	561.63964	22.19	1150
22.2	551.63964	22.2	1154
22.21	518.63964	22.21	1159
22.22	533.63964	22.22	1159
22.23	540.63964	22.23	1109
22.24	553.63964	22.24	1156
22.25	567.63964	22.25	1152
22.26	574.63964	22.26	1133
22.27	610.63964	22.27	1119

22.28	559.63964	22.28	1149
22.29	565.63964	22.29	1138
22.3	558.63964	22.3	1162
22.31	526.63964	22.31	1146
22.32	555.63964	22.32	1153
22.33	548.63964	22.33	1157
22.34	541.63964	22.34	1157
22.35	535.63964	22.35	1171
22.36	554.63964	22.36	1158
22.37	547.63964	22.37	1111
22.38	573.63964	22.38	1166
22.39	587.63964	22.39	1139
22.4	612.63964	22.4	1178
22.41	546.63964	22.41	1148
22.42	542.63964	22.42	1149
22.43	564.63964	22.43	1153
22.44	562.63964	22.44	1133
22.45	559.63964	22.45	1125
22.46	547.63964	22.46	1142
22.47	535.63964	22.47	1146
22.48	562.63964	22.48	1157
22.49	537.63964	22.49	1175

22.5	562.63964	22.5	1136
22.51	557.63964	22.51	1159
22.52	599.63964	22.52	1156
22.53	565.63964	22.53	1145
22.54	581.63964	22.54	1153
22.55	537.63964	22.55	1174
22.56	561.63964	22.56	1149
22.57	585.63964	22.57	1139
22.58	525.63964	22.58	1151
22.59	590.63964	22.59	1186
22.6	591.63964	22.6	1156
22.61	535.63964	22.61	1137
22.62	561.63964	22.62	1141
22.63	589.63964	22.63	1108
22.64	558.63964	22.64	1145
22.65	560.63964	22.65	1133
22.66	561.63964	22.66	1149
22.67	529.63964	22.67	1153
22.68	545.63964	22.68	1181
22.69	584.63964	22.69	1163
22.7	557.63964	22.7	1137
22.71	554.63964	22.71	1175

22.72	560.63964	22.72	1171
22.73	572.63964	22.73	1148
22.74	547.63964	22.74	1155
22.75	561.63964	22.75	1118
22.76	569.63964	22.76	1168
22.77	599.63964	22.77	1139
22.78	590.63964	22.78	1158
22.79	544.63964	22.79	1134
22.8	581.63964	22.8	1161
22.81	570.63964	22.81	1132
22.82	581.63964	22.82	1166
22.83	577.63964	22.83	1155
22.84	604.63964	22.84	1146
22.85	532.63964	22.85	1166
22.86	620.63964	22.86	1150
22.87	546.63964	22.87	1133
22.88	551.63964	22.88	1161
22.89	566.63964	22.89	1121
22.9	552.63964	22.9	1136
22.91	573.63964	22.91	1135
22.92	580.63964	22.92	1146
22.93	568.63964	22.93	1164

22.94	595.63964	22.94	1143
22.95	589.63964	22.95	1171
22.96	573.63964	22.96	1172
22.97	574.63964	22.97	1183
22.98	573.63964	22.98	1153
22.99	583.63964	22.99	1138
23	541.63964	23	1160
23.01	563.63964	23.01	1156
23.02	584.63964	23.02	1157
23.03	542.63964	23.03	1163
23.04	562.63964	23.04	1156
23.05	581.63964	23.05	1142
23.06	609.63964	23.06	1177
23.07	564.63964	23.07	1200
23.08	523.63964	23.08	1155
23.09	570.63964	23.09	1136
23.1	594.63964	23.1	1137
23.11	566.63964	23.11	1181
23.12	586.63964	23.12	1160
23.13	506.63964	23.13	1156
23.14	559.63964	23.14	1150
23.15	596.63964	23.15	1144

23.16	572.63964	23.16	1156
23.17	569.63964	23.17	1193
23.18	553.63964	23.18	1164
23.19	551.63964	23.19	1131
23.2	559.63964	23.2	1157
23.21	559.63964	23.21	1136
23.22	557.63964	23.22	1180
23.23	575.63964	23.23	1161
23.24	556.63964	23.24	1184
23.25	566.63964	23.25	1113
23.26	551.63964	23.26	1125
23.27	557.63964	23.27	1144
23.28	586.63964	23.28	1142
23.29	556.63964	23.29	1141
23.3	581.63964	23.3	1120
23.31	556.63964	23.31	1151
23.32	608.63964	23.32	1160
23.33	574.63964	23.33	1145
23.34	603.63964	23.34	1170
23.35	569.63964	23.35	1182
23.36	581.63964	23.36	1200
23.37	591.63964	23.37	1166

23.38	594.63964	23.38	1156
23.39	579.63964	23.39	1154
23.4	545.63964	23.4	1162
23.41	610.63964	23.41	1129
23.42	553.63964	23.42	1152
23.43	561.63964	23.43	1147
23.44	594.63964	23.44	1168
23.45	564.63964	23.45	1159
23.46	582.63964	23.46	1105
23.47	626.63964	23.47	1184
23.48	581.63964	23.48	1169
23.49	551.63964	23.49	1175
23.5	588.63964	23.5	1189
23.51	583.63964	23.51	1126
23.52	560.63964	23.52	1174
23.53	551.63964	23.53	1169
23.54	566.63964	23.54	1180
23.55	552.63964	23.55	1161
23.56	578.63964	23.56	1149
23.57	536.63964	23.57	1162
23.58	543.63964	23.58	1137
23.59	520.63964	23.59	1123

23.6	568.63964	23.6	1170
23.61	559.63964	23.61	1128
23.62	542.63964	23.62	1151
23.63	547.63964	23.63	1163
23.64	519.63964	23.64	1155
23.65	534.63964	23.65	1175
23.66	542.63964	23.66	1177
23.67	516.63964	23.67	1147
23.68	587.63964	23.68	1215
23.69	583.63964	23.69	1164
23.7	530.63964	23.7	1166
23.71	547.63964	23.71	1177
23.72	582.63964	23.72	1206
23.73	552.63964	23.73	1131
23.74	581.63964	23.74	1142
23.75	578.63964	23.75	1150
23.76	515.63964	23.76	1137
23.77	566.63964	23.77	1155
23.78	537.63964	23.78	1139
23.79	517.63964	23.79	1168
23.8	607.63964	23.8	1192
23.81	588.63964	23.81	1173

23.82	584.63964	23.82	1148
23.83	599.63964	23.83	1152
23.84	603.63964	23.84	1176
23.85	559.63964	23.85	1177
23.86	557.63964	23.86	1157
23.87	593.63964	23.87	1168
23.88	556.63964	23.88	1163
23.89	560.63964	23.89	1183
23.9	552.63964	23.9	1178
23.91	557.63964	23.91	1174
23.92	619.63964	23.92	1154
23.93	578.63964	23.93	1154
23.94	585.63964	23.94	1166
23.95	543.63964	23.95	1159
23.96	568.63964	23.96	1186
23.97	573.63964	23.97	1178
23.98	565.63964	23.98	1151
23.99	549.63964	23.99	1128
24	620.63964	24	1132
24.01	578.63964	24.01	1132
24.02	605.63964	24.02	1167
24.03	609.63964	24.03	1139

24.04	531.63964	24.04	1128
24.05	538.63964	24.05	1171
24.06	580.63964	24.06	1158
24.07	580.63964	24.07	1196
24.08	588.63964	24.08	1164
24.09	584.63964	24.09	1172
24.1	559.63964	24.1	1202
24.11	583.63964	24.11	1152
24.12	591.63964	24.12	1163
24.13	595.63964	24.13	1154
24.14	526.63964	24.14	1167
24.15	575.63964	24.15	1120
24.16	577.63964	24.16	1185
24.17	557.63964	24.17	1180
24.18	609.63964	24.18	1145
24.19	574.63964	24.19	1132
24.2	569.63964	24.2	1197
24.21	573.63964	24.21	1156
24.22	558.63964	24.22	1189
24.23	575.63964	24.23	1179
24.24	568.63964	24.24	1148
24.25	548.63964	24.25	1129

24.26	598.63964	24.26	1175
24.27	565.63964	24.27	1175
24.28	543.63964	24.28	1150
24.29	634.63964	24.29	1150
24.3	558.63964	24.3	1162
24.31	560.63964	24.31	1164
24.32	562.63964	24.32	1142
24.33	566.63964	24.33	1130
24.34	571.63964	24.34	1167
24.35	562.63964	24.35	1178
24.36	581.63964	24.36	1141
24.37	552.63964	24.37	1104
24.38	546.63964	24.38	1128
24.39	593.63964	24.39	1145
24.4	583.63964	24.4	1115
24.41	541.63964	24.41	1157
24.42	565.63964	24.42	1143
24.43	575.63964	24.43	1150
24.44	596.63964	24.44	1161
24.45	582.63964	24.45	1164
24.46	614.63964	24.46	1146
24.47	586.63964	24.47	1155

24.48	594.63964	24.48	1148
24.49	587.63964	24.49	1125
24.5	575.63964	24.5	1169
24.51	569.63964	24.51	1149
24.52	573.63964	24.52	1145
24.53	587.63964	24.53	1147
24.54	584.63964	24.54	1175
24.55	601.63964	24.55	1137
24.56	568.63964	24.56	1174
24.57	584.63964	24.57	1131
24.58	572.63964	24.58	1126
24.59	562.63964	24.59	1123
24.6	566.63964	24.6	1132
24.61	579.63964	24.61	1134
24.62	581.63964	24.62	1141
24.63	564.63964	24.63	1138
24.64	594.63964	24.64	1109
24.65	560.63964	24.65	1154
24.66	588.63964	24.66	1128
24.67	587.63964	24.67	1156
24.68	574.63964	24.68	1168
24.69	578.63964	24.69	1129

24.7	595.63964	24.7	1172
24.71	615.63964	24.71	1195
24.72	591.63964	24.72	1150
24.73	601.63964	24.73	1149
24.74	555.63964	24.74	1135
24.75	558.63964	24.75	1131
24.76	616.63964	24.76	1170
24.77	608.63964	24.77	1157
24.78	570.63964	24.78	1157
24.79	620.63964	24.79	1178
24.8	548.63964	24.8	1192
24.81	591.63964	24.81	1161
24.82	575.63964	24.82	1128
24.83	591.63964	24.83	1128
24.84	606.63964	24.84	1154
24.85	583.63964	24.85	1195
24.86	613.63964	24.86	1143
24.87	590.63964	24.87	1156
24.88	555.63964	24.88	1145
24.89	600.63964	24.89	1136
24.9	587.63964	24.9	1128
24.91	575.63964	24.91	1141

24.92	545.63964	24.92	1154
24.93	594.63964	24.93	1139
24.94	609.63964	24.94	1166
24.95	564.63964	24.95	1158
24.96	582.63964	24.96	1136
24.97	622.63964	24.97	1138
24.98	581.63964	24.98	1158
24.99	594.63964	24.99	1164
25	578.63964	25	1166
25.01	554.63964	25.01	1118
25.02	560.63964	25.02	1123
25.03	562.63964	25.03	1127
25.04	590.63964	25.04	1113
25.05	614.63964	25.05	1131
25.06	582.63964	25.06	1158
25.07	554.63964	25.07	1128
25.08	597.63964	25.08	1153
25.09	589.63964	25.09	1154
25.1	564.63964	25.1	1128
25.11	582.63964	25.11	1142
25.12	521.63964	25.12	1150
25.13	564.63964	25.13	1137

25.14	580.63964	25.14	1175
25.15	547.63964	25.15	1126
25.16	565.63964	25.16	1162
25.17	573.63964	25.17	1153
25.18	561.63964	25.18	1139
25.19	565.63964	25.19	1123
25.2	584.63964	25.2	1169
25.21	585.63964	25.21	1162
25.22	580.63964	25.22	1158
25.23	575.63964	25.23	1138
25.24	559.63964	25.24	1143
25.25	568.63964	25.25	1163
25.26	580.63964	25.26	1136
25.27	574.63964	25.27	1121
25.28	551.63964	25.28	1127
25.29	585.63964	25.29	1122
25.3	596.63964	25.3	1119
25.31	603.63964	25.31	1118
25.32	579.63964	25.32	1134
25.33	565.63964	25.33	1150
25.34	585.63964	25.34	1129
25.35	594.63964	25.35	1148

25.36	630.63964	25.36	1126
25.37	604.63964	25.37	1141
25.38	591.63964	25.38	1145
25.39	607.63964	25.39	1129
25.4	620.63964	25.4	1148
25.41	588.63964	25.41	1180
25.42	593.63964	25.42	1167
25.43	569.63964	25.43	1143
25.44	594.63964	25.44	1153
25.45	574.63964	25.45	1158
25.46	597.63964	25.46	1142
25.47	637.63964	25.47	1122
25.48	600.63964	25.48	1127
25.49	586.63964	25.49	1131
25.5	592.63964	25.5	1126
25.51	640.63964	25.51	1135
25.52	617.63964	25.52	1154
25.53	599.63964	25.53	1162
25.54	587.63964	25.54	1158
25.55	557.63964	25.55	1159
25.56	560.63964	25.56	1142
25.57	594.63964	25.57	1133

25.58	554.63964	25.58	1141
25.59	571.63964	25.59	1156
25.6	615.63964	25.6	1124
25.61	534.63964	25.61	1157
25.62	521.63964	25.62	1142
25.63	547.63964	25.63	1112
25.64	559.63964	25.64	1153
25.65	598.63964	25.65	1159
25.66	594.63964	25.66	1158
25.67	537.63964	25.67	1138
25.68	574.63964	25.68	1134
25.69	617.63964	25.69	1135
25.7	592.63964	25.7	1134
25.71	582.63964	25.71	1115
25.72	562.63964	25.72	1136
25.73	622.63964	25.73	1133
25.74	578.63964	25.74	1148
25.75	564.63964	25.75	1152
25.76	575.63964	25.76	1128
25.77	585.63964	25.77	1146
25.78	600.63964	25.78	1129
25.79	580.63964	25.79	1132

25.8	638.63964	25.8	1091
25.81	608.63964	25.81	1133
25.82	598.63964	25.82	1134
25.83	540.63964	25.83	1141
25.84	571.63964	25.84	1104
25.85	570.63964	25.85	1153
25.86	610.63964	25.86	1143
25.87	574.63964	25.87	1131
25.88	612.63964	25.88	1147
25.89	630.63964	25.89	1137
25.9	590.63964	25.9	1108
25.91	617.63964	25.91	1102
25.92	584.63964	25.92	1164
25.93	590.63964	25.93	1146
25.94	605.63964	25.94	1126
25.95	594.63964	25.95	1174
25.96	554.63964	25.96	1154
25.97	534.63964	25.97	1170
25.98	608.63964	25.98	1138
25.99	607.63964	25.99	1136
26	586.63964	26	1096
26.01	554.63964	26.01	1186

26.02	560.63964	26.02	1111
26.03	539.63964	26.03	1125
26.04	600.63964	26.04	1137
26.05	630.63964	26.05	1112
26.06	570.63964	26.06	1127
26.07	547.63964	26.07	1108
26.08	591.63964	26.08	1174
26.09	571.63964	26.09	1150
26.1	616.63964	26.1	1130
26.11	584.63964	26.11	1133
26.12	599.63964	26.12	1148
26.13	574.63964	26.13	1167
26.14	608.63964	26.14	1156
26.15	605.63964	26.15	1124
26.16	601.63964	26.16	1144
26.17	584.63964	26.17	1179
26.18	572.63964	26.18	1125
26.19	616.63964	26.19	1120
26.2	592.63964	26.2	1142
26.21	605.63964	26.21	1139
26.22	585.63964	26.22	1123
26.23	611.63964	26.23	1126

26.24	600.63964	26.24	1157
26.25	612.63964	26.25	1167
26.26	610.63964	26.26	1152
26.27	571.63964	26.27	1141
26.28	598.63964	26.28	1130
26.29	604.63964	26.29	1164
26.3	592.63964	26.3	1143
26.31	616.63964	26.31	1157
26.32	647.63964	26.32	1125
26.33	610.63964	26.33	1148
26.34	589.63964	26.34	1176
26.35	609.63964	26.35	1135
26.36	581.63964	26.36	1077
26.37	597.63964	26.37	1137
26.38	618.63964	26.38	1137
26.39	607.63964	26.39	1126
26.4	565.63964	26.4	1131
26.41	601.63964	26.41	1136
26.42	612.63964	26.42	1161
26.43	621.63964	26.43	1169
26.44	597.63964	26.44	1182
26.45	613.63964	26.45	1152

26.46	600.63964	26.46	1128
26.47	622.63964	26.47	1135
26.48	587.63964	26.48	1146
26.49	604.63964	26.49	1156
26.5	632.63964	26.5	1141
26.51	567.63964	26.51	1144
26.52	616.63964	26.52	1136
26.53	609.63964	26.53	1143
26.54	561.63964	26.54	1145
26.55	634.63964	26.55	1161
26.56	658.63964	26.56	1158
26.57	625.63964	26.57	1139
26.58	598.63964	26.58	1161
26.59	611.63964	26.59	1170
26.6	591.63964	26.6	1196
26.61	605.63964	26.61	1159
26.62	660.63964	26.62	1106
26.63	587.63964	26.63	1166
26.64	569.63964	26.64	1146
26.65	647.63964	26.65	1117
26.66	633.63964	26.66	1158
26.67	610.63964	26.67	1172

26.68	619.63964	26.68	1175
26.69	631.63964	26.69	1186
26.7	583.63964	26.7	1184
26.71	664.63964	26.71	1146
26.72	637.63964	26.72	1142
26.73	622.63964	26.73	1170
26.74	616.63964	26.74	1134
26.75	582.63964	26.75	1158
26.76	618.63964	26.76	1120
26.77	649.63964	26.77	1145
26.78	632.63964	26.78	1148
26.79	650.63964	26.79	1142
26.8	642.63964	26.8	1145
26.81	656.63964	26.81	1162
26.82	625.63964	26.82	1151
26.83	600.63964	26.83	1178
26.84	668.63964	26.84	1195
26.85	630.63964	26.85	1156
26.86	655.63964	26.86	1201
26.87	663.63964	26.87	1144
26.88	610.63964	26.88	1160
26.89	656.63964	26.89	1146

26.9	662.63964	26.9	1163
26.91	675.63964	26.91	1184
26.92	652.63964	26.92	1144
26.93	633.63964	26.93	1158
26.94	616.63964	26.94	1178
26.95	655.63964	26.95	1171
26.96	660.63964	26.96	1143
26.97	691.63964	26.97	1156
26.98	656.63964	26.98	1176
26.99	665.63964	26.99	1159
27	681.63964	27	1146
27.01	699.63964	27.01	1180
27.02	711.63964	27.02	1160
27.03	692.63964	27.03	1153
27.04	696.63964	27.04	1169
27.05	696.63964	27.05	1182
27.06	717.63964	27.06	1174
27.07	656.63964	27.07	1180
27.08	677.63964	27.08	1161
27.09	700.63964	27.09	1186
27.1	685.63964	27.1	1175
27.11	703.63964	27.11	1190

27.12	675.63964	27.12	1160
27.13	710.63964	27.13	1177
27.14	762.63964	27.14	1163
27.15	726.63964	27.15	1168
27.16	697.63964	27.16	1187
27.17	685.63964	27.17	1169
27.18	728.63964	27.18	1170
27.19	715.63964	27.19	1203
27.2	728.63964	27.2	1206
27.21	724.63964	27.21	1172
27.22	675.63964	27.22	1208
27.23	697.63964	27.23	1184
27.24	768.63964	27.24	1166
27.25	735.63964	27.25	1187
27.26	720.63964	27.26	1161
27.27	733.63964	27.27	1170
27.28	719.63964	27.28	1210
27.29	669.63964	27.29	1193
27.3	711.63964	27.3	1187
27.31	685.63964	27.31	1159
27.32	695.63964	27.32	1193
27.33	704.63964	27.33	1196

27.34	673.63964	27.34	1195
27.35	698.63964	27.35	1168
27.36	732.63964	27.36	1196
27.37	684.63964	27.37	1200
27.38	698.63964	27.38	1186
27.39	718.63964	27.39	1171
27.4	695.63964	27.4	1145
27.41	672.63964	27.41	1167
27.42	708.63964	27.42	1192
27.43	707.63964	27.43	1222
27.44	734.63964	27.44	1175
27.45	687.63964	27.45	1188
27.46	711.63964	27.46	1189
27.47	719.63964	27.47	1184
27.48	729.63964	27.48	1144
27.49	702.63964	27.49	1130
27.5	702.63964	27.5	1186
27.51	728.63964	27.51	1163
27.52	715.63964	27.52	1145
27.53	723.63964	27.53	1195
27.54	771.63964	27.54	1202
27.55	748.63964	27.55	1198

27.56	774.63964	27.56	1178
27.57	758.63964	27.57	1179
27.58	693.63964	27.58	1178
27.59	736.63964	27.59	1146
27.6	758.63964	27.6	1189
27.61	750.63964	27.61	1187
27.62	741.63964	27.62	1160
27.63	750.63964	27.63	1156
27.64	739.63964	27.64	1212
27.65	740.63964	27.65	1209
27.66	777.63964	27.66	1203
27.67	744.63964	27.67	1190
27.68	771.63964	27.68	1199
27.69	772.63964	27.69	1233
27.7	830.63964	27.7	1186
27.71	733.63964	27.71	1225
27.72	720.63964	27.72	1205
27.73	775.63964	27.73	1171
27.74	758.63964	27.74	1156
27.75	750.63964	27.75	1192
27.76	758.63964	27.76	1197
27.77	758.63964	27.77	1207

27.78	749.63964	27.78	1234
27.79	741.63964	27.79	1212
27.8	750.63964	27.8	1190
27.81	768.63964	27.81	1197
27.82	738.63964	27.82	1199
27.83	720.63964	27.83	1216
27.84	737.63964	27.84	1181
27.85	728.63964	27.85	1178
27.86	766.63964	27.86	1168
27.87	750.63964	27.87	1202
27.88	672.63964	27.88	1202
27.89	712.63964	27.89	1196
27.9	752.63964	27.9	1202
27.91	758.63964	27.91	1171
27.92	750.63964	27.92	1134
27.93	690.63964	27.93	1172
27.94	786.63964	27.94	1123
27.95	767.63964	27.95	1170
27.96	762.63964	27.96	1190
27.97	700.63964	27.97	1200
27.98	734.63964	27.98	1173
27.99	768.63964	27.99	1181

28	733.63964	28	1168
28.01	738.63964	28.01	1154
28.02	702.63964	28.02	1126
28.03	739.63964	28.03	1174
28.04	725.63964	28.04	1174
28.05	703.63964	28.05	1168
28.06	691.63964	28.06	1164
28.07	718.63964	28.07	1185
28.08	704.63964	28.08	1176
28.09	695.63964	28.09	1137
28.1	714.63964	28.1	1186
28.11	730.63964	28.11	1177
28.12	723.63964	28.12	1187
28.13	688.63964	28.13	1145
28.14	712.63964	28.14	1192
28.15	692.63964	28.15	1219
28.16	749.63964	28.16	1194
28.17	717.63964	28.17	1194
28.18	753.63964	28.18	1190
28.19	693.63964	28.19	1190
28.2	729.63964	28.2	1152
28.21	733.63964	28.21	1152

28.22	726.63964	28.22	1167
28.23	688.63964	28.23	1190
28.24	715.63964	28.24	1156
28.25	648.63964	28.25	1187
28.26	677.63964	28.26	1168
28.27	677.63964	28.27	1214
28.28	691.63964	28.28	1186
28.29	642.63964	28.29	1171
28.3	716.63964	28.3	1141
28.31	698.63964	28.31	1181
28.32	668.63964	28.32	1169
28.33	633.63964	28.33	1148
28.34	698.63964	28.34	1172
28.35	713.63964	28.35	1125
28.36	723.63964	28.36	1168
28.37	701.63964	28.37	1175
28.38	694.63964	28.38	1193
28.39	687.63964	28.39	1199
28.4	706.63964	28.4	1199
28.41	695.63964	28.41	1169
28.42	714.63964	28.42	1188
28.43	695.63964	28.43	1179

28.44	682.63964	28.44	1185
28.45	696.63964	28.45	1160
28.46	692.63964	28.46	1183
28.47	714.63964	28.47	1180
28.48	720.63964	28.48	1127
28.49	680.63964	28.49	1197
28.5	699.63964	28.5	1193
28.51	714.63964	28.51	1195
28.52	688.63964	28.52	1129
28.53	716.63964	28.53	1175
28.54	733.63964	28.54	1175
28.55	711.63964	28.55	1157
28.56	668.63964	28.56	1170
28.57	728.63964	28.57	1183
28.58	674.63964	28.58	1170
28.59	708.63964	28.59	1163
28.6	734.63964	28.6	1154
28.61	732.63964	28.61	1192
28.62	785.63964	28.62	1151
28.63	734.63964	28.63	1161
28.64	669.63964	28.64	1183
28.65	747.63964	28.65	1180

28.66	742.63964	28.66	1161
28.67	717.63964	28.67	1184
28.68	760.63964	28.68	1167
28.69	735.63964	28.69	1150
28.7	738.63964	28.7	1218
28.71	734.63964	28.71	1201
28.72	751.63964	28.72	1214
28.73	763.63964	28.73	1185
28.74	692.63964	28.74	1195
28.75	751.63964	28.75	1195
28.76	714.63964	28.76	1152
28.77	753.63964	28.77	1180
28.78	775.63964	28.78	1229
28.79	729.63964	28.79	1199
28.8	720.63964	28.8	1237
28.81	696.63964	28.81	1240
28.82	770.63964	28.82	1177
28.83	761.63964	28.83	1160
28.84	785.63964	28.84	1192
28.85	766.63964	28.85	1207
28.86	807.63964	28.86	1204
28.87	779.63964	28.87	1212

28.88	760.63964	28.88	1234
28.89	778.63964	28.89	1186
28.9	722.63964	28.9	1201
28.91	753.63964	28.91	1203
28.92	777.63964	28.92	1207
28.93	854.63964	28.93	1218
28.94	811.63964	28.94	1210
28.95	765.63964	28.95	1254
28.96	828.63964	28.96	1195
28.97	795.63964	28.97	1213
28.98	837.63964	28.98	1236
28.99	837.63964	28.99	1234
29	800.63964	29	1229
29.01	853.63964	29.01	1219
29.02	813.63964	29.02	1198
29.03	857.63964	29.03	1225
29.04	846.63964	29.04	1212
29.05	821.63964	29.05	1220
29.06	869.63964	29.06	1208
29.07	830.63964	29.07	1238
29.08	870.63964	29.08	1219
29.09	847.63964	29.09	1250

29.1	880.63964	29.1	1280
29.11	877.63964	29.11	1263
29.12	924.63964	29.12	1261
29.13	932.63964	29.13	1219
29.14	906.63964	29.14	1280
29.15	940.63964	29.15	1266
29.16	931.63964	29.16	1264
29.17	999.63964	29.17	1235
29.18	985.63964	29.18	1233
29.19	997.63964	29.19	1268
29.2	999.63964	29.2	1281
29.21	960.63964	29.21	1301
29.22	1021.63964	29.22	1299
29.23	1017.63964	29.23	1313
29.24	988.63964	29.24	1225
29.25	936.63964	29.25	1272
29.26	1009.63964	29.26	1252
29.27	968.63964	29.27	1221
29.28	986.63964	29.28	1262
29.29	1044.63964	29.29	1242
29.3	957.63964	29.3	1281
29.31	971.63964	29.31	1298

29.32	1023.63964	29.32	1294
29.33	1003.63964	29.33	1305
29.34	992.63964	29.34	1324
29.35	971.63964	29.35	1292
29.36	976.63964	29.36	1310
29.37	988.63964	29.37	1281
29.38	994.63964	29.38	1276
29.39	1017.63964	29.39	1276
29.4	960.63964	29.4	1286
29.41	974.63964	29.41	1229
29.42	1008.63964	29.42	1265
29.43	999.63964	29.43	1274
29.44	1002.63964	29.44	1265
29.45	968.63964	29.45	1246
29.46	1030.63964	29.46	1250
29.47	941.63964	29.47	1224
29.48	923.63964	29.48	1203
29.49	932.63964	29.49	1273
29.5	920.63964	29.5	1242
29.51	959.63964	29.51	1244
29.52	960.63964	29.52	1274
29.53	905.63964	29.53	1228

29.54	913.63964	29.54	1224
29.55	901.63964	29.55	1224
29.56	892.63964	29.56	1224
29.57	903.63964	29.57	1273
29.58	848.63964	29.58	1226
29.59	859.63964	29.59	1196
29.6	902.63964	29.6	1214
29.61	893.63964	29.61	1214
29.62	825.63964	29.62	1184
29.63	868.63964	29.63	1190
29.64	797.63964	29.64	1219
29.65	782.63964	29.65	1181
29.66	787.63964	29.66	1198
29.67	822.63964	29.67	1183
29.68	802.63964	29.68	1207
29.69	789.63964	29.69	1226
29.7	768.63964	29.7	1197
29.71	784.63964	29.71	1195
29.72	786.63964	29.72	1172
29.73	837.63964	29.73	1215
29.74	782.63964	29.74	1185
29.75	765.63964	29.75	1167

29.76	796.63964	29.76	1184
29.77	781.63964	29.77	1205
29.78	752.63964	29.78	1194
29.79	801.63964	29.79	1203
29.8	787.63964	29.8	1219
29.81	778.63964	29.81	1174
29.82	808.63964	29.82	1178
29.83	726.63964	29.83	1218
29.84	747.63964	29.84	1148
29.85	724.63964	29.85	1180
29.86	771.63964	29.86	1151
29.87	752.63964	29.87	1190
29.88	717.63964	29.88	1174
29.89	689.63964	29.89	1158
29.9	746.63964	29.9	1176
29.91	703.63964	29.91	1174
29.92	709.63964	29.92	1139
29.93	748.63964	29.93	1136
29.94	706.63964	29.94	1155
29.95	770.63964	29.95	1170
29.96	719.63964	29.96	1159
29.97	718.63964	29.97	1177

29.98	739.63964	29.98	1139
29.99	736.63964	29.99	1159
30	657.63964	30	1154
30.01	708.63964	30.01	1169
30.02	704.63964	30.02	1146
30.03	725.63964	30.03	1141
30.04	684.63964	30.04	1151
30.05	728.63964	30.05	1189
30.06	672.63964	30.06	1169
30.07	722.63964	30.07	1156
30.08	686.63964	30.08	1137
30.09	726.63964	30.09	1147
30.1	685.63964	30.1	1199
30.11	684.63964	30.11	1134
30.12	706.63964	30.12	1186
30.13	695.63964	30.13	1168
30.14	672.63964	30.14	1160
30.15	670.63964	30.15	1137
30.16	692.63964	30.16	1167
30.17	703.63964	30.17	1158
30.18	707.63964	30.18	1182
30.19	670.63964	30.19	1138

30.2	709.63964	30.2	1176
30.21	686.63964	30.21	1181
30.22	675.63964	30.22	1144
30.23	662.63964	30.23	1156
30.24	664.63964	30.24	1138
30.25	666.63964	30.25	1164
30.26	659.63964	30.26	1162
30.27	658.63964	30.27	1158
30.28	695.63964	30.28	1153
30.29	662.63964	30.29	1155
30.3	709.63964	30.3	1178
30.31	689.63964	30.31	1176
30.32	681.63964	30.32	1153
30.33	713.63964	30.33	1145
30.34	692.63964	30.34	1161
30.35	712.63964	30.35	1146
30.36	663.63964	30.36	1173
30.37	655.63964	30.37	1157
30.38	676.63964	30.38	1110
30.39	676.63964	30.39	1164
30.4	703.63964	30.4	1171
30.41	687.63964	30.41	1135

30.42	713.63964	30.42	1128
30.43	697.63964	30.43	1157
30.44	697.63964	30.44	1160
30.45	735.63964	30.45	1187
30.46	711.63964	30.46	1149
30.47	725.63964	30.47	1134
30.48	723.63964	30.48	1103
30.49	717.63964	30.49	1119
30.5	661.63964	30.5	1156
30.51	707.63964	30.51	1136
30.52	662.63964	30.52	1166
30.53	742.63964	30.53	1156
30.54	681.63964	30.54	1169
30.55	642.63964	30.55	1158
30.56	711.63964	30.56	1187
30.57	686.63964	30.57	1170
30.58	680.63964	30.58	1143
30.59	719.63964	30.59	1177
30.6	722.63964	30.6	1109
30.61	724.63964	30.61	1146
30.62	720.63964	30.62	1126
30.63	762.63964	30.63	1145

30.64	712.63964	30.64	1158
30.65	734.63964	30.65	1123
30.66	670.63964	30.66	1152
30.67	733.63964	30.67	1177
30.68	721.63964	30.68	1151
30.69	719.63964	30.69	1138
30.7	673.63964	30.7	1158
30.71	733.63964	30.71	1179
30.72	721.63964	30.72	1171
30.73	656.63964	30.73	1176
30.74	683.63964	30.74	1129
30.75	721.63964	30.75	1170
30.76	714.63964	30.76	1189
30.77	693.63964	30.77	1139
30.78	723.63964	30.78	1131
30.79	674.63964	30.79	1146
30.8	696.63964	30.8	1127
30.81	723.63964	30.81	1155
30.82	695.63964	30.82	1158
30.83	759.63964	30.83	1146
30.84	727.63964	30.84	1194
30.85	694.63964	30.85	1159

30.86	689.63964	30.86	1157
30.87	708.63964	30.87	1146
30.88	718.63964	30.88	1160
30.89	741.63964	30.89	1154
30.9	719.63964	30.9	1170
30.91	742.63964	30.91	1154
30.92	762.63964	30.92	1162
30.93	706.63964	30.93	1132
30.94	727.63964	30.94	1160
30.95	774.63964	30.95	1128
30.96	755.63964	30.96	1139
30.97	729.63964	30.97	1188
30.98	747.63964	30.98	1181
30.99	738.63964	30.99	1168
31	707.63964	31	1185
31.01	744.63964	31.01	1172
31.02	766.63964	31.02	1178
31.03	711.63964	31.03	1160
31.04	697.63964	31.04	1143
31.05	708.63964	31.05	1134
31.06	712.63964	31.06	1129
31.07	758.63964	31.07	1167

31.08	770.63964	31.08	1172
31.09	744.63964	31.09	1138
31.1	794.63964	31.1	1133
31.11	785.63964	31.11	1152
31.12	726.63964	31.12	1153
31.13	766.63964	31.13	1174
31.14	788.63964	31.14	1179
31.15	774.63964	31.15	1163
31.16	732.63964	31.16	1143
31.17	788.63964	31.17	1186
31.18	753.63964	31.18	1193
31.19	787.63964	31.19	1193
31.2	834.63964	31.2	1209
31.21	807.63964	31.21	1151
31.22	785.63964	31.22	1134
31.23	728.63964	31.23	1168
31.24	816.63964	31.24	1154
31.25	799.63964	31.25	1187
31.26	761.63964	31.26	1211
31.27	815.63964	31.27	1234
31.28	785.63964	31.28	1174
31.29	823.63964	31.29	1182

31.3	778.63964	31.3	1207
31.31	779.63964	31.31	1154
31.32	805.63964	31.32	1182
31.33	745.63964	31.33	1144
31.34	793.63964	31.34	1203
31.35	832.63964	31.35	1239
31.36	870.63964	31.36	1182
31.37	829.63964	31.37	1134
31.38	852.63964	31.38	1211
31.39	802.63964	31.39	1221
31.4	862.63964	31.4	1198
31.41	806.63964	31.41	1211
31.42	812.63964	31.42	1173
31.43	866.63964	31.43	1209
31.44	870.63964	31.44	1225
31.45	829.63964	31.45	1202
31.46	839.63964	31.46	1176
31.47	832.63964	31.47	1223
31.48	937.63964	31.48	1239
31.49	924.63964	31.49	1252
31.5	888.63964	31.5	1182
31.51	888.63964	31.51	1218

31.52	887.63964	31.52	1223
31.53	890.63964	31.53	1245
31.54	955.63964	31.54	1256
31.55	932.63964	31.55	1253
31.56	873.63964	31.56	1193
31.57	847.63964	31.57	1219
31.58	902.63964	31.58	1238
31.59	994.63964	31.59	1274
31.6	944.63964	31.6	1204
31.61	924.63964	31.61	1233
31.62	958.63964	31.62	1202
31.63	902.63964	31.63	1236
31.64	927.63964	31.64	1230
31.65	965.63964	31.65	1212
31.66	948.63964	31.66	1280
31.67	961.63964	31.67	1256
31.68	928.63964	31.68	1234
31.69	984.63964	31.69	1257
31.7	988.63964	31.7	1231
31.71	985.63964	31.71	1271
31.72	992.63964	31.72	1263
31.73	953.63964	31.73	1278

31.74	996.63964	31.74	1287
31.75	941.63964	31.75	1266
31.76	997.63964	31.76	1260
31.77	980.63964	31.77	1250
31.78	1060.63964	31.78	1235
31.79	986.63964	31.79	1266
31.8	1062.63964	31.8	1254
31.81	1032.63964	31.81	1258
31.82	994.63964	31.82	1219
31.83	1014.63964	31.83	1224
31.84	1070.63964	31.84	1289
31.85	1035.63964	31.85	1255
31.86	1053.63964	31.86	1246
31.87	1029.63964	31.87	1305
31.88	1058.63964	31.88	1286
31.89	1015.63964	31.89	1277
31.9	1037.63964	31.9	1270
31.91	1038.63964	31.91	1267
31.92	1052.63964	31.92	1314
31.93	1046.63964	31.93	1291
31.94	987.63964	31.94	1237
31.95	1017.63964	31.95	1268

31.96	1037.63964	31.96	1290
31.97	1089.63964	31.97	1263
31.98	1032.63964	31.98	1273
31.99	995.63964	31.99	1226
32	1032.63964	32	1259
32.01	1011.63964	32.01	1252
32.02	1022.63964	32.02	1294
32.03	992.63964	32.03	1248
32.04	978.63964	32.04	1230
32.05	1000.63964	32.05	1264
32.06	977.63964	32.06	1257
32.07	952.63964	32.07	1257
32.08	978.63964	32.08	1238
32.09	1040.63964	32.09	1212
32.1	979.63964	32.1	1224
32.11	1023.63964	32.11	1279
32.12	983.63964	32.12	1253
32.13	963.63964	32.13	1214
32.14	991.63964	32.14	1249
32.15	971.63964	32.15	1219
32.16	905.63964	32.16	1230
32.17	939.63964	32.17	1226

32.18	910.63964	32.18	1227
32.19	957.63964	32.19	1234
32.2	898.63964	32.2	1209
32.21	957.63964	32.21	1237
32.22	915.63964	32.22	1246
32.23	946.63964	32.23	1232
32.24	901.63964	32.24	1218
32.25	930.63964	32.25	1188
32.26	894.63964	32.26	1185
32.27	883.63964	32.27	1215
32.28	966.63964	32.28	1230
32.29	918.63964	32.29	1218
32.3	949.63964	32.3	1214
32.31	923.63964	32.31	1215
32.32	854.63964	32.32	1220
32.33	867.63964	32.33	1199
32.34	918.63964	32.34	1221
32.35	881.63964	32.35	1192
32.36	921.63964	32.36	1209
32.37	907.63964	32.37	1218
32.38	876.63964	32.38	1213
32.39	876.63964	32.39	1187

32.4	899.63964	32.4	1200
32.41	906.63964	32.41	1196
32.42	900.63964	32.42	1219
32.43	849.63964	32.43	1231
32.44	892.63964	32.44	1216
32.45	879.63964	32.45	1215
32.46	913.63964	32.46	1187
32.47	898.63964	32.47	1215
32.48	917.63964	32.48	1213
32.49	876.63964	32.49	1203
32.5	858.63964	32.5	1228
32.51	884.63964	32.51	1259
32.52	899.63964	32.52	1219
32.53	872.63964	32.53	1174
32.54	909.63964	32.54	1213
32.55	923.63964	32.55	1206
32.56	919.63964	32.56	1195
32.57	934.63964	32.57	1216
32.58	911.63964	32.58	1182
32.59	966.63964	32.59	1203
32.6	857.63964	32.6	1167
32.61	883.63964	32.61	1190

32.62	814.63964	32.62	1205
32.63	892.63964	32.63	1197
32.64	909.63964	32.64	1183
32.65	877.63964	32.65	1220
32.66	903.63964	32.66	1209
32.67	970.63964	32.67	1223
32.68	970.63964	32.68	1225
32.69	883.63964	32.69	1216
32.7	848.63964	32.7	1205
32.71	874.63964	32.71	1211
32.72	917.63964	32.72	1222
32.73	923.63964	32.73	1225
32.74	903.63964	32.74	1227
32.75	890.63964	32.75	1228
32.76	922.63964	32.76	1231
32.77	893.63964	32.77	1229
32.78	909.63964	32.78	1190
32.79	943.63964	32.79	1207
32.8	931.63964	32.8	1208
32.81	876.63964	32.81	1228
32.82	925.63964	32.82	1199
32.83	929.63964	32.83	1172

32.84	907.63964	32.84	1179
32.85	910.63964	32.85	1211
32.86	900.63964	32.86	1190
32.87	894.63964	32.87	1241
32.88	896.63964	32.88	1236
32.89	931.63964	32.89	1213
32.9	923.63964	32.9	1272
32.91	863.63964	32.91	1222
32.92	857.63964	32.92	1215
32.93	837.63964	32.93	1204
32.94	939.63964	32.94	1219
32.95	914.63964	32.95	1223
32.96	911.63964	32.96	1213
32.97	949.63964	32.97	1236
32.98	904.63964	32.98	1198
32.99	894.63964	32.99	1221
33	908.63964	33	1223
33.01	929.63964	33.01	1201
33.02	864.63964	33.02	1161
33.03	899.63964	33.03	1168
33.04	852.63964	33.04	1175
33.05	880.63964	33.05	1218

33.06	852.63964	33.06	1177
33.07	810.63964	33.07	1200
33.08	885.63964	33.08	1189
33.09	883.63964	33.09	1208
33.1	824.63964	33.1	1204
33.11	871.63964	33.11	1136
33.12	822.63964	33.12	1202
33.13	805.63964	33.13	1190
33.14	872.63964	33.14	1169
33.15	801.63964	33.15	1166
33.16	805.63964	33.16	1184
33.17	779.63964	33.17	1157
33.18	800.63964	33.18	1178
33.19	819.63964	33.19	1174
33.2	822.63964	33.2	1146
33.21	803.63964	33.21	1171
33.22	821.63964	33.22	1183
33.23	793.63964	33.23	1167
33.24	834.63964	33.24	1171
33.25	793.63964	33.25	1132
33.26	799.63964	33.26	1139
33.27	813.63964	33.27	1130

33.28	794.63964	33.28	1139
33.29	752.63964	33.29	1148
33.3	779.63964	33.3	1148
33.31	763.63964	33.31	1151
33.32	765.63964	33.32	1135
33.33	826.63964	33.33	1157
33.34	718.63964	33.34	1177
33.35	750.63964	33.35	1175
33.36	735.63964	33.36	1122
33.37	778.63964	33.37	1091
33.38	756.63964	33.38	1126
33.39	772.63964	33.39	1124
33.4	722.63964	33.4	1154
33.41	726.63964	33.41	1159
33.42	732.63964	33.42	1123
33.43	704.63964	33.43	1145
33.44	701.63964	33.44	1133
33.45	751.63964	33.45	1137
33.46	700.63964	33.46	1111
33.47	684.63964	33.47	1139
33.48	745.63964	33.48	1127
33.49	744.63964	33.49	1113

33.5	730.63964	33.5	1125
33.51	651.63964	33.51	1145
33.52	709.63964	33.52	1073
33.53	735.63964	33.53	1105
33.54	714.63964	33.54	1132
33.55	715.63964	33.55	1130
33.56	695.63964	33.56	1110
33.57	677.63964	33.57	1117
33.58	727.63964	33.58	1128
33.59	678.63964	33.59	1147
33.6	676.63964	33.6	1145
33.61	659.63964	33.61	1124
33.62	683.63964	33.62	1112
33.63	715.63964	33.63	1127
33.64	707.63964	33.64	1118
33.65	688.63964	33.65	1105
33.66	690.63964	33.66	1111
33.67	680.63964	33.67	1148
33.68	660.63964	33.68	1129
33.69	677.63964	33.69	1120
33.7	682.63964	33.7	1122
33.71	649.63964	33.71	1133

33.72	676.63964	33.72	1143
33.73	690.63964	33.73	1074
33.74	688.63964	33.74	1143
33.75	670.63964	33.75	1123
33.76	671.63964	33.76	1120
33.77	638.63964	33.77	1111
33.78	650.63964	33.78	1110
33.79	674.63964	33.79	1099
33.8	633.63964	33.8	1103
33.81	646.63964	33.81	1091
33.82	707.63964	33.82	1107
33.83	655.63964	33.83	1115
33.84	678.63964	33.84	1099
33.85	634.63964	33.85	1113
33.86	644.63964	33.86	1126
33.87	668.63964	33.87	1158
33.88	677.63964	33.88	1098
33.89	683.63964	33.89	1115
33.9	675.63964	33.9	1120
33.91	678.63964	33.91	1099
33.92	667.63964	33.92	1139
33.93	662.63964	33.93	1096

33.94	684.63964	33.94	1120
33.95	674.63964	33.95	1138
33.96	630.63964	33.96	1117
33.97	654.63964	33.97	1125
33.98	693.63964	33.98	1142
33.99	640.63964	33.99	1103
34	707.63964	34	1079
34.01	620.63964	34.01	1093
34.02	669.63964	34.02	1090
34.03	689.63964	34.03	1112
34.04	696.63964	34.04	1060
34.05	712.63964	34.05	1074
34.06	664.63964	34.06	1106
34.07	659.63964	34.07	1102
34.08	675.63964	34.08	1084
34.09	615.63964	34.09	1094
34.1	623.63964	34.1	1117
34.11	672.63964	34.11	1099
34.12	668.63964	34.12	1101
34.13	669.63964	34.13	1077
34.14	615.63964	34.14	1089
34.15	617.63964	34.15	1094

34.16	627.63964	34.16	1115
34.17	634.63964	34.17	1068
34.18	663.63964	34.18	1118
34.19	644.63964	34.19	1117
34.2	645.63964	34.2	1081
34.21	636.63964	34.21	1084
34.22	638.63964	34.22	1045
34.23	648.63964	34.23	1098
34.24	608.63964	34.24	1081
34.25	643.63964	34.25	1117
34.26	669.63964	34.26	1078
34.27	650.63964	34.27	1082
34.28	627.63964	34.28	1097
34.29	622.63964	34.29	1091
34.3	652.63964	34.3	1113
34.31	658.63964	34.31	1107
34.32	626.63964	34.32	1080
34.33	641.63964	34.33	1089
34.34	642.63964	34.34	1107
34.35	584.63964	34.35	1094
34.36	639.63964	34.36	1091
34.37	603.63964	34.37	1069

34.38	647.63964	34.38	1089
34.39	631.63964	34.39	1100
34.4	669.63964	34.4	1070
34.41	669.63964	34.41	1084
34.42	601.63964	34.42	1096
34.43	663.63964	34.43	1129
34.44	677.63964	34.44	1082
34.45	686.63964	34.45	1106
34.46	605.63964	34.46	1081
34.47	585.63964	34.47	1082
34.48	619.63964	34.48	1076
34.49	635.63964	34.49	1089
34.5	641.63964	34.5	1084
34.51	636.63964	34.51	1090
34.52	643.63964	34.52	1079
34.53	620.63964	34.53	1095
34.54	572.63964	34.54	1065
34.55	645.63964	34.55	1099
34.56	624.63964	34.56	1089
34.57	652.63964	34.57	1081
34.58	663.63964	34.58	1065
34.59	601.63964	34.59	1080

34.6	623.63964	34.6	1096
34.61	613.63964	34.61	1070
34.62	622.63964	34.62	1106
34.63	661.63964	34.63	1102
34.64	647.63964	34.64	1086
34.65	620.63964	34.65	1083
34.66	630.63964	34.66	1092
34.67	630.63964	34.67	1071
34.68	639.63964	34.68	1126
34.69	651.63964	34.69	1083
34.7	650.63964	34.7	1124
34.71	622.63964	34.71	1098
34.72	649.63964	34.72	1090
34.73	647.63964	34.73	1073
34.74	631.63964	34.74	1070
34.75	629.63964	34.75	1111
34.76	614.63964	34.76	1094
34.77	619.63964	34.77	1102
34.78	639.63964	34.78	1111
34.79	639.63964	34.79	1142
34.8	621.63964	34.8	1065
34.81	629.63964	34.81	1081

34.82	672.63964	34.82	1101
34.83	629.63964	34.83	1100
34.84	615.63964	34.84	1072
34.85	661.63964	34.85	1071
34.86	588.63964	34.86	1071
34.87	607.63964	34.87	1072
34.88	643.63964	34.88	1092
34.89	570.63964	34.89	1107
34.9	648.63964	34.9	1091
34.91	597.63964	34.91	1076
34.92	604.63964	34.92	1111
34.93	623.63964	34.93	1065
34.94	621.63964	34.94	1079
34.95	667.63964	34.95	1046
34.96	611.63964	34.96	1068
34.97	644.63964	34.97	1051
34.98	631.63964	34.98	1074
34.99	600.63964	34.99	1082
35	690.63964	35	1077
35.01	671.63964	35.01	1075
35.02	622.63964	35.02	1080
35.03	604.63964	35.03	1087

35.04	604.63964	35.04	1090
35.05	622.63964	35.05	1089
35.06	637.63964	35.06	1094
35.07	631.63964	35.07	1083
35.08	647.63964	35.08	1061
35.09	650.63964	35.09	1051
35.1	623.63964	35.1	1105
35.11	631.63964	35.11	1057
35.12	611.63964	35.12	1053
35.13	618.63964	35.13	1071
35.14	636.63964	35.14	1075
35.15	607.63964	35.15	1093
35.16	635.63964	35.16	1089
35.17	634.63964	35.17	1055
35.18	636.63964	35.18	1073
35.19	608.63964	35.19	1076
35.2	590.63964	35.2	1092
35.21	629.63964	35.21	1075
35.22	598.63964	35.22	1071
35.23	620.63964	35.23	1077
35.24	626.63964	35.24	1069
35.25	624.63964	35.25	1064

35.26	598.63964	35.26	1084
35.27	607.63964	35.27	1057
35.28	617.63964	35.28	1075
35.29	617.63964	35.29	1056
35.3	625.63964	35.3	1093
35.31	619.63964	35.31	1086
35.32	633.63964	35.32	1065
35.33	593.63964	35.33	1078
35.34	654.63964	35.34	1064
35.35	638.63964	35.35	1054
35.36	623.63964	35.36	1066
35.37	618.63964	35.37	1073
35.38	633.63964	35.38	1070
35.39	619.63964	35.39	1077
35.4	613.63964	35.4	1085
35.41	675.63964	35.41	1050
35.42	609.63964	35.42	1078
35.43	594.63964	35.43	1068
35.44	636.63964	35.44	1080
35.45	599.63964	35.45	1063
35.46	626.63964	35.46	1053
35.47	625.63964	35.47	1127

35.48	634.63964	35.48	1060
35.49	629.63964	35.49	1079
35.5	600.63964	35.5	1084
35.51	561.63964	35.51	1116
35.52	634.63964	35.52	1074
35.53	595.63964	35.53	1073
35.54	608.63964	35.54	1119
35.55	606.63964	35.55	1095
35.56	621.63964	35.56	1094
35.57	660.63964	35.57	1068
35.58	606.63964	35.58	1066
35.59	648.63964	35.59	1061
35.6	604.63964	35.6	1093
35.61	613.63964	35.61	1084
35.62	654.63964	35.62	1075
35.63	611.63964	35.63	1052
35.64	632.63964	35.64	1066
35.65	585.63964	35.65	1058
35.66	608.63964	35.66	1038
35.67	603.63964	35.67	1093
35.68	599.63964	35.68	1074
35.69	604.63964	35.69	1108

35.7	641.63964	35.7	1073
35.71	655.63964	35.71	1079
35.72	642.63964	35.72	1051
35.73	631.63964	35.73	1039
35.74	599.63964	35.74	1097
35.75	603.63964	35.75	1113
35.76	609.63964	35.76	1086
35.77	607.63964	35.77	1068
35.78	599.63964	35.78	1059
35.79	592.63964	35.79	1092
35.8	616.63964	35.8	1064
35.81	586.63964	35.81	1063
35.82	600.63964	35.82	1063
35.83	617.63964	35.83	1070
35.84	601.63964	35.84	1055
35.85	604.63964	35.85	1076
35.86	591.63964	35.86	1054
35.87	585.63964	35.87	1057
35.88	598.63964	35.88	1110
35.89	620.63964	35.89	1033
35.9	607.63964	35.9	1065
35.91	635.63964	35.91	1119

35.92	642.63964	35.92	1053
35.93	623.63964	35.93	1092
35.94	660.63964	35.94	1095
35.95	618.63964	35.95	1028
35.96	582.63964	35.96	1069
35.97	631.63964	35.97	1067
35.98	600.63964	35.98	1075
35.99	637.63964	35.99	1066
36	583.63964	36	1079
36.01	587.63964	36.01	1017
36.02	613.63964	36.02	1055
36.03	626.63964	36.03	1087
36.04	601.63964	36.04	1102
36.05	585.63964	36.05	1064
36.06	555.63964	36.06	1069
36.07	595.63964	36.07	1044
36.08	557.63964	36.08	1094
36.09	562.63964	36.09	1095
36.1	581.63964	36.1	1078
36.11	612.63964	36.11	1092
36.12	625.63964	36.12	1090
36.13	597.63964	36.13	1055

36.14	601.63964	36.14	1066
36.15	580.63964	36.15	1095
36.16	615.63964	36.16	1053
36.17	632.63964	36.17	1090
36.18	605.63964	36.18	1050
36.19	622.63964	36.19	1104
36.2	630.63964	36.2	1069
36.21	625.63964	36.21	1061
36.22	575.63964	36.22	1095
36.23	579.63964	36.23	1085
36.24	619.63964	36.24	1071
36.25	657.63964	36.25	1085
36.26	633.63964	36.26	1058
36.27	603.63964	36.27	1071
36.28	609.63964	36.28	1040
36.29	594.63964	36.29	1068
36.3	641.63964	36.3	1056
36.31	589.63964	36.31	1106
36.32	632.63964	36.32	1055
36.33	583.63964	36.33	1082
36.34	594.63964	36.34	1049
36.35	622.63964	36.35	1055

36.36	610.63964	36.36	1046
36.37	641.63964	36.37	1029
36.38	631.63964	36.38	1054
36.39	618.63964	36.39	1041
36.4	631.63964	36.4	1046
36.41	600.63964	36.41	1056
36.42	571.63964	36.42	1053
36.43	599.63964	36.43	1061
36.44	606.63964	36.44	1093
36.45	566.63964	36.45	1052
36.46	613.63964	36.46	1059
36.47	612.63964	36.47	1070
36.48	614.63964	36.48	1071
36.49	565.63964	36.49	1087
36.5	597.63964	36.5	1075
36.51	581.63964	36.51	1040
36.52	594.63964	36.52	1051
36.53	605.63964	36.53	1065
36.54	584.63964	36.54	1061
36.55	598.63964	36.55	1091
36.56	586.63964	36.56	1052
36.57	546.63964	36.57	1043

36.58	584.63964	36.58	1100
36.59	579.63964	36.59	1066
36.6	604.63964	36.6	1059
36.61	597.63964	36.61	1098
36.62	627.63964	36.62	1056
36.63	591.63964	36.63	1073
36.64	588.63964	36.64	1071
36.65	620.63964	36.65	1077
36.66	579.63964	36.66	1080
36.67	573.63964	36.67	1099
36.68	635.63964	36.68	1064
36.69	570.63964	36.69	1031
36.7	612.63964	36.7	1044
36.71	594.63964	36.71	1058
36.72	574.63964	36.72	1066
36.73	645.63964	36.73	1082
36.74	585.63964	36.74	1084
36.75	606.63964	36.75	1051
36.76	595.63964	36.76	1038
36.77	574.63964	36.77	1077
36.78	568.63964	36.78	1038
36.79	627.63964	36.79	1054

36.8	579.63964	36.8	1084
36.81	588.63964	36.81	1087
36.82	644.63964	36.82	1082
36.83	639.63964	36.83	1065
36.84	608.63964	36.84	1059
36.85	570.63964	36.85	1033
36.86	624.63964	36.86	1075
36.87	565.63964	36.87	1082
36.88	661.63964	36.88	1069
36.89	611.63964	36.89	1033
36.9	652.63964	36.9	1066
36.91	641.63964	36.91	1035
36.92	597.63964	36.92	1056
36.93	632.63964	36.93	1067
36.94	629.63964	36.94	1070
36.95	608.63964	36.95	1046
36.96	642.63964	36.96	1059
36.97	620.63964	36.97	1071
36.98	611.63964	36.98	1077
36.99	582.63964	36.99	1060
37	597.63964	37	1077
37.01	585.63964	37.01	1063

37.02	597.63964	37.02	1043
37.03	591.63964	37.03	1056
37.04	583.63964	37.04	1049
37.05	618.63964	37.05	1053
37.06	595.63964	37.06	1042
37.07	626.63964	37.07	1020
37.08	570.63964	37.08	1057
37.09	603.63964	37.09	1060
37.1	614.63964	37.1	1093
37.11	612.63964	37.11	1072
37.12	616.63964	37.12	1053
37.13	593.63964	37.13	1052
37.14	566.63964	37.14	1072
37.15	579.63964	37.15	1092
37.16	635.63964	37.16	1087
37.17	612.63964	37.17	1043
37.18	598.63964	37.18	1071
37.19	554.63964	37.19	1040
37.2	609.63964	37.2	1011
37.21	585.63964	37.21	1039
37.22	575.63964	37.22	1052
37.23	605.63964	37.23	1070

37.24	585.63964	37.24	1059
37.25	620.63964	37.25	1061
37.26	594.63964	37.26	1079
37.27	606.63964	37.27	1080
37.28	595.63964	37.28	1074
37.29	616.63964	37.29	1039
37.3	571.63964	37.3	1074
37.31	559.63964	37.31	1044
37.32	586.63964	37.32	1097
37.33	593.63964	37.33	1055
37.34	570.63964	37.34	1060
37.35	585.63964	37.35	1052
37.36	591.63964	37.36	1070
37.37	605.63964	37.37	1072
37.38	575.63964	37.38	1047
37.39	604.63964	37.39	1073
37.4	614.63964	37.4	1046
37.41	621.63964	37.41	1043
37.42	641.63964	37.42	1057
37.43	612.63964	37.43	1040
37.44	537.63964	37.44	1075
37.45	596.63964	37.45	1074

37.46	618.63964	37.46	1061
37.47	592.63964	37.47	1073
37.48	604.63964	37.48	1074
37.49	630.63964	37.49	1074
37.5	611.63964	37.5	1058
37.51	618.63964	37.51	1073
37.52	678.63964	37.52	1067
37.53	614.63964	37.53	1091
37.54	584.63964	37.54	1080
37.55	562.63964	37.55	1047
37.56	580.63964	37.56	1047
37.57	560.63964	37.57	1064
37.58	577.63964	37.58	1086
37.59	583.63964	37.59	1077
37.6	559.63964	37.6	1017
37.61	562.63964	37.61	1090
37.62	572.63964	37.62	1086
37.63	592.63964	37.63	1072
37.64	626.63964	37.64	1072
37.65	599.63964	37.65	1069
37.66	606.63964	37.66	1058
37.67	592.63964	37.67	1051

37.68	596.63964	37.68	1055
37.69	623.63964	37.69	1066
37.7	615.63964	37.7	1015
37.71	640.63964	37.71	1067
37.72	594.63964	37.72	1072
37.73	616.63964	37.73	1065
37.74	587.63964	37.74	1066
37.75	634.63964	37.75	1049
37.76	597.63964	37.76	1021
37.77	618.63964	37.77	1060
37.78	597.63964	37.78	1061
37.79	587.63964	37.79	1064
37.8	586.63964	37.8	1034
37.81	597.63964	37.81	1078
37.82	570.63964	37.82	1064
37.83	598.63964	37.83	1078
37.84	609.63964	37.84	1059
37.85	597.63964	37.85	1047
37.86	582.63964	37.86	1076
37.87	604.63964	37.87	1022
37.88	615.63964	37.88	1044
37.89	599.63964	37.89	1070

37.9	609.63964	37.9	1043
37.91	597.63964	37.91	1031
37.92	613.63964	37.92	1048
37.93	627.63964	37.93	1072
37.94	625.63964	37.94	1075
37.95	609.63964	37.95	1080
37.96	603.63964	37.96	1068
37.97	598.63964	37.97	1033
37.98	583.63964	37.98	1028
37.99	579.63964	37.99	1059
38	617.63964	38	1068
38.01	596.63964	38.01	1057
38.02	615.63964	38.02	1050
38.03	600.63964	38.03	1066
38.04	583.63964	38.04	1041
38.05	589.63964	38.05	1052
38.06	547.63964	38.06	1056
38.07	601.63964	38.07	1077
38.08	555.63964	38.08	1071
38.09	590.63964	38.09	1046
38.1	581.63964	38.1	1078
38.11	630.63964	38.11	1048

38.12	581.63964	38.12	1052
38.13	578.63964	38.13	1045
38.14	591.63964	38.14	1038
38.15	590.63964	38.15	1051
38.16	624.63964	38.16	1060
38.17	626.63964	38.17	1059
38.18	583.63964	38.18	1039
38.19	586.63964	38.19	1056
38.2	632.63964	38.2	1065
38.21	651.63964	38.21	1052
38.22	623.63964	38.22	1038
38.23	608.63964	38.23	1068
38.24	629.63964	38.24	1035
38.25	567.63964	38.25	1015
38.26	580.63964	38.26	1049
38.27	624.63964	38.27	1058
38.28	590.63964	38.28	1054
38.29	580.63964	38.29	1065
38.3	596.63964	38.3	1028
38.31	611.63964	38.31	1075
38.32	636.63964	38.32	1099
38.33	603.63964	38.33	1073

38.34	615.63964	38.34	1041
38.35	595.63964	38.35	1058
38.36	607.63964	38.36	1059
38.37	589.63964	38.37	1043
38.38	601.63964	38.38	1050
38.39	616.63964	38.39	1057
38.4	584.63964	38.4	1053
38.41	618.63964	38.41	1047
38.42	614.63964	38.42	1089
38.43	627.63964	38.43	1055
38.44	554.63964	38.44	1051
38.45	578.63964	38.45	1069
38.46	615.63964	38.46	1064
38.47	609.63964	38.47	1053
38.48	611.63964	38.48	1019
38.49	620.63964	38.49	1058
38.5	603.63964	38.5	1064
38.51	569.63964	38.51	1052
38.52	586.63964	38.52	1044
38.53	593.63964	38.53	1066
38.54	559.63964	38.54	1059
38.55	582.63964	38.55	1044

38.56	588.63964	38.56	1035
38.57	613.63964	38.57	1045
38.58	609.63964	38.58	1025
38.59	584.63964	38.59	1050
38.6	599.63964	38.6	1080
38.61	567.63964	38.61	1046
38.62	613.63964	38.62	1037
38.63	591.63964	38.63	1051
38.64	621.63964	38.64	1042
38.65	599.63964	38.65	1068
38.66	611.63964	38.66	1065
38.67	597.63964	38.67	1050
38.68	598.63964	38.68	1078
38.69	568.63964	38.69	1052
38.7	599.63964	38.7	1058
38.71	573.63964	38.71	1050
38.72	594.63964	38.72	1058
38.73	606.63964	38.73	1096
38.74	573.63964	38.74	1073
38.75	639.63964	38.75	1063
38.76	603.63964	38.76	1064
38.77	615.63964	38.77	1071

38.78	615.63964	38.78	1081
38.79	592.63964	38.79	1083
38.8	604.63964	38.8	1058
38.81	599.63964	38.81	1041
38.82	572.63964	38.82	1049
38.83	636.63964	38.83	1046
38.84	607.63964	38.84	1063
38.85	615.63964	38.85	1034
38.86	584.63964	38.86	1057
38.87	595.63964	38.87	1082
38.88	603.63964	38.88	1069
38.89	601.63964	38.89	1063
38.9	614.63964	38.9	1073
38.91	620.63964	38.91	1070
38.92	627.63964	38.92	1058
38.93	574.63964	38.93	1064
38.94	642.63964	38.94	1054
38.95	622.63964	38.95	1072
38.96	618.63964	38.96	1056
38.97	612.63964	38.97	1056
38.98	627.63964	38.98	1085
38.99	559.63964	38.99	1033

39	584.63964	39	1056
39.01	599.63964	39.01	1037
39.02	622.63964	39.02	1052
39.03	594.63964	39.03	1042
39.04	613.63964	39.04	1046
39.05	629.63964	39.05	1045
39.06	629.63964	39.06	1046
39.07	582.63964	39.07	1050
39.08	578.63964	39.08	1044
39.09	583.63964	39.09	1037
39.1	572.63964	39.1	1074
39.11	624.63964	39.11	1041
39.12	554.63964	39.12	1047
39.13	572.63964	39.13	1031
39.14	600.63964	39.14	1048
39.15	658.63964	39.15	1086
39.16	634.63964	39.16	1049
39.17	637.63964	39.17	1031
39.18	620.63964	39.18	1034
39.19	591.63964	39.19	1066
39.2	624.63964	39.2	1080
39.21	583.63964	39.21	1069

39.22	615.63964	39.22	1053
39.23	620.63964	39.23	1047
39.24	575.63964	39.24	1071
39.25	626.63964	39.25	1034
39.26	584.63964	39.26	1066
39.27	538.63964	39.27	1041
39.28	554.63964	39.28	1053
39.29	590.63964	39.29	1025
39.3	575.63964	39.3	1065
39.31	604.63964	39.31	1054
39.32	590.63964	39.32	1063
39.33	609.63964	39.33	1077
39.34	569.63964	39.34	1046
39.35	581.63964	39.35	1050
39.36	620.63964	39.36	1032
39.37	567.63964	39.37	1028
39.38	584.63964	39.38	1042
39.39	601.63964	39.39	1049
39.4	586.63964	39.4	1019
39.41	561.63964	39.41	1067
39.42	556.63964	39.42	1058
39.43	545.63964	39.43	1050

39.44	595.63964	39.44	1054
39.45	566.63964	39.45	1022
39.46	591.63964	39.46	1031
39.47	579.63964	39.47	1049
39.48	549.63964	39.48	1029
39.49	563.63964	39.49	1055
39.5	581.63964	39.5	1030
39.51	587.63964	39.51	1035
39.52	547.63964	39.52	1022
39.53	587.63964	39.53	1063
39.54	580.63964	39.54	1014
39.55	558.63964	39.55	1060
39.56	603.63964	39.56	1051
39.57	595.63964	39.57	1055
39.58	589.63964	39.58	1052
39.59	541.63964	39.59	1053
39.6	567.63964	39.6	1037
39.61	606.63964	39.61	1069
39.62	599.63964	39.62	1044
39.63	553.63964	39.63	1067
39.64	579.63964	39.64	1024
39.65	610.63964	39.65	1053

39.66	552.63964	39.66	1053
39.67	587.63964	39.67	1061
39.68	558.63964	39.68	1040
39.69	531.63964	39.69	1038
39.7	542.63964	39.7	1033
39.71	557.63964	39.71	1060
39.72	583.63964	39.72	1078
39.73	566.63964	39.73	1035
39.74	589.63964	39.74	1028
39.75	568.63964	39.75	1079
39.76	539.63964	39.76	1076
39.77	546.63964	39.77	1086
39.78	577.63964	39.78	1079
39.79	591.63964	39.79	1023
39.8	547.63964	39.8	1055
39.81	551.63964	39.81	1056
39.82	592.63964	39.82	1023
39.83	552.63964	39.83	1047
39.84	598.63964	39.84	1048
39.85	562.63964	39.85	1022
39.86	621.63964	39.86	1048
39.87	583.63964	39.87	1059

39.88	548.63964	39.88	1071
39.89	598.63964	39.89	1040
39.9	585.63964	39.9	1054
39.91	555.63964	39.91	1065
39.92	604.63964	39.92	1038
39.93	540.63964	39.93	1016
39.94	566.63964	39.94	1060
39.95	557.63964	39.95	1060
39.96	572.63964	39.96	1065
39.97	583.63964	39.97	1055
39.98	591.63964	39.98	1064
39.99	551.63964	39.99	1076
40	578.63964	40	1066
40.01	555.63964	40.01	1077
40.02	544.63964	40.02	1033
40.03	552.63964	40.03	1058
40.04	552.63964	40.04	1043
40.05	570.63964	40.05	1017
40.06	574.63964	40.06	1046
40.07	561.63964	40.07	1063
40.08	580.63964	40.08	1022
40.09	578.63964	40.09	1040

40.1	596.63964	40.1	1076
40.11	505.63964	40.11	1066
40.12	590.63964	40.12	1021
40.13	563.63964	40.13	1053
40.14	585.63964	40.14	1029
40.15	551.63964	40.15	1031
40.16	580.63964	40.16	1011
40.17	599.63964	40.17	1027
40.18	564.63964	40.18	1042
40.19	540.63964	40.19	1040
40.2	530.63964	40.2	1070
40.21	549.63964	40.21	1061
40.22	601.63964	40.22	1084
40.23	566.63964	40.23	1045
40.24	558.63964	40.24	1078
40.25	557.63964	40.25	1032
40.26	536.63964	40.26	1054
40.27	583.63964	40.27	1034
40.28	562.63964	40.28	1037
40.29	552.63964	40.29	1046
40.3	584.63964	40.3	1044
40.31	571.63964	40.31	1048

40.32	572.63964	40.32	1060
40.33	543.63964	40.33	1051
40.34	580.63964	40.34	1048
40.35	595.63964	40.35	1052
40.36	556.63964	40.36	1046
40.37	564.63964	40.37	1019
40.38	580.63964	40.38	1034
40.39	591.63964	40.39	1057
40.4	587.63964	40.4	1048
40.41	624.63964	40.41	1016
40.42	587.63964	40.42	1056
40.43	582.63964	40.43	1019
40.44	558.63964	40.44	1083
40.45	527.63964	40.45	1060
40.46	555.63964	40.46	1071
40.47	580.63964	40.47	1022
40.48	599.63964	40.48	1033
40.49	584.63964	40.49	1042
40.5	541.63964	40.5	1044
40.51	579.63964	40.51	1041
40.52	564.63964	40.52	1039
40.53	539.63964	40.53	1033

40.54	579.63964	40.54	1055
40.55	587.63964	40.55	1050
40.56	613.63964	40.56	1035
40.57	585.63964	40.57	1019
40.58	603.63964	40.58	1061
40.59	551.63964	40.59	1055
40.6	552.63964	40.6	1041
40.61	590.63964	40.61	1039
40.62	609.63964	40.62	1041
40.63	604.63964	40.63	1041
40.64	581.63964	40.64	1049
40.65	603.63964	40.65	1043
40.66	593.63964	40.66	1055
40.67	610.63964	40.67	1034
40.68	583.63964	40.68	1054
40.69	594.63964	40.69	1053
40.7	600.63964	40.7	1023
40.71	625.63964	40.71	1030
40.72	572.63964	40.72	1064
40.73	583.63964	40.73	1048
40.74	622.63964	40.74	1063
40.75	554.63964	40.75	1058

40.76	552.63964	40.76	1051
40.77	564.63964	40.77	1070
40.78	552.63964	40.78	1038
40.79	569.63964	40.79	1046
40.8	564.63964	40.8	1047
40.81	616.63964	40.81	1066
40.82	573.63964	40.82	1024
40.83	577.63964	40.83	1031
40.84	545.63964	40.84	1026
40.85	590.63964	40.85	1051
40.86	569.63964	40.86	1023
40.87	579.63964	40.87	1014
40.88	572.63964	40.88	1020
40.89	598.63964	40.89	1035
40.9	572.63964	40.9	1028
40.91	573.63964	40.91	1019
40.92	568.63964	40.92	1078
40.93	569.63964	40.93	1025
40.94	584.63964	40.94	1035
40.95	570.63964	40.95	1043
40.96	591.63964	40.96	1047
40.97	564.63964	40.97	1023

40.98	559.63964	40.98	1047
40.99	553.63964	40.99	1028
41	579.63964	41	1040
41.01	582.63964	41.01	1045
41.02	586.63964	41.02	1044
41.03	588.63964	41.03	1080
41.04	559.63964	41.04	1066
41.05	558.63964	41.05	1022
41.06	585.63964	41.06	1017
41.07	573.63964	41.07	1029
41.08	582.63964	41.08	1031
41.09	579.63964	41.09	1039
41.1	521.63964	41.1	1040
41.11	531.63964	41.11	1050
41.12	551.63964	41.12	1041
41.13	564.63964	41.13	1022
41.14	593.63964	41.14	1041
41.15	560.63964	41.15	1028
41.16	600.63964	41.16	1043
41.17	589.63964	41.17	1028
41.18	570.63964	41.18	1018
41.19	534.63964	41.19	1051

41.2	541.63964	41.2	1027
41.21	526.63964	41.21	1028
41.22	567.63964	41.22	1024
41.23	551.63964	41.23	1038
41.24	566.63964	41.24	1048
41.25	563.63964	41.25	1041
41.26	579.63964	41.26	1028
41.27	555.63964	41.27	1026
41.28	532.63964	41.28	1042
41.29	551.63964	41.29	1030
41.3	543.63964	41.3	1038
41.31	595.63964	41.31	1052
41.32	586.63964	41.32	1019
41.33	567.63964	41.33	1013
41.34	569.63964	41.34	1048
41.35	568.63964	41.35	1041
41.36	575.63964	41.36	1055
41.37	573.63964	41.37	1060
41.38	586.63964	41.38	1008
41.39	577.63964	41.39	1040
41.4	592.63964	41.4	1063
41.41	539.63964	41.41	1027

41.42	573.63964	41.42	1043
41.43	589.63964	41.43	1008
41.44	579.63964	41.44	1054
41.45	572.63964	41.45	1043
41.46	536.63964	41.46	993
41.47	604.63964	41.47	1042
41.48	580.63964	41.48	1039
41.49	577.63964	41.49	1042
41.5	584.63964	41.5	1061
41.51	553.63964	41.51	1011
41.52	541.63964	41.52	1016
41.53	611.63964	41.53	1020
41.54	571.63964	41.54	1044
41.55	566.63964	41.55	1064
41.56	588.63964	41.56	1045
41.57	552.63964	41.57	1051
41.58	569.63964	41.58	1077
41.59	587.63964	41.59	1035
41.6	567.63964	41.6	1048
41.61	561.63964	41.61	1027
41.62	561.63964	41.62	1025
41.63	561.63964	41.63	1041

41.64	581.63964	41.64	1055
41.65	565.63964	41.65	1057
41.66	582.63964	41.66	1033
41.67	570.63964	41.67	1023
41.68	543.63964	41.68	1068
41.69	548.63964	41.69	1052
41.7	538.63964	41.7	1016
41.71	559.63964	41.71	1045
41.72	600.63964	41.72	1052
41.73	579.63964	41.73	1037
41.74	624.63964	41.74	1044
41.75	560.63964	41.75	1059
41.76	554.63964	41.76	1050
41.77	537.63964	41.77	1051
41.78	540.63964	41.78	1038
41.79	588.63964	41.79	1030
41.8	585.63964	41.8	1039
41.81	553.63964	41.81	1058
41.82	579.63964	41.82	1022
41.83	553.63964	41.83	1032
41.84	556.63964	41.84	1046
41.85	575.63964	41.85	1024

41.86	563.63964	41.86	1051
41.87	548.63964	41.87	1052
41.88	532.63964	41.88	1050
41.89	523.63964	41.89	1021
41.9	560.63964	41.9	1033
41.91	562.63964	41.91	1051
41.92	543.63964	41.92	1055
41.93	510.63964	41.93	1029
41.94	595.63964	41.94	1014
41.95	616.63964	41.95	1013
41.96	601.63964	41.96	1040
41.97	575.63964	41.97	1025
41.98	569.63964	41.98	1065
41.99	559.63964	41.99	1078
42	595.63964	42	1048
42.01	617.63964	42.01	1042
42.02	574.63964	42.02	1043
42.03	594.63964	42.03	1031
42.04	521.63964	42.04	1058
42.05	566.63964	42.05	1042
42.06	583.63964	42.06	1019
42.07	580.63964	42.07	1051

42.08	633.63964	42.08	1007
42.09	552.63964	42.09	1056
42.1	567.63964	42.1	1034
42.11	566.63964	42.11	1049
42.12	526.63964	42.12	1041
42.13	541.63964	42.13	1031
42.14	558.63964	42.14	1046
42.15	584.63964	42.15	1059
42.16	584.63964	42.16	1039
42.17	552.63964	42.17	1038
42.18	610.63964	42.18	1030
42.19	525.63964	42.19	1044
42.2	542.63964	42.2	1050
42.21	603.63964	42.21	1022
42.22	579.63964	42.22	1038
42.23	588.63964	42.23	1020
42.24	558.63964	42.24	1077
42.25	606.63964	42.25	1045
42.26	566.63964	42.26	1012
42.27	554.63964	42.27	1033
42.28	597.63964	42.28	1045
42.29	563.63964	42.29	1034

42.3	593.63964	42.3	1046
42.31	579.63964	42.31	1050
42.32	540.63964	42.32	1048
42.33	585.63964	42.33	1027
42.34	623.63964	42.34	1026
42.35	549.63964	42.35	1058
42.36	579.63964	42.36	1017
42.37	540.63964	42.37	1038
42.38	572.63964	42.38	1024
42.39	565.63964	42.39	1069
42.4	518.63964	42.4	1077
42.41	582.63964	42.41	1058
42.42	581.63964	42.42	1046
42.43	519.63964	42.43	1005
42.44	542.63964	42.44	1063
42.45	604.63964	42.45	1013
42.46	586.63964	42.46	1079
42.47	561.63964	42.47	1056
42.48	570.63964	42.48	1064
42.49	595.63964	42.49	1047
42.5	563.63964	42.5	1032
42.51	588.63964	42.51	1077

42.52	575.63964	42.52	1054
42.53	548.63964	42.53	1046
42.54	546.63964	42.54	1025
42.55	544.63964	42.55	1054
42.56	566.63964	42.56	1044
42.57	565.63964	42.57	1056
42.58	580.63964	42.58	1064
42.59	536.63964	42.59	1047
42.6	587.63964	42.6	1022
42.61	555.63964	42.61	1049
42.62	594.63964	42.62	1037
42.63	548.63964	42.63	1033
42.64	571.63964	42.64	1070
42.65	590.63964	42.65	1070
42.66	563.63964	42.66	1042
42.67	568.63964	42.67	1059
42.68	563.63964	42.68	1056
42.69	574.63964	42.69	1039
42.7	584.63964	42.7	1038
42.71	560.63964	42.71	1075
42.72	577.63964	42.72	1040
42.73	583.63964	42.73	1044

42.74	541.63964	42.74	1041
42.75	567.63964	42.75	1035
42.76	589.63964	42.76	1054
42.77	557.63964	42.77	1050
42.78	560.63964	42.78	1065
42.79	554.63964	42.79	1054
42.8	579.63964	42.8	1066
42.81	581.63964	42.81	1028
42.82	567.63964	42.82	1013
42.83	606.63964	42.83	1026
42.84	583.63964	42.84	1069
42.85	586.63964	42.85	1053
42.86	572.63964	42.86	1019
42.87	606.63964	42.87	1029
42.88	547.63964	42.88	1054
42.89	578.63964	42.89	1051
42.9	582.63964	42.9	1046
42.91	569.63964	42.91	1052
42.92	553.63964	42.92	1052
42.93	567.63964	42.93	1070
42.94	568.63964	42.94	1060
42.95	582.63964	42.95	1014

42.96	617.63964	42.96	1044
42.97	605.63964	42.97	1061
42.98	579.63964	42.98	1080
42.99	601.63964	42.99	1047
43	604.63964	43	1058
43.01	567.63964	43.01	1074
43.02	589.63964	43.02	1023
43.03	569.63964	43.03	1038
43.04	616.63964	43.04	1064
43.05	606.63964	43.05	1049
43.06	597.63964	43.06	1040
43.07	589.63964	43.07	1051
43.08	627.63964	43.08	1018
43.09	605.63964	43.09	1029
43.1	612.63964	43.1	1051
43.11	595.63964	43.11	1054
43.12	585.63964	43.12	1061
43.13	539.63964	43.13	1043
43.14	562.63964	43.14	1026
43.15	603.63964	43.15	1033
43.16	561.63964	43.16	1017
43.17	601.63964	43.17	1024

43.18	611.63964	43.18	1049
43.19	596.63964	43.19	1043
43.2	619.63964	43.2	1039
43.21	570.63964	43.21	1035
43.22	614.63964	43.22	1001
43.23	590.63964	43.23	1070
43.24	552.63964	43.24	1074
43.25	609.63964	43.25	1067
43.26	562.63964	43.26	1038
43.27	595.63964	43.27	1050
43.28	586.63964	43.28	1031
43.29	507.63964	43.29	1039
43.3	607.63964	43.3	1049
43.31	615.63964	43.31	1057
43.32	597.63964	43.32	1021
43.33	546.63964	43.33	1050
43.34	560.63964	43.34	1038
43.35	558.63964	43.35	1082
43.36	580.63964	43.36	1076
43.37	600.63964	43.37	1022
43.38	573.63964	43.38	1058
43.39	554.63964	43.39	1022

43.4	625.63964	43.4	1048
43.41	584.63964	43.41	1035
43.42	563.63964	43.42	1008
43.43	573.63964	43.43	1048
43.44	577.63964	43.44	1020
43.45	553.63964	43.45	1064
43.46	585.63964	43.46	1051
43.47	594.63964	43.47	1057
43.48	591.63964	43.48	1066
43.49	629.63964	43.49	1031
43.5	592.63964	43.5	1031
43.51	637.63964	43.51	1043
43.52	563.63964	43.52	1049
43.53	587.63964	43.53	1061
43.54	544.63964	43.54	1050
43.55	583.63964	43.55	1021
43.56	569.63964	43.56	1056
43.57	539.63964	43.57	1056
43.58	590.63964	43.58	1044
43.59	599.63964	43.59	1037
43.6	610.63964	43.6	1015
43.61	571.63964	43.61	1026

43.62	580.63964	43.62	1050
43.63	584.63964	43.63	1041
43.64	569.63964	43.64	1061
43.65	573.63964	43.65	1033
43.66	589.63964	43.66	1027
43.67	592.63964	43.67	1032
43.68	594.63964	43.68	1041
43.69	577.63964	43.69	1033
43.7	568.63964	43.7	1025
43.71	570.63964	43.71	1041
43.72	608.63964	43.72	1047
43.73	548.63964	43.73	1033
43.74	570.63964	43.74	1041
43.75	581.63964	43.75	1028
43.76	565.63964	43.76	1017
43.77	582.63964	43.77	1023
43.78	584.63964	43.78	1050
43.79	583.63964	43.79	1048
43.8	583.63964	43.8	1048
43.81	575.63964	43.81	1029
43.82	546.63964	43.82	1054
43.83	562.63964	43.83	1012

43.84	611.63964	43.84	1032
43.85	582.63964	43.85	1065
43.86	588.63964	43.86	1054
43.87	554.63964	43.87	1018
43.88	564.63964	43.88	1035
43.89	634.63964	43.89	1032
43.9	554.63964	43.9	1041
43.91	609.63964	43.91	1039
43.92	556.63964	43.92	1049
43.93	551.63964	43.93	1052
43.94	620.63964	43.94	1052
43.95	611.63964	43.95	1047
43.96	559.63964	43.96	1043
43.97	580.63964	43.97	1028
43.98	593.63964	43.98	1048
43.99	546.63964	43.99	1059
44	565.63964	44	1059
44.01	607.63964	44.01	1081
44.02	546.63964	44.02	1015
44.03	566.63964	44.03	1057
44.04	642.63964	44.04	1060
44.05	523.63964	44.05	1078

44.06	587.63964	44.06	1066
44.07	555.63964	44.07	1054
44.08	566.63964	44.08	1074
44.09	592.63964	44.09	1047
44.1	597.63964	44.1	1071
44.11	588.63964	44.11	1059
44.12	586.63964	44.12	1022
44.13	612.63964	44.13	1050
44.14	581.63964	44.14	1038
44.15	608.63964	44.15	1046
44.16	572.63964	44.16	1053
44.17	604.63964	44.17	1053
44.18	572.63964	44.18	1055
44.19	595.63964	44.19	1029
44.2	604.63964	44.2	1048
44.21	597.63964	44.21	1051
44.22	597.63964	44.22	1038
44.23	543.63964	44.23	1060
44.24	561.63964	44.24	1025
44.25	599.63964	44.25	1069
44.26	611.63964	44.26	1066
44.27	569.63964	44.27	1061

44.28	564.63964	44.28	1039
44.29	608.63964	44.29	1051
44.3	613.63964	44.3	1028
44.31	601.63964	44.31	1071
44.32	608.63964	44.32	1067
44.33	575.63964	44.33	1065
44.34	588.63964	44.34	1053
44.35	595.63964	44.35	1050
44.36	599.63964	44.36	1031
44.37	579.63964	44.37	1073
44.38	568.63964	44.38	1080
44.39	622.63964	44.39	1070
44.4	611.63964	44.4	1037
44.41	592.63964	44.41	1034
44.42	588.63964	44.42	1069
44.43	604.63964	44.43	1052
44.44	614.63964	44.44	1027
44.45	568.63964	44.45	1035
44.46	592.63964	44.46	1024
44.47	656.63964	44.47	1042
44.48	549.63964	44.48	1043
44.49	559.63964	44.49	1014

44.5	564.63964	44.5	1048
44.51	592.63964	44.51	1066
44.52	563.63964	44.52	1051
44.53	564.63964	44.53	1037
44.54	617.63964	44.54	1072
44.55	549.63964	44.55	1040
44.56	619.63964	44.56	1018
44.57	575.63964	44.57	1050
44.58	563.63964	44.58	1065
44.59	595.63964	44.59	1059
44.6	578.63964	44.6	1050
44.61	587.63964	44.61	1042
44.62	615.63964	44.62	1079
44.63	563.63964	44.63	1071
44.64	632.63964	44.64	1016
44.65	575.63964	44.65	1031
44.66	589.63964	44.66	1030
44.67	554.63964	44.67	1049
44.68	523.63964	44.68	1031
44.69	598.63964	44.69	1039
44.7	573.63964	44.7	1034
44.71	580.63964	44.71	1026

44.72	587.63964	44.72	1046
44.73	571.63964	44.73	1043
44.74	650.63964	44.74	1035
44.75	616.63964	44.75	1041
44.76	575.63964	44.76	1048
44.77	594.63964	44.77	1046
44.78	559.63964	44.78	1047
44.79	587.63964	44.79	1055
44.8	582.63964	44.8	1027
44.81	593.63964	44.81	1056
44.82	599.63964	44.82	1065
44.83	590.63964	44.83	1040
44.84	589.63964	44.84	1042
44.85	618.63964	44.85	1021
44.86	573.63964	44.86	1034
44.87	540.63964	44.87	1067
44.88	600.63964	44.88	1021
44.89	639.63964	44.89	1017
44.9	572.63964	44.9	1071
44.91	588.63964	44.91	1086
44.92	572.63964	44.92	1054
44.93	569.63964	44.93	1079

44.94	549.63964	44.94	1054
44.95	546.63964	44.95	1057
44.96	560.63964	44.96	1042
44.97	527.63964	44.97	1020
44.98	580.63964	44.98	1073
44.99	607.63964	44.99	1034
45	601.63964	45	1034
45.01	585.63964	45.01	1030
45.02	621.63964	45.02	1063
45.03	579.63964	45.03	1047
45.04	570.63964	45.04	1027
45.05	587.63964	45.05	1064
45.06	559.63964	45.06	1031
45.07	561.63964	45.07	1035
45.08	553.63964	45.08	1011
45.09	567.63964	45.09	1037
45.1	585.63964	45.1	1034
45.11	589.63964	45.11	1033
45.12	547.63964	45.12	1042
45.13	564.63964	45.13	1016
45.14	560.63964	45.14	1038
45.15	580.63964	45.15	1044

45.16	589.63964	45.16	1043
45.17	591.63964	45.17	1048
45.18	578.63964	45.18	1033
45.19	559.63964	45.19	1026
45.2	538.63964	45.2	1066
45.21	562.63964	45.21	1057
45.22	572.63964	45.22	1051
45.23	619.63964	45.23	1034
45.24	594.63964	45.24	1009
45.25	591.63964	45.25	1080
45.26	574.63964	45.26	1061
45.27	591.63964	45.27	1033
45.28	586.63964	45.28	1037
45.29	590.63964	45.29	1074
45.3	596.63964	45.3	1020
45.31	603.63964	45.31	1028
45.32	588.63964	45.32	1052
45.33	663.63964	45.33	1011
45.34	561.63964	45.34	1019
45.35	608.63964	45.35	1060
45.36	617.63964	45.36	1057
45.37	590.63964	45.37	1044

45.38	543.63964	45.38	1067
45.39	581.63964	45.39	1012
45.4	604.63964	45.4	1041
45.41	569.63964	45.41	1059
45.42	618.63964	45.42	1049
45.43	599.63964	45.43	1045
45.44	621.63964	45.44	1034
45.45	600.63964	45.45	1047
45.46	608.63964	45.46	1068
45.47	588.63964	45.47	1041
45.48	623.63964	45.48	1048
45.49	594.63964	45.49	1017
45.5	614.63964	45.5	1063
45.51	596.63964	45.51	1026
45.52	595.63964	45.52	1044
45.53	543.63964	45.53	1031
45.54	534.63964	45.54	1026
45.55	572.63964	45.55	1028
45.56	574.63964	45.56	1063
45.57	599.63964	45.57	1059
45.58	604.63964	45.58	1055
45.59	574.63964	45.59	1035

45.6	578.63964	45.6	1041
45.61	597.63964	45.61	1049
45.62	572.63964	45.62	1057
45.63	552.63964	45.63	1037
45.64	605.63964	45.64	1038
45.65	563.63964	45.65	1044
45.66	586.63964	45.66	1027
45.67	564.63964	45.67	1038
45.68	626.63964	45.68	1060
45.69	603.63964	45.69	1040
45.7	591.63964	45.7	1071
45.71	588.63964	45.71	1048
45.72	598.63964	45.72	1053
45.73	595.63964	45.73	1042
45.74	557.63964	45.74	1064
45.75	541.63964	45.75	1067
45.76	580.63964	45.76	1091
45.77	581.63964	45.77	1038
45.78	567.63964	45.78	1061
45.79	634.63964	45.79	1077
45.8	603.63964	45.8	1070
45.81	535.63964	45.81	1039

45.82	619.63964	45.82	1052
45.83	577.63964	45.83	1044
45.84	561.63964	45.84	1054
45.85	580.63964	45.85	1060
45.86	572.63964	45.86	1058
45.87	587.63964	45.87	1052
45.88	646.63964	45.88	1038
45.89	656.63964	45.89	1059
45.9	586.63964	45.9	1013
45.91	610.63964	45.91	1027
45.92	585.63964	45.92	1063
45.93	597.63964	45.93	1061
45.94	619.63964	45.94	1037
45.95	648.63964	45.95	1003
45.96	562.63964	45.96	1065
45.97	645.63964	45.97	1041
45.98	592.63964	45.98	1039
45.99	638.63964	45.99	1047
46	623.63964	46	1056
46.01	556.63964	46.01	1046
46.02	587.63964	46.02	1048
46.03	614.63964	46.03	1026

46.04	621.63964	46.04	1035
46.05	579.63964	46.05	1034
46.06	595.63964	46.06	1019
46.07	600.63964	46.07	1032
46.08	561.63964	46.08	1057
46.09	595.63964	46.09	1082
46.1	569.63964	46.1	1046
46.11	583.63964	46.11	1047
46.12	606.63964	46.12	1055
46.13	661.63964	46.13	1029
46.14	612.63964	46.14	1066
46.15	606.63964	46.15	1038
46.16	604.63964	46.16	1105
46.17	592.63964	46.17	1064
46.18	623.63964	46.18	1064
46.19	624.63964	46.19	1059
46.2	634.63964	46.2	1066
46.21	620.63964	46.21	1039
46.22	621.63964	46.22	1050
46.23	596.63964	46.23	1057
46.24	579.63964	46.24	1073
46.25	610.63964	46.25	1078

46.26	618.63964	46.26	1051
46.27	612.63964	46.27	1068
46.28	599.63964	46.28	1027
46.29	588.63964	46.29	1033
46.3	588.63964	46.3	1029
46.31	620.63964	46.31	1054
46.32	668.63964	46.32	1056
46.33	603.63964	46.33	1058
46.34	598.63964	46.34	1047
46.35	606.63964	46.35	1057
46.36	554.63964	46.36	1049
46.37	625.63964	46.37	1082
46.38	619.63964	46.38	1029
46.39	591.63964	46.39	1050
46.4	589.63964	46.4	1079
46.41	635.63964	46.41	1039
46.42	586.63964	46.42	1041
46.43	610.63964	46.43	1058
46.44	642.63964	46.44	1079
46.45	625.63964	46.45	1051
46.46	617.63964	46.46	1066
46.47	618.63964	46.47	1053

46.48	613.63964	46.48	1052
46.49	669.63964	46.49	1058
46.5	654.63964	46.5	1066
46.51	648.63964	46.51	1076
46.52	623.63964	46.52	1074
46.53	654.63964	46.53	1052
46.54	606.63964	46.54	1074
46.55	648.63964	46.55	1053
46.56	619.63964	46.56	1060
46.57	655.63964	46.57	1058
46.58	650.63964	46.58	1092
46.59	591.63964	46.59	1042
46.6	588.63964	46.6	1074
46.61	645.63964	46.61	1027
46.62	591.63964	46.62	1031
46.63	634.63964	46.63	1041
46.64	657.63964	46.64	1051
46.65	671.63964	46.65	1038
46.66	667.63964	46.66	1077
46.67	581.63964	46.67	1033
46.68	654.63964	46.68	1064
46.69	696.63964	46.69	1051

46.7	669.63964	46.7	1050
46.71	682.63964	46.71	1068
46.72	648.63964	46.72	1056
46.73	633.63964	46.73	1087
46.74	645.63964	46.74	1106
46.75	631.63964	46.75	1042
46.76	621.63964	46.76	1053
46.77	671.63964	46.77	1064
46.78	688.63964	46.78	1093
46.79	637.63964	46.79	1089
46.8	659.63964	46.8	1096
46.81	676.63964	46.81	1076
46.82	618.63964	46.82	1079
46.83	641.63964	46.83	1096
46.84	637.63964	46.84	1085
46.85	681.63964	46.85	1076
46.86	671.63964	46.86	1078
46.87	652.63964	46.87	1083
46.88	660.63964	46.88	1098
46.89	651.63964	46.89	1074
46.9	666.63964	46.9	1095
46.91	647.63964	46.91	1058

46.92	646.63964	46.92	1083
46.93	626.63964	46.93	1066
46.94	681.63964	46.94	1079
46.95	698.63964	46.95	1056
46.96	643.63964	46.96	1087
46.97	656.63964	46.97	1082
46.98	676.63964	46.98	1050
46.99	706.63964	46.99	1073
47	723.63964	47	1082
47.01	677.63964	47.01	1091
47.02	729.63964	47.02	1091
47.03	688.63964	47.03	1093
47.04	692.63964	47.04	1029
47.05	708.63964	47.05	1113
47.06	680.63964	47.06	1061
47.07	730.63964	47.07	1056
47.08	686.63964	47.08	1112
47.09	670.63964	47.09	1098
47.1	759.63964	47.1	1089
47.11	683.63964	47.11	1095
47.12	712.63964	47.12	1071
47.13	711.63964	47.13	1099

47.14	677.63964	47.14	1095
47.15	685.63964	47.15	1078
47.16	693.63964	47.16	1093
47.17	703.63964	47.17	1082
47.18	675.63964	47.18	1106
47.19	713.63964	47.19	1084
47.2	724.63964	47.2	1096
47.21	716.63964	47.21	1104
47.22	728.63964	47.22	1079
47.23	782.63964	47.23	1075
47.24	717.63964	47.24	1118
47.25	747.63964	47.25	1112
47.26	770.63964	47.26	1066
47.27	699.63964	47.27	1100
47.28	712.63964	47.28	1094
47.29	713.63964	47.29	1105
47.3	718.63964	47.3	1134
47.31	717.63964	47.31	1065
47.32	706.63964	47.32	1105
47.33	747.63964	47.33	1099
47.34	724.63964	47.34	1141
47.35	754.63964	47.35	1090

47.36	720.63964	47.36	1108
47.37	767.63964	47.37	1099
47.38	745.63964	47.38	1122
47.39	777.63964	47.39	1086
47.4	790.63964	47.4	1122
47.41	758.63964	47.41	1103
47.42	744.63964	47.42	1132
47.43	760.63964	47.43	1172
47.44	741.63964	47.44	1137
47.45	742.63964	47.45	1153
47.46	736.63964	47.46	1171
47.47	772.63964	47.47	1077
47.48	752.63964	47.48	1137
47.49	773.63964	47.49	1112
47.5	732.63964	47.5	1182
47.51	780.63964	47.51	1134
47.52	781.63964	47.52	1144
47.53	830.63964	47.53	1157
47.54	780.63964	47.54	1107
47.55	823.63964	47.55	1152
47.56	851.63964	47.56	1161
47.57	863.63964	47.57	1147

47.58	830.63964	47.58	1195
47.59	782.63964	47.59	1157
47.6	812.63964	47.6	1177
47.61	842.63964	47.61	1155
47.62	815.63964	47.62	1145
47.63	856.63964	47.63	1178
47.64	899.63964	47.64	1195
47.65	882.63964	47.65	1177
47.66	855.63964	47.66	1160
47.67	917.63964	47.67	1170
47.68	884.63964	47.68	1155
47.69	916.63964	47.69	1162
47.7	918.63964	47.7	1171
47.71	929.63964	47.71	1160
47.72	976.63964	47.72	1178
47.73	950.63964	47.73	1197
47.74	953.63964	47.74	1194
47.75	932.63964	47.75	1232
47.76	991.63964	47.76	1244
47.77	998.63964	47.77	1262
47.78	980.63964	47.78	1176
47.79	951.63964	47.79	1218

47.8	995.63964	47.8	1251
47.81	975.63964	47.81	1222
47.82	1044.63964	47.82	1247
47.83	1017.63964	47.83	1223
47.84	1029.63964	47.84	1233
47.85	1046.63964	47.85	1225
47.86	1047.63964	47.86	1251
47.87	1114.63964	47.87	1250
47.88	1086.63964	47.88	1322
47.89	1066.63964	47.89	1272
47.9	1122.63964	47.9	1235
47.91	1098.63964	47.91	1267
47.92	1047.63964	47.92	1275
47.93	1051.63964	47.93	1278
47.94	1120.63964	47.94	1271
47.95	1156.63964	47.95	1262
47.96	1108.63964	47.96	1272
47.97	1136.63964	47.97	1253
47.98	1099.63964	47.98	1298
47.99	1072.63964	47.99	1275
48	1099.63964	48	1268
48.01	1131.63964	48.01	1270

48.02	1133.63964	48.02	1247
48.03	1090.63964	48.03	1251
48.04	1110.63964	48.04	1253
48.05	1093.63964	48.05	1242
48.06	1063.63964	48.06	1264
48.07	1110.63964	48.07	1266
48.08	1050.63964	48.08	1237
48.09	1034.63964	48.09	1246
48.1	1114.63964	48.1	1235
48.11	1025.63964	48.11	1267
48.12	1006.63964	48.12	1247
48.13	1057.63964	48.13	1245
48.14	1003.63964	48.14	1182
48.15	1008.63964	48.15	1196
48.16	1017.63964	48.16	1262
48.17	1036.63964	48.17	1196
48.18	989.63964	48.18	1236
48.19	999.63964	48.19	1197
48.2	1000.63964	48.2	1228
48.21	984.63964	48.21	1204
48.22	950.63964	48.22	1200
48.23	881.63964	48.23	1195

48.24	906.63964	48.24	1188
48.25	952.63964	48.25	1169
48.26	927.63964	48.26	1175
48.27	890.63964	48.27	1144
48.28	942.63964	48.28	1154
48.29	892.63964	48.29	1137
48.3	862.63964	48.3	1152
48.31	869.63964	48.31	1185
48.32	901.63964	48.32	1160
48.33	823.63964	48.33	1150
48.34	865.63964	48.34	1108
48.35	842.63964	48.35	1145
48.36	815.63964	48.36	1192
48.37	833.63964	48.37	1139
48.38	808.63964	48.38	1148
48.39	824.63964	48.39	1124
48.4	791.63964	48.4	1125
48.41	841.63964	48.41	1116
48.42	844.63964	48.42	1146
48.43	774.63964	48.43	1158
48.44	781.63964	48.44	1126
48.45	824.63964	48.45	1118

48.46	765.63964	48.46	1098
48.47	793.63964	48.47	1112
48.48	768.63964	48.48	1121
48.49	771.63964	48.49	1105
48.5	774.63964	48.5	1116
48.51	767.63964	48.51	1121
48.52	727.63964	48.52	1112
48.53	742.63964	48.53	1110
48.54	752.63964	48.54	1082
48.55	736.63964	48.55	1099
48.56	745.63964	48.56	1079
48.57	706.63964	48.57	1107
48.58	707.63964	48.58	1121
48.59	730.63964	48.59	1122
48.6	740.63964	48.6	1094
48.61	716.63964	48.61	1082
48.62	697.63964	48.62	1091
48.63	655.63964	48.63	1084
48.64	678.63964	48.64	1094
48.65	683.63964	48.65	1094
48.66	672.63964	48.66	1060
48.67	723.63964	48.67	1086

48.68	719.63964	48.68	1100
48.69	716.63964	48.69	1046
48.7	720.63964	48.7	1115
48.71	722.63964	48.71	1084
48.72	632.63964	48.72	1100
48.73	665.63964	48.73	1099
48.74	718.63964	48.74	1083
48.75	700.63964	48.75	1083
48.76	684.63964	48.76	1070
48.77	674.63964	48.77	1061
48.78	716.63964	48.78	1096
48.79	737.63964	48.79	1058
48.8	682.63964	48.8	1103
48.81	669.63964	48.81	1097
48.82	689.63964	48.82	1099
48.83	671.63964	48.83	1102
48.84	702.63964	48.84	1095
48.85	710.63964	48.85	1095
48.86	689.63964	48.86	1069
48.87	665.63964	48.87	1050
48.88	648.63964	48.88	1063
48.89	695.63964	48.89	1063

48.9	700.63964	48.9	1030
48.91	611.63964	48.91	1087
48.92	664.63964	48.92	1093
48.93	627.63964	48.93	1076
48.94	694.63964	48.94	1079
48.95	651.63964	48.95	1047
48.96	694.63964	48.96	1076
48.97	681.63964	48.97	1055
48.98	704.63964	48.98	1085
48.99	649.63964	48.99	1103
49	664.63964	49	1069
49.01	650.63964	49.01	1058
49.02	600.63964	49.02	1040
49.03	665.63964	49.03	1064
49.04	681.63964	49.04	1059
49.05	627.63964	49.05	1066
49.06	624.63964	49.06	1041
49.07	652.63964	49.07	1064
49.08	642.63964	49.08	1092
49.09	607.63964	49.09	1060
49.1	647.63964	49.1	1050
49.11	665.63964	49.11	1063

49.12	646.63964	49.12	1075
49.13	644.63964	49.13	1055
49.14	623.63964	49.14	1035
49.15	652.63964	49.15	1083
49.16	594.63964	49.16	1067
49.17	611.63964	49.17	1054
49.18	624.63964	49.18	1046
49.19	646.63964	49.19	1048
49.2	621.63964	49.2	1053
49.21	644.63964	49.21	1052
49.22	637.63964	49.22	1024
49.23	633.63964	49.23	1058
49.24	612.63964	49.24	1061
49.25	621.63964	49.25	1035
49.26	654.63964	49.26	1083
49.27	629.63964	49.27	1097
49.28	633.63964	49.28	1047
49.29	656.63964	49.29	1081
49.3	658.63964	49.3	1085
49.31	637.63964	49.31	1057
49.32	611.63964	49.32	1075
49.33	657.63964	49.33	1080

49.34	634.63964	49.34	1072
49.35	639.63964	49.35	1081
49.36	638.63964	49.36	1053
49.37	636.63964	49.37	1058
49.38	631.63964	49.38	1078
49.39	647.63964	49.39	1053
49.4	595.63964	49.4	1044
49.41	605.63964	49.41	1080
49.42	621.63964	49.42	1054
49.43	612.63964	49.43	1038
49.44	618.63964	49.44	1044
49.45	617.63964	49.45	1039
49.46	638.63964	49.46	1035
49.47	595.63964	49.47	1014
49.48	619.63964	49.48	1064
49.49	643.63964	49.49	1030
49.5	607.63964	49.5	1049
49.51	638.63964	49.51	1078
49.52	648.63964	49.52	1032
49.53	593.63964	49.53	1056
49.54	650.63964	49.54	1048
49.55	659.63964	49.55	1059

49.56	620.63964	49.56	1080
49.57	638.63964	49.57	1085
49.58	639.63964	49.58	1025
49.59	640.63964	49.59	1079
49.6	616.63964	49.6	1050
49.61	600.63964	49.61	1064
49.62	672.63964	49.62	1027
49.63	625.63964	49.63	1038
49.64	606.63964	49.64	1033
49.65	596.63964	49.65	1044
49.66	572.63964	49.66	1058
49.67	583.63964	49.67	1071
49.68	610.63964	49.68	1065
49.69	580.63964	49.69	1077
49.7	609.63964	49.7	1063
49.71	596.63964	49.71	1054
49.72	619.63964	49.72	1064
49.73	658.63964	49.73	1061
49.74	627.63964	49.74	1031
49.75	643.63964	49.75	1085
49.76	605.63964	49.76	1061
49.77	586.63964	49.77	1049

49.78	608.63964	49.78	1059
49.79	596.63964	49.79	1050
49.8	597.63964	49.8	1046
49.81	561.63964	49.81	1044
49.82	575.63964	49.82	1060
49.83	636.63964	49.83	1045
49.84	621.63964	49.84	1040
49.85	640.63964	49.85	1059
49.86	615.63964	49.86	1053
49.87	610.63964	49.87	1070
49.88	632.63964	49.88	1085
49.89	654.63964	49.89	1030
49.9	607.63964	49.9	1049
49.91	623.63964	49.91	1043
49.92	639.63964	49.92	1011
49.93	637.63964	49.93	1041
49.94	619.63964	49.94	1034
49.95	623.63964	49.95	1055
49.96	605.63964	49.96	1019
49.97	609.63964	49.97	1067
49.98	609.63964	49.98	1048
49.99	635.63964	49.99	1031

50	586.63964	50	1061
50.01	589.63964	50.01	1041
50.02	619.63964	50.02	1060
50.03	606.63964	50.03	1032
50.04	593.63964	50.04	1032
50.05	640.63964	50.05	1045
50.06	608.63964	50.06	1051
50.07	603.63964	50.07	1065
50.08	620.63964	50.08	1039
50.09	579.63964	50.09	1054
50.1	599.63964	50.1	1052
50.11	580.63964	50.11	1042
50.12	597.63964	50.12	1039
50.13	572.63964	50.13	1063
50.14	590.63964	50.14	1060
50.15	620.63964	50.15	1020
50.16	641.63964	50.16	1022
50.17	607.63964	50.17	1027
50.18	568.63964	50.18	1033
50.19	593.63964	50.19	1025
50.2	597.63964	50.2	1042
50.21	561.63964	50.21	1038

50.22	615.63964	50.22	1044
50.23	614.63964	50.23	1043
50.24	590.63964	50.24	1077
50.25	595.63964	50.25	1064
50.26	618.63964	50.26	1061
50.27	622.63964	50.27	1039
50.28	609.63964	50.28	1064
50.29	583.63964	50.29	1050
50.3	581.63964	50.3	1037
50.31	582.63964	50.31	1043
50.32	591.63964	50.32	1065
50.33	647.63964	50.33	1044
50.34	593.63964	50.34	1061
50.35	605.63964	50.35	1064
50.36	613.63964	50.36	1048
50.37	589.63964	50.37	1013
50.38	610.63964	50.38	1060
50.39	603.63964	50.39	1055
50.4	563.63964	50.4	1043
50.41	598.63964	50.41	1061
50.42	599.63964	50.42	1041
50.43	581.63964	50.43	1049

50.44	650.63964	50.44	1046
50.45	632.63964	50.45	1078
50.46	603.63964	50.46	1074
50.47	571.63964	50.47	1031
50.48	622.63964	50.48	1053
50.49	623.63964	50.49	1072
50.5	581.63964	50.5	1043
50.51	599.63964	50.51	1055
50.52	567.63964	50.52	1042
50.53	587.63964	50.53	1056
50.54	612.63964	50.54	1028
50.55	573.63964	50.55	1037
50.56	572.63964	50.56	1037
50.57	572.63964	50.57	1053
50.58	578.63964	50.58	1055
50.59	594.63964	50.59	1056
50.6	596.63964	50.6	1058
50.61	579.63964	50.61	1072
50.62	588.63964	50.62	1051
50.63	586.63964	50.63	1016
50.64	611.63964	50.64	1015
50.65	577.63964	50.65	1050

50.66	630.63964	50.66	1042
50.67	614.63964	50.67	1027
50.68	586.63964	50.68	1064
50.69	589.63964	50.69	1080
50.7	604.63964	50.7	1030
50.71	567.63964	50.71	1044
50.72	586.63964	50.72	1034
50.73	638.63964	50.73	1038
50.74	637.63964	50.74	1047
50.75	606.63964	50.75	1043
50.76	623.63964	50.76	1063
50.77	611.63964	50.77	1053
50.78	584.63964	50.78	1051
50.79	555.63964	50.79	1058
50.8	619.63964	50.8	1014
50.81	588.63964	50.81	1039
50.82	665.63964	50.82	1049
50.83	592.63964	50.83	1043
50.84	601.63964	50.84	1044
50.85	618.63964	50.85	1039
50.86	578.63964	50.86	1056
50.87	591.63964	50.87	1046

50.88	590.63964	50.88	1056
50.89	622.63964	50.89	1068
50.9	575.63964	50.9	1067
50.91	620.63964	50.91	1023
50.92	594.63964	50.92	1011
50.93	606.63964	50.93	1037
50.94	565.63964	50.94	1057
50.95	608.63964	50.95	1051
50.96	585.63964	50.96	1045
50.97	583.63964	50.97	1060
50.98	579.63964	50.98	1029
50.99	596.63964	50.99	1038
51	610.63964	51	1043
51.01	595.63964	51.01	1054
51.02	575.63964	51.02	1039
51.03	613.63964	51.03	1049
51.04	555.63964	51.04	1046
51.05	598.63964	51.05	1055
51.06	612.63964	51.06	1065
51.07	619.63964	51.07	1020
51.08	654.63964	51.08	1040
51.09	597.63964	51.09	1076

51.1	595.63964	51.1	1069
51.11	566.63964	51.11	1047
51.12	604.63964	51.12	1066
51.13	601.63964	51.13	1034
51.14	614.63964	51.14	1039
51.15	595.63964	51.15	1040
51.16	581.63964	51.16	1020
51.17	591.63964	51.17	1101
51.18	624.63964	51.18	1060
51.19	559.63964	51.19	1072
51.2	638.63964	51.2	1035
51.21	606.63964	51.21	1032
51.22	585.63964	51.22	1038
51.23	622.63964	51.23	1052
51.24	600.63964	51.24	1043
51.25	596.63964	51.25	1009
51.26	621.63964	51.26	1043
51.27	607.63964	51.27	1072
51.28	540.63964	51.28	1060
51.29	637.63964	51.29	1056
51.3	599.63964	51.3	1035
51.31	603.63964	51.31	1055

51.32	600.63964	51.32	1050
51.33	590.63964	51.33	1032
51.34	595.63964	51.34	1045
51.35	585.63964	51.35	1045
51.36	577.63964	51.36	1037
51.37	607.63964	51.37	1052
51.38	591.63964	51.38	1039
51.39	605.63964	51.39	1066
51.4	596.63964	51.4	1060
51.41	585.63964	51.41	1063
51.42	611.63964	51.42	1052
51.43	597.63964	51.43	1046
51.44	587.63964	51.44	1029
51.45	603.63964	51.45	1057
51.46	562.63964	51.46	1035
51.47	617.63964	51.47	1045
51.48	631.63964	51.48	1067
51.49	559.63964	51.49	1052
51.5	566.63964	51.5	1074
51.51	633.63964	51.51	1052
51.52	562.63964	51.52	1022
51.53	603.63964	51.53	1018

51.54	612.63964	51.54	1031
51.55	579.63964	51.55	1021
51.56	642.63964	51.56	1049
51.57	617.63964	51.57	1081
51.58	626.63964	51.58	1059
51.59	596.63964	51.59	996
51.6	614.63964	51.6	1016
51.61	597.63964	51.61	1068
51.62	626.63964	51.62	1038
51.63	621.63964	51.63	1073
51.64	586.63964	51.64	1057
51.65	624.63964	51.65	1040
51.66	617.63964	51.66	1032
51.67	593.63964	51.67	1033
51.68	586.63964	51.68	1041
51.69	613.63964	51.69	1071
51.7	581.63964	51.7	1070
51.71	565.63964	51.71	1053
51.72	587.63964	51.72	1028
51.73	635.63964	51.73	1048
51.74	626.63964	51.74	1044
51.75	631.63964	51.75	1040

51.76	575.63964	51.76	1054
51.77	570.63964	51.77	1025
51.78	617.63964	51.78	1026
51.79	615.63964	51.79	1065
51.8	582.63964	51.8	1025
51.81	594.63964	51.81	1030
51.82	598.63964	51.82	1007
51.83	621.63964	51.83	1033
51.84	635.63964	51.84	1041
51.85	604.63964	51.85	1015
51.86	591.63964	51.86	1047
51.87	637.63964	51.87	1070
51.88	656.63964	51.88	1017
51.89	646.63964	51.89	1052
51.9	647.63964	51.9	1055
51.91	601.63964	51.91	1049
51.92	606.63964	51.92	1055
51.93	629.63964	51.93	1064
51.94	593.63964	51.94	1043
51.95	621.63964	51.95	1035
51.96	588.63964	51.96	1046
51.97	588.63964	51.97	1051

51.98	625.63964	51.98	1038
51.99	612.63964	51.99	1051
52	640.63964	52	1050
52.01	626.63964	52.01	1032
52.02	635.63964	52.02	1063
52.03	570.63964	52.03	1042
52.04	640.63964	52.04	1050
52.05	640.63964	52.05	1024
52.06	624.63964	52.06	1044
52.07	633.63964	52.07	1038
52.08	661.63964	52.08	1060
52.09	673.63964	52.09	1081
52.1	630.63964	52.1	1030
52.11	578.63964	52.11	1060
52.12	600.63964	52.12	1049
52.13	617.63964	52.13	1051
52.14	626.63964	52.14	1066
52.15	610.63964	52.15	1071
52.16	607.63964	52.16	1067
52.17	624.63964	52.17	1054
52.18	585.63964	52.18	1067
52.19	585.63964	52.19	1019

52.2	620.63964	52.2	1057
52.21	614.63964	52.21	1055
52.22	664.63964	52.22	1055
52.23	664.63964	52.23	1066
52.24	609.63964	52.24	1059
52.25	675.63964	52.25	1035
52.26	622.63964	52.26	1058
52.27	620.63964	52.27	1052
52.28	630.63964	52.28	1055
52.29	593.63964	52.29	1051
52.3	670.63964	52.3	1048
52.31	625.63964	52.31	1021
52.32	677.63964	52.32	1067
52.33	617.63964	52.33	1064
52.34	647.63964	52.34	1038
52.35	720.63964	52.35	1050
52.36	669.63964	52.36	1043
52.37	662.63964	52.37	1043
52.38	660.63964	52.38	1050
52.39	662.63964	52.39	1076
52.4	644.63964	52.4	1045
52.41	715.63964	52.41	1067

52.42	687.63964	52.42	1077
52.43	657.63964	52.43	1057
52.44	650.63964	52.44	1050
52.45	672.63964	52.45	1065
52.46	682.63964	52.46	1116
52.47	632.63964	52.47	1076
52.48	695.63964	52.48	1093
52.49	652.63964	52.49	1027
52.5	664.63964	52.5	1064
52.51	670.63964	52.51	1092
52.52	703.63964	52.52	1100
52.53	654.63964	52.53	1069
52.54	694.63964	52.54	1089
52.55	651.63964	52.55	1054
52.56	667.63964	52.56	1097
52.57	651.63964	52.57	1103
52.58	672.63964	52.58	1072
52.59	714.63964	52.59	1046
52.6	652.63964	52.6	1079
52.61	655.63964	52.61	1069
52.62	689.63964	52.62	1059
52.63	672.63964	52.63	1069

52.64	678.63964	52.64	1057
52.65	671.63964	52.65	1050
52.66	685.63964	52.66	1064
52.67	635.63964	52.67	1058
52.68	668.63964	52.68	1078
52.69	672.63964	52.69	1069
52.7	637.63964	52.7	1032
52.71	636.63964	52.71	1056
52.72	697.63964	52.72	1110
52.73	720.63964	52.73	1089
52.74	654.63964	52.74	1089
52.75	644.63964	52.75	1072
52.76	687.63964	52.76	1087
52.77	708.63964	52.77	1085
52.78	648.63964	52.78	1089
52.79	712.63964	52.79	1085
52.8	657.63964	52.8	1065
52.81	663.63964	52.81	1053
52.82	642.63964	52.82	1098
52.83	657.63964	52.83	1091
52.84	687.63964	52.84	1083
52.85	652.63964	52.85	1078

52.86	671.63964	52.86	1057
52.87	659.63964	52.87	1053
52.88	738.63964	52.88	1084
52.89	699.63964	52.89	1061
52.9	691.63964	52.9	1059
52.91	690.63964	52.91	1057
52.92	660.63964	52.92	1086
52.93	696.63964	52.93	1083
52.94	681.63964	52.94	1098
52.95	685.63964	52.95	1101
52.96	646.63964	52.96	1064
52.97	666.63964	52.97	1089
52.98	666.63964	52.98	1049
52.99	680.63964	52.99	1085
53	674.63964	53	1057
53.01	689.63964	53.01	1076
53.02	663.63964	53.02	1079
53.03	645.63964	53.03	1081
53.04	643.63964	53.04	1055
53.05	631.63964	53.05	1020
53.06	674.63964	53.06	1037
53.07	660.63964	53.07	1098

53.08	650.63964	53.08	1052
53.09	655.63964	53.09	1059
53.1	676.63964	53.1	1058
53.11	658.63964	53.11	1085
53.12	637.63964	53.12	1039
53.13	662.63964	53.13	1065
53.14	632.63964	53.14	1080
53.15	655.63964	53.15	1065
53.16	656.63964	53.16	1080
53.17	645.63964	53.17	1085
53.18	660.63964	53.18	1071
53.19	663.63964	53.19	1054
53.2	630.63964	53.2	1073
53.21	650.63964	53.21	1049
53.22	624.63964	53.22	1052
53.23	644.63964	53.23	1064
53.24	644.63964	53.24	1065
53.25	643.63964	53.25	1060
53.26	606.63964	53.26	1039
53.27	608.63964	53.27	1051
53.28	615.63964	53.28	1081
53.29	660.63964	53.29	1027

53.3	625.63964	53.3	1071
53.31	652.63964	53.31	1064
53.32	565.63964	53.32	1042
53.33	571.63964	53.33	1051
53.34	648.63964	53.34	1082
53.35	639.63964	53.35	1028
53.36	604.63964	53.36	1067
53.37	627.63964	53.37	1045
53.38	614.63964	53.38	1043
53.39	567.63964	53.39	1073
53.4	631.63964	53.4	1071
53.41	572.63964	53.41	1099
53.42	613.63964	53.42	1055
53.43	644.63964	53.43	1095
53.44	595.63964	53.44	1074
53.45	571.63964	53.45	1050
53.46	613.63964	53.46	1055
53.47	590.63964	53.47	1044
53.48	596.63964	53.48	1037
53.49	611.63964	53.49	1049
53.5	635.63964	53.5	1039
53.51	587.63964	53.51	1055

53.52	610.63964	53.52	1034
53.53	610.63964	53.53	1058
53.54	598.63964	53.54	1061
53.55	589.63964	53.55	1065
53.56	597.63964	53.56	1029
53.57	596.63964	53.57	1049
53.58	598.63964	53.58	1043
53.59	575.63964	53.59	1024
53.6	613.63964	53.6	1029
53.61	592.63964	53.61	1063
53.62	595.63964	53.62	1030
53.63	547.63964	53.63	1026
53.64	569.63964	53.64	1030
53.65	590.63964	53.65	1043
53.66	601.63964	53.66	1038
53.67	609.63964	53.67	1017
53.68	623.63964	53.68	1041
53.69	549.63964	53.69	1009
53.7	627.63964	53.7	1040
53.71	597.63964	53.71	1023
53.72	596.63964	53.72	1060
53.73	579.63964	53.73	1049

53.74	611.63964	53.74	1038
53.75	568.63964	53.75	1072
53.76	588.63964	53.76	1071
53.77	603.63964	53.77	1048
53.78	597.63964	53.78	1041
53.79	600.63964	53.79	1041
53.8	616.63964	53.8	1094
53.81	587.63964	53.81	1029
53.82	633.63964	53.82	1053
53.83	598.63964	53.83	1027
53.84	611.63964	53.84	1026
53.85	567.63964	53.85	1053
53.86	597.63964	53.86	1003
53.87	606.63964	53.87	1051
53.88	582.63964	53.88	1078
53.89	545.63964	53.89	1038
53.9	561.63964	53.9	1068
53.91	573.63964	53.91	1028
53.92	612.63964	53.92	1024
53.93	577.63964	53.93	1051
53.94	585.63964	53.94	1051
53.95	545.63964	53.95	1034

53.96	548.63964	53.96	1006
53.97	579.63964	53.97	1027
53.98	579.63964	53.98	1060
53.99	525.63964	53.99	1049
54	583.63964	54	1076
54.01	564.63964	54.01	1060
54.02	591.63964	54.02	1050
54.03	586.63964	54.03	1049
54.04	572.63964	54.04	1032
54.05	573.63964	54.05	1033
54.06	620.63964	54.06	1053
54.07	624.63964	54.07	1048
54.08	562.63964	54.08	1033
54.09	520.63964	54.09	1020
54.1	542.63964	54.1	1025
54.11	570.63964	54.11	1043
54.12	583.63964	54.12	1035
54.13	544.63964	54.13	1043
54.14	592.63964	54.14	1050
54.15	580.63964	54.15	1022
54.16	596.63964	54.16	1074
54.17	615.63964	54.17	1022

54.18	575.63964	54.18	1031
54.19	570.63964	54.19	1058
54.2	566.63964	54.2	1059
54.21	585.63964	54.21	1020
54.22	597.63964	54.22	1040
54.23	579.63964	54.23	1018
54.24	583.63964	54.24	1057
54.25	593.63964	54.25	1044
54.26	523.63964	54.26	1004
54.27	622.63964	54.27	1017
54.28	589.63964	54.28	1035
54.29	604.63964	54.29	1038
54.3	611.63964	54.3	1021
54.31	530.63964	54.31	1026
54.32	584.63964	54.32	1050
54.33	564.63964	54.33	1040
54.34	587.63964	54.34	1064
54.35	562.63964	54.35	1031
54.36	586.63964	54.36	1005
54.37	588.63964	54.37	1016
54.38	597.63964	54.38	1024
54.39	563.63964	54.39	1054

54.4	598.63964	54.4	1027
54.41	560.63964	54.41	1006
54.42	560.63964	54.42	1042
54.43	552.63964	54.43	1039
54.44	605.63964	54.44	1069
54.45	590.63964	54.45	1068
54.46	577.63964	54.46	1027
54.47	600.63964	54.47	1009
54.48	567.63964	54.48	1012
54.49	584.63964	54.49	1026
54.5	614.63964	54.5	1046
54.51	553.63964	54.51	1026
54.52	586.63964	54.52	1016
54.53	606.63964	54.53	1023
54.54	563.63964	54.54	1005
54.55	599.63964	54.55	1027
54.56	612.63964	54.56	1044
54.57	631.63964	54.57	1045
54.58	574.63964	54.58	1066
54.59	589.63964	54.59	1040
54.6	599.63964	54.6	1017
54.61	594.63964	54.61	1029

54.62	558.63964	54.62	1042
54.63	575.63964	54.63	1032
54.64	560.63964	54.64	1031
54.65	584.63964	54.65	1031
54.66	578.63964	54.66	1070
54.67	611.63964	54.67	1033
54.68	580.63964	54.68	1029
54.69	604.63964	54.69	1041
54.7	560.63964	54.7	1040
54.71	581.63964	54.71	1023
54.72	564.63964	54.72	1066
54.73	563.63964	54.73	1058
54.74	584.63964	54.74	1014
54.75	552.63964	54.75	1003
54.76	542.63964	54.76	1027
54.77	543.63964	54.77	1048
54.78	544.63964	54.78	1026
54.79	563.63964	54.79	1009
54.8	528.63964	54.8	1035
54.81	587.63964	54.81	1058
54.82	565.63964	54.82	1015
54.83	569.63964	54.83	1029

54.84	567.63964	54.84	1051
54.85	603.63964	54.85	1032
54.86	545.63964	54.86	1022
54.87	596.63964	54.87	1032
54.88	566.63964	54.88	1084
54.89	572.63964	54.89	1014
54.9	598.63964	54.9	993
54.91	597.63964	54.91	994
54.92	561.63964	54.92	1043
54.93	569.63964	54.93	1009
54.94	604.63964	54.94	1043
54.95	574.63964	54.95	1023
54.96	575.63964	54.96	1045
54.97	605.63964	54.97	1049
54.98	615.63964	54.98	1027
54.99	566.63964	54.99	1005
55	545.63964	55	1021
55.01	517.63964	55.01	1015
55.02	563.63964	55.02	1006
55.03	547.63964	55.03	1031
55.04	597.63964	55.04	1026
55.05	584.63964	55.05	1038

55.06	573.63964	55.06	1038
55.07	578.63964	55.07	1035
55.08	608.63964	55.08	1023
55.09	615.63964	55.09	1072
55.1	573.63964	55.1	1030
55.11	560.63964	55.11	1006
55.12	563.63964	55.12	1051
55.13	565.63964	55.13	1043
55.14	593.63964	55.14	1040
55.15	585.63964	55.15	1031
55.16	580.63964	55.16	1032
55.17	624.63964	55.17	1023
55.18	572.63964	55.18	1026
55.19	562.63964	55.19	1011
55.2	593.63964	55.2	1033
55.21	577.63964	55.21	1017
55.22	605.63964	55.22	1031
55.23	577.63964	55.23	1048
55.24	530.63964	55.24	1020
55.25	580.63964	55.25	1039
55.26	560.63964	55.26	1028
55.27	557.63964	55.27	1033

55.28	546.63964	55.28	1041
55.29	598.63964	55.29	1047
55.3	559.63964	55.3	1024
55.31	598.63964	55.31	1032
55.32	603.63964	55.32	1054
55.33	567.63964	55.33	1015
55.34	561.63964	55.34	1025
55.35	541.63964	55.35	1025
55.36	529.63964	55.36	1053
55.37	580.63964	55.37	1045
55.38	607.63964	55.38	1084
55.39	585.63964	55.39	1052
55.4	586.63964	55.4	1028
55.41	574.63964	55.41	1037
55.42	535.63964	55.42	1051
55.43	562.63964	55.43	1029
55.44	604.63964	55.44	1002
55.45	567.63964	55.45	1020
55.46	554.63964	55.46	1026
55.47	600.63964	55.47	1025
55.48	604.63964	55.48	1038
55.49	606.63964	55.49	1005

55.5	577.63964	55.5	1019
55.51	585.63964	55.51	1059
55.52	566.63964	55.52	1038
55.53	538.63964	55.53	1029
55.54	547.63964	55.54	1026
55.55	567.63964	55.55	1039
55.56	611.63964	55.56	1021
55.57	615.63964	55.57	1058
55.58	592.63964	55.58	1021
55.59	580.63964	55.59	1038
55.6	574.63964	55.6	1050
55.61	569.63964	55.61	1007
55.62	577.63964	55.62	995
55.63	558.63964	55.63	1003
55.64	562.63964	55.64	1012
55.65	596.63964	55.65	1004
55.66	561.63964	55.66	1055
55.67	552.63964	55.67	1023
55.68	612.63964	55.68	1065
55.69	582.63964	55.69	1056
55.7	574.63964	55.7	1065
55.71	633.63964	55.71	1013

55.72	573.63964	55.72	1042
55.73	600.63964	55.73	1035
55.74	541.63964	55.74	1016
55.75	584.63964	55.75	1070
55.76	542.63964	55.76	1031
55.77	593.63964	55.77	1035
55.78	581.63964	55.78	1029
55.79	544.63964	55.79	1045
55.8	537.63964	55.8	1038
55.81	635.63964	55.81	1060
55.82	581.63964	55.82	1068
55.83	586.63964	55.83	1034
55.84	566.63964	55.84	1079
55.85	561.63964	55.85	1006
55.86	577.63964	55.86	1017
55.87	565.63964	55.87	1048
55.88	570.63964	55.88	1029
55.89	577.63964	55.89	1004
55.9	565.63964	55.9	1029
55.91	594.63964	55.91	1027
55.92	568.63964	55.92	1011
55.93	608.63964	55.93	1032

55.94	553.63964	55.94	1045
55.95	611.63964	55.95	1015
55.96	618.63964	55.96	1045
55.97	565.63964	55.97	1021
55.98	560.63964	55.98	1049
55.99	601.63964	55.99	1032
56	561.63964	56	1037
56.01	618.63964	56.01	1026
56.02	556.63964	56.02	1005
56.03	585.63964	56.03	1061
56.04	598.63964	56.04	1042
56.05	604.63964	56.05	1054
56.06	572.63964	56.06	1009
56.07	614.63964	56.07	1015
56.08	608.63964	56.08	1026
56.09	613.63964	56.09	1040
56.1	573.63964	56.1	1034
56.11	609.63964	56.11	1032
56.12	586.63964	56.12	1021
56.13	579.63964	56.13	1042
56.14	606.63964	56.14	1026
56.15	580.63964	56.15	1030

56.16	567.63964	56.16	1046
56.17	572.63964	56.17	1022
56.18	593.63964	56.18	1058
56.19	611.63964	56.19	1050
56.2	598.63964	56.2	1030
56.21	575.63964	56.21	1033
56.22	580.63964	56.22	1048
56.23	589.63964	56.23	1025
56.24	592.63964	56.24	1038
56.25	592.63964	56.25	1052
56.26	586.63964	56.26	1056
56.27	607.63964	56.27	1023
56.28	636.63964	56.28	1028
56.29	595.63964	56.29	1020
56.3	561.63964	56.3	1028
56.31	617.63964	56.31	1058
56.32	593.63964	56.32	1033
56.33	612.63964	56.33	1016
56.34	581.63964	56.34	1065
56.35	624.63964	56.35	1076
56.36	592.63964	56.36	1043
56.37	584.63964	56.37	1030

56.38	573.63964	56.38	1042
56.39	537.63964	56.39	1016
56.4	617.63964	56.4	1026
56.41	578.63964	56.41	1057
56.42	557.63964	56.42	1026
56.43	594.63964	56.43	1046
56.44	610.63964	56.44	1063
56.45	530.63964	56.45	1063
56.46	543.63964	56.46	1018
56.47	562.63964	56.47	1009
56.48	620.63964	56.48	1045
56.49	593.63964	56.49	1040
56.5	615.63964	56.5	1046
56.51	572.63964	56.51	1034
56.52	587.63964	56.52	1046
56.53	577.63964	56.53	1008
56.54	560.63964	56.54	1054
56.55	575.63964	56.55	1017
56.56	556.63964	56.56	1032
56.57	570.63964	56.57	1017
56.58	594.63964	56.58	1031
56.59	607.63964	56.59	1049

56.6	558.63964	56.6	992
56.61	557.63964	56.61	1037
56.62	575.63964	56.62	1028
56.63	573.63964	56.63	1049
56.64	601.63964	56.64	1060
56.65	601.63964	56.65	1047
56.66	577.63964	56.66	1051
56.67	571.63964	56.67	1045
56.68	614.63964	56.68	1031
56.69	601.63964	56.69	1058
56.7	604.63964	56.7	1060
56.71	565.63964	56.71	1015
56.72	593.63964	56.72	1053
56.73	585.63964	56.73	1046
56.74	621.63964	56.74	999
56.75	593.63964	56.75	1020
56.76	558.63964	56.76	1022
56.77	629.63964	56.77	1041
56.78	538.63964	56.78	1030
56.79	562.63964	56.79	1056
56.8	595.63964	56.8	1044
56.81	591.63964	56.81	1017

56.82	593.63964	56.82	1015
56.83	606.63964	56.83	1047
56.84	610.63964	56.84	1023
56.85	595.63964	56.85	1045
56.86	572.63964	56.86	1024
56.87	558.63964	56.87	1005
56.88	605.63964	56.88	1042
56.89	610.63964	56.89	1051
56.9	585.63964	56.9	1052
56.91	589.63964	56.91	1068
56.92	597.63964	56.92	1006
56.93	569.63964	56.93	1027
56.94	557.63964	56.94	1040
56.95	586.63964	56.95	1054
56.96	566.63964	56.96	1056
56.97	575.63964	56.97	1014
56.98	619.63964	56.98	1041
56.99	624.63964	56.99	1073
57	579.63964	57	1059
57.01	606.63964	57.01	1061
57.02	562.63964	57.02	1014
57.03	588.63964	57.03	1054

57.04	577.63964	57.04	1047
57.05	574.63964	57.05	1024
57.06	594.63964	57.06	1038
57.07	617.63964	57.07	1058
57.08	591.63964	57.08	1068
57.09	552.63964	57.09	1032
57.1	626.63964	57.1	1037
57.11	630.63964	57.11	1051
57.12	641.63964	57.12	1044
57.13	594.63964	57.13	1021
57.14	591.63964	57.14	1046
57.15	612.63964	57.15	1081
57.16	612.63964	57.16	1053
57.17	657.63964	57.17	1044
57.18	574.63964	57.18	1035
57.19	588.63964	57.19	1019
57.2	604.63964	57.2	1078
57.21	548.63964	57.21	1039
57.22	588.63964	57.22	1048
57.23	627.63964	57.23	1060
57.24	622.63964	57.24	1060
57.25	600.63964	57.25	1056

57.26	632.63964	57.26	1028
57.27	585.63964	57.27	1061
57.28	521.63964	57.28	1039
57.29	610.63964	57.29	1017
57.3	621.63964	57.3	1048
57.31	630.63964	57.31	1067
57.32	597.63964	57.32	1089
57.33	647.63964	57.33	1060
57.34	640.63964	57.34	1049
57.35	584.63964	57.35	1035
57.36	646.63964	57.36	1038
57.37	593.63964	57.37	1048
57.38	580.63964	57.38	1006
57.39	581.63964	57.39	1005
57.4	604.63964	57.4	1040
57.41	654.63964	57.41	1072
57.42	573.63964	57.42	1042
57.43	577.63964	57.43	1050
57.44	580.63964	57.44	1067
57.45	557.63964	57.45	1025
57.46	642.63964	57.46	1035
57.47	566.63964	57.47	1049

57.48	552.63964	57.48	1035
57.49	599.63964	57.49	1063
57.5	619.63964	57.5	1065
57.51	582.63964	57.51	1053
57.52	607.63964	57.52	1057
57.53	596.63964	57.53	1045
57.54	571.63964	57.54	1045
57.55	581.63964	57.55	1063
57.56	583.63964	57.56	1043
57.57	617.63964	57.57	1028
57.58	591.63964	57.58	1045
57.59	552.63964	57.59	1033
57.6	605.63964	57.6	1045
57.61	620.63964	57.61	1028
57.62	636.63964	57.62	1021
57.63	592.63964	57.63	1069
57.64	619.63964	57.64	1060
57.65	564.63964	57.65	1072
57.66	609.63964	57.66	1027
57.67	585.63964	57.67	1021
57.68	588.63964	57.68	1040
57.69	579.63964	57.69	1061

57.7	587.63964	57.7	1017
57.71	609.63964	57.71	1051
57.72	597.63964	57.72	1034
57.73	608.63964	57.73	1068
57.74	591.63964	57.74	1050
57.75	563.63964	57.75	1016
57.76	600.63964	57.76	1038
57.77	605.63964	57.77	1032
57.78	616.63964	57.78	1025
57.79	613.63964	57.79	1042
57.8	592.63964	57.8	1049
57.81	579.63964	57.81	1076
57.82	566.63964	57.82	1037
57.83	564.63964	57.83	1057
57.84	577.63964	57.84	1035
57.85	633.63964	57.85	1033
57.86	567.63964	57.86	1051
57.87	587.63964	57.87	1023
57.88	590.63964	57.88	1019
57.89	594.63964	57.89	1006
57.9	617.63964	57.9	1012
57.91	581.63964	57.91	1041

57.92	592.63964	57.92	1063
57.93	543.63964	57.93	1030
57.94	560.63964	57.94	1037
57.95	587.63964	57.95	1032
57.96	553.63964	57.96	1024
57.97	612.63964	57.97	1023
57.98	538.63964	57.98	1040
57.99	584.63964	57.99	1056
58	618.63964	58	1047
58.01	613.63964	58.01	1009
58.02	624.63964	58.02	1021
58.03	620.63964	58.03	1045
58.04	563.63964	58.04	1042
58.05	596.63964	58.05	1049
58.06	566.63964	58.06	1045
58.07	563.63964	58.07	1035
58.08	577.63964	58.08	1053
58.09	551.63964	58.09	1024
58.1	574.63964	58.1	1059
58.11	592.63964	58.11	1032
58.12	574.63964	58.12	1043
58.13	644.63964	58.13	1056

58.14	613.63964	58.14	1037
58.15	606.63964	58.15	1045
58.16	572.63964	58.16	1050
58.17	580.63964	58.17	1042
58.18	571.63964	58.18	1046
58.19	545.63964	58.19	1040
58.2	578.63964	58.2	1037
58.21	561.63964	58.21	1035
58.22	600.63964	58.22	1014
58.23	648.63964	58.23	1041
58.24	652.63964	58.24	1041
58.25	599.63964	58.25	1042
58.26	611.63964	58.26	1050
58.27	614.63964	58.27	1029
58.28	622.63964	58.28	1044
58.29	600.63964	58.29	1057
58.3	555.63964	58.3	1068
58.31	612.63964	58.31	1045
58.32	594.63964	58.32	1030
58.33	625.63964	58.33	1043
58.34	590.63964	58.34	1025
58.35	596.63964	58.35	1027

58.36	627.63964	58.36	1020
58.37	612.63964	58.37	1030
58.38	607.63964	58.38	1050
58.39	577.63964	58.39	1051
58.4	580.63964	58.4	1053
58.41	620.63964	58.41	1051
58.42	594.63964	58.42	1034
58.43	607.63964	58.43	1044
58.44	649.63964	58.44	1061
58.45	638.63964	58.45	1051
58.46	630.63964	58.46	1063
58.47	640.63964	58.47	1054
58.48	647.63964	58.48	1051
58.49	647.63964	58.49	1033
58.5	618.63964	58.5	1027
58.51	654.63964	58.51	1041
58.52	652.63964	58.52	1042
58.53	663.63964	58.53	1049
58.54	632.63964	58.54	1100
58.55	630.63964	58.55	1097
58.56	624.63964	58.56	1065
58.57	642.63964	58.57	1067

58.58	599.63964	58.58	1045
58.59	624.63964	58.59	1034
58.6	667.63964	58.6	1059
58.61	664.63964	58.61	1068
58.62	647.63964	58.62	1077
58.63	606.63964	58.63	1030
58.64	647.63964	58.64	1054
58.65	661.63964	58.65	1066
58.66	627.63964	58.66	1056
58.67	631.63964	58.67	1040
58.68	616.63964	58.68	1063
58.69	672.63964	58.69	1070
58.7	664.63964	58.7	1059
58.71	658.63964	58.71	1046
58.72	667.63964	58.72	1064
58.73	638.63964	58.73	1090
58.74	637.63964	58.74	1064
58.75	658.63964	58.75	1084
58.76	625.63964	58.76	1064
58.77	651.63964	58.77	1061
58.78	642.63964	58.78	1061
58.79	652.63964	58.79	1047

58.8	673.63964	58.8	1087
58.81	650.63964	58.81	1037
58.82	661.63964	58.82	1086
58.83	723.63964	58.83	1038
58.84	691.63964	58.84	1040
58.85	661.63964	58.85	1057
58.86	656.63964	58.86	1063
58.87	633.63964	58.87	1063
58.88	638.63964	58.88	1084
58.89	680.63964	58.89	1073
58.9	691.63964	58.9	1087
58.91	678.63964	58.91	1120
58.92	672.63964	58.92	1056
58.93	683.63964	58.93	1032
58.94	647.63964	58.94	1105
58.95	703.63964	58.95	1087
58.96	710.63964	58.96	1102
58.97	644.63964	58.97	1063
58.98	684.63964	58.98	1081
58.99	691.63964	58.99	1080
59	688.63964	59	1056
59.01	627.63964	59.01	1076

59.02	672.63964	59.02	1060
59.03	641.63964	59.03	1083
59.04	662.63964	59.04	1081
59.05	633.63964	59.05	1079
59.06	670.63964	59.06	1065
59.07	690.63964	59.07	1049
59.08	694.63964	59.08	1071
59.09	647.63964	59.09	1084
59.1	601.63964	59.1	1086
59.11	677.63964	59.11	1065
59.12	681.63964	59.12	1079
59.13	670.63964	59.13	1089
59.14	677.63964	59.14	1098
59.15	700.63964	59.15	1083
59.16	770.63964	59.16	1063
59.17	714.63964	59.17	1099
59.18	707.63964	59.18	1122
59.19	690.63964	59.19	1101
59.2	713.63964	59.2	1084
59.21	714.63964	59.21	1076
59.22	681.63964	59.22	1120
59.23	685.63964	59.23	1078

59.24	662.63964	59.24	1078
59.25	732.63964	59.25	1117
59.26	671.63964	59.26	1067
59.27	671.63964	59.27	1065
59.28	696.63964	59.28	1090
59.29	724.63964	59.29	1067
59.3	726.63964	59.3	1080
59.31	735.63964	59.31	1081
59.32	748.63964	59.32	1100
59.33	747.63964	59.33	1107
59.34	738.63964	59.34	1077
59.35	687.63964	59.35	1065
59.36	675.63964	59.36	1073
59.37	687.63964	59.37	1091
59.38	682.63964	59.38	1106
59.39	699.63964	59.39	1076
59.4	689.63964	59.4	1103
59.41	716.63964	59.41	1100
59.42	746.63964	59.42	1099
59.43	710.63964	59.43	1123
59.44	720.63964	59.44	1060
59.45	657.63964	59.45	1081

59.46	703.63964	59.46	1075
59.47	762.63964	59.47	1099
59.48	734.63964	59.48	1066
59.49	740.63964	59.49	1070
59.5	689.63964	59.5	1091
59.51	710.63964	59.51	1095
59.52	704.63964	59.52	1071
59.53	704.63964	59.53	1111
59.54	720.63964	59.54	1080
59.55	711.63964	59.55	1069
59.56	691.63964	59.56	1105
59.57	667.63964	59.57	1065
59.58	646.63964	59.58	1053
59.59	681.63964	59.59	1039
59.6	703.63964	59.6	1076
59.61	651.63964	59.61	1099
59.62	631.63964	59.62	1060
59.63	687.63964	59.63	1046
59.64	681.63964	59.64	1076
59.65	740.63964	59.65	1080
59.66	693.63964	59.66	1079
59.67	676.63964	59.67	1054

59.68	640.63964	59.68	1096
59.69	627.63964	59.69	1067
59.7	643.63964	59.7	1078
59.71	649.63964	59.71	1060
59.72	664.63964	59.72	1043
59.73	667.63964	59.73	1057
59.74	660.63964	59.74	1063
59.75	639.63964	59.75	1077
59.76	635.63964	59.76	1064
59.77	644.63964	59.77	1048
59.78	621.63964	59.78	1058
59.79	615.63964	59.79	1081
59.8	626.63964	59.8	1057
59.81	676.63964	59.81	1054
59.82	619.63964	59.82	1045
59.83	662.63964	59.83	1007
59.84	615.63964	59.84	1042
59.85	664.63964	59.85	1049
59.86	632.63964	59.86	1042
59.87	622.63964	59.87	1047
59.88	586.63964	59.88	1040
59.89	594.63964	59.89	1075

59.9	606.63964	59.9	1051
59.91	632.63964	59.91	1042
59.92	644.63964	59.92	1027
59.93	604.63964	59.93	1087
59.94	619.63964	59.94	1035
59.95	661.63964	59.95	1058
59.96	571.63964	59.96	1056
59.97	619.63964	59.97	1043
59.98	589.63964	59.98	1060
59.99	598.63964	59.99	1047
60	633.63964	60	1005
60.01	607.63964	60.01	1023
60.02	626.63964	60.02	1039
60.03	639.63964	60.03	1041
60.04	592.63964	60.04	1029
60.05	589.63964	60.05	1030
60.06	598.63964	60.06	1027
60.07	593.63964	60.07	1030
60.08	588.63964	60.08	1017
60.09	601.63964	60.09	1042
60.1	587.63964	60.1	1047
60.11	615.63964	60.11	1008

60.12	534.63964	60.12	1024
60.13	588.63964	60.13	1059
60.14	560.63964	60.14	1024
60.15	556.63964	60.15	1063
60.16	594.63964	60.16	1046
60.17	573.63964	60.17	1059
60.18	584.63964	60.18	1013
60.19	579.63964	60.19	1027
60.2	605.63964	60.2	1075
60.21	571.63964	60.21	1052
60.22	622.63964	60.22	1037
60.23	591.63964	60.23	1040
60.24	551.63964	60.24	999
60.25	583.63964	60.25	1025
60.26	573.63964	60.26	995
60.27	557.63964	60.27	1012
60.28	557.63964	60.28	1050
60.29	605.63964	60.29	1040
60.3	603.63964	60.3	1051
60.31	570.63964	60.31	1030
60.32	544.63964	60.32	1011
60.33	546.63964	60.33	1054

60.34	549.63964	60.34	1016
60.35	562.63964	60.35	1012
60.36	596.63964	60.36	1049
60.37	575.63964	60.37	1020
60.38	532.63964	60.38	1029
60.39	525.63964	60.39	1005
60.4	594.63964	60.4	1008
60.41	519.63964	60.41	1012
60.42	595.63964	60.42	1051
60.43	555.63964	60.43	1035
60.44	527.63964	60.44	1070
60.45	565.63964	60.45	1035
60.46	572.63964	60.46	1028
60.47	607.63964	60.47	1022
60.48	535.63964	60.48	1014
60.49	608.63964	60.49	1025
60.5	564.63964	60.5	1065
60.51	527.63964	60.51	1013
60.52	566.63964	60.52	1048
60.53	598.63964	60.53	1007
60.54	601.63964	60.54	1026
60.55	605.63964	60.55	1034

60.56	537.63964	60.56	1025
60.57	596.63964	60.57	1015
60.58	597.63964	60.58	1050
60.59	548.63964	60.59	1038
60.6	586.63964	60.6	1021
60.61	620.63964	60.61	1041
60.62	598.63964	60.62	1041
60.63	570.63964	60.63	1043
60.64	578.63964	60.64	1043
60.65	553.63964	60.65	986
60.66	543.63964	60.66	1015
60.67	564.63964	60.67	1051
60.68	571.63964	60.68	1026
60.69	568.63964	60.69	1019
60.7	545.63964	60.7	1014
60.71	525.63964	60.71	1015
60.72	539.63964	60.72	1045
60.73	552.63964	60.73	1059
60.74	584.63964	60.74	1015
60.75	552.63964	60.75	1012
60.76	532.63964	60.76	1018
60.77	557.63964	60.77	1035

60.78	545.63964	60.78	1037
60.79	539.63964	60.79	1014
60.8	561.63964	60.8	1018
60.81	597.63964	60.81	1024
60.82	541.63964	60.82	1026
60.83	557.63964	60.83	1027
60.84	555.63964	60.84	1027
60.85	520.63964	60.85	1046
60.86	508.63964	60.86	995
60.87	561.63964	60.87	1027
60.88	565.63964	60.88	1032
60.89	539.63964	60.89	1049
60.9	548.63964	60.9	1034
60.91	530.63964	60.91	1025
60.92	568.63964	60.92	1039
60.93	558.63964	60.93	1015
60.94	569.63964	60.94	1033
60.95	596.63964	60.95	998
60.96	566.63964	60.96	1009
60.97	537.63964	60.97	1033
60.98	548.63964	60.98	991
60.99	546.63964	60.99	1007

61	554.63964	61	1001
61.01	552.63964	61.01	1033
61.02	580.63964	61.02	989
61.03	539.63964	61.03	1022
61.04	567.63964	61.04	1037
61.05	556.63964	61.05	1040
61.06	589.63964	61.06	1044
61.07	526.63964	61.07	1039
61.08	544.63964	61.08	1017
61.09	557.63964	61.09	1018
61.1	586.63964	61.1	1027
61.11	561.63964	61.11	1008
61.12	559.63964	61.12	1033
61.13	556.63964	61.13	1017
61.14	521.63964	61.14	1023
61.15	581.63964	61.15	1009
61.16	561.63964	61.16	1028
61.17	556.63964	61.17	1017
61.18	516.63964	61.18	1023
61.19	529.63964	61.19	1020
61.2	555.63964	61.2	979
61.21	525.63964	61.21	993

61.22	579.63964	61.22	1005
61.23	531.63964	61.23	1033
61.24	536.63964	61.24	1003
61.25	522.63964	61.25	1024
61.26	533.63964	61.26	1022
61.27	540.63964	61.27	1016
61.28	553.63964	61.28	1067
61.29	544.63964	61.29	1014
61.3	543.63964	61.3	1037
61.31	529.63964	61.31	1009
61.32	574.63964	61.32	1037
61.33	542.63964	61.33	1025
61.34	538.63964	61.34	1015
61.35	542.63964	61.35	1024
61.36	533.63964	61.36	965
61.37	539.63964	61.37	1012
61.38	521.63964	61.38	1013
61.39	542.63964	61.39	1041
61.4	537.63964	61.4	1043
61.41	584.63964	61.41	1015
61.42	555.63964	61.42	1052
61.43	547.63964	61.43	1012

61.44	546.63964	61.44	1022
61.45	532.63964	61.45	1027
61.46	569.63964	61.46	1016
61.47	535.63964	61.47	1026
61.48	527.63964	61.48	1009
61.49	587.63964	61.49	1042
61.5	571.63964	61.5	1020
61.51	530.63964	61.51	1027
61.52	530.63964	61.52	1040
61.53	486.63964	61.53	1000
61.54	559.63964	61.54	1019
61.55	566.63964	61.55	1031
61.56	541.63964	61.56	1021
61.57	507.63964	61.57	987
61.58	539.63964	61.58	997
61.59	552.63964	61.59	1014
61.6	552.63964	61.6	1000
61.61	528.63964	61.61	1013
61.62	520.63964	61.62	1026
61.63	571.63964	61.63	1000
61.64	577.63964	61.64	1002
61.65	531.63964	61.65	1006

61.66	572.63964	61.66	1042
61.67	529.63964	61.67	1012
61.68	533.63964	61.68	1020
61.69	590.63964	61.69	1004
61.7	521.63964	61.7	1044
61.71	549.63964	61.71	1005
61.72	535.63964	61.72	1018
61.73	520.63964	61.73	993
61.74	568.63964	61.74	1014
61.75	538.63964	61.75	1004
61.76	515.63964	61.76	1006
61.77	549.63964	61.77	1028
61.78	538.63964	61.78	1025
61.79	506.63964	61.79	997
61.8	566.63964	61.8	1014
61.81	567.63964	61.81	1031
61.82	544.63964	61.82	1040
61.83	522.63964	61.83	997
61.84	529.63964	61.84	991
61.85	546.63964	61.85	1020
61.86	523.63964	61.86	1020
61.87	557.63964	61.87	977

61.88	553.63964	61.88	1018
61.89	571.63964	61.89	1030
61.9	521.63964	61.9	1013
61.91	532.63964	61.91	1026
61.92	526.63964	61.92	1018
61.93	567.63964	61.93	1017
61.94	537.63964	61.94	971
61.95	545.63964	61.95	1006
61.96	552.63964	61.96	1008
61.97	566.63964	61.97	1009
61.98	517.63964	61.98	1021
61.99	538.63964	61.99	999
62	584.63964	62	985
62.01	503.63964	62.01	1024
62.02	543.63964	62.02	994
62.03	558.63964	62.03	1008
62.04	534.63964	62.04	1047
62.05	562.63964	62.05	1007
62.06	555.63964	62.06	997
62.07	543.63964	62.07	1005
62.08	579.63964	62.08	1030
62.09	546.63964	62.09	993

62.1	518.63964	62.1	1024
62.11	540.63964	62.11	1000
62.12	556.63964	62.12	981
62.13	521.63964	62.13	1025
62.14	553.63964	62.14	1014
62.15	532.63964	62.15	1006
62.16	570.63964	62.16	1028
62.17	562.63964	62.17	1035
62.18	520.63964	62.18	1027
62.19	556.63964	62.19	1004
62.2	541.63964	62.2	999
62.21	501.63964	62.21	1006
62.22	577.63964	62.22	1025
62.23	574.63964	62.23	1012
62.24	529.63964	62.24	1034
62.25	527.63964	62.25	1011
62.26	556.63964	62.26	1019
62.27	543.63964	62.27	1016
62.28	521.63964	62.28	1044
62.29	563.63964	62.29	1017
62.3	572.63964	62.3	992
62.31	562.63964	62.31	1016

62.32	571.63964	62.32	980
62.33	539.63964	62.33	1004
62.34	567.63964	62.34	1005
62.35	568.63964	62.35	1031
62.36	566.63964	62.36	1025
62.37	542.63964	62.37	1031
62.38	552.63964	62.38	1028
62.39	570.63964	62.39	1017
62.4	554.63964	62.4	1017
62.41	534.63964	62.41	1022
62.42	558.63964	62.42	1007
62.43	552.63964	62.43	1023
62.44	535.63964	62.44	1012
62.45	535.63964	62.45	1016
62.46	520.63964	62.46	1021
62.47	522.63964	62.47	1021
62.48	507.63964	62.48	1038
62.49	510.63964	62.49	1029
62.5	517.63964	62.5	1026
62.51	562.63964	62.51	1032
62.52	519.63964	62.52	1021
62.53	552.63964	62.53	1001

62.54	566.63964	62.54	1050
62.55	520.63964	62.55	1006
62.56	517.63964	62.56	1035
62.57	542.63964	62.57	987
62.58	538.63964	62.58	1001
62.59	530.63964	62.59	985
62.6	549.63964	62.6	1011
62.61	544.63964	62.61	1058
62.62	523.63964	62.62	1020
62.63	526.63964	62.63	1001
62.64	507.63964	62.64	1018
62.65	573.63964	62.65	1056
62.66	556.63964	62.66	1018
62.67	538.63964	62.67	1003
62.68	540.63964	62.68	1035
62.69	523.63964	62.69	1029
62.7	561.63964	62.7	998
62.71	547.63964	62.71	1028
62.72	551.63964	62.72	1024
62.73	518.63964	62.73	998
62.74	569.63964	62.74	1037
62.75	560.63964	62.75	1017

62.76	531.63964	62.76	993
62.77	573.63964	62.77	1015
62.78	526.63964	62.78	1013
62.79	555.63964	62.79	995
62.8	528.63964	62.8	1003
62.81	527.63964	62.81	1018
62.82	553.63964	62.82	998
62.83	554.63964	62.83	990
62.84	541.63964	62.84	1019
62.85	552.63964	62.85	1021
62.86	547.63964	62.86	1004
62.87	506.63964	62.87	1042
62.88	531.63964	62.88	1005
62.89	588.63964	62.89	1040
62.9	488.63964	62.9	1021
62.91	527.63964	62.91	999
62.92	558.63964	62.92	1012
62.93	571.63964	62.93	1044
62.94	523.63964	62.94	1030
62.95	537.63964	62.95	1020
62.96	509.63964	62.96	1020
62.97	515.63964	62.97	1047

62.98	546.63964	62.98	987
62.99	512.63964	62.99	1018
63	536.63964	63	1021
63.01	533.63964	63.01	1001
63.02	552.63964	63.02	992
63.03	530.63964	63.03	1012
63.04	551.63964	63.04	1025
63.05	518.63964	63.05	1023
63.06	533.63964	63.06	1047
63.07	554.63964	63.07	994
63.08	540.63964	63.08	1038
63.09	533.63964	63.09	1013
63.1	537.63964	63.1	1025
63.11	567.63964	63.11	1000
63.12	507.63964	63.12	1024
63.13	508.63964	63.13	992
63.14	516.63964	63.14	1003
63.15	531.63964	63.15	1016
63.16	531.63964	63.16	1007
63.17	554.63964	63.17	1007
63.18	546.63964	63.18	1000
63.19	526.63964	63.19	1020

63.2	553.63964	63.2	1043
63.21	508.63964	63.21	1024
63.22	533.63964	63.22	1003
63.23	546.63964	63.23	1024
63.24	560.63964	63.24	1037
63.25	540.63964	63.25	1016
63.26	520.63964	63.26	1022
63.27	556.63964	63.27	1002
63.28	558.63964	63.28	1037
63.29	530.63964	63.29	1008
63.3	578.63964	63.3	1023
63.31	549.63964	63.31	1029
63.32	585.63964	63.32	1067
63.33	533.63964	63.33	1030
63.34	507.63964	63.34	1045
63.35	511.63964	63.35	1023
63.36	626.63964	63.36	1045
63.37	519.63964	63.37	1005
63.38	500.63964	63.38	1019
63.39	536.63964	63.39	1017
63.4	497.63964	63.4	1049
63.41	546.63964	63.41	1033

63.42	560.63964	63.42	1039
63.43	505.63964	63.43	1007
63.44	539.63964	63.44	995
63.45	523.63964	63.45	1025
63.46	543.63964	63.46	1021
63.47	499.63964	63.47	1059
63.48	512.63964	63.48	1011
63.49	516.63964	63.49	1006
63.5	521.63964	63.5	1011
63.51	533.63964	63.51	1042
63.52	559.63964	63.52	1021
63.53	551.63964	63.53	1029
63.54	569.63964	63.54	1020
63.55	507.63964	63.55	1016
63.56	539.63964	63.56	1018
63.57	565.63964	63.57	1057
63.58	506.63964	63.58	1024
63.59	509.63964	63.59	1026
63.6	556.63964	63.6	1026
63.61	551.63964	63.61	1056
63.62	555.63964	63.62	1019
63.63	522.63964	63.63	1024

63.64	518.63964	63.64	1007
63.65	525.63964	63.65	1012
63.66	539.63964	63.66	1015
63.67	531.63964	63.67	1023
63.68	534.63964	63.68	1003
63.69	548.63964	63.69	1021
63.7	544.63964	63.7	1014
63.71	533.63964	63.71	1039
63.72	530.63964	63.72	1012
63.73	518.63964	63.73	1011
63.74	527.63964	63.74	1043
63.75	527.63964	63.75	995
63.76	519.63964	63.76	1035
63.77	555.63964	63.77	1002
63.78	552.63964	63.78	1030
63.79	540.63964	63.79	1025
63.8	522.63964	63.8	1006
63.81	535.63964	63.81	1009
63.82	565.63964	63.82	991
63.83	483.63964	63.83	1013
63.84	516.63964	63.84	1016
63.85	562.63964	63.85	1013

63.86	539.63964	63.86	988
63.87	522.63964	63.87	987
63.88	512.63964	63.88	1007
63.89	560.63964	63.89	1035
63.9	519.63964	63.9	1029
63.91	530.63964	63.91	1008
63.92	535.63964	63.92	1011
63.93	506.63964	63.93	1023
63.94	522.63964	63.94	1002
63.95	537.63964	63.95	1008
63.96	554.63964	63.96	1014
63.97	508.63964	63.97	1012
63.98	558.63964	63.98	1027
63.99	523.63964	63.99	1022
64	571.63964	64	991
64.01	563.63964	64.01	1021
64.02	529.63964	64.02	1003
64.03	547.63964	64.03	1028
64.04	533.63964	64.04	968
64.05	554.63964	64.05	972
64.06	532.63964	64.06	994
64.07	520.63964	64.07	992

64.08	523.63964	64.08	1016
64.09	546.63964	64.09	1004
64.1	517.63964	64.1	1008
64.11	541.63964	64.11	1022
64.12	514.63964	64.12	1017
64.13	526.63964	64.13	1026
64.14	555.63964	64.14	1023
64.15	516.63964	64.15	1024
64.16	537.63964	64.16	1020
64.17	539.63964	64.17	998
64.18	496.63964	64.18	1001
64.19	537.63964	64.19	1000
64.2	542.63964	64.2	996
64.21	501.63964	64.21	1025
64.22	519.63964	64.22	1009
64.23	564.63964	64.23	1041
64.24	506.63964	64.24	1014
64.25	520.63964	64.25	1027
64.26	542.63964	64.26	995
64.27	533.63964	64.27	991
64.28	491.63964	64.28	1021
64.29	530.63964	64.29	999

64.3	561.63964	64.3	1008
64.31	499.63964	64.31	1016
64.32	542.63964	64.32	1042
64.33	551.63964	64.33	1011
64.34	565.63964	64.34	1024
64.35	537.63964	64.35	994
64.36	540.63964	64.36	1021
64.37	549.63964	64.37	1020
64.38	552.63964	64.38	998
64.39	537.63964	64.39	987
64.4	564.63964	64.4	998
64.41	545.63964	64.41	999
64.42	500.63964	64.42	1023
64.43	547.63964	64.43	1007
64.44	516.63964	64.44	1018
64.45	586.63964	64.45	1000
64.46	592.63964	64.46	1005
64.47	527.63964	64.47	1004
64.48	497.63964	64.48	1018
64.49	542.63964	64.49	993
64.5	532.63964	64.5	994
64.51	555.63964	64.51	1011

64.52	539.63964	64.52	1018
64.53	508.63964	64.53	1017
64.54	529.63964	64.54	1046
64.55	531.63964	64.55	981
64.56	538.63964	64.56	992
64.57	509.63964	64.57	985
64.58	497.63964	64.58	988
64.59	534.63964	64.59	1019
64.6	548.63964	64.6	1011
64.61	559.63964	64.61	1008
64.62	555.63964	64.62	995
64.63	502.63964	64.63	969
64.64	528.63964	64.64	1004
64.65	529.63964	64.65	1030
64.66	564.63964	64.66	981
64.67	518.63964	64.67	1021
64.68	574.63964	64.68	989
64.69	502.63964	64.69	996
64.7	553.63964	64.7	1049
64.71	505.63964	64.71	998
64.72	530.63964	64.72	1026
64.73	558.63964	64.73	1029

64.74	531.63964	64.74	1051
64.75	545.63964	64.75	1040
64.76	520.63964	64.76	1033
64.77	513.63964	64.77	1018
64.78	584.63964	64.78	1026
64.79	504.63964	64.79	1007
64.8	556.63964	64.8	991
64.81	538.63964	64.81	1034
64.82	532.63964	64.82	1027
64.83	528.63964	64.83	1023
64.84	495.63964	64.84	1002
64.85	521.63964	64.85	1011
64.86	511.63964	64.86	1021
64.87	514.63964	64.87	1018
64.88	503.63964	64.88	1013
64.89	525.63964	64.89	1004
64.9	533.63964	64.9	1011
64.91	505.63964	64.91	1023
64.92	536.63964	64.92	1002
64.93	516.63964	64.93	998
64.94	532.63964	64.94	1016
64.95	518.63964	64.95	999

64.96	510.63964	64.96	1012
64.97	512.63964	64.97	988
64.98	543.63964	64.98	1031
64.99	510.63964	64.99	1023
65	559.63964	65	1028
65.01	537.63964	65.01	998
65.02	514.63964	65.02	996
65.03	529.63964	65.03	1040
65.04	509.63964	65.04	992
65.05	554.63964	65.05	1018
65.06	526.63964	65.06	1013
65.07	513.63964	65.07	1030
65.08	515.63964	65.08	993
65.09	511.63964	65.09	1022
65.1	565.63964	65.1	1034
65.11	523.63964	65.11	1000
65.12	484.63964	65.12	992
65.13	513.63964	65.13	1022
65.14	546.63964	65.14	1002
65.15	540.63964	65.15	992
65.16	511.63964	65.16	999
65.17	507.63964	65.17	998

65.18	542.63964	65.18	1041
65.19	545.63964	65.19	1000
65.2	535.63964	65.2	1001
65.21	509.63964	65.21	1024
65.22	512.63964	65.22	1011
65.23	511.63964	65.23	1017
65.24	552.63964	65.24	1006
65.25	541.63964	65.25	1026
65.26	515.63964	65.26	1021
65.27	501.63964	65.27	1011
65.28	522.63964	65.28	1034
65.29	539.63964	65.29	1000
65.3	571.63964	65.3	1016
65.31	549.63964	65.31	1011
65.32	539.63964	65.32	1040
65.33	531.63964	65.33	987
65.34	554.63964	65.34	990
65.35	515.63964	65.35	991
65.36	542.63964	65.36	999
65.37	532.63964	65.37	1025
65.38	514.63964	65.38	1006
65.39	541.63964	65.39	1027

65.4	505.63964	65.4	997
65.41	499.63964	65.41	997
65.42	510.63964	65.42	1003
65.43	501.63964	65.43	1015
65.44	534.63964	65.44	992
65.45	529.63964	65.45	994
65.46	522.63964	65.46	1003
65.47	552.63964	65.47	994
65.48	527.63964	65.48	1022
65.49	493.63964	65.49	1004
65.5	537.63964	65.5	1004
65.51	554.63964	65.51	998
65.52	523.63964	65.52	1026
65.53	537.63964	65.53	1027
65.54	539.63964	65.54	1028
65.55	528.63964	65.55	1008
65.56	537.63964	65.56	1007
65.57	556.63964	65.57	1000
65.58	521.63964	65.58	972
65.59	545.63964	65.59	994
65.6	517.63964	65.6	1007
65.61	520.63964	65.61	994

65.62	537.63964	65.62	998
65.63	502.63964	65.63	1049
65.64	560.63964	65.64	1027
65.65	557.63964	65.65	983
65.66	539.63964	65.66	999
65.67	529.63964	65.67	1016
65.68	515.63964	65.68	997
65.69	535.63964	65.69	980
65.7	506.63964	65.7	1030
65.71	500.63964	65.71	1016
65.72	482.63964	65.72	995
65.73	530.63964	65.73	1005
65.74	500.63964	65.74	1015
65.75	507.63964	65.75	1006
65.76	497.63964	65.76	1014
65.77	509.63964	65.77	990
65.78	558.63964	65.78	1031
65.79	511.63964	65.79	1003
65.8	538.63964	65.8	996
65.81	543.63964	65.81	1019
65.82	511.63964	65.82	1002
65.83	521.63964	65.83	966

65.84	537.63964	65.84	1004
65.85	539.63964	65.85	1008
65.86	543.63964	65.86	1007
65.87	572.63964	65.87	986
65.88	561.63964	65.88	1012
65.89	518.63964	65.89	976
65.9	544.63964	65.9	998
65.91	532.63964	65.91	1014
65.92	526.63964	65.92	1013
65.93	511.63964	65.93	1047
65.94	542.63964	65.94	1009
65.95	513.63964	65.95	992
65.96	537.63964	65.96	1011
65.97	538.63964	65.97	1013
65.98	526.63964	65.98	970
65.99	496.63964	65.99	975
66	515.63964	66	1031
66.01	519.63964	66.01	1009
66.02	523.63964	66.02	981
66.03	519.63964	66.03	1022
66.04	474.63964	66.04	990
66.05	525.63964	66.05	1004

66.06	502.63964	66.06	1014
66.07	527.63964	66.07	992
66.08	511.63964	66.08	1001
66.09	525.63964	66.09	1017
66.1	535.63964	66.1	1013
66.11	476.63964	66.11	1013
66.12	509.63964	66.12	1014
66.13	520.63964	66.13	1008
66.14	531.63964	66.14	1016
66.15	511.63964	66.15	1001
66.16	507.63964	66.16	993
66.17	545.63964	66.17	1000
66.18	510.63964	66.18	994
66.19	540.63964	66.19	992
66.2	528.63964	66.2	987
66.21	526.63964	66.21	978
66.22	493.63964	66.22	998
66.23	529.63964	66.23	999
66.24	562.63964	66.24	1018
66.25	503.63964	66.25	1043
66.26	494.63964	66.26	1002
66.27	537.63964	66.27	1018

66.28	502.63964	66.28	1009
66.29	495.63964	66.29	1032
66.3	529.63964	66.3	1031
66.31	511.63964	66.31	1006
66.32	574.63964	66.32	1006
66.33	504.63964	66.33	1016
66.34	556.63964	66.34	1017
66.35	530.63964	66.35	1009
66.36	505.63964	66.36	987
66.37	507.63964	66.37	996
66.38	539.63964	66.38	1003
66.39	519.63964	66.39	974
66.4	514.63964	66.4	1011
66.41	531.63964	66.41	992
66.42	513.63964	66.42	1023
66.43	516.63964	66.43	1014
66.44	549.63964	66.44	1019
66.45	541.63964	66.45	1009
66.46	510.63964	66.46	1001
66.47	535.63964	66.47	995
66.48	533.63964	66.48	1014
66.49	558.63964	66.49	1011

66.5	516.63964	66.5	1008
66.51	525.63964	66.51	991
66.52	538.63964	66.52	1013
66.53	529.63964	66.53	1002
66.54	549.63964	66.54	1000
66.55	546.63964	66.55	1022
66.56	519.63964	66.56	985
66.57	505.63964	66.57	1012
66.58	519.63964	66.58	1022
66.59	489.63964	66.59	993
66.6	543.63964	66.6	977
66.61	526.63964	66.61	1020
66.62	474.63964	66.62	1005
66.63	512.63964	66.63	1004
66.64	467.63964	66.64	1017
66.65	520.63964	66.65	1005
66.66	585.63964	66.66	1017
66.67	567.63964	66.67	1024
66.68	561.63964	66.68	1006
66.69	551.63964	66.69	1025
66.7	515.63964	66.7	1027
66.71	493.63964	66.71	1006

66.72	534.63964	66.72	1007
66.73	533.63964	66.73	1003
66.74	509.63964	66.74	1005
66.75	541.63964	66.75	1025
66.76	506.63964	66.76	1024
66.77	505.63964	66.77	1002
66.78	534.63964	66.78	998
66.79	495.63964	66.79	987
66.8	546.63964	66.8	976
66.81	522.63964	66.81	1011
66.82	534.63964	66.82	1001
66.83	532.63964	66.83	1011
66.84	488.63964	66.84	1011
66.85	545.63964	66.85	995
66.86	504.63964	66.86	1030
66.87	536.63964	66.87	1018
66.88	508.63964	66.88	1029
66.89	536.63964	66.89	976
66.9	503.63964	66.9	998
66.91	493.63964	66.91	1015
66.92	514.63964	66.92	1019
66.93	504.63964	66.93	1009

66.94	519.63964	66.94	1021
66.95	528.63964	66.95	1055
66.96	500.63964	66.96	1038
66.97	567.63964	66.97	996
66.98	514.63964	66.98	1015
66.99	511.63964	66.99	995
67	582.63964	67	1021
67.01	502.63964	67.01	1008
67.02	537.63964	67.02	967
67.03	543.63964	67.03	995
67.04	513.63964	67.04	1003
67.05	536.63964	67.05	1028
67.06	509.63964	67.06	1017
67.07	504.63964	67.07	1011
67.08	511.63964	67.08	1003
67.09	515.63964	67.09	1013
67.1	512.63964	67.1	1037
67.11	530.63964	67.11	999
67.12	526.63964	67.12	978
67.13	537.63964	67.13	1019
67.14	538.63964	67.14	1016
67.15	555.63964	67.15	1041

67.16	532.63964	67.16	1025
67.17	523.63964	67.17	1011
67.18	562.63964	67.18	1035
67.19	537.63964	67.19	1028
67.2	499.63964	67.2	1014
67.21	527.63964	67.21	1013
67.22	531.63964	67.22	983
67.23	532.63964	67.23	1006
67.24	549.63964	67.24	1006
67.25	523.63964	67.25	1017
67.26	526.63964	67.26	1047
67.27	483.63964	67.27	983
67.28	569.63964	67.28	999
67.29	548.63964	67.29	997
67.3	519.63964	67.3	997
67.31	518.63964	67.31	1014
67.32	515.63964	67.32	1033
67.33	519.63964	67.33	1033
67.34	532.63964	67.34	972
67.35	515.63964	67.35	1015
67.36	528.63964	67.36	973
67.37	569.63964	67.37	977

67.38	527.63964	67.38	985
67.39	542.63964	67.39	1012
67.4	532.63964	67.4	997
67.41	520.63964	67.41	991
67.42	530.63964	67.42	1002
67.43	531.63964	67.43	1016
67.44	521.63964	67.44	1009
67.45	513.63964	67.45	1013
67.46	517.63964	67.46	987
67.47	517.63964	67.47	1023
67.48	548.63964	67.48	1000
67.49	570.63964	67.49	1013
67.5	563.63964	67.5	1003
67.51	543.63964	67.51	1022
67.52	526.63964	67.52	1018
67.53	579.63964	67.53	1022
67.54	575.63964	67.54	996
67.55	516.63964	67.55	1038
67.56	552.63964	67.56	1001
67.57	536.63964	67.57	989
67.58	506.63964	67.58	1025
67.59	564.63964	67.59	1013

67.6	495.63964	67.6	1011
67.61	548.63964	67.61	1033
67.62	549.63964	67.62	1017
67.63	492.63964	67.63	998
67.64	512.63964	67.64	979
67.65	536.63964	67.65	1001
67.66	555.63964	67.66	1014
67.67	523.63964	67.67	989
67.68	530.63964	67.68	1009
67.69	511.63964	67.69	1001
67.7	505.63964	67.7	1018
67.71	512.63964	67.71	1031
67.72	560.63964	67.72	969
67.73	487.63964	67.73	1016
67.74	501.63964	67.74	1009
67.75	541.63964	67.75	1032
67.76	532.63964	67.76	993
67.77	506.63964	67.77	1002
67.78	511.63964	67.78	1032
67.79	564.63964	67.79	1000
67.8	553.63964	67.8	1022
67.81	528.63964	67.81	989

67.82	532.63964	67.82	1016
67.83	546.63964	67.83	991
67.84	538.63964	67.84	979
67.85	509.63964	67.85	994
67.86	500.63964	67.86	989
67.87	518.63964	67.87	996
67.88	539.63964	67.88	1014
67.89	540.63964	67.89	1008
67.9	561.63964	67.9	1004
67.91	536.63964	67.91	1021
67.92	483.63964	67.92	1038
67.93	521.63964	67.93	1017
67.94	506.63964	67.94	991
67.95	548.63964	67.95	980
67.96	518.63964	67.96	1029
67.97	529.63964	67.97	993
67.98	489.63964	67.98	981
67.99	479.63964	67.99	1005
68	490.63964	68	991
68.01	539.63964	68.01	996
68.02	565.63964	68.02	1005
68.03	515.63964	68.03	967

68.04	515.63964	68.04	1015
68.05	549.63964	68.05	995
68.06	496.63964	68.06	989
68.07	553.63964	68.07	996
68.08	536.63964	68.08	1003
68.09	533.63964	68.09	992
68.1	536.63964	68.1	998
68.11	551.63964	68.11	1005
68.12	526.63964	68.12	1020
68.13	537.63964	68.13	996
68.14	544.63964	68.14	1000
68.15	529.63964	68.15	989
68.16	554.63964	68.16	982
68.17	536.63964	68.17	1028
68.18	547.63964	68.18	991
68.19	530.63964	68.19	993
68.2	508.63964	68.2	1024
68.21	478.63964	68.21	990
68.22	561.63964	68.22	998
68.23	528.63964	68.23	1039
68.24	540.63964	68.24	997
68.25	497.63964	68.25	995

68.26	520.63964	68.26	979
68.27	547.63964	68.27	991
68.28	531.63964	68.28	1024
68.29	543.63964	68.29	1033
68.3	535.63964	68.3	1003
68.31	569.63964	68.31	995
68.32	513.63964	68.32	1035
68.33	562.63964	68.33	978
68.34	588.63964	68.34	996
68.35	575.63964	68.35	985
68.36	537.63964	68.36	1002
68.37	510.63964	68.37	999
68.38	533.63964	68.38	1003
68.39	516.63964	68.39	996
68.4	495.63964	68.4	1007
68.41	566.63964	68.41	1018
68.42	542.63964	68.42	1027
68.43	499.63964	68.43	1004
68.44	502.63964	68.44	982
68.45	512.63964	68.45	1034
68.46	488.63964	68.46	971
68.47	534.63964	68.47	993

68.48	528.63964	68.48	1021
68.49	526.63964	68.49	1011
68.5	506.63964	68.5	1012
68.51	536.63964	68.51	999
68.52	507.63964	68.52	998
68.53	517.63964	68.53	1028
68.54	557.63964	68.54	1024
68.55	523.63964	68.55	978
68.56	539.63964	68.56	990
68.57	505.63964	68.57	1004
68.58	519.63964	68.58	1030
68.59	510.63964	68.59	1030
68.6	508.63964	68.6	990
68.61	557.63964	68.61	999
68.62	515.63964	68.62	1006
68.63	525.63964	68.63	1023
68.64	534.63964	68.64	993
68.65	500.63964	68.65	1000
68.66	531.63964	68.66	1032
68.67	557.63964	68.67	988
68.68	529.63964	68.68	1015
68.69	489.63964	68.69	977

68.7	495.63964	68.7	988
68.71	519.63964	68.71	989
68.72	492.63964	68.72	994
68.73	529.63964	68.73	989
68.74	549.63964	68.74	1021
68.75	559.63964	68.75	988
68.76	517.63964	68.76	1013
68.77	521.63964	68.77	1011
68.78	505.63964	68.78	1021
68.79	529.63964	68.79	985
68.8	506.63964	68.8	998
68.81	476.63964	68.81	994
68.82	560.63964	68.82	980
68.83	497.63964	68.83	1009
68.84	514.63964	68.84	1006
68.85	547.63964	68.85	1001
68.86	506.63964	68.86	1007
68.87	521.63964	68.87	1002
68.88	515.63964	68.88	1001
68.89	516.63964	68.89	1016
68.9	552.63964	68.9	1018
68.91	553.63964	68.91	1004

68.92	572.63964	68.92	999
68.93	521.63964	68.93	1013
68.94	538.63964	68.94	995
68.95	525.63964	68.95	989
68.96	561.63964	68.96	992
68.97	561.63964	68.97	1009
68.98	552.63964	68.98	1001
68.99	527.63964	68.99	1023
69	553.63964	69	969
69.01	530.63964	69.01	1011
69.02	561.63964	69.02	1015
69.03	521.63964	69.03	1022
69.04	513.63964	69.04	1018
69.05	509.63964	69.05	981
69.06	541.63964	69.06	996
69.07	522.63964	69.07	991
69.08	535.63964	69.08	972
69.09	563.63964	69.09	992
69.1	535.63964	69.1	981
69.11	544.63964	69.11	1009
69.12	510.63964	69.12	979
69.13	541.63964	69.13	997

69.14	504.63964	69.14	1013
69.15	496.63964	69.15	981
69.16	530.63964	69.16	999
69.17	507.63964	69.17	993
69.18	508.63964	69.18	1013
69.19	523.63964	69.19	999
69.2	518.63964	69.2	1006
69.21	505.63964	69.21	1005
69.22	553.63964	69.22	1003
69.23	531.63964	69.23	1001
69.24	496.63964	69.24	998
69.25	530.63964	69.25	1000
69.26	523.63964	69.26	1007
69.27	542.63964	69.27	980
69.28	543.63964	69.28	998
69.29	544.63964	69.29	1006
69.3	522.63964	69.3	982
69.31	535.63964	69.31	975
69.32	528.63964	69.32	998
69.33	555.63964	69.33	999
69.34	570.63964	69.34	1003
69.35	549.63964	69.35	977

69.36	546.63964	69.36	978
69.37	527.63964	69.37	983
69.38	549.63964	69.38	987
69.39	549.63964	69.39	997
69.4	464.63964	69.4	995
69.41	512.63964	69.41	999
69.42	555.63964	69.42	998
69.43	547.63964	69.43	985
69.44	532.63964	69.44	1023
69.45	555.63964	69.45	989
69.46	530.63964	69.46	1011
69.47	535.63964	69.47	988
69.48	558.63964	69.48	979
69.49	537.63964	69.49	955
69.5	539.63964	69.5	980
69.51	510.63964	69.51	1027
69.52	531.63964	69.52	1025
69.53	558.63964	69.53	988
69.54	528.63964	69.54	1017
69.55	523.63964	69.55	1018
69.56	529.63964	69.56	980
69.57	512.63964	69.57	1012

69.58	523.63964	69.58	991
69.59	523.63964	69.59	1021
69.6	510.63964	69.6	1017
69.61	508.63964	69.61	988
69.62	515.63964	69.62	1001
69.63	549.63964	69.63	1025
69.64	562.63964	69.64	1004
69.65	518.63964	69.65	968
69.66	556.63964	69.66	1015
69.67	560.63964	69.67	1017
69.68	522.63964	69.68	1009
69.69	518.63964	69.69	990
69.7	529.63964	69.7	1015
69.71	528.63964	69.71	1038
69.72	516.63964	69.72	1002
69.73	552.63964	69.73	989
69.74	520.63964	69.74	968
69.75	503.63964	69.75	995
69.76	496.63964	69.76	1003
69.77	497.63964	69.77	1005
69.78	516.63964	69.78	996
69.79	511.63964	69.79	1021

69.8	560.63964	69.8	1004
69.81	530.63964	69.81	1012
69.82	527.63964	69.82	989
69.83	522.63964	69.83	998
69.84	535.63964	69.84	979
69.85	504.63964	69.85	1028
69.86	473.63964	69.86	957
69.87	515.63964	69.87	1012
69.88	584.63964	69.88	986
69.89	535.63964	69.89	1009
69.9	514.63964	69.9	1003
69.91	548.63964	69.91	1009
69.92	536.63964	69.92	1015
69.93	521.63964	69.93	992
69.94	528.63964	69.94	1008
69.95	529.63964	69.95	999
69.96	527.63964	69.96	1018
69.97	548.63964	69.97	1008
69.98	526.63964	69.98	1009
69.99	493.63964	69.99	1008
70	512.63964	70	972
70.01	516.63964	70.01	994

70.02	497.63964	70.02	989
70.03	508.63964	70.03	995
70.04	486.63964	70.04	1003
70.05	505.63964	70.05	993
70.06	552.63964	70.06	1003
70.07	514.63964	70.07	998
70.08	517.63964	70.08	1042
70.09	531.63964	70.09	1023
70.1	555.63964	70.1	997
70.11	547.63964	70.11	1026
70.12	567.63964	70.12	1003
70.13	509.63964	70.13	1015
70.14	530.63964	70.14	1006
70.15	522.63964	70.15	1014
70.16	522.63964	70.16	1005
70.17	559.63964	70.17	1008
70.18	514.63964	70.18	1000
70.19	532.63964	70.19	1023
70.2	562.63964	70.2	1026
70.21	559.63964	70.21	1022
70.22	528.63964	70.22	1004
70.23	538.63964	70.23	995

70.24	563.63964	70.24	999
70.25	569.63964	70.25	976
70.26	536.63964	70.26	985
70.27	542.63964	70.27	1019
70.28	505.63964	70.28	1004
70.29	531.63964	70.29	961
70.3	544.63964	70.3	995
70.31	504.63964	70.31	986
70.32	516.63964	70.32	1011
70.33	515.63964	70.33	1008
70.34	514.63964	70.34	997
70.35	526.63964	70.35	990
70.36	516.63964	70.36	1005
70.37	505.63964	70.37	1012
70.38	537.63964	70.38	1017
70.39	532.63964	70.39	980
70.4	521.63964	70.4	997
70.41	485.63964	70.41	983
70.42	519.63964	70.42	983
70.43	503.63964	70.43	991
70.44	539.63964	70.44	977
70.45	555.63964	70.45	1017

70.46	518.63964	70.46	1018
70.47	537.63964	70.47	985
70.48	536.63964	70.48	1002
70.49	485.63964	70.49	993
70.5	489.63964	70.5	1015
70.51	535.63964	70.51	997
70.52	523.63964	70.52	1005
70.53	515.63964	70.53	969
70.54	486.63964	70.54	1007
70.55	508.63964	70.55	998
70.56	562.63964	70.56	990
70.57	511.63964	70.57	977
70.58	546.63964	70.58	978
70.59	545.63964	70.59	983
70.6	527.63964	70.6	993
70.61	562.63964	70.61	995
70.62	549.63964	70.62	1009
70.63	503.63964	70.63	1009
70.64	537.63964	70.64	995
70.65	473.63964	70.65	968
70.66	505.63964	70.66	1001
70.67	557.63964	70.67	982

70.68	536.63964	70.68	991
70.69	529.63964	70.69	995
70.7	515.63964	70.7	1013
70.71	506.63964	70.71	981
70.72	497.63964	70.72	998
70.73	505.63964	70.73	1019
70.74	516.63964	70.74	978
70.75	541.63964	70.75	988
70.76	553.63964	70.76	992
70.77	551.63964	70.77	997
70.78	538.63964	70.78	1021
70.79	500.63964	70.79	1012
70.8	534.63964	70.8	991
70.81	518.63964	70.81	1000
70.82	584.63964	70.82	993
70.83	547.63964	70.83	1007
70.84	499.63964	70.84	980
70.85	537.63964	70.85	987
70.86	493.63964	70.86	1014
70.87	509.63964	70.87	976
70.88	558.63964	70.88	994
70.89	521.63964	70.89	988

70.9	517.63964	70.9	968
70.91	467.63964	70.91	989
70.92	518.63964	70.92	1003
70.93	508.63964	70.93	1008
70.94	510.63964	70.94	981
70.95	540.63964	70.95	968
70.96	568.63964	70.96	1003
70.97	578.63964	70.97	968
70.98	554.63964	70.98	1000
70.99	556.63964	70.99	985
71	557.63964	71	968
71.01	558.63964	71.01	992
71.02	509.63964	71.02	969
71.03	551.63964	71.03	1012
71.04	518.63964	71.04	1020
71.05	546.63964	71.05	1002
71.06	543.63964	71.06	989
71.07	521.63964	71.07	1006
71.08	460.63964	71.08	991
71.09	517.63964	71.09	962
71.1	520.63964	71.1	991
71.11	540.63964	71.11	993

71.12	515.63964	71.12	996
71.13	496.63964	71.13	988
71.14	507.63964	71.14	975
71.15	529.63964	71.15	996
71.16	540.63964	71.16	980
71.17	526.63964	71.17	976
71.18	523.63964	71.18	964
71.19	506.63964	71.19	1003
71.2	507.63964	71.2	988
71.21	484.63964	71.21	1034
71.22	520.63964	71.22	983
71.23	547.63964	71.23	1005
71.24	525.63964	71.24	1000
71.25	466.63964	71.25	952
71.26	479.63964	71.26	1018
71.27	494.63964	71.27	986
71.28	518.63964	71.28	1002
71.29	517.63964	71.29	991
71.3	510.63964	71.3	981
71.31	514.63964	71.31	1013
71.32	530.63964	71.32	998
71.33	557.63964	71.33	1004

71.34	522.63964	71.34	1027
71.35	492.63964	71.35	996
71.36	500.63964	71.36	986
71.37	538.63964	71.37	993
71.38	500.63964	71.38	1005
71.39	510.63964	71.39	995
71.4	506.63964	71.4	988
71.41	503.63964	71.41	966
71.42	538.63964	71.42	1017
71.43	506.63964	71.43	1002
71.44	526.63964	71.44	996
71.45	509.63964	71.45	1004
71.46	521.63964	71.46	1018
71.47	541.63964	71.47	950
71.48	519.63964	71.48	963
71.49	478.63964	71.49	1011
71.5	510.63964	71.5	996
71.51	490.63964	71.51	1020
71.52	500.63964	71.52	971
71.53	483.63964	71.53	977
71.54	521.63964	71.54	967
71.55	529.63964	71.55	997

71.56	531.63964	71.56	970
71.57	505.63964	71.57	998
71.58	528.63964	71.58	1033
71.59	539.63964	71.59	1004
71.6	538.63964	71.6	988
71.61	542.63964	71.61	983
71.62	523.63964	71.62	978
71.63	487.63964	71.63	971
71.64	509.63964	71.64	1007
71.65	530.63964	71.65	1006
71.66	537.63964	71.66	1004
71.67	525.63964	71.67	990
71.68	463.63964	71.68	997
71.69	526.63964	71.69	988
71.7	511.63964	71.7	998
71.71	508.63964	71.71	995
71.72	529.63964	71.72	1001
71.73	528.63964	71.73	1016
71.74	526.63964	71.74	992
71.75	518.63964	71.75	989
71.76	517.63964	71.76	999
71.77	530.63964	71.77	996

71.78	525.63964	71.78	1011
71.79	490.63964	71.79	979
71.8	495.63964	71.8	967
71.81	449.63964	71.81	1002
71.82	488.63964	71.82	1014
71.83	482.63964	71.83	987
71.84	512.63964	71.84	1006
71.85	494.63964	71.85	982
71.86	474.63964	71.86	998
71.87	514.63964	71.87	1012
71.88	507.63964	71.88	985
71.89	510.63964	71.89	995
71.9	489.63964	71.9	1016
71.91	500.63964	71.91	1003
71.92	506.63964	71.92	991
71.93	502.63964	71.93	1005
71.94	506.63964	71.94	966
71.95	509.63964	71.95	995
71.96	514.63964	71.96	982
71.97	533.63964	71.97	975
71.98	513.63964	71.98	983
71.99	481.63964	71.99	979

72	526.63964	72	963
72.01	532.63964	72.01	978
72.02	534.63964	72.02	986
72.03	523.63964	72.03	1000
72.04	513.63964	72.04	999
72.05	476.63964	72.05	968
72.06	521.63964	72.06	1007
72.07	527.63964	72.07	997
72.08	539.63964	72.08	992
72.09	508.63964	72.09	998
72.1	494.63964	72.1	978
72.11	502.63964	72.11	987
72.12	526.63964	72.12	980
72.13	520.63964	72.13	1011
72.14	533.63964	72.14	1003
72.15	513.63964	72.15	989
72.16	514.63964	72.16	1009
72.17	516.63964	72.17	999
72.18	532.63964	72.18	1014
72.19	529.63964	72.19	1016
72.2	518.63964	72.2	983
72.21	520.63964	72.21	1005

72.22	490.63964	72.22	975
72.23	496.63964	72.23	1011
72.24	499.63964	72.24	997
72.25	510.63964	72.25	993
72.26	492.63964	72.26	979
72.27	485.63964	72.27	997
72.28	492.63964	72.28	989
72.29	485.63964	72.29	983
72.3	510.63964	72.3	1020
72.31	493.63964	72.31	1013
72.32	533.63964	72.32	975
72.33	507.63964	72.33	1003
72.34	562.63964	72.34	979
72.35	547.63964	72.35	978
72.36	521.63964	72.36	991
72.37	496.63964	72.37	1018
72.38	478.63964	72.38	1008
72.39	530.63964	72.39	987
72.4	521.63964	72.4	987
72.41	520.63964	72.41	1032
72.42	542.63964	72.42	987
72.43	515.63964	72.43	947

72.44	502.63964	72.44	979
72.45	482.63964	72.45	976
72.46	546.63964	72.46	975
72.47	476.63964	72.47	998
72.48	516.63964	72.48	972
72.49	476.63964	72.49	962
72.5	483.63964	72.5	980
72.51	497.63964	72.51	971
72.52	481.63964	72.52	985
72.53	474.63964	72.53	996
72.54	495.63964	72.54	978
72.55	483.63964	72.55	994
72.56	515.63964	72.56	991
72.57	542.63964	72.57	1021
72.58	513.63964	72.58	963
72.59	504.63964	72.59	978
72.6	487.63964	72.6	1015
72.61	511.63964	72.61	1020
72.62	499.63964	72.62	996
72.63	491.63964	72.63	998
72.64	505.63964	72.64	1022
72.65	508.63964	72.65	1009

72.66	481.63964	72.66	987
72.67	525.63964	72.67	996
72.68	515.63964	72.68	1013
72.69	517.63964	72.69	986
72.7	485.63964	72.7	999
72.71	500.63964	72.71	999
72.72	520.63964	72.72	959
72.73	523.63964	72.73	979
72.74	508.63964	72.74	994
72.75	520.63964	72.75	992
72.76	549.63964	72.76	972
72.77	466.63964	72.77	965
72.78	527.63964	72.78	961
72.79	501.63964	72.79	1013
72.8	534.63964	72.8	1005
72.81	487.63964	72.81	979
72.82	519.63964	72.82	991
72.83	458.63964	72.83	974
72.84	496.63964	72.84	985
72.85	532.63964	72.85	972
72.86	547.63964	72.86	987
72.87	478.63964	72.87	989

72.88	511.63964	72.88	980
72.89	508.63964	72.89	993
72.9	494.63964	72.9	971
72.91	500.63964	72.91	983
72.92	500.63964	72.92	996
72.93	497.63964	72.93	1007
72.94	528.63964	72.94	1003
72.95	551.63964	72.95	987
72.96	549.63964	72.96	1001
72.97	481.63964	72.97	998
72.98	504.63964	72.98	1009
72.99	500.63964	72.99	999
73	561.63964	73	973
73.01	545.63964	73.01	1014
73.02	520.63964	73.02	990
73.03	475.63964	73.03	988
73.04	527.63964	73.04	982
73.05	531.63964	73.05	1005
73.06	501.63964	73.06	979
73.07	521.63964	73.07	1008
73.08	483.63964	73.08	1008
73.09	506.63964	73.09	1001

73.1	509.63964	73.1	1005
73.11	509.63964	73.11	973
73.12	482.63964	73.12	985
73.13	517.63964	73.13	985
73.14	531.63964	73.14	991
73.15	556.63964	73.15	999
73.16	519.63964	73.16	995
73.17	483.63964	73.17	1007
73.18	494.63964	73.18	1006
73.19	507.63964	73.19	994
73.2	507.63964	73.2	980
73.21	543.63964	73.21	981
73.22	521.63964	73.22	1032
73.23	491.63964	73.23	983
73.24	511.63964	73.24	996
73.25	518.63964	73.25	989
73.26	518.63964	73.26	980
73.27	548.63964	73.27	985
73.28	520.63964	73.28	939
73.29	537.63964	73.29	980
73.3	497.63964	73.3	1021
73.31	480.63964	73.31	1023

73.32	488.63964	73.32	999
73.33	539.63964	73.33	1003
73.34	569.63964	73.34	992
73.35	531.63964	73.35	975
73.36	440.63964	73.36	982
73.37	507.63964	73.37	975
73.38	528.63964	73.38	1009
73.39	539.63964	73.39	981
73.4	477.63964	73.4	1017
73.41	545.63964	73.41	993
73.42	527.63964	73.42	1026
73.43	529.63964	73.43	978
73.44	529.63964	73.44	1029
73.45	504.63964	73.45	968
73.46	493.63964	73.46	973
73.47	507.63964	73.47	986
73.48	499.63964	73.48	996
73.49	502.63964	73.49	979
73.5	505.63964	73.5	973
73.51	480.63964	73.51	976
73.52	495.63964	73.52	950
73.53	518.63964	73.53	990

73.54	536.63964	73.54	981
73.55	512.63964	73.55	978
73.56	557.63964	73.56	983
73.57	538.63964	73.57	976
73.58	500.63964	73.58	1003
73.59	513.63964	73.59	999
73.6	506.63964	73.6	1004
73.61	493.63964	73.61	1022
73.62	518.63964	73.62	1015
73.63	492.63964	73.63	968
73.64	516.63964	73.64	1000
73.65	540.63964	73.65	988
73.66	540.63964	73.66	960
73.67	568.63964	73.67	978
73.68	511.63964	73.68	986
73.69	563.63964	73.69	990
73.7	536.63964	73.7	977
73.71	513.63964	73.71	976
73.72	540.63964	73.72	1001
73.73	479.63964	73.73	1003
73.74	516.63964	73.74	1003
73.75	525.63964	73.75	994

73.76	512.63964	73.76	977
73.77	536.63964	73.77	1017
73.78	529.63964	73.78	1008
73.79	523.63964	73.79	985
73.8	530.63964	73.8	997
73.81	513.63964	73.81	1019
73.82	553.63964	73.82	1018
73.83	512.63964	73.83	969
73.84	522.63964	73.84	994
73.85	487.63964	73.85	987
73.86	513.63964	73.86	997
73.87	553.63964	73.87	1000
73.88	514.63964	73.88	970
73.89	538.63964	73.89	1013
73.9	517.63964	73.9	976
73.91	491.63964	73.91	999
73.92	557.63964	73.92	987
73.93	537.63964	73.93	1012
73.94	503.63964	73.94	977
73.95	517.63964	73.95	994
73.96	547.63964	73.96	986
73.97	510.63964	73.97	988

73.98	553.63964	73.98	1014
73.99	567.63964	73.99	1016
74	539.63964	74	1016
74.01	529.63964	74.01	1004
74.02	522.63964	74.02	981
74.03	520.63964	74.03	1006
74.04	551.63964	74.04	997
74.05	529.63964	74.05	993
74.06	563.63964	74.06	982
74.07	513.63964	74.07	977
74.08	527.63964	74.08	995
74.09	517.63964	74.09	983
74.1	536.63964	74.1	1004
74.11	514.63964	74.11	985
74.12	547.63964	74.12	1000
74.13	542.63964	74.13	999
74.14	555.63964	74.14	998
74.15	547.63964	74.15	1002
74.16	502.63964	74.16	990
74.17	508.63964	74.17	987
74.18	548.63964	74.18	1027
74.19	519.63964	74.19	1017

74.2	509.63964	74.2	1023
74.21	523.63964	74.21	972
74.22	533.63964	74.22	993
74.23	540.63964	74.23	996
74.24	519.63964	74.24	986
74.25	543.63964	74.25	997
74.26	557.63964	74.26	999
74.27	518.63964	74.27	1021
74.28	520.63964	74.28	1009
74.29	531.63964	74.29	987
74.3	507.63964	74.3	988
74.31	516.63964	74.31	989
74.32	532.63964	74.32	1008
74.33	549.63964	74.33	980
74.34	526.63964	74.34	998
74.35	505.63964	74.35	985
74.36	509.63964	74.36	989
74.37	542.63964	74.37	961
74.38	556.63964	74.38	1015
74.39	548.63964	74.39	991
74.4	549.63964	74.4	1009
74.41	527.63964	74.41	1003

74.42	500.63964	74.42	1020
74.43	513.63964	74.43	1001
74.44	541.63964	74.44	993
74.45	563.63964	74.45	970
74.46	548.63964	74.46	982
74.47	529.63964	74.47	965
74.48	512.63964	74.48	996
74.49	528.63964	74.49	978
74.5	527.63964	74.5	983
74.51	488.63964	74.51	985
74.52	482.63964	74.52	979
74.53	501.63964	74.53	979
74.54	476.63964	74.54	969
74.55	454.63964	74.55	996
74.56	510.63964	74.56	976
74.57	514.63964	74.57	966
74.58	504.63964	74.58	991
74.59	574.63964	74.59	972
74.6	543.63964	74.6	994
74.61	543.63964	74.61	972
74.62	503.63964	74.62	999
74.63	530.63964	74.63	969

74.64	539.63964	74.64	995
74.65	545.63964	74.65	1026
74.66	513.63964	74.66	987
74.67	560.63964	74.67	1000
74.68	516.63964	74.68	985
74.69	507.63964	74.69	1005
74.7	531.63964	74.7	1032
74.71	512.63964	74.71	998
74.72	502.63964	74.72	989
74.73	551.63964	74.73	997
74.74	469.63964	74.74	1002
74.75	494.63964	74.75	978
74.76	516.63964	74.76	992
74.77	471.63964	74.77	992
74.78	483.63964	74.78	959
74.79	516.63964	74.79	973
74.8	511.63964	74.8	998
74.81	529.63964	74.81	1000
74.82	509.63964	74.82	940
74.83	533.63964	74.83	977
74.84	511.63964	74.84	979
74.85	549.63964	74.85	969

74.86	515.63964	74.86	1001
74.87	492.63964	74.87	985
74.88	511.63964	74.88	980
74.89	505.63964	74.89	980
74.9	516.63964	74.9	980
74.91	515.63964	74.91	1009
74.92	492.63964	74.92	993
74.93	494.63964	74.93	982
74.94	509.63964	74.94	991
74.95	510.63964	74.95	990
74.96	504.63964	74.96	1014
74.97	512.63964	74.97	985
74.98	560.63964	74.98	1012
74.99	523.63964	74.99	1006
75	522.63964	75	1008
75.01	529.63964	75.01	1006
75.02	515.63964	75.02	998
75.03	503.63964	75.03	1013
75.04	470.63964	75.04	969
75.05	517.63964	75.05	981
75.06	516.63964	75.06	978
75.07	520.63964	75.07	965

75.08	533.63964	75.08	1006
75.09	506.63964	75.09	973
75.1	521.63964	75.1	988
75.11	508.63964	75.11	979
75.12	473.63964	75.12	999
75.13	470.63964	75.13	1005
75.14	492.63964	75.14	969
75.15	481.63964	75.15	987
75.16	492.63964	75.16	965
75.17	531.63964	75.17	986
75.18	507.63964	75.18	992
75.19	462.63964	75.19	964
75.2	493.63964	75.2	975
75.21	525.63964	75.21	994
75.22	520.63964	75.22	1000
75.23	493.63964	75.23	988
75.24	502.63964	75.24	1002
75.25	506.63964	75.25	991
75.26	522.63964	75.26	987
75.27	521.63964	75.27	992
75.28	523.63964	75.28	963
75.29	527.63964	75.29	992

75.3	513.63964	75.3	988
75.31	504.63964	75.31	994
75.32	518.63964	75.32	993
75.33	507.63964	75.33	986
75.34	510.63964	75.34	996
75.35	515.63964	75.35	982
75.36	502.63964	75.36	968
75.37	493.63964	75.37	1025
75.38	541.63964	75.38	990
75.39	497.63964	75.39	1000
75.4	539.63964	75.4	999
75.41	545.63964	75.41	996
75.42	517.63964	75.42	993
75.43	511.63964	75.43	982
75.44	528.63964	75.44	978
75.45	489.63964	75.45	980
75.46	503.63964	75.46	964
75.47	512.63964	75.47	970
75.48	517.63964	75.48	1001
75.49	491.63964	75.49	977
75.5	521.63964	75.5	966
75.51	493.63964	75.51	986

75.52	538.63964	75.52	995
75.53	493.63964	75.53	992
75.54	468.63964	75.54	992
75.55	505.63964	75.55	1006
75.56	513.63964	75.56	1011
75.57	538.63964	75.57	950
75.58	529.63964	75.58	987
75.59	500.63964	75.59	972
75.6	510.63964	75.6	995
75.61	533.63964	75.61	974
75.62	528.63964	75.62	973
75.63	490.63964	75.63	980
75.64	503.63964	75.64	977
75.65	510.63964	75.65	985
75.66	538.63964	75.66	989
75.67	474.63964	75.67	975
75.68	486.63964	75.68	989
75.69	526.63964	75.69	976
75.7	489.63964	75.7	976
75.71	507.63964	75.71	983
75.72	517.63964	75.72	1004
75.73	519.63964	75.73	970

75.74	483.63964	75.74	1001
75.75	553.63964	75.75	961
75.76	484.63964	75.76	993
75.77	455.63964	75.77	982
75.78	504.63964	75.78	979
75.79	499.63964	75.79	968
75.8	497.63964	75.8	970
75.81	495.63964	75.81	996
75.82	487.63964	75.82	992
75.83	512.63964	75.83	986
75.84	503.63964	75.84	969
75.85	496.63964	75.85	981
75.86	501.63964	75.86	996
75.87	492.63964	75.87	1017
75.88	516.63964	75.88	987
75.89	508.63964	75.89	981
75.9	540.63964	75.9	989
75.91	478.63964	75.91	989
75.92	473.63964	75.92	987
75.93	525.63964	75.93	989
75.94	530.63964	75.94	987
75.95	533.63964	75.95	986

75.96	492.63964	75.96	981
75.97	477.63964	75.97	1019
75.98	475.63964	75.98	981
75.99	536.63964	75.99	981
76	485.63964	76	987
76.01	491.63964	76.01	1013
76.02	530.63964	76.02	981
76.03	555.63964	76.03	957
76.04	494.63964	76.04	967
76.05	540.63964	76.05	977
76.06	514.63964	76.06	977
76.07	454.63964	76.07	995
76.08	514.63964	76.08	968
76.09	528.63964	76.09	964
76.1	531.63964	76.1	982
76.11	501.63964	76.11	977
76.12	501.63964	76.12	973
76.13	529.63964	76.13	953
76.14	478.63964	76.14	985
76.15	523.63964	76.15	991
76.16	507.63964	76.16	963
76.17	522.63964	76.17	943

76.18	509.63964	76.18	972
76.19	503.63964	76.19	992
76.2	516.63964	76.2	999
76.21	497.63964	76.21	983
76.22	479.63964	76.22	987
76.23	469.63964	76.23	995
76.24	483.63964	76.24	992
76.25	530.63964	76.25	998
76.26	518.63964	76.26	997
76.27	508.63964	76.27	957
76.28	486.63964	76.28	989
76.29	493.63964	76.29	1005
76.3	544.63964	76.3	1019
76.31	529.63964	76.31	981
76.32	519.63964	76.32	1003
76.33	529.63964	76.33	1009
76.34	522.63964	76.34	976
76.35	497.63964	76.35	991
76.36	497.63964	76.36	989
76.37	500.63964	76.37	1013
76.38	510.63964	76.38	1004
76.39	526.63964	76.39	991

76.4	521.63964	76.4	994
76.41	504.63964	76.41	1001
76.42	512.63964	76.42	955
76.43	505.63964	76.43	992
76.44	495.63964	76.44	995
76.45	514.63964	76.45	1022
76.46	507.63964	76.46	992
76.47	530.63964	76.47	1014
76.48	517.63964	76.48	976
76.49	558.63964	76.49	1000
76.5	493.63964	76.5	988
76.51	509.63964	76.51	993
76.52	514.63964	76.52	977
76.53	537.63964	76.53	1008
76.54	519.63964	76.54	985
76.55	500.63964	76.55	1007
76.56	505.63964	76.56	1004
76.57	510.63964	76.57	990
76.58	493.63964	76.58	966
76.59	566.63964	76.59	960
76.6	502.63964	76.6	981
76.61	499.63964	76.61	977

76.62	504.63964	76.62	951
76.63	507.63964	76.63	975
76.64	499.63964	76.64	1019
76.65	483.63964	76.65	973
76.66	542.63964	76.66	1009
76.67	503.63964	76.67	986
76.68	530.63964	76.68	977
76.69	515.63964	76.69	991
76.7	490.63964	76.7	978
76.71	492.63964	76.71	980
76.72	508.63964	76.72	1001
76.73	541.63964	76.73	996
76.74	529.63964	76.74	967
76.75	503.63964	76.75	1001
76.76	518.63964	76.76	995
76.77	504.63964	76.77	979
76.78	487.63964	76.78	963
76.79	505.63964	76.79	994
76.8	501.63964	76.8	964
76.81	520.63964	76.81	956
76.82	512.63964	76.82	966
76.83	492.63964	76.83	999

76.84	503.63964	76.84	973
76.85	504.63964	76.85	983
76.86	519.63964	76.86	998
76.87	502.63964	76.87	990
76.88	493.63964	76.88	985
76.89	540.63964	76.89	988
76.9	485.63964	76.9	977
76.91	500.63964	76.91	991
76.92	528.63964	76.92	964
76.93	514.63964	76.93	973
76.94	494.63964	76.94	989
76.95	533.63964	76.95	1007
76.96	484.63964	76.96	963
76.97	504.63964	76.97	968
76.98	518.63964	76.98	1018
76.99	546.63964	76.99	987
77	556.63964	77	1009
77.01	501.63964	77.01	988
77.02	470.63964	77.02	1002
77.03	475.63964	77.03	1018
77.04	511.63964	77.04	981
77.05	526.63964	77.05	981

77.06	547.63964	77.06	989
77.07	531.63964	77.07	986
77.08	514.63964	77.08	977
77.09	515.63964	77.09	1001
77.1	486.63964	77.1	1000
77.11	522.63964	77.11	979
77.12	477.63964	77.12	991
77.13	514.63964	77.13	1012
77.14	503.63964	77.14	982
77.15	516.63964	77.15	1001
77.16	528.63964	77.16	975
77.17	514.63964	77.17	996
77.18	520.63964	77.18	993
77.19	527.63964	77.19	979
77.2	462.63964	77.2	983
77.21	513.63964	77.21	974
77.22	522.63964	77.22	988
77.23	509.63964	77.23	996
77.24	530.63964	77.24	981
77.25	530.63964	77.25	985
77.26	520.63964	77.26	985
77.27	522.63964	77.27	981

77.28	516.63964	77.28	986
77.29	546.63964	77.29	1002
77.3	514.63964	77.3	1007
77.31	505.63964	77.31	989
77.32	534.63964	77.32	949
77.33	514.63964	77.33	963
77.34	497.63964	77.34	965
77.35	519.63964	77.35	975
77.36	514.63964	77.36	961
77.37	488.63964	77.37	992
77.38	521.63964	77.38	1013
77.39	490.63964	77.39	976
77.4	495.63964	77.4	996
77.41	529.63964	77.41	976
77.42	534.63964	77.42	972
77.43	558.63964	77.43	965
77.44	561.63964	77.44	988
77.45	535.63964	77.45	990
77.46	534.63964	77.46	1019
77.47	491.63964	77.47	988
77.48	500.63964	77.48	1014
77.49	507.63964	77.49	1038

77.5	503.63964	77.5	982
77.51	532.63964	77.51	983
77.52	520.63964	77.52	1013
77.53	520.63964	77.53	1027
77.54	485.63964	77.54	992
77.55	488.63964	77.55	1015
77.56	521.63964	77.56	970
77.57	500.63964	77.57	968
77.58	534.63964	77.58	969
77.59	556.63964	77.59	981
77.6	518.63964	77.6	990
77.61	528.63964	77.61	1001
77.62	466.63964	77.62	1031
77.63	522.63964	77.63	973
77.64	568.63964	77.64	1003
77.65	531.63964	77.65	1003
77.66	549.63964	77.66	977
77.67	510.63964	77.67	971
77.68	508.63964	77.68	997
77.69	532.63964	77.69	996
77.7	543.63964	77.7	990
77.71	546.63964	77.71	975

77.72	552.63964	77.72	989
77.73	493.63964	77.73	963
77.74	527.63964	77.74	973
77.75	525.63964	77.75	986
77.76	523.63964	77.76	979
77.77	485.63964	77.77	985
77.78	509.63964	77.78	980
77.79	523.63964	77.79	967
77.8	528.63964	77.8	997
77.81	499.63964	77.81	962
77.82	490.63964	77.82	1020
77.83	541.63964	77.83	963
77.84	527.63964	77.84	988
77.85	477.63964	77.85	986
77.86	529.63964	77.86	960
77.87	567.63964	77.87	988
77.88	520.63964	77.88	987
77.89	526.63964	77.89	970
77.9	479.63964	77.9	986
77.91	464.63964	77.91	988
77.92	479.63964	77.92	963
77.93	532.63964	77.93	964

77.94	515.63964	77.94	992
77.95	501.63964	77.95	985
77.96	529.63964	77.96	989
77.97	523.63964	77.97	966
77.98	499.63964	77.98	960
77.99	516.63964	77.99	974
78	508.63964	78	971
78.01	502.63964	78.01	990
78.02	534.63964	78.02	966
78.03	544.63964	78.03	981
78.04	538.63964	78.04	1001
78.05	545.63964	78.05	988
78.06	536.63964	78.06	980
78.07	491.63964	78.07	970
78.08	522.63964	78.08	976
78.09	496.63964	78.09	972
78.1	505.63964	78.1	973
78.11	500.63964	78.11	986
78.12	515.63964	78.12	981
78.13	543.63964	78.13	1009
78.14	528.63964	78.14	1007
78.15	489.63964	78.15	991

78.16	485.63964	78.16	966
78.17	518.63964	78.17	981
78.18	525.63964	78.18	1006
78.19	522.63964	78.19	981
78.2	519.63964	78.2	989
78.21	499.63964	78.21	985
78.22	542.63964	78.22	1007
78.23	520.63964	78.23	977
78.24	515.63964	78.24	1006
78.25	528.63964	78.25	1000
78.26	512.63964	78.26	1001
78.27	513.63964	78.27	990
78.28	504.63964	78.28	975
78.29	546.63964	78.29	942
78.3	487.63964	78.3	982
78.31	493.63964	78.31	996
78.32	520.63964	78.32	1005
78.33	523.63964	78.33	980
78.34	486.63964	78.34	955
78.35	485.63964	78.35	1020
78.36	501.63964	78.36	979
78.37	518.63964	78.37	987

78.38	513.63964	78.38	1009
78.39	551.63964	78.39	995
78.4	507.63964	78.4	1016
78.41	501.63964	78.41	977
78.42	493.63964	78.42	996
78.43	535.63964	78.43	995
78.44	505.63964	78.44	1011
78.45	512.63964	78.45	961
78.46	510.63964	78.46	955
78.47	489.63964	78.47	966
78.48	508.63964	78.48	965
78.49	501.63964	78.49	1005
78.5	479.63964	78.5	968
78.51	496.63964	78.51	971
78.52	520.63964	78.52	989
78.53	469.63964	78.53	993
78.54	512.63964	78.54	977
78.55	527.63964	78.55	959
78.56	519.63964	78.56	971
78.57	505.63964	78.57	972
78.58	455.63964	78.58	962
78.59	509.63964	78.59	982

78.6	574.63964	78.6	992
78.61	536.63964	78.61	996
78.62	542.63964	78.62	990
78.63	521.63964	78.63	985
78.64	527.63964	78.64	986
78.65	495.63964	78.65	997
78.66	513.63964	78.66	978
78.67	549.63964	78.67	969
78.68	545.63964	78.68	955
78.69	510.63964	78.69	987
78.7	548.63964	78.7	990
78.71	493.63964	78.71	992
78.72	536.63964	78.72	985
78.73	495.63964	78.73	982
78.74	548.63964	78.74	973
78.75	517.63964	78.75	980
78.76	528.63964	78.76	985
78.77	484.63964	78.77	960
78.78	528.63964	78.78	971
78.79	557.63964	78.79	990
78.8	502.63964	78.8	1005
78.81	496.63964	78.81	1001

78.82	518.63964	78.82	999
78.83	514.63964	78.83	961
78.84	538.63964	78.84	1001
78.85	504.63964	78.85	996
78.86	532.63964	78.86	971
78.87	476.63964	78.87	986
78.88	500.63964	78.88	1016
78.89	499.63964	78.89	974
78.9	520.63964	78.9	996
78.91	511.63964	78.91	990
78.92	495.63964	78.92	973
78.93	545.63964	78.93	991
78.94	530.63964	78.94	965
78.95	493.63964	78.95	998
78.96	483.63964	78.96	1011
78.97	543.63964	78.97	993
78.98	515.63964	78.98	976
78.99	478.63964	78.99	994
79	508.63964	79	978
79.01	514.63964	79.01	991
79.02	564.63964	79.02	983
79.03	552.63964	79.03	1001

79.04	508.63964	79.04	991
79.05	512.63964	79.05	987
79.06	545.63964	79.06	1002
79.07	565.63964	79.07	985
79.08	501.63964	79.08	991
79.09	528.63964	79.09	995
79.1	520.63964	79.1	972
79.11	534.63964	79.11	965
79.12	518.63964	79.12	992
79.13	543.63964	79.13	995
79.14	552.63964	79.14	1016
79.15	560.63964	79.15	998
79.16	515.63964	79.16	976
79.17	495.63964	79.17	991
79.18	536.63964	79.18	1012
79.19	526.63964	79.19	980
79.2	534.63964	79.2	980
79.21	501.63964	79.21	983
79.22	476.63964	79.22	980
79.23	457.63964	79.23	974
79.24	481.63964	79.24	1007
79.25	531.63964	79.25	1020

79.26	496.63964	79.26	1002
79.27	512.63964	79.27	986
79.28	523.63964	79.28	980
79.29	547.63964	79.29	1012
79.3	517.63964	79.3	990
79.31	526.63964	79.31	954
79.32	569.63964	79.32	946
79.33	529.63964	79.33	979
79.34	527.63964	79.34	965
79.35	528.63964	79.35	965
79.36	482.63964	79.36	1001
79.37	523.63964	79.37	1005
79.38	497.63964	79.38	974
79.39	536.63964	79.39	981
79.4	511.63964	79.4	996
79.41	486.63964	79.41	1011
79.42	541.63964	79.42	978
79.43	514.63964	79.43	960
79.44	505.63964	79.44	997
79.45	517.63964	79.45	974
79.46	516.63964	79.46	992
79.47	521.63964	79.47	978

79.48	486.63964	79.48	994
79.49	510.63964	79.49	968
79.5	523.63964	79.5	976
79.51	516.63964	79.51	955
79.52	532.63964	79.52	1006
79.53	530.63964	79.53	990
79.54	499.63964	79.54	995
79.55	515.63964	79.55	989
79.56	544.63964	79.56	1000
79.57	571.63964	79.57	1005
79.58	532.63964	79.58	976
79.59	546.63964	79.59	982
79.6	539.63964	79.6	953
79.61	519.63964	79.61	981
79.62	522.63964	79.62	989
79.63	544.63964	79.63	992
79.64	478.63964	79.64	977
79.65	467.63964	79.65	974
79.66	515.63964	79.66	987
79.67	500.63964	79.67	996
79.68	500.63964	79.68	954
79.69	510.63964	79.69	977

79.7	536.63964	79.7	989
79.71	493.63964	79.71	980
79.72	495.63964	79.72	963
79.73	543.63964	79.73	968
79.74	478.63964	79.74	978
79.75	489.63964	79.75	982
79.76	496.63964	79.76	1016
79.77	522.63964	79.77	972
79.78	513.63964	79.78	989
79.79	494.63964	79.79	972
79.8	525.63964	79.8	966
79.81	506.63964	79.81	997
79.82	490.63964	79.82	971
79.83	542.63964	79.83	990
79.84	548.63964	79.84	985
79.85	502.63964	79.85	987
79.86	463.63964	79.86	982
79.87	517.63964	79.87	954
79.88	516.63964	79.88	971
79.89	496.63964	79.89	971
79.9	490.63964	79.9	976
79.91	500.63964	79.91	979

79.92	537.63964	79.92	955
79.93	497.63964	79.93	998
79.94	489.63964	79.94	1003
79.95	494.63964	79.95	990
79.96	496.63964	79.96	985
79.97	476.63964	79.97	985
79.98	512.63964	79.98	992
79.99	483.63964	79.99	977
80	496.63964	80	959
80.01	526.63964	80.01	963
80.02	507.63964	80.02	987
80.03	527.63964	80.03	1029
80.04	493.63964	80.04	990
80.05	510.63964	80.05	1002
80.06	490.63964	80.06	1004
80.07	516.63964	80.07	995
80.08	548.63964	80.08	1008
80.09	531.63964	80.09	981
80.1	520.63964	80.1	992
80.11	477.63964	80.11	999
80.12	503.63964	80.12	990
80.13	511.63964	80.13	996

80.14	521.63964	80.14	1015
80.15	551.63964	80.15	995
80.16	521.63964	80.16	987
80.17	480.63964	80.17	969
80.18	482.63964	80.18	974
80.19	505.63964	80.19	997
80.2	503.63964	80.2	978
80.21	482.63964	80.21	969
80.22	523.63964	80.22	960
80.23	490.63964	80.23	967
80.24	526.63964	80.24	974
80.25	507.63964	80.25	965
80.26	489.63964	80.26	987
80.27	459.63964	80.27	980
80.28	485.63964	80.28	974
80.29	490.63964	80.29	993
80.3	505.63964	80.3	964
80.31	470.63964	80.31	963
80.32	519.63964	80.32	969
80.33	526.63964	80.33	950
80.34	514.63964	80.34	979
80.35	474.63964	80.35	992

80.36	503.63964	80.36	1012
80.37	489.63964	80.37	969
80.38	485.63964	80.38	970
80.39	458.63964	80.39	975
80.4	525.63964	80.4	980
80.41	491.63964	80.41	960
80.42	485.63964	80.42	972
80.43	512.63964	80.43	972
80.44	490.63964	80.44	995
80.45	534.63964	80.45	997
80.46	523.63964	80.46	1029
80.47	515.63964	80.47	1004
80.48	522.63964	80.48	980
80.49	512.63964	80.49	977
80.5	486.63964	80.5	1017
80.51	499.63964	80.51	976
80.52	526.63964	80.52	964
80.53	513.63964	80.53	972
80.54	494.63964	80.54	950
80.55	500.63964	80.55	982
80.56	464.63964	80.56	978
80.57	500.63964	80.57	998

80.58	478.63964	80.58	990
80.59	506.63964	80.59	985
80.6	548.63964	80.6	959
80.61	496.63964	80.61	972
80.62	459.63964	80.62	963
80.63	500.63964	80.63	973
80.64	490.63964	80.64	930
80.65	481.63964	80.65	959
80.66	504.63964	80.66	994
80.67	497.63964	80.67	975
80.68	499.63964	80.68	981
80.69	467.63964	80.69	1012
80.7	481.63964	80.7	994
80.71	542.63964	80.71	997
80.72	487.63964	80.72	1004
80.73	514.63964	80.73	976
80.74	516.63964	80.74	977
80.75	491.63964	80.75	990
80.76	492.63964	80.76	996
80.77	503.63964	80.77	971
80.78	475.63964	80.78	963
80.79	504.63964	80.79	963

80.8	503.63964	80.8	960
80.81	533.63964	80.81	997
80.82	506.63964	80.82	1014
80.83	544.63964	80.83	990
80.84	507.63964	80.84	974
80.85	549.63964	80.85	968
80.86	495.63964	80.86	954
80.87	486.63964	80.87	964
80.88	514.63964	80.88	974
80.89	473.63964	80.89	948
80.9	486.63964	80.9	975
80.91	514.63964	80.91	961
80.92	518.63964	80.92	973
80.93	520.63964	80.93	998
80.94	503.63964	80.94	965
80.95	464.63964	80.95	992
80.96	514.63964	80.96	985
80.97	517.63964	80.97	960
80.98	511.63964	80.98	953
80.99	494.63964	80.99	995
81	523.63964	81	979
81.01	511.63964	81.01	989

81.02	528.63964	81.02	989
81.03	478.63964	81.03	979
81.04	477.63964	81.04	963
81.05	483.63964	81.05	966
81.06	494.63964	81.06	970
81.07	502.63964	81.07	964
81.08	503.63964	81.08	973
81.09	493.63964	81.09	957
81.1	520.63964	81.1	975
81.11	518.63964	81.11	995
81.12	514.63964	81.12	963
81.13	515.63964	81.13	1007
81.14	520.63964	81.14	974
81.15	506.63964	81.15	965
81.16	457.63964	81.16	960
81.17	473.63964	81.17	970
81.18	479.63964	81.18	1000
81.19	502.63964	81.19	977
81.2	493.63964	81.2	988
81.21	482.63964	81.21	976
81.22	469.63964	81.22	992
81.23	515.63964	81.23	999

81.24	462.63964	81.24	973
81.25	509.63964	81.25	972
81.26	516.63964	81.26	980
81.27	520.63964	81.27	933
81.28	526.63964	81.28	948
81.29	492.63964	81.29	957
81.3	504.63964	81.3	975
81.31	512.63964	81.31	978
81.32	519.63964	81.32	977
81.33	526.63964	81.33	992
81.34	482.63964	81.34	995
81.35	503.63964	81.35	964
81.36	500.63964	81.36	955
81.37	505.63964	81.37	963
81.38	478.63964	81.38	991
81.39	492.63964	81.39	963
81.4	519.63964	81.4	967
81.41	533.63964	81.41	956
81.42	484.63964	81.42	990
81.43	475.63964	81.43	974
81.44	494.63964	81.44	982
81.45	501.63964	81.45	977

81.46	529.63964	81.46	985
81.47	505.63964	81.47	981
81.48	504.63964	81.48	957
81.49	521.63964	81.49	993
81.5	460.63964	81.5	975
81.51	487.63964	81.51	979
81.52	484.63964	81.52	953
81.53	507.63964	81.53	970
81.54	501.63964	81.54	972
81.55	506.63964	81.55	977
81.56	529.63964	81.56	971
81.57	489.63964	81.57	983
81.58	469.63964	81.58	960
81.59	506.63964	81.59	995
81.6	517.63964	81.6	971
81.61	515.63964	81.61	988
81.62	513.63964	81.62	997
81.63	505.63964	81.63	977
81.64	499.63964	81.64	941
81.65	497.63964	81.65	963
81.66	510.63964	81.66	966
81.67	542.63964	81.67	981

81.68	487.63964	81.68	967
81.69	554.63964	81.69	959
81.7	525.63964	81.7	965
81.71	486.63964	81.71	977
81.72	496.63964	81.72	959
81.73	507.63964	81.73	968
81.74	496.63964	81.74	952
81.75	478.63964	81.75	990
81.76	504.63964	81.76	970
81.77	489.63964	81.77	1001
81.78	488.63964	81.78	991
81.79	478.63964	81.79	976
81.8	528.63964	81.8	954
81.81	486.63964	81.81	992
81.82	494.63964	81.82	963
81.83	507.63964	81.83	963
81.84	497.63964	81.84	964
81.85	490.63964	81.85	968
81.86	473.63964	81.86	973
81.87	503.63964	81.87	972
81.88	469.63964	81.88	982
81.89	467.63964	81.89	981

81.9	487.63964	81.9	968
81.91	490.63964	81.91	953
81.92	490.63964	81.92	974
81.93	521.63964	81.93	972
81.94	504.63964	81.94	956
81.95	475.63964	81.95	974
81.96	502.63964	81.96	965
81.97	503.63964	81.97	972
81.98	519.63964	81.98	989
81.99	492.63964	81.99	977
82	474.63964	82	956
82.01	519.63964	82.01	970
82.02	495.63964	82.02	971
82.03	500.63964	82.03	939
82.04	510.63964	82.04	977
82.05	528.63964	82.05	920
82.06	499.63964	82.06	943
82.07	460.63964	82.07	990
82.08	506.63964	82.08	987
82.09	469.63964	82.09	975
82.1	517.63964	82.1	979
82.11	514.63964	82.11	971

82.12	513.63964	82.12	951
82.13	546.63964	82.13	977
82.14	502.63964	82.14	1012
82.15	476.63964	82.15	980
82.16	475.63964	82.16	972
82.17	511.63964	82.17	970
82.18	536.63964	82.18	980
82.19	487.63964	82.19	976
82.2	516.63964	82.2	962
82.21	506.63964	82.21	969
82.22	489.63964	82.22	972
82.23	504.63964	82.23	953
82.24	511.63964	82.24	961
82.25	499.63964	82.25	964
82.26	490.63964	82.26	950
82.27	491.63964	82.27	959
82.28	528.63964	82.28	959
82.29	447.63964	82.29	976
82.3	489.63964	82.3	938
82.31	505.63964	82.31	945
82.32	535.63964	82.32	966
82.33	511.63964	82.33	986

82.34	516.63964	82.34	994
82.35	496.63964	82.35	971
82.36	532.63964	82.36	995
82.37	490.63964	82.37	983
82.38	492.63964	82.38	965
82.39	489.63964	82.39	981
82.4	489.63964	82.4	989
82.41	508.63964	82.41	959
82.42	515.63964	82.42	963
82.43	514.63964	82.43	970
82.44	507.63964	82.44	985
82.45	485.63964	82.45	1003
82.46	490.63964	82.46	990
82.47	526.63964	82.47	965
82.48	514.63964	82.48	976
82.49	485.63964	82.49	975
82.5	464.63964	82.5	990
82.51	499.63964	82.51	962
82.52	485.63964	82.52	1001
82.53	485.63964	82.53	959
82.54	501.63964	82.54	971
82.55	530.63964	82.55	985

82.56	536.63964	82.56	997
82.57	543.63964	82.57	1000
82.58	497.63964	82.58	1009
82.59	491.63964	82.59	988
82.6	462.63964	82.6	988
82.61	488.63964	82.61	970
82.62	481.63964	82.62	965
82.63	490.63964	82.63	956
82.64	507.63964	82.64	979
82.65	534.63964	82.65	998
82.66	487.63964	82.66	974
82.67	513.63964	82.67	981
82.68	526.63964	82.68	985
82.69	475.63964	82.69	961
82.7	493.63964	82.7	975
82.71	503.63964	82.71	987
82.72	509.63964	82.72	1004
82.73	477.63964	82.73	1002
82.74	541.63964	82.74	940
82.75	529.63964	82.75	975
82.76	526.63964	82.76	942
82.77	480.63964	82.77	968

82.78	474.63964	82.78	946
82.79	475.63964	82.79	980
82.8	469.63964	82.8	951
82.81	476.63964	82.81	950
82.82	500.63964	82.82	1014
82.83	516.63964	82.83	975
82.84	489.63964	82.84	971
82.85	512.63964	82.85	962
82.86	475.63964	82.86	995
82.87	527.63964	82.87	981
82.88	523.63964	82.88	992
82.89	484.63964	82.89	998
82.9	491.63964	82.9	988
82.91	492.63964	82.91	985
82.92	566.63964	82.92	973
82.93	539.63964	82.93	985
82.94	506.63964	82.94	1011
82.95	523.63964	82.95	994
82.96	510.63964	82.96	947
82.97	496.63964	82.97	972
82.98	475.63964	82.98	962
82.99	476.63964	82.99	976

83	502.63964	83	981
83.01	496.63964	83.01	949
83.02	513.63964	83.02	1014
83.03	481.63964	83.03	961
83.04	504.63964	83.04	980
83.05	468.63964	83.05	962
83.06	458.63964	83.06	966
83.07	462.63964	83.07	1023
83.08	482.63964	83.08	996
83.09	482.63964	83.09	961
83.1	485.63964	83.1	981
83.11	502.63964	83.11	1001
83.12	508.63964	83.12	988
83.13	509.63964	83.13	994
83.14	519.63964	83.14	983
83.15	507.63964	83.15	964
83.16	526.63964	83.16	969
83.17	479.63964	83.17	990
83.18	503.63964	83.18	1017
83.19	512.63964	83.19	965
83.2	527.63964	83.2	995
83.21	555.63964	83.21	996

83.22	511.63964	83.22	956
83.23	489.63964	83.23	983
83.24	504.63964	83.24	976
83.25	501.63964	83.25	951
83.26	517.63964	83.26	965
83.27	506.63964	83.27	986
83.28	515.63964	83.28	993
83.29	484.63964	83.29	957
83.3	486.63964	83.3	975
83.31	484.63964	83.31	987
83.32	480.63964	83.32	981
83.33	514.63964	83.33	973
83.34	483.63964	83.34	960
83.35	475.63964	83.35	957
83.36	511.63964	83.36	989
83.37	539.63964	83.37	978
83.38	496.63964	83.38	957
83.39	484.63964	83.39	994
83.4	511.63964	83.4	981
83.41	513.63964	83.41	962
83.42	457.63964	83.42	960
83.43	474.63964	83.43	961

83.44	520.63964	83.44	999
83.45	438.63964	83.45	961
83.46	484.63964	83.46	959
83.47	468.63964	83.47	1012
83.48	495.63964	83.48	998
83.49	530.63964	83.49	972
83.5	466.63964	83.5	967
83.51	500.63964	83.51	971
83.52	494.63964	83.52	982
83.53	534.63964	83.53	985
83.54	522.63964	83.54	996
83.55	499.63964	83.55	979
83.56	463.63964	83.56	999
83.57	479.63964	83.57	973
83.58	519.63964	83.58	937
83.59	501.63964	83.59	975
83.6	434.63964	83.6	981
83.61	490.63964	83.61	970
83.62	492.63964	83.62	982
83.63	483.63964	83.63	946
83.64	491.63964	83.64	954
83.65	473.63964	83.65	967

83.66	520.63964	83.66	977
83.67	490.63964	83.67	978
83.68	478.63964	83.68	973
83.69	487.63964	83.69	972
83.7	509.63964	83.7	986
83.71	508.63964	83.71	981
83.72	476.63964	83.72	972
83.73	505.63964	83.73	981
83.74	503.63964	83.74	961
83.75	491.63964	83.75	977
83.76	493.63964	83.76	1005
83.77	502.63964	83.77	978
83.78	482.63964	83.78	1013
83.79	459.63964	83.79	976
83.8	482.63964	83.8	967
83.81	447.63964	83.81	992
83.82	476.63964	83.82	978
83.83	476.63964	83.83	998
83.84	471.63964	83.84	986
83.85	475.63964	83.85	962
83.86	489.63964	83.86	952
83.87	534.63964	83.87	962

83.88	488.63964	83.88	953
83.89	453.63964	83.89	975
83.9	463.63964	83.9	970
83.91	488.63964	83.91	962
83.92	523.63964	83.92	957
83.93	488.63964	83.93	985
83.94	506.63964	83.94	951
83.95	538.63964	83.95	947
83.96	484.63964	83.96	955
83.97	507.63964	83.97	979
83.98	500.63964	83.98	982
83.99	493.63964	83.99	968
84	497.63964	84	939
84.01	490.63964	84.01	959
84.02	489.63964	84.02	953
84.03	503.63964	84.03	957
84.04	505.63964	84.04	969
84.05	484.63964	84.05	975
84.06	518.63964	84.06	966
84.07	554.63964	84.07	987
84.08	455.63964	84.08	969
84.09	482.63964	84.09	986

84.1	515.63964	84.1	945
84.11	529.63964	84.11	993
84.12	484.63964	84.12	974
84.13	537.63964	84.13	976
84.14	473.63964	84.14	987
84.15	474.63964	84.15	974
84.16	489.63964	84.16	951
84.17	528.63964	84.17	969
84.18	485.63964	84.18	965
84.19	501.63964	84.19	972
84.2	495.63964	84.2	976
84.21	522.63964	84.21	972
84.22	503.63964	84.22	999
84.23	514.63964	84.23	997
84.24	525.63964	84.24	964
84.25	490.63964	84.25	974
84.26	507.63964	84.26	948
84.27	510.63964	84.27	973
84.28	520.63964	84.28	982
84.29	513.63964	84.29	997
84.3	480.63964	84.3	962
84.31	500.63964	84.31	978

84.32	478.63964	84.32	982
84.33	494.63964	84.33	963
84.34	512.63964	84.34	957
84.35	485.63964	84.35	974
84.36	481.63964	84.36	972
84.37	499.63964	84.37	948
84.38	534.63964	84.38	961
84.39	505.63964	84.39	983
84.4	449.63964	84.4	970
84.41	476.63964	84.41	961
84.42	459.63964	84.42	995
84.43	510.63964	84.43	969
84.44	494.63964	84.44	967
84.45	485.63964	84.45	974
84.46	499.63964	84.46	983
84.47	527.63964	84.47	955
84.48	497.63964	84.48	938
84.49	489.63964	84.49	978
84.5	527.63964	84.5	979
84.51	531.63964	84.51	952
84.52	475.63964	84.52	983
84.53	525.63964	84.53	956

84.54	483.63964	84.54	963
84.55	470.63964	84.55	971
84.56	476.63964	84.56	991
84.57	491.63964	84.57	972
84.58	521.63964	84.58	976
84.59	485.63964	84.59	977
84.6	518.63964	84.6	955
84.61	485.63964	84.61	949
84.62	485.63964	84.62	952
84.63	505.63964	84.63	948
84.64	500.63964	84.64	962
84.65	505.63964	84.65	975
84.66	500.63964	84.66	979
84.67	512.63964	84.67	950
84.68	533.63964	84.68	974
84.69	499.63964	84.69	989
84.7	480.63964	84.7	966
84.71	532.63964	84.71	1016
84.72	510.63964	84.72	976
84.73	483.63964	84.73	975
84.74	512.63964	84.74	956
84.75	515.63964	84.75	962

84.76	496.63964	84.76	988
84.77	453.63964	84.77	985
84.78	461.63964	84.78	978
84.79	481.63964	84.79	964
84.8	460.63964	84.8	957
84.81	522.63964	84.81	961
84.82	519.63964	84.82	973
84.83	473.63964	84.83	994
84.84	518.63964	84.84	978
84.85	473.63964	84.85	1005
84.86	462.63964	84.86	964
84.87	513.63964	84.87	970
84.88	480.63964	84.88	986
84.89	475.63964	84.89	992
84.9	511.63964	84.9	963
84.91	476.63964	84.91	955
84.92	489.63964	84.92	978
84.93	504.63964	84.93	962
84.94	475.63964	84.94	961
84.95	520.63964	84.95	983
84.96	556.63964	84.96	952
84.97	513.63964	84.97	976

84.98	487.63964	84.98	946
84.99	482.63964	84.99	979
85	506.63964	85	989
85.01	546.63964	85.01	965
85.02	496.63964	85.02	975
85.03	535.63964	85.03	986
85.04	481.63964	85.04	983
85.05	513.63964	85.05	965
85.06	475.63964	85.06	994
85.07	477.63964	85.07	961
85.08	515.63964	85.08	989
85.09	534.63964	85.09	985
85.1	470.63964	85.1	962
85.11	506.63964	85.11	997
85.12	468.63964	85.12	974
85.13	493.63964	85.13	952
85.14	502.63964	85.14	974
85.15	465.63964	85.15	964
85.16	449.63964	85.16	964
85.17	511.63964	85.17	987
85.18	505.63964	85.18	997
85.19	514.63964	85.19	971

85.2	511.63964	85.2	961
85.21	502.63964	85.21	946
85.22	487.63964	85.22	980
85.23	502.63964	85.23	997
85.24	462.63964	85.24	985
85.25	494.63964	85.25	972
85.26	502.63964	85.26	991
85.27	445.63964	85.27	971
85.28	525.63964	85.28	947
85.29	496.63964	85.29	988
85.3	479.63964	85.3	987
85.31	457.63964	85.31	966
85.32	489.63964	85.32	975
85.33	512.63964	85.33	985
85.34	489.63964	85.34	941
85.35	485.63964	85.35	987
85.36	482.63964	85.36	975
85.37	497.63964	85.37	967
85.38	483.63964	85.38	972
85.39	483.63964	85.39	988
85.4	490.63964	85.4	959
85.41	484.63964	85.41	989

85.42	509.63964	85.42	941
85.43	529.63964	85.43	945
85.44	514.63964	85.44	966
85.45	471.63964	85.45	973
85.46	504.63964	85.46	948
85.47	490.63964	85.47	990
85.48	485.63964	85.48	990
85.49	480.63964	85.49	949
85.5	485.63964	85.5	974
85.51	500.63964	85.51	968
85.52	461.63964	85.52	961
85.53	515.63964	85.53	974
85.54	465.63964	85.54	951
85.55	474.63964	85.55	994
85.56	515.63964	85.56	1003
85.57	502.63964	85.57	980
85.58	458.63964	85.58	960
85.59	530.63964	85.59	1019
85.6	493.63964	85.6	991
85.61	488.63964	85.61	959
85.62	542.63964	85.62	980
85.63	525.63964	85.63	965

85.64	494.63964	85.64	962
85.65	473.63964	85.65	964
85.66	460.63964	85.66	967
85.67	516.63964	85.67	953
85.68	505.63964	85.68	968
85.69	512.63964	85.69	967
85.7	515.63964	85.7	947
85.71	520.63964	85.71	970
85.72	514.63964	85.72	973
85.73	505.63964	85.73	956
85.74	486.63964	85.74	979
85.75	481.63964	85.75	992
85.76	474.63964	85.76	950
85.77	467.63964	85.77	959
85.78	488.63964	85.78	985
85.79	463.63964	85.79	966
85.8	513.63964	85.8	971
85.81	530.63964	85.81	970
85.82	470.63964	85.82	977
85.83	493.63964	85.83	978
85.84	518.63964	85.84	968
85.85	492.63964	85.85	986

85.86	520.63964	85.86	980
85.87	547.63964	85.87	973
85.88	493.63964	85.88	955
85.89	506.63964	85.89	949
85.9	476.63964	85.9	985
85.91	470.63964	85.91	959
85.92	506.63964	85.92	963
85.93	505.63964	85.93	952
85.94	499.63964	85.94	957
85.95	457.63964	85.95	954
85.96	523.63964	85.96	965
85.97	512.63964	85.97	981
85.98	509.63964	85.98	976
85.99	515.63964	85.99	982
86	518.63964	86	966
86.01	549.63964	86.01	947
86.02	511.63964	86.02	966
86.03	478.63964	86.03	962
86.04	507.63964	86.04	974
86.05	452.63964	86.05	983
86.06	484.63964	86.06	966
86.07	467.63964	86.07	971

86.08	497.63964	86.08	965
86.09	504.63964	86.09	978
86.1	493.63964	86.1	944
86.11	482.63964	86.11	942
86.12	479.63964	86.12	980
86.13	537.63964	86.13	986
86.14	500.63964	86.14	1000
86.15	488.63964	86.15	962
86.16	480.63964	86.16	952
86.17	440.63964	86.17	970
86.18	466.63964	86.18	983
86.19	506.63964	86.19	975
86.2	488.63964	86.2	979
86.21	467.63964	86.21	948
86.22	488.63964	86.22	967
86.23	499.63964	86.23	931
86.24	481.63964	86.24	957
86.25	493.63964	86.25	970
86.26	490.63964	86.26	942
86.27	507.63964	86.27	977
86.28	448.63964	86.28	949
86.29	509.63964	86.29	952

86.3	503.63964	86.3	959
86.31	470.63964	86.31	957
86.32	465.63964	86.32	978
86.33	502.63964	86.33	985
86.34	447.63964	86.34	981
86.35	513.63964	86.35	972
86.36	490.63964	86.36	974
86.37	477.63964	86.37	977
86.38	465.63964	86.38	983
86.39	518.63964	86.39	938
86.4	495.63964	86.4	995
86.41	512.63964	86.41	970
86.42	453.63964	86.42	979
86.43	491.63964	86.43	970
86.44	488.63964	86.44	977
86.45	456.63964	86.45	989
86.46	497.63964	86.46	976
86.47	486.63964	86.47	961
86.48	525.63964	86.48	975
86.49	503.63964	86.49	952
86.5	537.63964	86.5	963
86.51	508.63964	86.51	960

86.52	478.63964	86.52	955
86.53	447.63964	86.53	965
86.54	499.63964	86.54	967
86.55	466.63964	86.55	956
86.56	471.63964	86.56	946
86.57	456.63964	86.57	946
86.58	533.63964	86.58	959
86.59	501.63964	86.59	951
86.6	477.63964	86.6	973
86.61	513.63964	86.61	976
86.62	492.63964	86.62	953
86.63	496.63964	86.63	946
86.64	469.63964	86.64	972
86.65	486.63964	86.65	956
86.66	488.63964	86.66	941
86.67	514.63964	86.67	949
86.68	491.63964	86.68	959
86.69	465.63964	86.69	945
86.7	521.63964	86.7	949
86.71	465.63964	86.71	978
86.72	518.63964	86.72	955
86.73	504.63964	86.73	975

86.74	482.63964	86.74	963
86.75	476.63964	86.75	959
86.76	510.63964	86.76	957
86.77	480.63964	86.77	959
86.78	491.63964	86.78	948
86.79	466.63964	86.79	972
86.8	455.63964	86.8	975
86.81	470.63964	86.81	966
86.82	499.63964	86.82	976
86.83	491.63964	86.83	971
86.84	505.63964	86.84	933
86.85	497.63964	86.85	987
86.86	483.63964	86.86	988
86.87	503.63964	86.87	963
86.88	509.63964	86.88	991
86.89	533.63964	86.89	974
86.9	490.63964	86.9	968
86.91	467.63964	86.91	979
86.92	441.63964	86.92	979
86.93	456.63964	86.93	975
86.94	470.63964	86.94	982
86.95	471.63964	86.95	967

86.96	485.63964	86.96	946
86.97	478.63964	86.97	968
86.98	504.63964	86.98	976
86.99	473.63964	86.99	960
87	532.63964	87	962
87.01	508.63964	87.01	929
87.02	469.63964	87.02	946
87.03	478.63964	87.03	977
87.04	506.63964	87.04	943
87.05	485.63964	87.05	989
87.06	445.63964	87.06	949
87.07	456.63964	87.07	927
87.08	443.63964	87.08	955
87.09	487.63964	87.09	962
87.1	494.63964	87.1	952
87.11	539.63964	87.11	944
87.12	514.63964	87.12	957
87.13	463.63964	87.13	956
87.14	481.63964	87.14	971
87.15	528.63964	87.15	961
87.16	500.63964	87.16	974
87.17	506.63964	87.17	963

87.18	502.63964	87.18	974
87.19	497.63964	87.19	969
87.2	470.63964	87.2	976
87.21	463.63964	87.21	978
87.22	469.63964	87.22	992
87.23	468.63964	87.23	991
87.24	474.63964	87.24	963
87.25	500.63964	87.25	980
87.26	536.63964	87.26	971
87.27	464.63964	87.27	983
87.28	477.63964	87.28	985
87.29	497.63964	87.29	960
87.3	543.63964	87.3	943
87.31	464.63964	87.31	978
87.32	481.63964	87.32	949
87.33	464.63964	87.33	996
87.34	504.63964	87.34	986
87.35	486.63964	87.35	990
87.36	499.63964	87.36	947
87.37	470.63964	87.37	953
87.38	533.63964	87.38	955
87.39	501.63964	87.39	963

87.4	517.63964	87.4	989
87.41	521.63964	87.41	952
87.42	480.63964	87.42	979
87.43	473.63964	87.43	974
87.44	519.63964	87.44	939
87.45	520.63964	87.45	965
87.46	489.63964	87.46	951
87.47	474.63964	87.47	983
87.48	522.63964	87.48	970
87.49	451.63964	87.49	933
87.5	516.63964	87.5	975
87.51	527.63964	87.51	972
87.52	491.63964	87.52	994
87.53	476.63964	87.53	975
87.54	515.63964	87.54	965
87.55	449.63964	87.55	968
87.56	505.63964	87.56	951
87.57	463.63964	87.57	951
87.58	477.63964	87.58	948
87.59	518.63964	87.59	956
87.6	466.63964	87.6	960
87.61	476.63964	87.61	971

87.62	469.63964	87.62	965
87.63	477.63964	87.63	980
87.64	520.63964	87.64	986
87.65	511.63964	87.65	981
87.66	515.63964	87.66	954
87.67	473.63964	87.67	975
87.68	482.63964	87.68	960
87.69	480.63964	87.69	985
87.7	476.63964	87.7	979
87.71	509.63964	87.71	982
87.72	517.63964	87.72	962
87.73	463.63964	87.73	972
87.74	526.63964	87.74	974
87.75	523.63964	87.75	940
87.76	497.63964	87.76	944
87.77	482.63964	87.77	976
87.78	484.63964	87.78	1003
87.79	519.63964	87.79	990
87.8	508.63964	87.8	952
87.81	500.63964	87.81	975
87.82	482.63964	87.82	968
87.83	495.63964	87.83	966

87.84	456.63964	87.84	997
87.85	467.63964	87.85	982
87.86	509.63964	87.86	980
87.87	515.63964	87.87	982
87.88	543.63964	87.88	985
87.89	479.63964	87.89	974
87.9	496.63964	87.9	965
87.91	510.63964	87.91	955
87.92	490.63964	87.92	961
87.93	470.63964	87.93	956
87.94	519.63964	87.94	987
87.95	507.63964	87.95	993
87.96	517.63964	87.96	979
87.97	515.63964	87.97	939
87.98	496.63964	87.98	933
87.99	506.63964	87.99	957
88	493.63964	88	976
88.01	495.63964	88.01	961
88.02	501.63964	88.02	953
88.03	519.63964	88.03	960
88.04	502.63964	88.04	987
88.05	489.63964	88.05	954

88.06	490.63964	88.06	974
88.07	486.63964	88.07	963
88.08	509.63964	88.08	964
88.09	529.63964	88.09	979
88.1	514.63964	88.1	933
88.11	515.63964	88.11	977
88.12	461.63964	88.12	959
88.13	510.63964	88.13	950
88.14	517.63964	88.14	942
88.15	543.63964	88.15	957
88.16	440.63964	88.16	934
88.17	460.63964	88.17	964
88.18	502.63964	88.18	973
88.19	511.63964	88.19	968
88.2	499.63964	88.2	980
88.21	452.63964	88.21	974
88.22	504.63964	88.22	974
88.23	501.63964	88.23	933
88.24	540.63964	88.24	979
88.25	506.63964	88.25	964
88.26	515.63964	88.26	951
88.27	483.63964	88.27	965

88.28	535.63964	88.28	949
88.29	474.63964	88.29	926
88.3	487.63964	88.3	974
88.31	492.63964	88.31	967
88.32	491.63964	88.32	971
88.33	454.63964	88.33	949
88.34	488.63964	88.34	971
88.35	500.63964	88.35	943
88.36	490.63964	88.36	967
88.37	497.63964	88.37	975
88.38	507.63964	88.38	979
88.39	453.63964	88.39	978
88.4	506.63964	88.4	1000
88.41	518.63964	88.41	998
88.42	474.63964	88.42	950
88.43	513.63964	88.43	936
88.44	501.63964	88.44	952
88.45	522.63964	88.45	966
88.46	495.63964	88.46	979
88.47	506.63964	88.47	968
88.48	499.63964	88.48	971
88.49	463.63964	88.49	988

88.5	527.63964	88.5	970
88.51	544.63964	88.51	950
88.52	536.63964	88.52	981
88.53	475.63964	88.53	974
88.54	489.63964	88.54	946
88.55	496.63964	88.55	954
88.56	476.63964	88.56	947
88.57	479.63964	88.57	969
88.58	497.63964	88.58	991
88.59	526.63964	88.59	966
88.6	494.63964	88.6	956
88.61	493.63964	88.61	963
88.62	482.63964	88.62	992
88.63	528.63964	88.63	996
88.64	537.63964	88.64	987
88.65	480.63964	88.65	974
88.66	504.63964	88.66	957
88.67	489.63964	88.67	965
88.68	503.63964	88.68	992
88.69	496.63964	88.69	946
88.7	510.63964	88.7	1013
88.71	473.63964	88.71	980

88.72	479.63964	88.72	940
88.73	518.63964	88.73	949
88.74	525.63964	88.74	994
88.75	513.63964	88.75	990
88.76	521.63964	88.76	952
88.77	503.63964	88.77	983
88.78	501.63964	88.78	960
88.79	479.63964	88.79	990
88.8	515.63964	88.8	994
88.81	504.63964	88.81	971
88.82	516.63964	88.82	973
88.83	506.63964	88.83	982
88.84	551.63964	88.84	965
88.85	494.63964	88.85	963
88.86	520.63964	88.86	951
88.87	499.63964	88.87	985
88.88	501.63964	88.88	965
88.89	489.63964	88.89	977
88.9	528.63964	88.9	976
88.91	500.63964	88.91	971
88.92	515.63964	88.92	955
88.93	527.63964	88.93	955

88.94	513.63964	88.94	982
88.95	511.63964	88.95	971
88.96	513.63964	88.96	982
88.97	531.63964	88.97	977
88.98	493.63964	88.98	968
88.99	529.63964	88.99	985
89	525.63964	89	986
89.01	548.63964	89.01	977
89.02	522.63964	89.02	1004
89.03	523.63964	89.03	979
89.04	538.63964	89.04	979
89.05	533.63964	89.05	979
89.06	526.63964	89.06	982
89.07	505.63964	89.07	969
89.08	543.63964	89.08	954
89.09	527.63964	89.09	954
89.1	559.63964	89.1	954
89.11	522.63964	89.11	963
89.12	522.63964	89.12	1003
89.13	540.63964	89.13	962
89.14	556.63964	89.14	953
89.15	541.63964	89.15	987

89.16	507.63964	89.16	975
89.17	517.63964	89.17	961
89.18	541.63964	89.18	978
89.19	532.63964	89.19	962
89.2	519.63964	89.2	956
89.21	500.63964	89.21	961
89.22	531.63964	89.22	983
89.23	527.63964	89.23	977
89.24	499.63964	89.24	986
89.25	511.63964	89.25	968
89.26	539.63964	89.26	943
89.27	540.63964	89.27	1009
89.28	491.63964	89.28	961
89.29	505.63964	89.29	975
89.3	535.63964	89.3	979
89.31	486.63964	89.31	985
89.32	527.63964	89.32	996
89.33	533.63964	89.33	977
89.34	516.63964	89.34	960
89.35	537.63964	89.35	969
89.36	504.63964	89.36	990
89.37	519.63964	89.37	965

89.38	527.63964	89.38	978
89.39	482.63964	89.39	975
89.4	516.63964	89.4	998
89.41	523.63964	89.41	996
89.42	531.63964	89.42	954
89.43	557.63964	89.43	980
89.44	530.63964	89.44	985
89.45	557.63964	89.45	985
89.46	549.63964	89.46	980
89.47	563.63964	89.47	985
89.48	517.63964	89.48	968
89.49	493.63964	89.49	959
89.5	499.63964	89.5	975
89.51	529.63964	89.51	950
89.52	511.63964	89.52	966
89.53	489.63964	89.53	997
89.54	518.63964	89.54	1007
89.55	507.63964	89.55	977
89.56	487.63964	89.56	966
89.57	555.63964	89.57	1001
89.58	539.63964	89.58	980
89.59	554.63964	89.59	970

89.6	553.63964	89.6	999
89.61	548.63964	89.61	986
89.62	517.63964	89.62	986
89.63	514.63964	89.63	997
89.64	503.63964	89.64	971
89.65	564.63964	89.65	998
89.66	528.63964	89.66	978
89.67	493.63964	89.67	967
89.68	495.63964	89.68	955
89.69	547.63964	89.69	993
89.7	537.63964	89.7	968
89.71	469.63964	89.71	979
89.72	531.63964	89.72	966
89.73	557.63964	89.73	980
89.74	503.63964	89.74	960
89.75	538.63964	89.75	974
89.76	539.63964	89.76	957
89.77	514.63964	89.77	964
89.78	520.63964	89.78	965
89.79	527.63964	89.79	980
89.8	570.63964	89.8	985
89.81	543.63964	89.81	978

89.82	481.63964	89.82	974
89.83	495.63964	89.83	955
89.84	538.63964	89.84	963
89.85	513.63964	89.85	935
89.86	518.63964	89.86	980
89.87	526.63964	89.87	957
89.88	539.63964	89.88	964
89.89	522.63964	89.89	981
89.9	518.63964	89.9	948
89.91	537.63964	89.91	974
89.92	552.63964	89.92	948
89.93	562.63964	89.93	960
89.94	504.63964	89.94	964
89.95	508.63964	89.95	970
89.96	503.63964	89.96	950
89.97	556.63964	89.97	965
89.98	522.63964	89.98	956
89.99	502.63964	89.99	962
90	529.63964	90	939

UV-vis-NIR absorption spectra of Cu_9S_8 nanoparticles:

1300	0.05777	0.05777	0.37858
1295	0.05864	0.05865	0.38702

1290	0.05953	0.05954	0.39448
1285	0.06044	0.06045	0.40088
1280	0.06132	0.0613	0.40597
1275	0.06199	0.062	0.40979
1270	0.06252	0.06253	0.41243
1265	0.06282	0.06276	0.41382
1260	0.06267	0.06274	0.41415
1255	0.06261	0.06258	0.41349
1250	0.06241	0.06243	0.41217
1245	0.06222	0.06221	0.41019
1240	0.06195	0.06194	0.40795
1235	0.06163	0.06164	0.40557
1230	0.06135	0.06134	0.40339
1225	0.06112	0.06114	0.40161
1220	0.06103	0.06099	0.40055
1215	0.06093	0.061	0.40042
1210	0.06114	0.06109	0.40141
1205	0.06125	0.06124	0.40385
1200	0.06152	0.06158	0.40788
1195	0.06229	0.06222	0.41428
1190	0.06317	0.06321	0.42299
1185	0.06459	0.06459	0.43395

1180	0.06647	0.06645	0.44715
1175	0.06871	0.06872	0.46246
1170	0.07148	0.07152	0.47956
1165	0.0748	0.07477	0.4981
1160	0.07818	0.07813	0.5179
1155	0.08143	0.08149	0.53869
1150	0.08491	0.08483	0.56008
1145	0.08821	0.08829	0.58159
1140	0.09183	0.09179	0.60284
1135	0.0953	0.09528	0.62357
1130	0.09857	0.09863	0.64337
1125	0.10174	0.10168	0.66191
1120	0.10424	0.10423	0.67894
1115	0.10628	0.10634	0.69445
1110	0.10818	0.10814	0.70805
1105	0.10957	0.10955	0.71986
1100	0.11052	0.11055	0.72963
1095	0.11137	0.11139	0.73768
1090	0.11216	0.11211	0.74395
1085	0.11273	0.11278	0.7487
1080	0.11342	0.11339	0.75214
1075	0.11394	0.11393	0.75451

1070	0.11429	0.11428	0.75616
1065	0.11449	0.11446	0.75728
1060	0.11461	0.11467	0.75781
1055	0.11502	0.115	0.75827
1050	0.11532	0.11526	0.7588
1045	0.1153	0.11535	0.75933
1040	0.11543	0.11543	0.75979
1035	0.11561	0.11559	0.76118
1030	0.11571	0.11572	0.76309
1025	0.11587	0.11583	0.76553
1020	0.11605	0.11614	0.76857
1015	0.11669	0.11656	0.7718
1010	0.11718	0.11737	0.77669
1005	0.11844	0.11823	0.78223
1000	0.11911	0.11927	0.78929
995	0.12029	0.12019	0.79662
990	0.12124	0.12134	0.80362
985	0.12247	0.12235	0.81061
980	0.12334	0.12344	0.81787
975	0.12462	0.12456	0.82533
970	0.12593	0.12606	0.83239
965	0.1276	0.12733	0.83932

960	0.12846	0.12876	0.84619
955	0.13045	0.13037	0.85279
950	0.13206	0.13204	0.85952
945	0.13267	0.13231	0.86473
940	0.13188	0.13251	0.86935
935	0.13345	0.1331	0.87245
930	0.13379	0.13371	0.87562
925	0.13336	0.13377	0.87892
920	0.13346	0.13245	0.88229
915	0.13054	0.13193	0.88572
910	0.13286	0.13157	0.88922
905	0.1325	0.13376	0.89265
900	0.13661	0.13585	0.89588
895	0.13762	0.13755	0.89892
890	0.1368	0.13689	0.90156
885	0.13617	0.13633	0.90387
880	0.13671	0.13679	0.90565
875	0.13757	0.13722	0.9071
870	0.13688	0.13706	0.9079
865	0.1368	0.13691	0.90803
860	0.13741	0.13697	0.90724
855	0.13747	0.13858	0.90552

850	0.14079	0.13931	0.90242
845	0.13821	0.13951	0.89773
840	0.13789	0.13659	0.89153
835	0.13286	0.13383	0.88433
830	0.1313	0.1311	0.87549
825	0.13011	0.13002	0.86533
820	0.12852	0.12849	0.85404
815	0.12675	0.12685	0.84183
810	0.1253	0.1252	0.83008
805	0.12346	0.12351	0.81754
800	0.12172	0.12175	0.80533
795	0.11999	0.11991	0.79187
790	0.11781	0.11786	0.77768
785	0.11568	0.11565	0.76362
780	0.11356	0.11363	0.74983
775	0.1117	0.11161	0.73616
770	0.10954	0.10959	0.72237
765	0.10758	0.10758	0.7091
760	0.10571	0.1057	0.69637
755	0.1038	0.10378	0.68383
750	0.10179	0.10181	0.67162
745	0.09984	0.09985	0.65822

740	0.0979	0.09787	0.64502
735	0.09585	0.09588	0.63122
730	0.09392	0.09392	0.61862
725	0.09204	0.09203	0.60654
720	0.09016	0.09019	0.59486
715	0.08841	0.08838	0.58377
710	0.08666	0.08668	0.57341
705	0.08512	0.08512	0.56357
700	0.08379	0.0838	0.55447
695	0.08258	0.08253	0.54608
690	0.08128	0.08133	0.53836
685	0.08028	0.08026	0.53137
680	0.07942	0.07943	0.52516
675	0.0787	0.07869	0.51975
670	0.07801	0.07799	0.51506
665	0.07735	0.07739	0.5111
660	0.07697	0.07694	0.5078
655	0.07665	0.07666	0.50516
650	0.07645	0.07644	0.50305
645	0.07628	0.07629	0.50153
640	0.07612	0.07609	0.50041
635	0.07588	0.0759	0.49982

630	0.0758	0.07581	0.49962
625	0.07585	0.07584	0.49988
620	0.07592	0.07592	0.50068
615	0.07606	0.07606	0.50193
610	0.07631	0.07632	0.50378
605	0.0767	0.07669	0.50629
600	0.07717	0.07716	0.50945
595	0.07774	0.07776	0.51335
590	0.07847	0.07844	0.5179
585	0.07924	0.07927	0.52338
580	0.08024	0.08022	0.52972
575	0.08132	0.08132	0.53704
570	0.08256	0.08257	0.54536
565	0.08403	0.08402	0.5548
560	0.08565	0.08567	0.56536
555	0.08745	0.08741	0.5771
550	0.08931	0.08936	0.58997
545	0.09155	0.09151	0.60403
540	0.09384	0.09385	0.61915
535	0.09627	0.09627	0.63532
530	0.09885	0.09886	0.65254
525	0.10163	0.1016	0.67102

520	0.10449	0.10452	0.69043
515	0.10759	0.10756	0.71089
510	0.11078	0.11081	0.73234
505	0.11425	0.11421	0.75484
500	0.11776	0.11782	0.77768
495	0.12153	0.12147	0.80183
490	0.12512	0.12517	0.82625
485	0.12883	0.12877	0.8512
480	0.1324	0.13248	0.87589
475	0.13632	0.13628	0.90077
470	0.14016	0.14014	0.92545
465	0.14391	0.14394	0.94967
460	0.14778	0.14777	0.97357
455	0.15161	0.15158	0.99719
450	0.15533	0.1554	1.01957
445	0.15919	0.15913	1.043
440	0.16266	0.16267	1.06689
435	0.16592	0.16588	1.09144
430	0.16898	0.16909	1.1152
425	0.17231	0.1722	1.13883
420	0.17515	0.17518	1.1618
415	0.17804	0.17802	1.18457

410	0.18114	0.18143	1.208
405	0.18483	0.184	1.23011
400	0.1861	0.18727	1.2509
395	0.19165	0.19067	1.27169
390	0.19449	0.19521	1.29129
385	0.19896	0.19822	1.30951
380	0.20098	0.20166	1.32627
375	0.20507	0.20476	1.34152
370	0.20782	0.20775	1.35524
365	0.20946	0.20956	1.36745
360	0.21107	0.21154	1.37834
355	0.21258	0.21105	1.38811
350	0.20845	0.21011	1.39709
345	0.21094	0.21009	1.40567
340	0.21293	0.21376	1.41425
335	0.21726	0.21605	1.42362
330	0.21705	0.21805	1.43444
325	0.21951	0.21877	1.44764
320	0.22018	0.22029	1.46421
315	0.22319	0.22423	1.4854
310	0.23139	0.23019	1.51246
305	0.23554	0.23544	1.54697

300 0.24038 0.24063 1.5906

(2) Rheological and mechanical properties of hydrogels:

0 137.807 78.211

6.11673 148.479 80.1119

12.14403 156.814 81.249

18.23793 162.977 82.9322

24.34703 168.68 84.1515

30.46164 5.89745 31.9143

36.41123 5.59222 31.812

42.48353 5.41663 31.7789

48.57133 5.311 31.7848

54.78353 5.24377 31.8179

60.79336 194.429 92.7927

66.83393 200.881 93.692

72.91263 206.746 94.6971

78.99883 211.482 95.4823

85.15083 215.516 96.2428

91.05465 5.06999 32.1513

97.09533 5.03165 32.0625

103.21633 5.02827 32.0438

109.27493 5.03919 32.0615

115.41543 5.05818 32.1004

121.29067	202.897	95.6039
127.42173	210.256	96.2593
133.57953	215.21	96.9646
139.48893	219.685	97.9168
145.72193	223.57	98.2134
151.88732	5.09259	32.4308
157.81653	5.09417	32.3456
163.94963	5.11567	32.3313
169.94143	5.14875	32.348
176.04503	5.18955	32.389

thixotropic recovery behavior, strain-dependent storage modulus (G′) and loss modulus (G″):

0.01064	99.1825	68.7548
0.01708	76.4891	43.9062
0.02337	117.957	55.1104
0.03938	121.551	57.4981
0.06344	113.667	63.285
0.0993	118.355	62.701
0.15806	118.758	62.6959
0.25121	119.418	61.6301
0.39853	117.583	62.8009
0.63029	118.988	61.7316

0.99786	120.095	62.25	
1.58165	121.237	62.6608	
2.50832	121.96	62.981	
3.97749	122.38	63.3881	
6.30778	122.52	63.351	
10.0035	122.198	63.337	
15.8709	121.279	63.0977	
25.2099	118.949	62.6329	
40.1338	113.762	61.7081	
64.181	102.845	60.1951	
103.282	81.8454	58.7924	
167.337	48.1837	55.8811	
265.38	21.0256	43.8987	
411.454	9.12858	30.8388	
643.243	3.95419	20.6013	
1011.21	1.67086	13.3956	
0.01004		279.682	120.094
0.01669		220.797	116.042
0.02451		243.501	107.511
0.03966		245.362	109.724
0.0635		240.44	111.124
0.09831		252.055	115.966

0.16032	243.988	112.209	
0.24924	248.175	116.804	
0.39788	249.014	116.322	
0.63085	249.515	115.609	
0.99852	250.835	115.209	
1.58367	251.077	115.681	
2.50728	252.638	115.669	
3.97318	253.778	116.51	
6.30524	254.171	116.609	
10.0077	253.473	116.558	
15.9019	250.729	116.256	
25.3364	243.747	115.525	
40.6475	227.09	114.16	
65.9855	190.739	113.395	
109.99	118.707	110.873	
176.053	55.9331	91.009	
268.753	26.5186	67.093	
415.98	12.1397	46.6969	
648.12	5.37486	31.5709	
1016.29	2.2925	20.7194	
0.0104		400.744	201.966
0.01619		391.182	156.915

0.0246	406.521	169.068
0.03949	415.969	167.203
0.06317	413.277	170.561
0.09936	420.136	167.652
0.15773	425.162	168.878
0.25096	426.158	168.382
0.39608	431.903	169.07
0.62891	435.345	170.063
0.99615	439.326	171.22
1.57845	443.656	172.18
2.50535	446.456	172.448
3.97361	448.447	172.686
6.30197	449.693	173.003
10.0073	448.655	173.178
15.9378	441.95	172.682
25.5315	423.196	171.156
41.3852	378.849	171.557
69.707	266.818	186.421
119.157	125.966	166.729
177.576	63.6943	128.861
273.228	30.7058	90.9934
420.293	14.9271	62.1754

652.444 7.25263 41.9112

1020.97 3.54434 27.7287

frequency-dependent G′ and G″：

0.1 12.7654 17.9148

0.15849 21.5904 21.1148

0.25119 30.8746 24.3528

0.39811 38.1987 25.7721

0.63096 45.3011 30.1599

1 54.5325 33.8043

1.5849 65.4214 39.1487

2.51189 77.2783 43.5392

3.98105 89.1138 50.77

6.30957 104.646 57.2051

10.0001 122.551 64.716

15.849 142.489 73.756

25.1188 165.493 85.1523

39.8105 195.561 96.5569

63.0957 236.018 107.745

100 322.176 113.509

0.1 64.3421 41.5241

0.15849 84.8182 48.3232

0.25119 102.967 56.43

0.39811	122.939	62.9762
0.63096	142.075	69.6681
1	162.888	78.0073
1.5849	187.569	87.8873
2.51189	213.751	96.4465
3.98105	243.502	109.859
6.30957	276.987	122.523
10.0001	312.6	137.647
15.849	352.921	154.312
25.1188	396.905	172.995
39.8105	445.368	194.285
63.0957	493.85	215.955
100	561.983	233.167
0.1	113.208	69.9509
0.15849	141.734	77.256
0.25119	171.775	84.77
0.39811	201.628	90.2071
0.63096	227.615	98.6248
1	258.594	107.676
1.5849	291.742	118.293
2.51189	326.536	129.992
3.98105	364.804	143.505

6.30957	407.486	158.179
10.0001	454.327	175.631
15.849	505.78	194.898
25.1188	563.325	216.605
39.8105	627.734	240.76
63.0957	696.599	266.205
100	793.884	285.61

stress–strain curves:

0.01064	0.01284
0.01708	0.01506
0.02337	0.03043
0.03938	0.05295
0.06344	0.08253
0.0993	0.13299
0.15806	0.21226
0.25121	0.33758
0.39853	0.53126
0.63029	0.84489
0.99786	1.3498
1.58165	2.15851
2.50832	3.44297
3.97749	5.48187

6.30778	8.70026
10.0035	13.7685
15.8709	21.6972
25.2099	33.89
40.1338	51.9415
64.181	76.4819
103.282	104.08
167.337	123.471
265.38	129.171
411.454	132.33
643.243	134.935
1011.21	136.507
0.01004	0.03055
0.01669	0.04162
0.02451	0.06523
0.03966	0.10661
0.0635	0.1682
0.09831	0.27276
0.16032	0.43055
0.24924	0.68362
0.39788	1.09354
0.63085	1.7348

0.99852	2.75619
1.58367	4.37796
2.50728	6.96668
3.97318	11.0949
6.30524	17.6322
10.0077	27.9204
15.9019	43.9482
25.3364	68.3419
40.6475	103.314
65.9855	146.422
109.99	178.659
176.053	188.065
268.753	193.888
415.98	200.706
648.12	207.561
1016.29	211.853
0.0104	0.04668
0.01619	0.06825
0.0246	0.10831
0.03949	0.17703
0.06317	0.28242
0.09936	0.44947

0.15773	0.72155
0.25096	1.14996
0.39608	1.83709
0.62891	2.9394
0.99615	4.69695
1.57845	7.51179
2.50535	11.9907
3.97361	19.0951
6.30197	30.3643
10.0073	48.1268
15.9378	75.6231
25.5315	116.551
41.3852	172.114
69.707	226.89
119.157	248.995
177.576	255.253
273.228	262.393
420.293	268.744
652.444	277.511
1020.97	285.404

time-dependent swelling ratio:

0	0

3 74.90446

10 86.30573

30 98.34395

70 116.17834

110 126.6242

150 129.10828

240 129.68153

0 0

3 49.41776

10 76.34643

30 98.39884

70 122.92576

110 145.56041

150 160.18923

240 161.57205

temperature-dependent G′ and G″:

25.6 55.6816 33.449

26.27 55.2531 34.1553

26.91 55.5186 33.498

27.48 55.5439 33.9115

28 55.6365 33.4337

28.52 55.2695 33.4544

29.03	55.3816	34.2527
29.51	55.2987	33.3536
30.03	55.0503	33.6114
30.54	55.3711	33.3719
31.04	55.1113	33.2392
31.56	54.7044	33.4447
32.05	55.143	33.4662
32.54	54.8622	33.6645
33.08	54.9308	34.1756
33.56	54.558	33.4468
34.05	54.7783	33.9939
34.56	54.7801	34.2572
35.07	53.9073	33.8804
35.62	53.9507	33.8493
36.12	54.1946	34.0961
36.61	53.865	33.588
37.09	54.1465	34.1214
37.63	53.8059	34.1811
38.13	54.3996	34.5403
38.61	54.2686	34.3593
39.14	54.0946	34.1237
39.63	54.2574	34.7042

40.13	54.8442	34.4635
40.66	54.8031	35.0853
41.18	54.8924	35.2627
41.68	55.0156	35.3162
42.15	55.4567	35.251
42.68	55.7624	34.813
43.19	55.8932	35.149
43.68	56.0094	35.6567
44.18	56.1681	35.7287
44.7	56.6002	35.7258
45.21	57.319	36.3119
45.68	56.9459	36.057
46.21	56.9239	36.1667
46.72	57.5663	36.8382
47.21	58.3854	37.2535
47.75	58.9033	37.1737
48.24	59.2601	37.4214
48.76	59.6336	36.1878
49.25	60.5449	37.3874
49.78	60.6721	38.0212
50.26	61.2192	38.0503
50.75	61.5263	38.6341

51.27	61.6612	38.6074	
51.79	63.7177	38.6837	
52.29	63.4972	38.8132	
52.8	63.8166	39.3195	
53.29	64.4238	39.4041	
53.83	65.0479	39.6708	
54.31	65.624	39.6157	
54.82	66.1866	40.004	
55.25	66.6422	40.6551	
55.35	66.9681	40.3207	
25.61		228.393	98.5091
26.3		228.046	97.776
26.89		226.747	97.9428
27.47		225.296	97.2044
28.01		223.775	97.2598
28.52		221.797	96.6783
29.05		220.387	96.5488
29.52		218.61	95.7829
30.02		216.377	96.0163
30.55		214.484	95.3593
31.02		212.627	95.2224
31.55		209.903	94.4539

32.06	208.516	93.7897
32.54	205.83	93.4977
33.05	203.992	93.286
33.55	201.747	93.2457
34.05	200.049	92.1231
34.59	198.178	91.8362
35.09	195.878	92.0103
35.57	194.399	91.3112
36.09	192.048	90.6066
36.62	190.722	89.9127
37.09	188.919	90.1017
37.6	187.233	89.474
38.11	185.49	89.6187
38.62	183.756	89.2441
39.14	182.417	88.5276
39.64	180.983	88.2903
40.14	179.593	87.7636
40.65	178.002	87.2152
41.16	176.19	86.8968
41.68	175.256	86.8476
42.16	173.939	86.0993
42.69	172.621	85.7154

43.19	170.829	85.1206
43.67	168.89	84.8574
44.17	168.547	84.7705
44.72	166.704	84.3102
45.21	165.485	83.5469
45.7	164.12	82.8017
46.2	162.676	82.2271
46.71	160.857	82.0485
47.22	159.173	81.1538
47.72	157.094	80.3074
48.25	155.846	80.2319
48.74	153.844	79.0179
49.22	152.456	78.6292
49.75	150.452	78.3671
50.28	147.933	77.1121
50.76	146.104	76.3214
51.24	144.068	76.0341
51.75	141.99	74.9859
52.28	139.339	73.6069
52.77	136.412	72.9855
53.3	134.101	72.109
53.81	131.384	70.7219

54.31	128.516	69.978	
54.8	125.288	68.4586	
55.25	122.449	67.2545	
55.37	120.479	66.9144	
25.61		283.697	110.198
26.27		283.033	110.27
26.89		282.262	109.722
27.48		281.13	109.111
28.02		280.246	109.385
28.54		278.722	108.479
29.03		277.396	108.482
29.52		275.385	107.688
30.03		273.547	107.37
30.54		271.75	106.466
31.02		269.514	106.061
31.55		268.271	105.722
32.05		265.343	105.237
32.56		264.299	104.806
33.06		262.087	104.372
33.59		259.924	103.444
34.06		257.823	103.049
34.57		256.155	102.414

35.08	253.971	102.041
35.6	252.222	101.09
36.12	250.331	101.31
36.62	248.502	100.321
37.11	247.094	100.051
37.61	244.736	99.3408
38.12	243.582	99.0518
38.62	241.824	98.0829
39.14	240.124	97.7132
39.66	238.668	97.3422
40.14	237.082	97.0428
40.65	235.626	96.8584
41.17	234.395	96.594
41.66	232.685	96.3557
42.14	231.532	95.9123
42.68	230.463	95.182
43.2	228.956	95.0381
43.69	227.75	94.2731
44.18	226.423	93.7973
44.71	225.027	93.4937
45.22	223.826	92.9813
45.7	222.421	92.382

46.21	220.718	91.9475
46.73	219.052	91.9695
47.22	218.098	91.0587
47.74	217.188	90.8125
48.24	215.582	90.3408
48.76	214.301	90.1175
49.24	212.361	89.6721
49.77	210.908	88.7094
50.28	209.414	88.8858
50.78	207.137	88.5765
51.26	205.938	87.947
51.77	203.826	87.2455
52.28	201.445	86.3971
52.77	199.484	85.9329
53.29	197.402	85.525
53.82	194.722	84.2726
54.33	191.422	83.5694
54.81	189.523	82.7121
55.25	186.847	82.1555
55.36	184.866	81.9283

(3) Photothermal performance data:

photothermal conversion efficiency data of Cu_9S_8 nanoparticles:

0	27.7	0	0
40	39	0.10616	40
80	44.6	0.27418	80
120	51.4	0.41996	120
160	55.7	0.56503	160
200	57.5	0.70037	200
240	59	0.83459	240
280	61.5	0.95168	280
320	63.4	1.07024	320
360	65.2	1.1968	360
400	66.4	1.36026	400
440	67.5	1.46874	440
480	68.7	1.56718	480
520	64.5	1.67638	520
560	58.7	1.81358	560
600	54.4	1.90595	600
640	50.7	2.0441	640
680	47.7	2.18294	680
720	45.1	2.31951	720
760	43.1	2.47774	760
800	41.3	2.56735	800
840	39.6	2.63189	840

880	37.7	2.81421	880
920	36.6	2.94204	920
960	35.7	3.03735	960
1000	34.8	3.19987	1000
1040	33.8	3.39403	1040
1080	33.2	3.63519	1080
1120	32.4	3.63519	1120
1160	31.7	3.63519	1160
1200	31.1	3.7305	1200
1240	30.5	3.95364	1240
1280	30.2		
1320	30		
1360	29.5		
1400	29.2		
1440	29		
1480	28.7		
1520	28.4		
1560	28.1		
1600	28.1		
1640	28.1		
1680	28		
1720	27.8		

phothothermal heating test data of CSGGT and GGT hydrogels:

0 18.4 18.4

60 23 35

120 25 43

180 26.5 49

240 27.7 53

300 28.7 56.7

360 29.5 58.1

420 30.1 58.6

480 30.5 59

540 30.8 59.3

600 30.9 59.45

(4) Antibacterial assay data: quantitative statistical results of bacterial killing efficacy against *Escherichia coli* (*E. coli*) and *Staphylococcus aureus* (*S. aureus*) for different experimental groups.

GGT 0.05799 0.04576 0.00764 0.03713 0.02626

GGT + NIR 0.13717 0.18046 0.08551 0.13438 0.04754

GGT + CS 1.22488 1.01773 1.29003 1.17755 0.14219

GGT + CS + NIR 2.30103 2 2.33579 2.21227 0.18465

GGT 0.1478 0.10084 0.30103 0.18322 0.10469

GGT + NIR	0.21632	0.25181	0.36218	0.27677	0.07607
GGT + CS	1.18966	1.02872	1.17609	1.13149	0.08926
GGT + CS + NIR	1.98361	2.16749	3.47712	2.54274	0.8144

PBS + NIR	0.15393	0.05461	0.47712	0.22856	0.22092
CS	0.70057	0.45255	1.18932	0.78081	0.37488
CS + NIR	2.08279	1.75358	3.17609	2.33749	0.74467

PBS + NIR	0.10474	0.09691	0.01886	0.0735	0.04748
CS	0.91313	0.77815	0.91334	0.86821	0.07799
CS + NIR	1.94201	2.52288	3.38021	2.61503	0.72352
